# Identification and characterization of a novel rodent bocavirus from different rodent species in China

**DOI:** 10.1038/s41426-018-0052-y

**Published:** 2018-03-29

**Authors:** Chi Zhang, Fenglin Song, Leshan Xiu, Yang Liu, Jian Yang, Lisi Yao, Junping Peng

**Affiliations:** 1 Ministry of Health Key Laboratory of Systems Biology of Pathogens, Institute of Pathogen Biology, Chinese Academy of Medical Sciences & Peking Union Medical College, Beijing, 100176 China; 2 Liaoning Entry-Exit Inspection and Quarantine Bureau, Shenyang, 116001 China; 3 Jilin Entry-Exit Inspection and Quarantine Bureau, Changchun, 130062 China; 4 Chinese Academy of Inspection and Quarantine, Beijing, 100176 China

## Abstract

Members in the genus *Bocaparvovirus* are closely related to human health and have a wide host range. The diverse hosts raise the possibility of crossing species barrier, which is a feature of emerging viruses. Among the mammalian hosts, rodents are generally acknowledged to be important reservoirs of emerging viruses. Here, rodent samples collected from six provinces and autonomous regions of China (Liaoning, Inner Mongolia, Tibet, Xinjiang, Guangxi and Yunnan) were used to investigate the prevalence and distribution of bocaparvoviruses. By using next-generation sequencing first, a partial non-structural protein 1 (NS1) gene belonging to a possible novel bocaparvovirus was discovered. Following this, PCR-based screening of NS1 gene was conducted in 485 rodent samples, with 106 positive results found in seven rodent species (*Rattus norvegicus*, *Mus musculus*, *Apodemus agrarius*, *Cricetulus barabensis*, *Rattus flavipectus*, *Rattus rattus* and *Rhombomys opimus*). Finally, six nearly full-length genomes and three complete CDS were obtained and the newly identified bocaparvovirus was tentatively named rodent bocavirus (RoBoV). RoBoV has three ORFs: NS1, NP1, and VP, which are characteristics of bocaparvoviruses. Phylogenetic analyses revealed that porcine bocavirus isolate PBoV-KU14, a member of *Ungulate bocaparvovirus 4*, was the most related virus to RoBoV, with 92.1–92.9% amino acid identities in NS1 protein. Alignments of RoBoV-related sequences showed RoBoV isolates could be classified into two clades, demonstrating an inter-host genetic diversity. The results indicate a potential interspecies transmission of RoBoV between rodents and swine and expand our knowledge on bocaparvoviruses in rodent populations.

## Introduction

Members of the family *Parvoviridae* are small viruses that have an icosahedral, non-enveloped capsid and a linear single-stranded DNA (ssDNA) genome of approximate 5 kb in length. According to the currently available reports on virus taxonomy published by International Committee on Taxonomy of Viruses (ICTV), *Parvoviridae* can be divided into two subfamilies: *Densovirinae* whose members infect invertebrates, and *Parvovirinae* whose members infect vertebrates. *Parvovirinae* contains eight different genera, including *Amdoparvovirus*, *Aveparvovirus*, *Bocaparvovirus*, *Copiparvovirus*, *Dependoparvovirus*, *Erythroparvovirus*, *Protoparvovirus*, and *Tetraparvovirus*^1^. Human bocavirus (HBoV) is one of the four groups of parvoviruses known to infect humans, with the others being parvovirus B19, human parvovirus 4 (PARV4) and adeno-associated viruses^2^. HBoV1 was identified in 2005, until then, bovine parvovirus (BPV) and canine minute virus had been the only two members of the genus *Bocaparvovirus*^3^. After that, three novel human bocaviruses were discovered and the four HBoVs are named HBoV1 to HBoV4 respectively^4–6^. Accumulating evidence has suggested a link between HBoV1 and respiratory tract infections. Unlike HBoV1, HBoV2 to HBoV4 are primarily detected in fecal samples, but the roles they play in gastrointestinal infections are controversial^7^.

Accompanied by advances of molecular biology technology, new members of *Bocaparvovirus* are continuously being discovered and the host range of bocaparvoviruses has expanded greatly. Besides human, bocaparvoviruses are also found in a variety of mammals such as porcine bocavirus (PBoV)^8–10^. feline bocavirus (FBoV)^11^, canine bocavirus (CBoV)^12^, gorilla bocavirus (GBoV)^13,14^, California sea lion bocavirus (CslBoV)^15^, rat bocavirus (RBoV)^16^, dromedary camel bocaparvovirus (DBoV)^17^, bat bocaparvovirus (BtBoV)^18^, mink bocavirus^19^, and rabbit bocaparvovirus^20^.

Rodents are the most diverse species of mammals and are widely distributed in the world, with 2277 species in total^21^. Rodents are in close contact with humans and are the natural reservoir of a variety of emerging viruses, such as hantaviruses, tick-borne encephalitis virus (TBEV), lymphocytic choriomeningitis virus (LCMV) and Lassa virus (LASV)^22^. In this study, rodent samples collected from six provinces and autonomous regions of China were used to investigate the prevalence and distribution of bocaparvoviruses.

## Materials and methods

### Sample collection and DNA extraction

From 2011 to 2017, a total of 485 rodents were captured using specialized baits and mousetraps. The collection sites were chosen as ports of entry and exit located in 9 different cities belonging to 6 provinces and autonomous regions of China (Liaoning, Inner Mongolia, Tibet, Xinjiang, Guangxi and Yunnan) and the capture was conducted by local entry-exit inspection and quarantine bureaus. These sampling sites differ greatly in climate and landform, and the 485 rodents covering 15 different species showed good representatives of rodent diversity throughout corresponding region. All the rodents were dissected in biosafety cabinet and their lungs or spleens were collected. These consisted of 467 lungs and 18 spleens. Disposable scalpels and protective gloves were used to prevent crossover contamination when dissection and collection were conducted. All the samples were collected from the center of the tissue and were stored in liquid nitrogen then transported to Chinese Academy of Inspection and Quarantine for further analyses. Organ specimens were initially suspended in RPMI 1640 medium and then mechanically homogenized with sterile zirconia beads. Finally, viral nucleic acids were extracted from 200 μl of suspension using the TIANamp virus DNA/RNA Kit (TIANGEN BIOTECH, Beijing, China).

### Next-generation sequencing

DNA extracted from eight *Rattus norvegicus* samples in Liaoning Province were pooled and used for library preparation. The library was analyzed by Hiseq 2500 System (Illumina, Inc., San Diego, CA, USA) with paired-end reads of an average 150 bp in length. An in-house Perl script was employed for quality control and reads filtering. BLASTx and BLASTn were then used to align each read passing the quality control with the sequences of NCBI non-redundant protein database and nucleotide database. The reads having hits with an E-value less than 10^−5^ were parsed and extracted by MEGAN 5 software^23^. Extracted reads were de novo assembled by SeqMan software (DNASTAR, Inc., Madison, WI, USA).

### Rodent bocavirus screening and whole-genome sequencing

Nested primers used for screening PCR were designed on the basis of a partial NS1 sequence obtained by NGS. Primers PBNS1-F1 (5′-CCCAGTACAGGAAAGACCAACC-3′) and PBNS2-R1 (5′-GAAGGGCATAACTTAGCCAACG-3′) were designed for the first round of nested PCR and primers PBNS1-F2 (5′-GTAAATCTATTCGGCAATGTGA-3’) and PBNS1-R2 (5′-CATGTAGTGCAGTATCCGTCCA-3′) were designed for the second round of nested PCR, amplifying a 413-bp fragment. We also designed primers to screen rat bocavirus (RBoV). Primers RBoV-HK-F1 (5′-CTACTGGGCATGCGAACGTA-3′) and RBoV-HK-R1 (5′-CAGTTGCCTGTTGGTGTGTG-3′) were designed for the first round of nested PCR. Primers RBoV-HK-F2 (5′-ACAGCAGACAAGCCAACCAA-3′) and RBoV-HK-R2 (5′-TGCATTGTCTTCTGGCTGTCT-3′) were designed for the second round of nested PCR, amplifying a 248-bp fragment. Primers L7 (5′-ACCAATGACATGAAAAATCATCGTT-3′) and H15915 (5′-TCTCCATTTCTGGTTTACAAGAC-3′) targeting the *cytb* (cytochrome b) gene of mitochondrial DNA were used to amplify a 1242 bp fragment to identify the species of rat or rodent^24^. The PCR mixture (25 μl) contained 5 μl DNA template, 200 μM nucleotide mix (dATP, dCTP, dGTP and dTTP), 0.4 μM of forward primer and reverse primer, 1.25U high fidelity polymerase and 2.5 μl 10 × reaction buffer (Roche, Mannheim Germany). The reaction conditions of screening PCR were: 95 °C for 2 min; 35 cycles of 95 °C for 30 s, 55 °C for 30 s, and 72 °C for 1 min; and then a final extension step at 72 °C for 7 min. The reaction conditions of *cytb* PCR were: 95 °C for 2 min; 35 cycles of 95 °C for 30 s, 48 °C for 30 s, and 72 °C for 2 min; and then a final extension step at 72 °C for 7 min. Deionized water was used as negative control and yielded no positive results during the screening stage. PCR products were separated on 1.5% agarose gel followed by purification with QIAquick gel extraction kit (QIAgen, Hilden, Germany). Sanger sequencing was performed by BGI (Beijing, China). Initial whole-genome sequencing was performed using a primer walking strategy described previously^25^. After that, complete genomes were amplified from the positive samples by using ten sets of specific nested primers derived from the full viral genome obtained before. The initial sequences obtained by NGS were also reconfirmed. The primers used for target screening and genome amplifying were listed in Supplementary Table S1. PCR mixture and conditions were same as those of screening PCR. Finally, six nearly full-length genomes and three full CDS were obtained and have been deposited in GenBank under accession no. KY927867-KY927875.

### Sequence alignments and phylogenetic analyses

MegAlign software (DNASTAR Inc., Madison, WI, USA) was used to calculate the nucleotide and amino acid identity of each ORF between sequences obtained in this study and other viruses belonging to the genus *Bocaparvovirus*. The multiple alignments were done by Clustal W program with default parameters. Maximum likelihood (ML) phylogenetic trees based on NS1 amino acid sequences were constructed by MEGA 6 software^26^. Before tree construction, the Modeltest software in MEGA 6 was used to test and determine optimal model-fitting of the sequence data. Models with the lowest BIC (Bayesian Information Criterion) scores are selected. Reliability of the dataset was assessed by bootstrap.

### Analyses of inter-host genetic diversity

By using nested PCR, a partial VP1 sequence was amplified and sequenced to study the inter-host genetic diversity of RoBoV. Primers VP1-F1 (5′-TCCAGAACCTGAGATCCCTCA-3′) and VP1-R1 (5′-TGGTATCAGCTCTGAGTCCCA-3′) were designed for the first round of nested PCR and primers VP1-F2 (5′-TTTGCACGACAAGCACAAGG-3′) and VP1-R2 (5′-TGAATATGGATACTGATGTGAGCCA-3′) were designed for the second round of nested PCR, amplifying a 531-bp fragment. The PCR conditions were same as that of screening PCR. A 460-bp fragment within the amplicon was used to perform the Clustal W multiple alignments and construct the maximum likelihood (ML) phylogenetic tree.

## Results

### Detection of bocaparvoviruses in rodents

According to the results of metagenomic analyses, a partial non-structural protein 1 (*NS1*) gene belonging to a possible novel bocaparvovirus was discovered. Three contigs were obtained by *de novo* assembly and covered 1386 bp of the target. One hundred and six of 485 samples were found to be positive in the following nested PCR screening. All the positive samples were rodents’ lungs and were collected from seven different rodent species (*Rattus norvegicus*, *Mus musculus*, *Apodemus agrarius*, *Cricetulus barabensis*, *Rattus flavipectus*, *Rattus rattus* and *Rhombomys opimus*) in 9 cities (Table 1). The detection rates differed greatly among the sampling sites and the 15 rodent species. Yunnan Province had the highest positive rate (42.4%) followed by Tibet (42.2%), while Inner Mongolia Autonomous Region and Xinjiang Uygur Autonomous Region had a low positive rate (1.4% and 6.7% respectively). *Rattus rattus*, *Rattus flavipectus*, and *Rattus norvegicus* had relatively high detection rates, with positive rates 44.4%, 37.5% and 25.9% respectively. The rodent bocaparvovirus was detected throughout the year but no particular seasonal patterns were observed (Table 2). In addition, rat bocavirus (RBoV) was not detected in our samples.

**Table 1:** Detection rates of rodent bocavirus among different rodent species in Dalian, Dandong, Huludao, Manzhouli, Zhangmu, Hami, Pingxiang, Ruili and Hekou from 2011 to 2017

Scientific name	No. positive/no. tested (%) in each city									Total
	Dalian Liaoning	Dandong Liaoning	Huludao Liaoning	Manzhouli Inner Mongolia	Zhangmu Tibet	Hami Xinjiang	Pingxiang Guangxi	Ruili Yunnan	Hekou Yunnan	
*Rattus norvegicus*	24/86 (27.9)	5/19 (26.3)	3/74 (4.1)	0/5	7/14 (50)	0	2/7 (28.6)	0	26/54 (48.1)	67/259 (25.9)
*Mus musculus*	1/11 (9)	0/6	0/5	0/10	0	0	0	0	1/1 (100)	2/33 (6.1)
*Apodemus agrarius*	0/1	4/35 (11.4)	0	0	0	0	0	0	0	4/36 (11.1)
*Citellus dauricus*	0	0	0	0/23	0	0	0	0	0	0/23
*Meriones unguiculatus*	0	0	0	0/5	0	0	0	0	0	0/5
*Microtus gregalis*	0	0	0	0/2	0	0	0	0	0	0/2
*Cricetulus barabensis*	0	0	0	1/28 (3.6)	0	0	0	0	0	1/28 (3.6)
*Rattus flavipectus*	0	0	0	0	16/38 (42.1)	0	2/4 (50)	8/26 (30.8)	1/4 (25)	27/72 (37.5)
*Rattus rattus*	0	0	0	0	4/9 (44.4)	0	0	0	0	4/9 (44.4)
*Apodemus peninsulae*	0	0	0	0	0/3	0	0	0	0	0/3
*Cricetulus migratorius*	0	0	0	0	0	0/2	0	0	0	0/2
*Tamarisk Jird*	0	0	0	0	0	0/1	0	0	0	0/1
*Meriones meridianus*	0	0	0	0	0	0/5	0	0	0	0/5
*Allactaga sibirica*	0	0	0	0	0	0/1	0	0	0	0/1
*Rhombomys opimus*	0	0	0	0	0	1/6 (16.7)	0	0	0	1/6 (16.7)
Total	25/98 (25.5)	9/60 (15)	3/79 (3.8)	1/73 (1.4)	27/64 (42.2)	1/15 (6.7)	4/11 (36.4)	8/26 (30.8)	28/59 (47.5)	106/485 (21.9)

**Table 2:** Detection rates of rodent bocavirus in different months in in Dalian, Dandong, Huludao, Manzhouli, Zhangmu, Hami, Pingxiang, Ruili and Hekou from 2011 to 2017

Sampling time	No. positive/no. tested (%) in each city									Total
	Dalian Liaoning	Dandong Liaoning	Huludao Liaoning	Manzhouli Inner Mongolia	Zhangmu Tibet	Hami Xinjiang	Pingxiang Guangxi	Ruili Yunnan	Hekou Yunnan	
February	0	0	0	0	14/34 (41.2)	0	0	0	0	14/34 (41.2)
March	0	0	0	0	1/2 (50)	0	0	0	0	1/2 (50)
April	1/7 (14.3)	0/1	0	0	0	0	0	0	15/21 (71.4)	16/29 (55.2)
May	8/34 (23.5)	3/10 (30)	0	0	0	0	0	0	0	11/44 (25)
June	1/3 (33.3)	1/27 (3.7)	0	0/19	0	1/15 (6.7)	0	0	10/20 (50)	13/84 (15.5)
July	0	2/11 (18.2)	0	1/36 (2.8)	8/12 (66.7)	0	0	0	0	11/59 (18.6)
August	4/22 (18.2)	2/7 (28.6)	3/79 (3.8)	0/18	0/6	0	4/11 (36.4)	8/26 (30.8)	3/18 (16.7)	24/187 (12.8)
September	0/12	0/2	0	0	1/4 (25)	0	0	0	0	1/18 (5.6)
October	5/10 (50)	0/1	0	0	0	0	0	0	0	5/11 (45.5)
November	6/8 (75)	1/1 (100)	0	0	0	0	0	0	0	7/9 (77.8)
Not available	0/2	0	0	0	3/6 (50)	0	0	0	0	3/8 (37.5)
Total	25/98 (25.5)	9/60 (15)	3/79 (3.8)	1/73 (1.4)	27/64 (42.2)	1/15 (6.7)	4/11 (36.4)	8/26 (30.8)	28/59 (47.5)	106/485 (21.9)

### Genome analyses of the newly identified bocaparvovirus

**Fig. 1:**
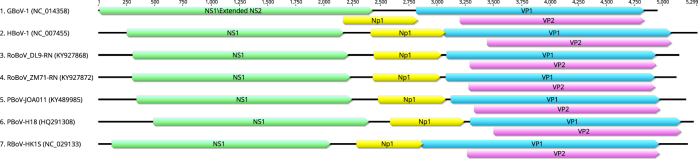
Genomic organization of rodent bocavirus (RoBoV) compared with that of GBoV, HBoV and PBoV. Different colored boxes represent four ORFs of bocaparvoviruses. The illustration was constructed by geneious software. (Biomatters Ltd, Auckland, New Zealand)

For all the screening PCR positive samples, we attempted to amplify the whole genome of virus by using ten sets of nested PCR. Six nearly full-length genomes (lacking fraction of 5′ and 3′ untranslated regions) were obtained from three *Rattus norvegicus* in Dalian, Liaoning (isolate DL8-RN, DL9-RN, DL16-RN), one *Rattus flavipectus*, one *Rattus rattus* and one *Rattus norvegicus* in Zhangmu Tibet (isolate ZM14-RF, ZM37-RR, ZM71-RN respectively). Additionally, full CDS (coding sequences) of three other isolates from *Rattus norvegicus* in Dalian, Liaoning were obtained and named DL7-RN, DL14-RN and DL23-RN. The six genomes contain 5110 to 5140 base pairs and the GC contents range from 40.8% to 41.5% (Table 3). ORF finder (https://www.ncbi.nlm.nih.gov/orffinder) was used to determine ORFs of the six genomes and the three CDS. It was predicted that the six genomes contained three ORFs, which are typical structures of *Bocaparvovirus*. ORF1 encodes the non-structure protein NS1 of 643 aa (isolates ZM14-RF and ZM71-RN) and 635 aa (other seven isolates). Similar to other bocaparvoviruses, ORF2 encodes two overlapping capsid proteins including the larger VP1 and the truncated VP2 but the 616 aa VP1 of our isolates is shorter than that of most bocaparvoviruses. The smaller VP1 results from the shorter unique VP1 (VP1u) region with only 68aa. For all isolates, ORF3 encodes protein NP1 of 199 aa, which is a characteristic feature of bocaparvoviruses (Fig. 1). InterProScan program was used for analysis and classification of protein sequences (http://www.ebi.ac.uk/interpro/). In ORF1, translated products of 1156 to 1659 nt and 1096 to 1674 nt showed features of helicase domain and nucleoside triphosphate hydrolase domain respectively. The VP1u region is short because the phospholipase A2 (PLA2) motif is missing in our sequences within this region. Alignments showing the absence of PLA2 motif can be found in Supplementary Figure S1. We also investigated the possible splicing in NS1 transcripts, which has already been found in human bocavirus and gorilla bocavirus^4,13,27^. The predicted RNA splicing signals and the extended NS1 protein generated by RNA splicing were shown in Supplementary Figure S2.

**Table 3:** Nine isolates of rodent bocavirus identified in this study

Accession no.	Isolate name	Host	Length	GC%	Sequence type
KY927867	DL8-RN	*Rattus norvegicus*	5136	41.2	Nearly full-length genome
KY927868	DL9-RN	*Rattus norvegicus*	5138	41.1	Nearly full-length genome
KY927869	DL16-RN	*Rattus norvegicus*	5140	41.5	Nearly full-length genome
KY927870	ZM14-RF	*Rattus flavipectus*	5118	40.8	Nearly full-length genome
KY927871	ZM37-RR	*Rattus rattus*	5116	41.4	Nearly full-length genome
KY927872	ZM71-RN	*Rattus norvegicus*	5110	41	Nearly full-length genome
KY927873	DL7-RN	*Rattus norvegicus*	4699	41.6	Complete CDS
KY927874	DL14-RN	*Rattus norvegicus*	4850	41.3	Complete CDS
KY927875	DL23-RN	*Rattus norvegicus*	4852	41.3	Complete CDS

### Phylogenetic and recombination analyses of RoBoV

**Fig. 2:**
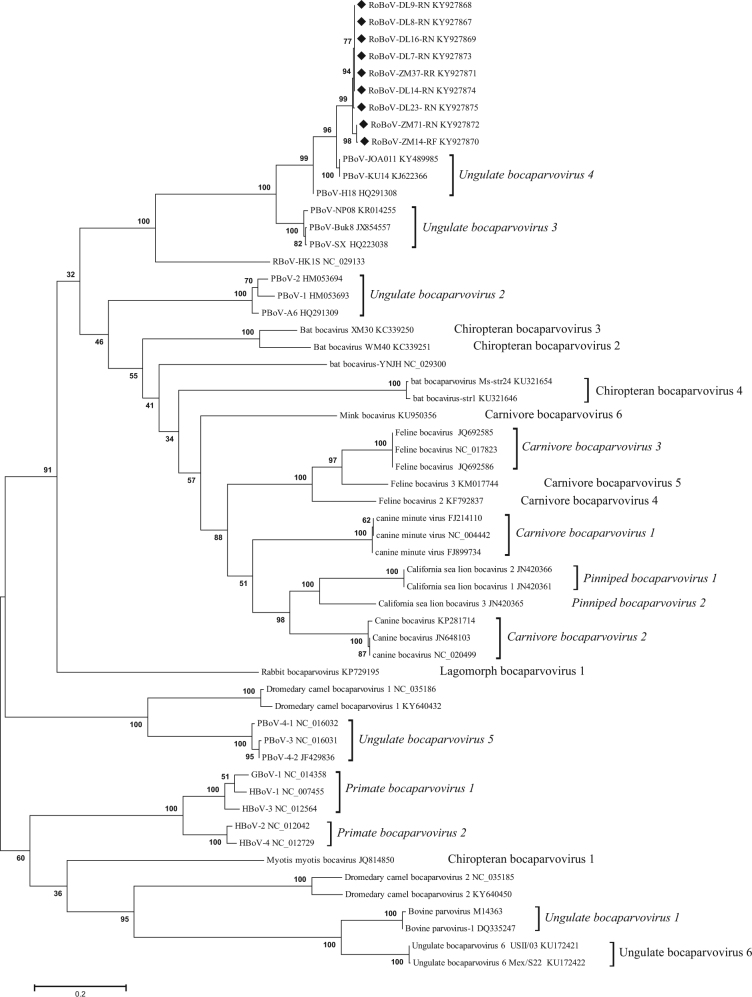
Phylogenetic tree of NS1 amino acid sequences of RoBoV and other members belonging to the genus *Bocaparvovirus*. Maximum likelihood method was used to generate the tree under the best model (LG + G + I), which was selected based on the BIC scores (Supplementary Table S2). Scale bar indicates amino acid substitutions per site. Bootstrap values obtained in 100 replicates are indicated at the nodes. The sequences obtained in this study are marked with a black diamond. The tree is midpoint rooted. Isolate’s name DL8-RN denotes that the isolate’s host is *Rattus norvegicus* which was captured in Dalian. Likewise, the name ZM14-RF denotes this isolate’s host is *Rattus flavipectus* which was captured in Zhangmu. Csl bocavirus California sea lion bocavirus, GBoV gorilla bocavirus, HBoV human bocavirus, PBoV porcine bocavirus, RoBoV rodent bocavirus, RBoV rat bocavirus

To identify the genetic relationship between the newly identified bocaparvovirus and other viruses belonging to the genus *Bocaparvovirus*, we performed both nucleotide and amino acid sequence alignments. Alignments were shown in Supplementary Figure S3 and S4. Genomes of our study possess 92.1–92.9% amino acid identities (87.9–88% nucleotide identities) in NS1, between the most closely related bocaparvovirus, porcine bocavirus isolate PBoV-KU14, but are quite divergent with rat bocavirus described in Hong Kong^16^, with the NS1 amino acid identity only 50.9–51.4% (Table 4). The results demonstrated that the novel virus might be a new bocaparvovirus species in rodents and we provisionally named it rodent bocavirus (RoBoV). The maximum likelihood trees based on NS1 amino acid sequences showed that RoBoV was most closely related to *Ungulate bocaparvovirus 4* but formed a distinct clade (Fig. 2). As bocaparvoviruses are highly recombination prone, we compared genome wide distances among RoBoV and PBoV-H18, PBoV-JOA11, rat bocavirus RBoV-HK1S but did not find evidence of recombination (data not shown).

**Table 4:** Amino acid and nucleotide (data in parentheses) identities of NS1, NP1, VP1 between RoBoV isolates DL9-RN, ZM71-RN and other bocaparvoviruses

Isolate	DL9-RN	ZM71-RN	PBoV- KU14	PBoV- H18	PBoV- JOA011	PBoV-2	PBoV- SX	PBoV-3	RBoV- HK1S	HBoV-1
DL9-RN										
NS1	—	97.5 (94.9)	92.1 (88)	88.7 (84.9)	92 (87.7)	48.3 (54.9)	75.6 (72)	39.8 (49.5)	51.4 (56.7)	41.7 (49.4)
NP1	—	96.5 (95.2)	86.4 (86.5)	65.2 (72.7)	91.3 (88.9)	40.7 (53.5)	65.2 (73)	31.8 (45.4)	33.2 (52.6)	29.4 (53.6)
VP1	—	99.5 (94.3)	91.4 (86.8)	75.8 (73.3)	91.6 (86.9)	46.6 (52.9)	75.1 (73.1)	38 (48.7)	45.8 (52.9)	40.1 (52.1)
ZM71-RN										
NS1	97.5 (94.9)	—	92.9 (87.9)	89.3 (84.9)	92.8 (87.9)	47.5 (54.1)	75.4 (71.9)	39.5 (49)	50.9 (56.7)	41.4 (49.3)
NP1	96.5 (95.2)	—	87.9 (86.3)	64.1 (72.4)	91.9 (88)	42.2 (51.9)	64.1 (72.4)	32.3 (45.1)	33.2 (52.2)	29.9 (53.4)
VP1	99.5 (94.3)	—	91.6 (87.3)	75.9 (73.8)	91.7 (87.5)	46.6 (52.6)	75.3 (73.6)	38.1 (48.7)	46 (53.3)	40.2 (52)

*HBoV* human bocavirus, *PBoV* porcine bocavirus, *RBoV* rat bocavirus

### Inter-host genetic diversity of RoBoV

**Fig. 3:**
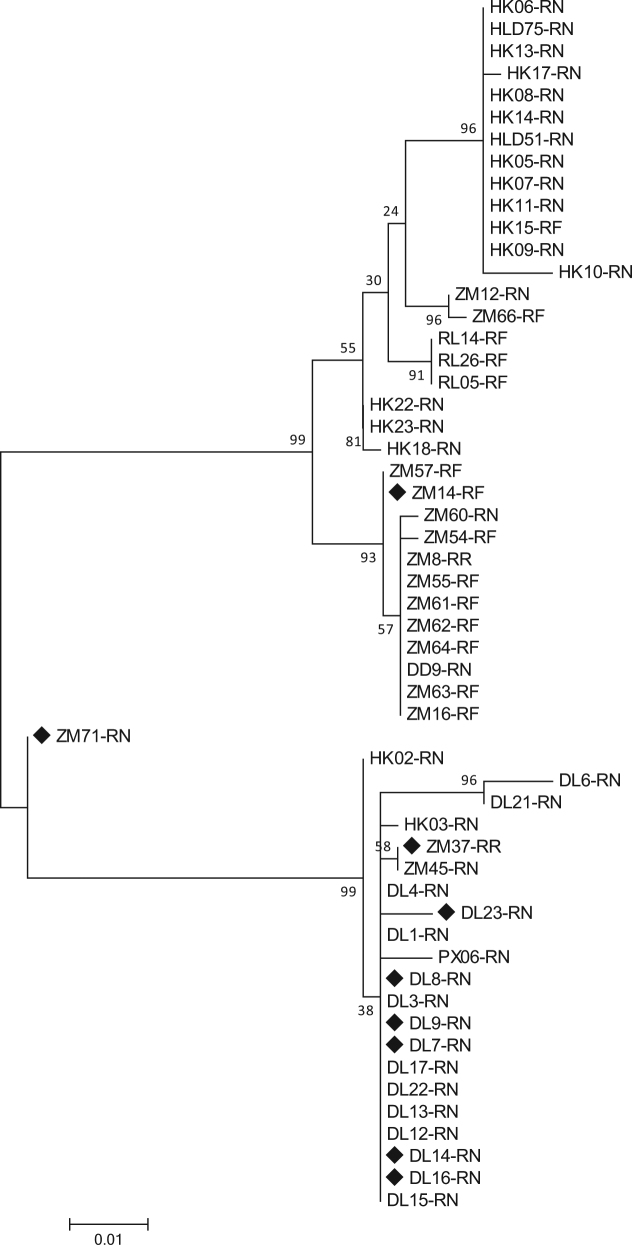
Phylogenetic tree of partial VP1 nucleotide sequences (460-bp fragment) from 55 RoBoV isolates showing the genetic diversity. The maximum likelihood tree was constructed under the best model (HKY + G), which was selected based on the BIC scores (Supplementary Table S3). Scale bar indicates nucleotide substitutions per site. Bootstrap values obtained in 100 replicates are indicated at the nodes. The nine isolates with genome or CDS sequenced are marked with a black diamond. The tree is midpoint rooted. DL, DD, HLD, ZM, HM, MZL, PX, RL and HK in isolates’ name represent samples collected from Dalian, Dandong, Huludao, Zhangmu, Hami, Manzhouli, Pingxiang, Ruili, and Hekou respectively. Suffixes of isolates’ name denote the host species: AA *Apodemus agrarius*, CB *Cricetulus barabensis*, MM *Mus musculus*, RN *Rattus norvegicus*, RF *Rattus flavipectus*, RO *Rhombomys opimus*, RR *Rattus rattus*

As the capsid proteins are important determinants for the host tropism and tissue tropism of parvoviruses, the VP1 sequence was selected to perform the phylogenetic analysis, thereby determining the inter-host genetic diversity of RoBoV. Multiple alignments based on 460-bp partial VP1 fragments from 55 different RoBoV isolates showed that they possessed up to 10.7% nucleotide difference to each other. Alignments were shown in Supplementary Figure S5. The maximum likelihood tree demonstrated that the 55 isolates can be classified into two clades. Isolates sampling from adjacent regions were prone to form a cluster whereas the divergence among isolates from different rodent species was not significant (Fig. 3).

## Discussion

In this study, we identified a novel bocaparvovirus, RoBoV and characterized its genomic features. Then we conducted a large-scale study to screen RoBoV in China and acquired the prevalence of RoBoV among different rodent species. We obtained six nearly full-length genomes and three complete CDS of RoBoV. As some samples were archived several years before, the limited sample volume and degraded DNA prevented us from obtaining more genomes.

A stricter criterion used for species demarcation in the family *Parvoviridae* has been proposed, because the former one is so lenient that will lead to much confusion. The new proposal requires viruses belonging to a species to have more than 85% amino acid sequence identity of the NS1 protein^1^. Subject to the new criteria, RoBoV should be classified as a new member of species *Ungulate bocaparvovirus 4* together with porcine bocavirus PBoV-H18. Moreover, besides sequence identity, important factors such as host species, antigenic properties and genome features should be taken into consideration as well when characterizing a species. Although the antigenicity of RoBoV remains to be discovered, considering that its hosts are rodents and the virus shares an amino acid identity of 50.9% to 51.4% between the only known rat bocavirus RBoV-HK1S, we speculate that the virus is a novel species in rodent population.

A similar study was conducted by Lau et al.^16^ in China recently. In the study, rats were collected in Guangdong Province and Hong Kong, where a novel rat bocavirus (RBoV) was discovered. The study first identified and sequenced rat bocavirus and described its prevalence in southern China among different rodent species. Revealed by phylogenetic analyses and sequence alignments, RoBoV and RBoV are located in distinctly different branches of the evolutionary tree and they possess low sequence identity, which indicates that diverse species of bocaparvoviruses exist in rodent population. For RoBoV, the detection rate in respiratory samples was higher than that of RBoV, suggesting a possible difference of tissue tropism between the two viruses. Moreover, the host of RBoV is likely limited to *Rattus norvegicus*, whereas RoBoV probably infects various species of rodents. The natural habitats of *Rattus norvegicus* (brown rat) cover wide geographical areas across China, but RBoV was not detected in our samples. Given that our samples were collected in China’s northeast and western regions, this phenomenon may owe to the distribution discrepancy of bocaparvoviruses. To better elucidate the tissue tropism, host range, and distribution of bocaparvoviruses in rodent population, as well as discovering new species, more epidemiological studies should be conducted.

It is noteworthy that the closest neighbor of RoBoV is *Ungulate bocaparvovirus 4* whose members are all porcine bocaviruses. The first member of *Ungulate bocaparvovirus 4* is porcine bocavirus isolate H18 (PBoV-H18) identified in China^10^ and other members include isolate KU14 from South Korea^28^, isolate S1142/13 from Germany^29^ and isolates JOA011, JOA015 from East Africa^30^. Besides sequence identity, RoBoV possesses some genomic features which have already found in different species of porcine bocavirus. RoBoV contains a short NP1 protein of 199 amino acids, same as that of isolate PBoV-KU14. The conserved RNA splicing signals and a very short VP1u region of 68 amino acids are characteristics of isolate PBoV-SX (now classified as *Ungulate bocaparvovirus 3*)^31^.

Previous researches have revealed that VP1u was critical for the life cycle of parvovirus. The VP1u-associated PLA2 activity is required by parvovirus for its release from endosome^32^. The activity of PLA2 has been proved in parvovirus B19^33^ and HBoV1^34^ and PLA2-related motif has been identified in some newly discovered parvoviruses^8,11,14,16,35^. However, in nine sequenced isolates of RoBoV, such motif was absent from the amino acid sequences of VP1 and other proteins. Furthermore, the motif was also absent from isolates PBoV-H18, PBoV-KU14 and PBoV-SX, which show high-sequence identity with RoBoV and share short VP1u sequences of 51–69 aa. In dromedary camel bocavirus 2 (DBoV2), such events are also observed^17^, so it is suggested that lack of PLA2 motif and shorter VP1u are characteristics of some species in the genus *Bocaparvovirus*. As to these parvoviruses, the mechanism of release from endosome and therefore how this affect their transmission are needed to be verified by further experiments.

The fact that isolates of RoBoV can be classified into two clades indicates an inter-host genetic diversity. Recombination events have been detected frequently in human bocavirus and porcine bocavirus^6,9,36,37^. Although no clear evidence showed that recombination existed in RoBoV, the inter-host genetic diversity identified in the present study demonstrated that RoBoV might evolve rapidly. Some studies have already shown that parvoviruses had a mutation rate close to that of single-stranded RNA viruses^38–41^. Importantly, recently discovered bat bocaparvoviruses Rs-BtBoV2 and Rol-BtBoV1 were closely related to PBoVs belonging to *Ungulate bocaparvovirus 5* and *Ungulate bocaparvovirus 2* respectively, which suggests an interspecies transmission of BtBoV between bats and swine. Likewise, these BtBoVs were also detected from multiple bat species^35^. Considering the possibly high mutation rate of RoBoV and the above evidence, we speculate that during the process of occurrence and transmission of RoBoV, overcoming the interspecies barrier has taken place. Meanwhile, *Ungulate bocaparvovirus 4* has been found on three different continents and on this regard, whether RoBoV has the probability of intercontinental prevalence, should be confirmed by worldwide epidemiological studies.

The pathogenicity of RoBoV remains to be determined. Bocaparvoviruses can infect different hosts and cause dissimilar syndromes. BPV is the first discovered bocaparvovirus, which was found in the gastrointestinal tract of diarrheal calves^42^. BPV was also reported to be associated with reproductive disorders^43^. Canine minute virus (CnMV) can cause enteritis and breathing difficulty in newborn puppies^44,45^. More evidence has been provided to support HBoV1 an indeed etiological pathogen, but for HBoV2-4, the roles they play in human diseases are still pending^7^. Following the discovery of four HBoV, the first non-human primate bocavirus, GBoV1 was identified in fecal samples from African gorillas suffering diarrhea^13^. As to PBoV, although have been detected in clinically sick pigs or piglet, the commonly reported co-infection with other viruses confused the issue further^46^. For other bocaparvoviruses, CslBoV, FBoV, and RBoV, the relationship between infection and symptom has not been clarified yet^11,15,16,47^. Similarly, to fully understand the pathogenicity of RoBoV, more studies are required.

On epidemiology, the geographical distribution of RoBoV is not confined to one region of China and its host range includes at least seven species of rodents. The results suggest that RoBoV can adapt to different climates and hosts. On sequence and evolution, RoBoV shows genetic diversity and potentially underwent host transferring between rodents and swine. All these features make RoBoV a remarkable bocaparvovirus, so persistent surveillance should be performed to monitor its transmission and evolution.

## Electronic supplementary material

Supplementary Table S1(PDF 79 kb)

Supplementary Table S2(PDF 759 kb)

Supplementary Table S3(PDF 141 kb)

Supplementary Figure S1(PDF 109 kb)

Supplementary Figure S2(PDF 625 kb)

Supplementary Figure S3(PDF 2733 kb)

Supplementary Figure S4(PDF 3041 kb)

Supplementary Figure S5(PDF 758 kb)
