# Supplementary material for: Identification and characterization of a novel rodent bocavirus from different rodent species in China
Source: Emerg Microbes Infect. 2018 Mar 29;7:48. doi: 10.1038/s41426-018-0052-y (PMC5874251; doi:10.1038/s41426-018-0052-y)
Supplement: Supplementary file 1 — Supplementary Table S1(PDF 79 kb) [file 41426_2018_52_MOESM1_ESM.pdf]

Supplementary Material

Identification and characterization of a novel rodent bocavirus from different rodent species in China

Chi Zhang[1], Fenglin Song[1], Leshan Xiu[1], Yang Liu, Jian Yang, Lisi Yao[2], Junping Peng[2]

[1]These authors contributed equally to this article.

[2]These authors are both corresponding authors.

Correspondence:

Junping Peng, pengjp@hotmail.com

Lisi Yao, yaolisi@hotmail.com

Supplementary information

Table S1, primers used for target screening and genome amplifying

Table S1. Primers used for target screening and genome amplifying. Target region was calculated based on the full genome of RoBoV isolate DL8-RN (KY927867).

Name	Sequence	Target Region	Description
PBNS1-F1	CCCAGTACAGGAAAGACCAACC	1626-2038	Primers used for targeting screening.
PBNS2-R1	GAAGGGCATAACTTAGCCAACG		
PBNS1-F2	GTAAATCTATTCGGCAATGTGA		
PBNS1-R2	CATGTAGTGCAGTATCCGTCCA		
5UTR1-F	AAGGCGGGGCGATATAAGCA	1-495	Ten sets of specific nested primers used for genome amplifying.
5UTR1-R-OUT	TGCTGATTTTCAAACACCTTTC		
5UTR1-R-IN	CATTTGGAAATTCTGGATAGTGT		
5UTR2-F	CAATCAACCTGGTGGTGAGTAA	282-495	
5UTR2-R-OUT	TGCTGATTTTCAAACACCTTTC		
5UTR2-R-IN	CATTTGGAAATTCTGGATAGTGT		
N1-F-OUT	TTTCCTAAATTTCCTACGGATGACT	418-1316	
N1-R-OUT	CACCTGCTTGGCTTTCCATC		
N1-F-IN	AGTGCTTAGGAGATAGCGAAAACA		
N1-R-IN	TTCCGGGTGTTTGTCTACTAATTG		

N2-F-OUT	TGGCTGGAAGATTTGCATGG	1133-2057
N2-R-OUT	CCTGCGAATGAGAAGGGCAT	
N2-F-IN	CGAGCCGGTCTTTACTGGAG	
N2-R-IN	GGTCACGTTCTTCGTTTTCCA	
N3-F-OUT	CCGCAGACTCCATGCATCA	1850-2821
N3-R-OUT	AAAATGCCACATCATGCTTCTGT	
N3-F-IN	CGAGTGTGTAGGTGGAAACCA	
N3-R-IN	ACCCATTCCACCTTCCCTTC	
N4-F-OUT	AACAAAAGGCCAGGGAGGAT	2642-3563
N4-R-OUT	AGCCTCTGCCAGTCATTTGG	
N4-F-IN	ACAACGAGTTAGCGTGGTAGA	
N4-R-IN	ACCCCCATGGAGTCCTGATT	
N5-F-OUT	GCTAAGAGGGCCAGAATGGG	3305-4220
N5-R-OUT	AATGCTGGTGAGCCCATGTA	
N5-F-IN	GGGGAAACAGACGATGTGGT	
N5-R-IN	TGAATGCCCCTGCTGATGTT	

N6-F-OUT	ACATAAGGGAAACATGGCTAGGG	
N6-R-OUT	ATGAAATTCTGAGCTCTGGTCTCC	3984-4817
N6-F-IN	TTGAATTTGACTTCGATTGCGGT	
N6-R-IN	AAATTCTGAGCTCTGGTCTCCAA	
3UTR2-F-OUT	GGGAACAGTATGGAGTCAAGCC	
3UTR2-F-IN	GGGACAAACAACCAAATACAGA	4584-4985
3UTR2-R	AATGGCGTCGTGGTTGTTGTAT	
3UTR1-F-OUT	GGGAACAGTATGGAGTCAAGCC	
3UTR1-F-IN	GGGACAAACAACCAAATACAGA	4584-5133
3UTR1-R	ATGTTATATTACCGGCGCACAT	
