# Supplementary material for: Identification and characterization of a novel rodent bocavirus from different rodent species in China
Source: Emerg Microbes Infect. 2018 Mar 29;7:48. doi: 10.1038/s41426-018-0052-y (PMC5874251; doi:10.1038/s41426-018-0052-y)
Supplement: Supplementary file 2 — Supplementary Table S2(PDF 759 kb) [file 41426_2018_52_MOESM2_ESM.pdf]

Supplementary Material

Identification and characterization of a novel rodent bocavirus from different rodent species in China

Chi Zhang[1], Fenglin Song[1], Leshan Xiu[1], Yang Liu, Jian Yang, Lisi Yao[2], Junping Peng[2]

[1]These authors contributed equally to this article.

[2]These authors are both corresponding authors.

Correspondence:

Junping Peng, pengjp@hotmail.com

Lisi Yao, yaolisi@hotmail.com

Supplementary information

Table S2. Maximum Likelihood fits of 48 different amino acid substitution models of phylogenetic analyses of NS1 amino acid sequences

Table S2. Maximum Likelihood fits of 48 different amino acid substitution models of phylogenetic analyses of NS1 amino acid sequences

Model	#Param	BIC	AICc	lnL	Invariant	Gamma	Freq A	Freq R	Freq N	Freq D	Freq C	Freq Q	Freq E	Freq G	Freq H	Freq I	Freq L	Freq K	Freq M	Freq F	Freq P	Freq S	Freq T	Freq W	Freq Y	Freq V
LG+G+I	113	33140.50819	32221.58132	-15997.2805	0.11717063	1.121845545	0.079066	0.055941	0.041977	0.053052	0.012937	0.040767	0.071586	0.057337	0.022355	0.062157	0.099081	0.0646	0.022951	0.042302	0.04404	0.061197	0.053287	0.012066	0.034155	0.069147
LG+G+I+F	132	33233.22689	32159.98989	-15947.29916	0.116293703	1.135713697	0.052868125	0.040528287	0.062172285	0.04691504	0.040410014	0.048294895	0.054760497	0.054445102	0.031894343	0.052434457	0.098009068	0.070411985	0.025744136	0.040331165	0.042933176	0.049911295	0.065720481	0.023575793	0.036428149	0.062211709
rtREV+G+I	113	33247.21301	32328.28614	-16050.63291	0.117278687	1.174313611	0.0646	0.0453	0.0376	0.0422	0.0114	0.0606	0.0607	0.0639	0.0273	0.0679	0.1018	0.0751	0.015	0.0287	0.0681	0.0488	0.0622	0.0251	0.0318	0.0619
rtREV+G+I+F	132	33314.59123	32241.35422	-15987.98133	0.115218394	1.14139686	0.052868125	0.040528287	0.062172285	0.04691504	0.040410014	0.048294895	0.054760497	0.054445102	0.031894343	0.052434457	0.098009068	0.070411985	0.025744136	0.040331165	0.042933176	0.049911295	0.065720481	0.023575793	0.036428149	0.062211709
rtREV+G+F	131	33322.59956	32257.4827	-15997.05606	n/a	0.713305525	0.052868125	0.040528287	0.062172285	0.04691504	0.040410014	0.048294895	0.054760497	0.054445102	0.031894343	0.052434457	0.098009068	0.070411985	0.025744136	0.040331165	0.042933176	0.049911295	0.065720481	0.023575793	0.036428149	0.062211709
LG+G	112	33333.83318	32423.0295	-16099.01356	n/a	1.654905724	0.079066	0.055941	0.041977	0.053052	0.012937	0.040767	0.071586	0.057337	0.022355	0.062157	0.099081	0.0646	0.022951	0.042302	0.04404	0.061197	0.053287	0.012066	0.034155	0.069147
LG+G+F	131	33421.86165	32356.74479	-16046.6871	n/a	1.654905724	0.052868125	0.040528287	0.062172285	0.04691504	0.040410014	0.048294895	0.054760497	0.054445102	0.031894343	0.052434457	0.098009068	0.070411985	0.025744136	0.040331165	0.042933176	0.049911295	0.065720481	0.023575793	0.036428149	0.062211709
WAG+G+I	113	33441.36897	32522.4421	-16147.71089	0.124941924	1.318133266	0.0866279	0.043972	0.0390894	0.0570451	0.0193078	0.0367281	0.0580589	0.0832518	0.0244313	0.048466	0.086209	0.0620286	0.0195027	0.0384319	0.0457631	0.0695179	0.0610127	0.0143859	0.0352742	0.0708956
rtREV+G	112	33453.74616	32542.94247	-16158.97005	n/a	1.688507014	0.0646	0.0453	0.0376	0.0422	0.0114	0.0606	0.0607	0.0639	0.0273	0.0679	0.1018	0.0751	0.015	0.0287	0.0681	0.0488	0.0622	0.0251	0.0318	0.0619
WAG+G	112	33462.64157	32551.83789	-16163.41776	n/a	0.778426837	0.0866279	0.043972	0.0390894	0.0570451	0.0193078	0.0367281	0.0580589	0.0832518	0.0244313	0.048466	0.086209	0.0620286	0.0195027	0.0384319	0.0457631	0.0695179	0.0610127	0.0143859	0.0352742	0.0708956
WAG+G+I+F	132	33501.40719	32428.17018	-16081.38931	0.122627845	1.303960991	0.052868125	0.040528287	0.062172285	0.04691504	0.040410014	0.048294895	0.054760497	0.054445102	0.031894343	0.052434457	0.098009068	0.070411985	0.025744136	0.040331165	0.042933176	0.049911295	0.065720481	0.023575793	0.036428149	0.062211709
WAG+G+F	131	33520.09589	32454.97903	-16095.80422	n/a	0.777964175	0.052868125	0.040528287	0.062172285	0.04691504	0.040410014	0.048294895	0.054760497	0.054445102	0.031894343	0.052434457	0.098009068	0.070411985	0.025744136	0.040331165	0.042933176	0.049911295	0.065720481	0.023575793	0.036428149	0.062211709
JTT+G+I	113	33639.85552	32720.92865	-16246.95416	0.126396865	1.228214219	0.0769	0.0511	0.0425	0.0513	0.0203	0.0411	0.0618	0.0747	0.023	0.0526	0.0911	0.0595	0.0234	0.0405	0.0505	0.0682	0.0585	0.0143	0.0323	0.0664
JTT+G	112	33657.153	32746.34931	-16260.67347	n/a	0.723622582	0.0769	0.0511	0.0425	0.0513	0.0203	0.0411	0.0618	0.0747	0.023	0.0526	0.0911	0.0595	0.0234	0.0405	0.0505	0.0682	0.0585	0.0143	0.0323	0.0664
JTT+G+I+F	132	33694.70071	32621.46371	-16178.03607	0.124897199	1.248295012	0.052868125	0.040528287	0.062172285	0.04691504	0.040410014	0.048294895	0.054760497	0.054445102	0.031894343	0.052434457	0.098009068	0.070411985	0.025744136	0.040331165	0.042933176	0.049911295	0.065720481	0.023575793	0.036428149	0.062211709
JTT+G+F	131	33705.52432	32640.40746	-16188.51844	n/a	0.726532213	0.052868125	0.040528287	0.062172285	0.04691504	0.040410014	0.048294895	0.054760497	0.054445102	0.031894343	0.052434457	0.098009068	0.070411985	0.025744136	0.040331165	0.042933176	0.049911295	0.065720481	0.023575793	0.036428149	0.062211709
Dayhoff+G+I+F	132	33810.33849	32737.10148	-16235.85496	0.123266941	1.218187796	0.052868125	0.040528287	0.062172285	0.04691504	0.040410014	0.048294895	0.054760497	0.054445102	0.031894343	0.052434457	0.098009068	0.070411985	0.025744136	0.040331165	0.042933176	0.049911295	0.065720481	0.023575793	0.036428149	0.062211709
Dayhoff+G+F	131	33832.33652	32767.21966	-16251.92454	n/a	0.710707762	0.052868125	0.040528287	0.062172285	0.04691504	0.040410014	0.048294895	0.054760497	0.054445102	0.031894343	0.052434457	0.098009068	0.070411985	0.025744136	0.040331165	0.042933176	0.049911295	0.065720481	0.023575793	0.036428149	0.062211709
Dayhoff+G+I	113	33883.13053	32964.20366	-16368.59167	0.124294335	1.205470495	0.0871	0.0409	0.0404	0.0469	0.0335	0.0383	0.0495	0.0886	0.0336	0.0369	0.0854	0.0805	0.0148	0.0398	0.0507	0.0696	0.0585	0.0105	0.0299	0.0647
Dayhoff+G	112	33906.32739	32995.52371	-16385.26067	n/a	0.694370112	0.0871	0.0409	0.0404	0.0469	0.0335	0.0383	0.0495	0.0886	0.0336	0.0369	0.0854	0.0805	0.0148	0.0398	0.0507	0.0696	0.0585	0.0105	0.0299	0.0647
cpREV+G+I	113	33914.90459	32995.97772	-16384.4787	0.14584568	1.115286637	0.0756	0.0621	0.041	0.0371	0.0091	0.0382	0.0495	0.0838	0.0246	0.0806	0.1011	0.0504	0.022	0.0506	0.0431	0.0622	0.0543	0.0181	0.0307	0.066
cpREV+G	112	33927.30043	33016.49675	-16395.74718	n/a	0.704955849	0.0756	0.0621	0.041	0.0371	0.0091	0.0382	0.0495	0.0838	0.0246	0.0806	0.1011	0.0504	0.022	0.0506	0.0431	0.0622	0.0543	0.0181	0.0307	0.066
cpREV+G+I+F	132	34002.88799	32929.65098	-16332.12971	0.152376324	1.12053375	0.052868125	0.040528287	0.062172285	0.04691504	0.040410014	0.048294895	0.054760497	0.054445102	0.031894343	0.052434457	0.098009068	0.070411985	0.025744136	0.040331165	0.042933176	0.049911295	0.065720481	0.023575793	0.036428149	0.062211709
cpREV+G+F	131	34016.43227	32951.31541	-16343.97241	n/a	0.697726277	0.052868125	0.040528287	0.062172285	0.04691504	0.040410014	0.048294895	0.054760497	0.054445102	0.031894343	0.052434457	0.098009068	0.070411985	0.025744136	0.040331165	0.042933176	0.049911295	0.065720481	0.023575793	0.036428149	0.062211709
LG+I	112	34274.80215	33363.99846	-16569.49804	0.15842614	n/a	0.079066	0.055941	0.041977	0.053052	0.012937	0.040767	0.071586	0.057337	0.022355	0.062157	0.099081	0.0646	0.022951	0.042302	0.04404	0.061197	0.053287	0.012066	0.034155	0.069147
mtREV24+G+I+F	132	34313.39032	33240.15332	-16487.38088	0.114642802	1.061687216	0.052868125	0.040528287	0.062172285	0.04691504	0.040410014	0.048294895	0.054760497	0.054445102	0.031894343	0.052434457	0.098009068	0.070411985	0.025744136	0.040331165	0.042933176	0.049911295	0.065720481	0.023575793	0.036428149	0.062211709
mtREV24+G+F	131	34326.9921	33261.87524	-16499.25233	n/a	0.653603575	0.052868125	0.040528287	0.062172285	0.04691504	0.040410014	0.048294895	0.054760497	0.054445102	0.031894343	0.052434457	0.098009068	0.070411985	0.025744136	0.040331165	0.042933176	0.049911295	0.065720481	0.023575793	0.036428149	0.062211709
rtREV+I	112	34338.73825	33427.93457	-16601.4661	0.157004241	n/a	0.0646	0.0453	0.0376	0.0422	0.0114	0.0606	0.0607	0.0639	0.0273	0.0679	0.1018	0.0751	0.015	0.0287	0.0681	0.0488	0.0622	0.0251	0.0318	0.0619
LG+I+F	131	34347.61583	33282.49897	-16509.56419	0.15812588	n/a	0.052868125	0.040528287	0.062172285	0.04691504	0.040410014	0.048294895	0.054760497	0.054445102	0.031894343	0.052434457	0.098009068	0.070411985	0.025744136	0.040331165	0.042933176	0.049911295	0.065720481	0.023575793	0.036428149	0.062211709
WAG+I	112	34394.53719	33483.73351	-16629.36557	0.158409039	n/a	0.0866279	0.043972	0.0390894	0.0570451	0.0193078	0.0367281	0.0580589	0.0832518	0.0244313	0.048466	0.086209	0.0620286	0.0195027	0.0384319	0.0457631	0.0695179	0.0610127	0.0143859	0.0352742	0.0708956
rtREV+I+F	131	34422.80607	33357.6892	-16547.15931	0.157176303	n/a	0.052868125	0.040528287	0.062172285	0.04691504	0.040410014	0.048294895	0.054760497	0.054445102	0.031894343	0.052434457	0.098009068	0.070411985	0.025744136	0.040331165	0.042933176	0.049911295	0.065720481	0.023575793	0.036428149	0.062211709
WAG+I+F	131	34475.8677	33410.75084	-16573.69013	0.157220726	n/a	0.052868125	0.040528287	0.062172285	0.04691504	0.040410014	0.048294895	0.054760497	0.054445102	0.031894343	0.052434457	0.098009068	0.070411985	0.025744136	0.040331165	0.042933176	0.049911295	0.065720481	0.023575793	0.036428149	0.062211709
JTT+I	112	34690.20466	33779.40097	-16777.1993	0.158650517	n/a	0.0769	0.0511	0.0425	0.0513	0.0203	0.0411	0.0618	0.0747	0.023	0.0526	0.0911	0.0595	0.0234	0.0405	0.0505	0.0682	0.0585	0.0143	0.0323	0.0664
JTT+I+F	131	34730.18183	33665.06497	-16700.84719	0.158557071	n/a	0.052868125	0.040528287	0.062172285	0.04691504	0.040410014	0.048294895	0.054760497	0.054445102	0.031894343	0.052434457	0.098009068	0.070411985	0.025744136	0.040331165	0.042933176	0.049911295	0.065720481	0.023575793	0.036428149	0.062211709
Dayhoff+I+F	131	34907.98837	33842.87151	-16789.75046	0.154771634	n/a	0.052868125	0.040528287	0.062172285	0.04691504	0.040410014	0.048294895	0.054760497	0.054445102	0.031894343	0.052434457	0.098009068	0.070411985	0.025744136	0.040331165	0.042933176	0.049911295	0.065720481	0.023575793	0.036428149	0.062211709
mtREV24+G+I	113	34961.34672	34042.41985	-16907.69977	0.099612908	0.956970924	0.072	0.019	0.039	0.019	0.006	0.025	0.024	0.056	0.028	0.087	0.168	0.023	0.053	0.06	0.055	0.072	0.088	0.029	0.033	0.044
mtREV24+G	112	34962.78494	34051.98125	-16913.48944	n/a	0.627599139	0.072	0.019	0.039	0.019	0.006	0.025	0.024	0.056	0.028	0.087	0.168	0.023	0.053	0.06	0.055	0.072	0.088	0.029	0.033	0.044
Dayhoff+I	112	34966.41511	34055.61143	-16915.30453	0.155928794	n/a	0.0871	0.0409	0.0404	0.0469	0.0335	0.0383	0.0495	0.0886	0.0336	0.0369	0.0854	0.0805	0.0148	0.0398	0.0507	0.0696	0.0585	0.0105	0.0299	0.0647
cpREV+I	112	34983.63064	34072.82695	-16923.91229	0.159550562	n/a	0.0756	0.0621	0.041	0.0371	0.0091	0.0382	0.0495	0.0838	0.0246	0.0806	0.1011	0.0504	0.022	0.0506	0.0431	0.0622	0.0543	0.0181	0.0307	0.066
cpREV+I+F	131	35060.59619	33995.47933	-16866.05437	0.159550562	n/a	0.052868125	0.040528287	0.062172285	0.04691504	0.040410014	0.048294895	0.054760497	0.054445102	0.031894343	0.052434457	0.098009068	0.070411985	0.025744136	0.040331165	0.042933176	0.049911295	0.065720481	0.023575793	0.036428149	0.062211709
LG	111	35138.81658	34236.13624	-17006.57582	n/a	n/a	0.079066	0.055941	0.041977	0.053052	0.012937	0.040767	0.071586	0.057337	0.022355	0.062157	0.099081	0.0646	0.022951	0.042302	0.04404	0.061197	0.053287	0.012066	0.034155	0.069147
rtREV	111	35190.43407	34287.75373	-17032.38457	n/a	n/a	0.0646	0.0453	0.0376	0.0422	0.0114	0.0606	0.0607	0.0639	0.0273	0.0679	0.1018	0.0751	0.015	0.0287	0.0681	0.0488	0.0622	0.0251	0.0318	0.0619
LG+F	130	35206.85601	34149.85946	-16944.25484	n/a	n/a	0.052868125	0.040528287	0.062172285	0.04691504	0.040410014	0.048294895	0.054760497	0.054445102	0.031894343	0.052434457	0.098009068	0.070411985	0.025744136	0.040331165	0.042933176	0.049911295	0.065720481	0.023575793	0.036428149	0.062211709
WAG	111	35246.4996	34343.81926	-17060.41733	n/a	n/a	0.0866279	0.043972	0.0390894	0.0570451	0.0193078	0.0367281	0.0580589	0.0832518	0.0244313	0.048466	0.086209	0.0620286	0.0195027	0.0384319	0.0457631	0.0695179	0.0610127	0.0143859	0.0352742	0.0708956
rtREV+F	130	35271.42292	34214.42636	-16976.5383	n/a	n/a	0.052868125	0.040528287	0.062172285	0.04691504	0.040410014	0.048294895	0.054760497	0.054445102	0.031894343	0.052434457	0.098009068	0.070411985	0.025744136	0.040331165	0.042933176	0.049911295	0.065720481	0.023575793	0.036428149	0.062211709
WAG+F	130	35310.26092	34253.26437	-16995.9573	n/a	n/a	0.052868125	0.040528287	0.062172285	0.04691504	0.040410014	0.048294895	0.054760497	0.054445102	0.031894343	0.052434457	0.098009068	0.070411985	0.025744136	0.040331165	0.042933176	0.049911295	0.065720481	0.023575793	0.036428149	0.062211709
JTT	111	35596.4533	34693.77296	-17235.39418	n/a	n/a	0.0769	0.0511	0.0425	0.0513	0.0203	0.0411	0.0618	0.0747	0.023	0.0526	0.0911	0.0595	0.0234	0.0405	0.0505	0.0682	0.0585	0.0143	0.0323	0.0664
mtREV24+I+F	131	35613.48152	34548.36466	-17142.49704	0.152303622	n/a	0.052868125	0.040528287	0.062172285	0.04691504	0.040410014	0.048294895	0.054760497	0.054445102	0.031894343	0.052434457	0.098009068	0.070411985	0.025744136	0.040331165	0.042933176	0.049911295	0.065720481	0.023575793	0.036428149	0.062211709
JTT+F	130	35624.79799	34567.80143	-17153.22583	n/a	n/a	0.052868125	0.040528287	0.062172285	0.04691504	0.040410014	0.048294895	0.054760497	0.054445102	0.031894343	0.052434457	0.098009068	0.070411985	0.025744136	0.040331165	0.042933176	0.049911295	0.065720481	0.023575793	0.036428149	0.062211709
Dayhoff+F	130	35833.7116	34776.71505	-17257.68264	n/a	n/a	0.052868125	0.040528287	0.062172285	0.04691504	0.040410014	0.048294895	0.054760497	0.054445102	0.031894343	0.052434457	0.098009068	0.070411985	0.025744136	0.040331165	0.042933176	0.049911295	0.065720481	0.023575793	0.036428149	0.062211709
cpREV	111	35865.26417	34962.58383	-17369.79962	n/a	n/a	0.0756	0.0621	0.041	0.0371	0.0091	0.0382	0.0495	0.0838	0.0246	0.0806	0.1011	0.0504	0.022	0.0506	0.0431	0.0622	0.0543	0.0181	0.0307	0.066
Dayhoff	111	35926.30154	35023.6212	-17400.3183	n/a	n/a	0.0871	0.0409	0.0404	0.0469	0.0335	0.0383	0.0495	0.0886	0.0336	0.0369	0.0854	0.0805	0.0148	0.0398	0.0507	0.0696	0.0585	0.0105	0.0299	0.0647
cpREV+F	130	35966.89975	34909.9032	-17324.27672	n/a	n/a	0.052868125	0.040528287	0.062172285	0.04691504	0.040410014	0.048294895	0.054760497	0.054445102	0.031894343	0.052434457	0.098009068	0.070411985	0.025744136	0.040331165	0.042933176	0.049911295	0.065720481	0.023575793	0.036428149	0.062211709
mtREV24+I	112	36536.73238	35625.92869	-17700.46316	0.149877017	n/a	0.072	0.019	0.039	0.019	0.006	0.025	0.024	0.056	0.028	0.087	0.168	0.023	0.053	0.06	0.055	0.072	0.088	0.029	0.033	0.044
mtREV24+F	130	36553.64749	35496.65094	-17617.65059	n/a	n/a	0.052868125	0.040528287	0.062172285	0.04691504	0.040410014	0.048294895	0.054760497	0.054445102	0.031894343	0.052434457	0.098009068	0.070411985	0.025744136	0.040331165	0.042933176	0.049911295	0.065720481	0.023575793	0.036428149	0.062211709
mtREV24	111	37428.93895	36526.25861	-18151.63701	n/a	n/a	0.072	0.019	0.039	0.019	0.006	0.025	0.024	0.056	0.028	0.087	0.168	0.023	0.053	0.06	0.055	0.072	0.088	0.029	0.033	0.044

NOTE.-- Models with the lowest BIC scores (Bayesian Information Criterion) are considered to describe the substitution pattern the best. For each model, AICc value (Akaike Information Criterion, corrected), Maximum Likelihood value (lnL), and the number of parameters (including branch lengths) are also presented. Non-uniformity of evolutionary rates among sites may be modeled by using a discrete Gamma distribution (+G) with 5 rate categories and by assuming that a certain fraction of sites are evolutionarily invariable (+I). Whenever applicable, estimates of gamma shape parameter and/or the estimated fraction of invariant sites are shown. They are followed by amino acid frequencies (f) and rates of amino acid substitutions (r) for each amino acid pair Relative values of instantaneous r should be considered when evaluating them. For simplicity, sum of r values is made equal to 1 for each model. For estimating ML values, a tree topology was automatically computed. The analysis involved 57 amino acid sequences. All positions containing gaps and missing data were eliminated. There were a total of 445 positions in the final dataset. Evolutionary analyses were conducted in MEGA6.

Abbreviations: GTR: General Time Reversible; JTT: Jones-Taylor-Thornton; rtREV: General Reverse Transcriptase; cpREV: General Reversible Chloroplast; mtREV24: General Reversible Mitochondrial.

From\To	A	R	N	D	C	Q	E	G	H	I	L	K	M	F	P	S	T	W	Y	V
A	-	0.001207701	0.000590135	0.001064639	0.001635379	0.002008068	0.003775711	0.006016155	0.00040742	0.000472971	0.001989313	0.001760201	0.001310167	0.00054504	0.00263396	0.014691894	0.005790007	0.000110741	0.000379807	0.008947381
R	0.001706943	-	0.001602891	0.00033397	0.000351211	0.00581349	0.001323241	0.00113621	0.00275498	0.000400874	0.001518882	0.02075447	0.000564302	0.000113266	0.000743751	0.002667099	0.001566879	0.000363754	0.000545428	0.000600106
N	0.001111551	0.002136107	-	0.013676704	0.000347411	0.003510884	0.001969436	0.004186316	0.005119448	0.00060452	0.000344321	0.007037541	0.00043244	0.000192332	0.000361856	0.012457818	0.005414321	2.78058E-05	0.001061619	0.000293888
D	0.001586684	0.000352157	0.01082159	-	4.11006E-05	0.001083618	0.019064497	0.002460362	0.001052575	3.37453E-05	7.58616E-05	0.000928328	2.97786E-05	3.74158E-05	0.000882249	0.003854726	0.00115248	1.83162E-05	0.000234357	0.000133329
C	0.009994813	0.001518675	0.001127254	0.000168545	-	0.000175586	1.27209E-05	0.001657657	0.000727224	0.001012128	0.002989009	4.35229E-05	0.001041667	0.002374473	0.000168601	0.008654047	0.003094533	0.000410645	0.002021733	0.006880462
Q	0.003894569	0.007977346	0.00361509	0.001410163	5.57206E-05	-	0.015009813	0.000780277	0.00546489	0.000229979	0.00293089	0.010611025	0.001949535	7.70293E-05	0.00139631	0.003803609	0.002923109	0.000144739	0.000446375	0.000738625
E	0.004170234	0.001034049	0.001154849	0.014128596	2.29891E-06	0.008547831	-	0.001015817	0.000481242	0.000139732	0.000350591	0.00592896	0.000202504	4.04127E-05	0.00093806	0.001901988	0.001636045	4.77066E-05	0.000208216	0.000860488
G	0.008296097	0.001108547	0.003064844	0.00227649	0.000374019	0.000554782	0.00126826	-	0.000353635	2.74792E-05	0.000222719	0.000973199	0.000162645	0.000192463	0.000440527	0.00540782	0.000351368	0.000164527	9.48463E-05	0.000269352
H	0.001440979	0.006894042	0.009613021	0.00249793	0.00042085	0.009965877	0.001541052	0.000907018	-	0.000343709	0.001843286	0.002287574	0.000515742	0.001465477	0.001138107	0.003076918	0.001581154	0.000365866	0.00920524	0.000417939
I	0.000601636	0.000360785	0.000408256	2.88021E-05	0.000210659	0.000150837	0.000160929	2.53483E-05	0.000123616	-	0.020857741	0.000521871	0.004981287	0.002390534	0.000175085	0.000199236	0.002797548	6.84237E-05	0.000403335	0.037396578
L	0.001587459	0.000857559	0.000145876	4.06194E-05	0.000390275	0.001205919	0.000253302	0.000128885	0.000415889	0.013084795	-	0.000451108	0.007357641	0.005570025	0.000557053	0.000566541	0.000819818	0.000379702	0.00051977	0.005979547
K	0.002154366	0.017972535	0.004572985	0.000762378	8.71603E-06	0.00669628	0.006570132	0.000863782	0.000791621	0.000502136	0.000691892	-	0.000765333	5.13844E-05	0.000873003	0.002326877	0.003076626	3.05817E-05	0.000228849	0.000650376
M	0.004513515	0.001375436	0.000790925	6.88342E-05	0.000587166	0.003462886	0.000631627	0.000406324	0.000502349	0.01349056	0.031763426	0.002154177	-	0.003864577	0.000223324	0.001078338	0.005467599	0.000426606	0.000834874	0.006667748
F	0.001018726	0.000149785	0.000190854	4.69241E-05	0.000726173	7.42342E-05	6.83889E-05	0.000260868	0.000774449	0.003512562	0.013046278	7.84698E-05	0.002096731	-	0.00021128	0.001124519	0.000446533	0.001505689	0.013536657	0.002299057
P	0.004728808	0.000944736	0.000344906	0.001062786	4.95275E-05	0.001292538	0.001524794	0.000573536	0.000577711	0.000247111	0.001253256	0.001280563	0.000116383	0.000202942	-	0.004158861	0.001546531	5.82949E-05	0.000155443	0.001041226
S	0.018981801	0.002438031	0.00854522	0.003341682	0.001829459	0.002533812	0.002224875	0.005066722	0.001123985	0.000202361	0.000917258	0.002456268	0.000404414	0.000777316	0.002992896	-	0.017515552	0.000152499	0.000694789	0.000345443
T	0.008591077	0.001644918	0.004265148	0.001147398	0.00075129	0.002236313	0.00219787	0.000378073	0.000663327	0.003263219	0.001524357	0.003729804	0.002354925	0.000354481	0.001278158	0.020115586	-	8.62956E-05	0.000426436	0.007684177
W	0.000725662	0.001686454	9.67348E-05	8.05328E-05	0.000440288	0.000489026	0.000283037	0.000781826	0.00067785	0.000352479	0.003117953	0.000163731	0.000811457	0.00527877	0.000212772	0.000773453	0.000381107	-	0.005467142	0.000665504
Y	0.000879221	0.000893333	0.001304746	0.00036402	0.000765778	0.000532788	0.000436404	0.000159221	0.006024979	0.000734009	0.001507812	0.000432841	0.000561007	0.016765559	0.00020043	0.001244884	0.000665305	0.001931387	-	0.000875515
V	0.010230865	0.000485495	0.00017841	0.000102295	0.001287294	0.000435471	0.00089084	0.000223348	0.000135118	0.033616196	0.008568116	0.000607608	0.002213133	0.001406492	0.000663161	0.000305727	0.005921685	0.000116129	0.000432459	-

From\To	A	R	N	D	C	Q	E	G	H	I
A	-	0.000876381	0.000875472	0.000943015	0.005116576	0.002382739	0.002892968	0.005722008	0.00058222	0.000399638
R	0.001143217	-	0.002377908	0.000295817	0.001098826	0.006898187	0.001013874	0.001080657	0.003936977	0.00033872
N	0.000744456	0.001550089	-	0.012114273	0.001086939	0.004165954	0.001508991	0.003981634	0.007315899	0.000510791
D	0.001062674	0.000255546	0.016053956	-	0.00012859	0.001285802	0.014607308	0.002340067	0.001504173	2.85132E-05
C	0.006693979	0.001102042	0.001672295	0.00014929	-	0.000208348	9.7468E-06	0.00157661	0.001039233	0.0008552
Q	0.002608369	0.005788846	0.005363028	0.001249065	0.000174332	-	0.01150059	0.000742127	0.007809549	0.000194322
E	0.002792995	0.000750369	0.001713232	0.01251454	7.19257E-06	0.010142709	-	0.00096615	0.000687715	0.000118067
G	0.005556272	0.000804429	0.004546732	0.002016423	0.001170185	0.000658295	0.000971747	-	0.000505359	2.32186E-05
H	0.000965089	0.005002735	0.014261029	0.002212565	0.001316704	0.011825337	0.001180762	0.000862671	-	0.000290418
I	0.000402943	0.000261808	0.000605652	2.55118E-05	0.000659083	0.00017898	0.000123304	2.4109E-05	0.000176653	-
L	0.001063193	0.000622297	0.000216409	3.5979E-05	0.001221044	0.001430922	0.000194081	0.000122583	0.000594322	0.01105603
K	0.001442876	0.013041962	0.006784078	0.000675284	2.72697E-05	0.00794569	0.005034067	0.000821549	0.001131258	0.000424281
M	0.003022906	0.000998099	0.001173347	6.09705E-05	0.001837054	0.004109	0.000483956	0.000386458	0.000717877	0.011398882
F	0.000682287	0.000108693	0.000283134	4.15634E-05	0.002271961	8.8085E-05	5.23999E-05	0.000248113	0.001106719	0.002967948
P	0.003167097	0.000685558	0.000511672	0.000941373	0.000154956	0.001533703	0.001168304	0.000545494	0.000825572	0.000208797
S	0.012712973	0.001769183	0.012676934	0.002959927	0.00572379	0.003006578	0.00170471	0.004818995	0.00160622	0.000170986
T	0.005753834	0.001193653	0.006327398	0.001016319	0.002350544	0.00265357	0.001684019	0.000359588	0.000947921	0.002757265
W	0.000486008	0.001223794	0.000143507	7.13327E-05	0.001377519	0.00058027	0.000216864	0.0007436	0.000968675	0.000297828
Y	0.000588854	0.000648257	0.001935606	0.000322434	0.002395875	0.000632197	0.000334375	0.000151436	0.008609939	0.000620203
V	0.006852074	0.000352304	0.000264674	9.06086E-05	0.00402753	0.000516723	0.000682566	0.000212427	0.00019309	0.028404089

From\To	L	K	M	F	P	S	T	W	Y	V
A	0.001970991	0.001921683	0.001472004	0.000520492	0.002571938	0.012001957	0.007152603	0.000216729	0.000405743	0.008063067
R	0.001504892	0.022658506	0.000634007	0.000108164	0.000726237	0.00217878	0.001935622	0.000711895	0.000582674	0.000540794
N	0.000341149	0.007683172	0.000485856	0.000183669	0.000353336	0.010176917	0.006688505	5.44181E-05	0.001134115	0.000264842
D	7.51629E-05	0.001013494	3.34569E-05	3.57306E-05	0.000861475	0.003148964	0.0014237	3.58462E-05	0.000250361	0.000120152
C	0.002961479	4.75157E-05	0.001170338	0.002267528	0.000164631	0.007069579	0.003822788	0.000803665	0.002159793	0.006200432
Q	0.002903896	0.011584492	0.002190349	7.35599E-05	0.001363431	0.003107207	0.003611021	0.000283267	0.000476857	0.000665623
E	0.000347361	0.006472889	0.000227518	3.85926E-05	0.000915971	0.001553753	0.002021065	9.33656E-05	0.000222435	0.000775442
G	0.000220667	0.001062481	0.000182735	0.000183794	0.000430154	0.004417703	0.000434057	0.000321993	0.000101323	0.00024273
H	0.001826309	0.002497438	0.000579449	0.001399473	0.001111308	0.002513565	0.001953256	0.000716029	0.009833848	0.000376632
I	0.020665633	0.000569748	0.005596594	0.002282865	0.000170962	0.000162758	0.003455911	0.000133911	0.000430878	0.03370049
L	-	0.000492493	0.008266484	0.005319154	0.000543936	0.000462813	0.001012751	0.000743106	0.000555264	0.005388559
K	0.00068552	-	0.00085987	4.90701E-05	0.000852446	0.001900849	0.003800666	5.98508E-05	0.000244477	0.000586096
M	0.031470872	0.002351803	-	0.003690518	0.000218066	0.000880905	0.006754321	0.000834902	0.000891886	0.006008741
F	0.012926117	8.56687E-05	0.002355727	-	0.000206305	0.000918631	0.000551618	0.002946753	0.014461049	0.00207183
P	0.001241713	0.001398043	0.000130759	0.000193802	-	0.003397416	0.001910485	0.000114088	0.000166058	0.000938316
S	0.000908809	0.002681609	0.000454369	0.000742306	0.002922422	-	0.021637588	0.000298453	0.000742235	0.000311302
T	0.001510317	0.00407198	0.002645814	0.000338515	0.001248061	0.016432626	-	0.000168887	0.000455557	0.006924712
W	0.003089235	0.000178752	0.000911691	0.005041017	0.000207762	0.000631842	0.000470795	-	0.005840482	0.000599729
Y	0.001493924	0.00047255	0.000630305	0.016010447	0.000195711	0.001016958	0.000821875	0.003779879	-	0.000788984
V	0.0084892	0.000663351	0.002486507	0.001343144	0.000647545	0.000249751	0.007315269	0.000227274	0.00046199	-

From\To	A	R	N	D	C	Q	E	G	H	I
A	-	0.000817463	0.001017768	0.000223977	0.002656196	0.001029232	0.002609543	0.004578523	0.000434685	3.6038E-05
R	0.001165741	-	0.000698468	0.000671931	0.000556652	0.007108135	0.000322166	0.001390514	0.001304055	0.000864912
N	0.001748612	0.000841506	-	0.008600712	0.000774472	0.007590587	0.00254511	0.003188008	0.00463664	0.00126133
D	0.000342865	0.00072129	0.007663194	-	6.05056E-06	0.002508753	0.017461387	0.002068814	0.001318544	3.6038E-05
C	0.015051777	0.002211957	0.002554398	2.23977E-05	-	0.002251445	3.22166E-05	0.001627919	0.001796698	0.003747951
Q	0.001097168	0.005313507	0.004709671	0.00174702	0.000423539	-	0.011984568	0.00061047	0.005607436	0.001189254
E	0.002777207	0.00024043	0.001576543	0.012139547	6.05056E-06	0.011964824	-	0.002374049	0.000492643	3.6038E-05
G	0.004628679	0.000985764	0.001875886	0.001366259	0.000290427	0.000578943	0.002255161	-	0.000985286	3.6038E-05
H	0.001028595	0.002163871	0.006385995	0.00203819	0.000750269	0.012447277	0.001095364	0.002306219	-	0.001225292
I	3.42865E-05	0.000577032	0.000698468	2.23977E-05	0.000629258	0.001061396	3.22166E-05	3.3915E-05	0.000492643	-
L	0.001542893	0.000432774	0.000299344	0.000111988	0.000665562	0.001736829	0.000676548	0.000101745	0.000738964	0.013874625
K	0.001302887	0.014257509	0.002454617	0.000447954	9.6809E-05	0.009938523	0.004542538	0.001017449	0.001101202	0.001225292
M	0.00805733	0.001370452	1.99562E-05	2.23977E-05	0.000943887	0.005081834	3.22166E-05	0.001254854	0.001680782	0.013514245
F	3.42865E-05	0.000168301	0.000977855	2.23977E-05	0.000423539	3.21635E-05	3.22166E-05	0.000237405	0.002043019	0.002306431
P	0.003325791	0.000577032	0.000658556	0.001231873	6.05056E-06	0.002187118	0.001675262	0.000576555	0.000637538	0.00036038
S	0.015771794	0.002452388	0.005867133	0.003650823	0.000453792	0.007236789	0.003060575	0.005155077	0.002651578	0.000144152
T	0.008845919	0.001538753	0.002953523	0.001231873	0.000707916	0.004695872	0.00264176	0.000237405	0.000709985	0.002594735
W	0.000171433	0.000312559	0.0003193	2.23977E-05	0.000332781	0.000321635	0.000547682	0.000780045	0.000695496	0.001405481
Y	0.001885758	0.001130022	0.000558775	2.23977E-05	0.000792623	0.001447358	3.22166E-05	0.000712215	0.004448276	0.000936988
V	0.006754442	0.000697247	0.000419081	0.000134386	0.001784915	0.001157886	0.00112758	0.000101745	1.44895E-05	0.03776781

From\To	L	K	M	F	P	S	T	W	Y	V
A	0.00243137	0.001514657	0.001870897	1.52326E-05	0.003505981	0.011914296	0.008517278	6.66092E-05	0.000928283	0.006472136
R	0.000972548	0.02363662	0.000453792	0.000106628	0.000867459	0.002641866	0.002112813	0.000173184	0.00079326	0.000952751
N	0.000810457	0.004902705	7.96126E-06	0.000746395	0.001192757	0.007614789	0.004885881	0.00021315	0.000472581	0.000689923
D	0.000270152	0.000797188	7.96126E-06	1.52326E-05	0.001987928	0.004221805	0.001815699	1.33218E-05	1.68779E-05	0.000197121
C	0.005943349	0.00063775	0.001241957	0.001066279	3.61441E-05	0.001942548	0.003862487	0.000732702	0.002211002	0.009691777
Q	0.002917644	0.012316553	0.00125788	1.52326E-05	0.002457801	0.005827645	0.004819855	0.000133218	0.000759505	0.001182725
E	0.001134639	0.005620174	7.96126E-06	1.52326E-05	0.001879495	0.002460561	0.002707042	0.000226471	1.68779E-05	0.001149872
G	0.000162091	0.001195782	0.000294567	0.000106628	0.00061445	0.003936898	0.000231089	0.000306403	0.000354435	9.85604E-05
H	0.002755553	0.003029314	0.000923507	0.00214779	0.001590342	0.004739818	0.001617623	0.000639449	0.005181509	3.28535E-05
I	0.02080172	0.001355219	0.002985474	0.000974883	0.000361441	0.000103603	0.002376915	0.000519552	0.000438825	0.034430449
L	-	0.000916766	0.004625494	0.002726627	0.000795171	0.000621615	0.000825318	0.000626127	0.001080184	0.00367959
K	0.0012427	-	0.001066809	0.000213256	0.001554198	0.00199435	0.003631398	7.99311E-05	1.68779E-05	0.000624216
M	0.031391687	0.005341159	-	0.00376244	3.61441E-05	2.59006E-05	0.004324665	0.001478725	0.001248963	0.007753422
F	0.009671449	0.000558032	0.001966432	-	0.000397586	0.000518013	0.002277877	0.002424576	0.017164803	0.00302252
P	0.00118867	0.001713954	7.96126E-06	0.000167558	-	0.003470686	0.002046788	0.000119897	0.00023629	0.000821337
S	0.001296731	0.003069173	7.96126E-06	0.000304651	0.004843314	-	0.022151526	0.000186506	0.000523214	0.001281286
T	0.001350761	0.004384533	0.001042926	0.001051046	0.002240936	0.017379332	-	1.33218E-05	0.000573848	0.006439282
W	0.002539431	0.000239156	0.0008837	0.002772324	0.000325297	0.000362609	3.30127E-05	-	0.002970507	0.000854191
Y	0.003457948	3.98594E-05	0.000589134	0.015491505	0.000506018	0.00080292	0.001122432	0.002344645	-	0.001938355
V	0.00605141	0.000757328	0.001878858	0.001401395	0.000903603	0.001010125	0.006470491	0.000346368	0.000995795	-

From\To	A	R	N	D	C	Q	E	G	H	I	L	K	M	F	P	S	T	W	Y	V
A	-	0.000711895	0.001638121	0.000242377	0.009164999	0.000798418	0.00229156	0.00379727	0.000494326	2.70892E-05	0.002278545	0.001382322	0.00312554	2.08363E-05	0.002151511	0.01186139	0.008759904	6.08997E-05	0.001035091	0.006331655
R	0.000928649	-	0.001124201	0.00072713	0.001920683	0.005514072	0.000282909	0.001153245	0.001482979	0.00065014	0.000911418	0.021571492	0.00075811	0.000145854	0.000532333	0.002630134	0.002172999	0.000158339	0.000884533	0.000932071
N	0.001392974	0.000732833	-	0.009307268	0.002672255	0.00588833	0.002234979	0.002644025	0.005272815	0.000948121	0.000759515	0.004474357	1.33002E-05	0.001020977	0.000731957	0.007580975	0.005025061	0.000194879	0.000526956	0.000674948
D	0.000273132	0.000628143	0.012334086	-	2.0877E-05	0.001946143	0.015333651	0.001715804	0.001499457	2.70892E-05	0.000253172	0.000727538	1.33002E-05	2.08363E-05	0.001219929	0.004203058	0.001867421	1.21799E-05	1.88198E-05	0.000192842
C	0.011990501	0.001926305	0.004111362	2.42377E-05	-	0.001746539	2.82909E-05	0.00135014	0.002043216	0.002817274	0.005569778	0.00058203	0.002074827	0.001458538	2.21805E-05	0.001933922	0.003972515	0.000669897	0.0024654	0.009481413
Q	0.000874023	0.004627319	0.007580324	0.001890539	0.001461389	-	0.010524203	0.000506303	0.006376811	0.000893943	0.002734254	0.011240457	0.002101427	2.08363E-05	0.001508276	0.005801767	0.004957155	0.000121799	0.000846893	0.001157054
E	0.00221237	0.000209381	0.002537481	0.013136821	2.0877E-05	0.009281605	-	0.001968955	0.000560237	2.70892E-05	0.001063321	0.005129141	1.33002E-05	2.08363E-05	0.001153388	0.002449635	0.002784156	0.000207059	1.88198E-05	0.001124913
G	0.003687284	0.000858462	0.003019281	0.001478498	0.001002096	0.00044911	0.001980361	-	0.001120473	2.70892E-05	0.000151903	0.001091307	0.000492106	0.000145854	0.000377069	0.003919416	0.000237672	0.000280139	0.000395217	9.64211E-05
H	0.000819396	0.001884429	0.010278405	0.002205629	0.002588747	0.009655863	0.00096189	0.001912699	-	0.000921032	0.002582351	0.002764643	0.00154282	0.002937912	0.000975943	0.00471877	0.001663703	0.000584637	0.005777692	3.21404E-05
I	2.73132E-05	0.000502514	0.001124201	2.42377E-05	0.002171207	0.000823368	2.82909E-05	2.81279E-05	0.000560237	-	0.019494222	0.001236814	0.004987564	0.00133352	0.000221805	0.000103143	0.002444624	0.000475018	0.000489316	0.033683121
L	0.001229095	0.000376886	0.0004818	0.000121188	0.002296469	0.00134733	0.000594108	8.43838E-05	0.000840355	0.01042933	-	0.000836668	0.007727399	0.00372969	0.000487972	0.000618855	0.000848828	0.000572457	0.00120447	0.003599723
K	0.001037902	0.01241629	0.003950762	0.000484754	0.000334032	0.00770972	0.003989013	0.000843838	0.001252294	0.000921032	0.00116459	-	0.001782223	0.000291708	0.000953763	0.001985494	0.003734843	7.30797E-05	1.88198E-05	0.000610667
M	0.006418606	0.001193471	3.212E-05	2.42377E-05	0.003256811	0.003942187	2.82909E-05	0.001040733	0.001911395	0.010158438	0.029418553	0.004874502	-	0.005146555	2.21805E-05	2.57856E-05	0.004447858	0.001351974	0.001392668	0.00758513
F	2.73132E-05	0.000146567	0.001573881	2.42377E-05	0.001461389	2.49506E-05	2.82909E-05	0.000196895	0.002323334	0.001733707	0.009063547	0.000509276	0.003285142	-	0.000243986	0.000515713	0.002342765	0.002216749	0.019139781	0.002956915
P	0.002649382	0.000502514	0.00105996	0.001333072	2.0877E-05	0.001696637	0.001471125	0.000478175	0.000725012	0.000270892	0.001113956	0.001564206	1.33002E-05	0.000229199	-	0.003455274	0.002105093	0.000109619	0.000263478	0.00080351
S	0.012564079	0.002135686	0.009443284	0.003950741	0.001565774	0.005613874	0.002687633	0.004275445	0.003015391	0.000108357	0.001215224	0.00280102	1.33002E-05	0.000416725	0.002972191	-	0.022782541	0.000170519	0.000583415	0.001253475
T	0.007046809	0.001340038	0.004753762	0.001333072	0.002442608	0.00364278	0.002319851	0.000196895	0.0008074	0.00195042	0.001265859	0.004001457	0.001742322	0.001437702	0.001375193	0.017302158	-	1.21799E-05	0.000639875	0.006299515
W	0.000136566	0.000272195	0.00051392	2.42377E-05	0.001148235	0.000249506	0.000480945	0.000646942	0.000790922	0.001056478	0.002379814	0.000218261	0.001476319	0.003792199	0.000199625	0.000360999	3.39531E-05	-	0.003312293	0.00083565
Y	0.001502227	0.00098409	0.00089936	2.42377E-05	0.002734886	0.001122775	2.82909E-05	0.000590686	0.005058607	0.000704318	0.003240598	3.63769E-05	0.000984213	0.021190472	0.000310527	0.000799355	0.001154406	0.00214367	-	0.001896283
V	0.005380703	0.000607205	0.00067452	0.000145426	0.006158712	0.00089822	0.00099018	8.43838E-05	1.64775E-05	0.028389449	0.005671046	0.000691161	0.00313884	0.001916936	0.000554513	0.00100564	0.006654811	0.000316678	0.001110371	-

From\Tc	A	R	N	D	C	Q	E	G	H	I	L	K	M	F	P	S	T	W	Y	V
A	-	0.000711895	0.001638121	0.000242377	0.009164999	0.000798418	0.00229156	0.00379727	0.000494326	2.70892E-05	0.002278545	0.001382322	0.00312554	2.08363E-05	0.002151511	0.01186139	0.008759904	6.08997E-05	0.001035091	0.006331655
R	0.000928649	-	0.001124201	0.00072713	0.001920683	0.005514072	0.000282909	0.001153245	0.001482979	0.00065014	0.000911418	0.021571492	0.00075811	0.000145854	0.000532333	0.002630134	0.002172999	0.000158339	0.000884533	0.000932071
N	0.001392974	0.000732833	-	0.009307268	0.002672255	0.00588833	0.002234979	0.002644025	0.005272815	0.000948121	0.000759515	0.004474357	1.33002E-05	0.001020977	0.000731957	0.007580975	0.005025061	0.000194879	0.000526956	0.000674948
D	0.000273132	0.000628143	0.012334086	-	2.0877E-05	0.001946143	0.015333651	0.001715804	0.001499457	2.70892E-05	0.000253172	0.000727538	1.33002E-05	2.08363E-05	0.001219929	0.004203058	0.001867421	1.21799E-05	1.88198E-05	0.000192842
C	0.011990501	0.001926305	0.004111362	2.42377E-05	-	0.001746539	2.82909E-05	0.00135014	0.002043216	0.002817274	0.005569778	0.00058203	0.002074827	0.001458538	2.21805E-05	0.001933922	0.003972515	0.000669897	0.0024654	0.009481413
Q	0.000874023	0.004627319	0.007580324	0.001890539	0.001461389	-	0.010524203	0.000506303	0.006376811	0.000893943	0.002734254	0.011240457	0.002101427	2.08363E-05	0.001508276	0.005801767	0.004957155	0.000121799	0.000846893	0.001157054
E	0.00221237	0.000209381	0.002537481	0.013136821	2.0877E-05	0.009281605	-	0.001968955	0.000560237	2.70892E-05	0.001063321	0.005129141	1.33002E-05	2.08363E-05	0.001153388	0.002449635	0.002784156	0.000207059	1.88198E-05	0.001124913
G	0.003687284	0.000858462	0.003019281	0.001478498	0.001002096	0.00044911	0.001980361	-	0.001120473	2.70892E-05	0.000151903	0.001091307	0.000492106	0.000145854	0.000377069	0.003919416	0.000237672	0.000280139	0.000395217	9.64211E-05
H	0.000819396	0.001884429	0.010278405	0.002205629	0.002588747	0.009655863	0.00096189	0.001912699	-	0.000921032	0.002582351	0.002764643	0.00154282	0.002937912	0.000975943	0.00471877	0.001663703	0.000584637	0.005777692	3.21404E-05
I	2.73132E-05	0.000502514	0.001124201	2.42377E-05	0.002171207	0.000823368	2.82909E-05	2.81279E-05	0.000560237	-	0.019494222	0.001236814	0.004987564	0.00133352	0.000221805	0.000103143	0.002444624	0.000475018	0.000489316	0.033683121
L	0.001229095	0.000376886	0.0004818	0.000121188	0.002296469	0.00134733	0.000594108	8.43838E-05	0.000840355	0.01042933	-	0.000836668	0.007727399	0.00372969	0.000487972	0.000618855	0.000848828	0.000572457	0.00120447	0.003599723
K	0.001037902	0.01241629	0.003950762	0.000484754	0.000334032	0.00770972	0.003989013	0.000843838	0.001252294	0.000921032	0.00116459	-	0.001782223	0.000291708	0.000953763	0.001985494	0.003734843	7.30797E-05	1.88198E-05	0.000610667
M	0.006418606	0.001193471	3.212E-05	2.42377E-05	0.003256811	0.003942187	2.82909E-05	0.001040733	0.001911395	0.010158438	0.029418553	0.004874502	-	0.005146555	2.21805E-05	2.57856E-05	0.004447858	0.001351974	0.001392668	0.00758513
F	2.73132E-05	0.000146567	0.001573881	2.42377E-05	0.001461389	2.49506E-05	2.82909E-05	0.000196895	0.002323334	0.001733707	0.009063547	0.000509276	0.003285142	-	0.000243986	0.000515713	0.002342765	0.002216749	0.019139781	0.002956915
P	0.002649382	0.000502514	0.00105996	0.001333072	2.0877E-05	0.001696637	0.001471125	0.000478175	0.000725012	0.000270892	0.001113956	0.001564206	1.33002E-05	0.000229199	-	0.003455274	0.002105093	0.000109619	0.000263478	0.00080351
S	0.012564079	0.002135686	0.009443284	0.003950741	0.001565774	0.005613874	0.002687633	0.004275445	0.003015391	0.000108357	0.001215224	0.00280102	1.33002E-05	0.000416725	0.002972191	-	0.022782541	0.000170519	0.000583415	0.001253475
T	0.007046809	0.001340038	0.004753762	0.001333072	0.002442608	0.00364278	0.002319851	0.000196895	0.0008074	0.00195042	0.001265859	0.004001457	0.001742322	0.001437702	0.001375193	0.017302158	-	1.21799E-05	0.000639875	0.006299515
W	0.000136566	0.000272195	0.00051392	2.42377E-05	0.001148235	0.000249506	0.000480945	0.000646942	0.000790922	0.001056478	0.002379814	0.000218261	0.001476319	0.003792199	0.000199625	0.000360999	3.39531E-05	-	0.003312293	0.00083565
Y	0.001502227	0.00098409	0.00089936	2.42377E-05	0.002734886	0.001122775	2.82909E-05	0.000590686	0.005058607	0.000704318	0.003240598	3.63769E-05	0.000984213	0.021190472	0.000310527	0.000799355	0.001154406	0.00214367	-	0.001896283
V	0.005380703	0.000607205	0.00067452	0.000145426	0.006158712	0.00089822	0.00099018	8.43838E-05	1.64775E-05	0.028389449	0.005671046	0.000691161	0.00313884	0.001916936	0.000554513	0.00100564	0.006654811	0.000316678	0.001110371	-

From\To	A	R	N	D	C	Q	E	G	H	I
A	-	0.001207701	0.000590135	0.001064639	0.001635379	0.002008068	0.003775711	0.006016155	0.00040742	0.000472971
R	0.001706943	-	0.001602891	0.00033397	0.000351211	0.00581349	0.001323241	0.00113621	0.00275498	0.000400874
N	0.001111551	0.002136107	-	0.013676704	0.000347411	0.003510884	0.001969436	0.004186316	0.005119448	0.00060452
D	0.001586684	0.000352157	0.01082159	-	4.11006E-05	0.001083618	0.019064497	0.002460362	0.001052575	3.37453E-05
C	0.009994813	0.001518675	0.001127254	0.000168545	-	0.000175586	1.27209E-05	0.001657657	0.000727224	0.001012128
Q	0.003894569	0.007977346	0.00361509	0.001410163	5.57206E-05	-	0.015009813	0.000780277	0.00546489	0.000229979
E	0.004170234	0.001034049	0.001154849	0.014128596	2.29891E-06	0.008547831	-	0.001015817	0.000481242	0.000139732
G	0.008296097	0.001108547	0.003064844	0.00227649	0.000374019	0.000554782	0.00126826	-	0.000353635	2.74792E-05
H	0.001440979	0.006894042	0.009613021	0.00249793	0.00042085	0.009965877	0.001541052	0.000907018	-	0.000343709
I	0.000601636	0.000360785	0.000408256	2.88021E-05	0.000210659	0.000150837	0.000160929	2.53483E-05	0.000123616	-
L	0.001587459	0.000857559	0.000145876	4.06194E-05	0.000390275	0.001205919	0.000253302	0.000128885	0.000415889	0.013084795
K	0.002154366	0.017972535	0.004572985	0.000762378	8.71603E-06	0.00669628	0.006570132	0.000863782	0.000791621	0.000502136
M	0.004513515	0.001375436	0.000790925	6.88342E-05	0.000587166	0.003462886	0.000631627	0.000406324	0.000502349	0.01349056
F	0.001018726	0.000149785	0.000190854	4.69241E-05	0.000726173	7.42342E-05	6.83889E-05	0.000260868	0.000774449	0.003512562
P	0.004728808	0.000944736	0.000344906	0.001062786	4.95275E-05	0.001292538	0.001524794	0.000573536	0.000577711	0.000247111
S	0.018981801	0.002438031	0.00854522	0.003341682	0.001829459	0.002533812	0.002224875	0.005066722	0.001123985	0.000202361
T	0.008591077	0.001644918	0.004265148	0.001147398	0.00075129	0.002236313	0.00219787	0.000378073	0.000663327	0.003263219
W	0.000725662	0.001686454	9.67348E-05	8.05328E-05	0.000440288	0.000489026	0.000283037	0.000781826	0.00067785	0.000352479
Y	0.000879221	0.000893333	0.001304746	0.00036402	0.000765778	0.000532788	0.000436404	0.000159221	0.006024979	0.000734009
V	0.010230865	0.000485495	0.00017841	0.000102295	0.001287294	0.000435471	0.00089084	0.000223348	0.000135118	0.033616196

From\To	L	K	M	F	P	S	T	W	Y	V
A	0.001989313	0.001760201	0.001310167	0.00054504	0.00263396	0.014691894	0.005790007	0.000110741	0.000379807	0.008947381
R	0.001518882	0.02075447	0.000564302	0.000113266	0.000743751	0.002667099	0.001566879	0.000363754	0.000545428	0.000600106
N	0.000344321	0.007037541	0.00043244	0.000192332	0.000361856	0.012457818	0.005414321	2.78058E-05	0.001061619	0.000293888
D	7.58616E-05	0.000928328	2.97786E-05	3.74158E-05	0.000882249	0.003854726	0.00115248	1.83162E-05	0.000234357	0.000133329
C	0.002989009	4.35229E-05	0.001041667	0.002374473	0.000168601	0.008654047	0.003094533	0.000410645	0.002021733	0.006880462
Q	0.00293089	0.010611025	0.001949535	7.70293E-05	0.00139631	0.003803609	0.002923109	0.000144739	0.000446375	0.000738625
E	0.000350591	0.00592896	0.000202504	4.04127E-05	0.00093806	0.001901988	0.001636045	4.77066E-05	0.000208216	0.000860488
G	0.000222719	0.000973199	0.000162645	0.000192463	0.000440527	0.00540782	0.000351368	0.000164527	9.48463E-05	0.000269352
H	0.001843286	0.002287574	0.000515742	0.001465477	0.001138107	0.003076918	0.001581154	0.000365866	0.00920524	0.000417939
I	0.020857741	0.000521871	0.004981287	0.002390534	0.000175085	0.000199236	0.002797548	6.84237E-05	0.000403335	0.037396578
L	-	0.000451108	0.007357641	0.005570025	0.000557053	0.000566541	0.000819818	0.000379702	0.00051977	0.005979547
K	0.000691892	-	0.000765333	5.13844E-05	0.000873003	0.002326877	0.003076626	3.05817E-05	0.000228849	0.000650376
M	0.031763426	0.002154177	-	0.003864577	0.000223324	0.001078338	0.005467599	0.000426606	0.000834874	0.006667748
F	0.013046278	7.84698E-05	0.002096731	-	0.00021128	0.001124519	0.000446533	0.001505689	0.013536657	0.002299057
P	0.001253256	0.001280563	0.000116383	0.000202942	-	0.004158861	0.001546531	5.82949E-05	0.000155443	0.001041226
S	0.000917258	0.002456268	0.000404414	0.000777316	0.002992896	-	0.017515552	0.000152499	0.000694789	0.000345443
T	0.001524357	0.003729804	0.002354925	0.000354481	0.001278158	0.020115586	-	8.62956E-05	0.000426436	0.007684177
W	0.003117953	0.000163731	0.000811457	0.00527877	0.000212772	0.000773453	0.000381107	-	0.005467142	0.000665504
Y	0.001507812	0.000432841	0.000561007	0.016765559	0.00020043	0.001244884	0.000665305	0.001931387	-	0.000875515
V	0.008568116	0.000607608	0.002213133	0.001406492	0.000663161	0.000305727	0.005921685	0.000116129	0.000432459	-

From\To	A	R	N	D	C	Q	E	G	H	I	L	K	M	F	P	S	T	W	Y	V
A	-	0.000876381	0.000875472	0.000943015	0.005116576	0.002382739	0.002892968	0.005722008	0.00058222	0.000399638	0.001970991	0.001921683	0.001472004	0.000520492	0.002571938	0.012001957	0.007152603	0.000216729	0.000405743	0.008063067
R	0.001143217	-	0.002377908	0.000295817	0.001098826	0.006898187	0.001013874	0.001080657	0.003936977	0.00033872	0.001504892	0.022658506	0.000634007	0.000108164	0.000726237	0.00217878	0.001935622	0.000711895	0.000582674	0.000540794
N	0.000744456	0.001550089	-	0.012114273	0.001086939	0.004165954	0.001508991	0.003981634	0.007315899	0.000510791	0.000341149	0.007683172	0.000485856	0.000183669	0.000353336	0.010176917	0.006688505	5.44181E-05	0.001134115	0.000264842
D	0.001062674	0.000255546	0.016053956	-	0.00012859	0.001285802	0.014607308	0.002340067	0.001504173	2.85132E-05	7.51629E-05	0.001013494	3.34569E-05	3.57306E-05	0.000861475	0.003148964	0.0014237	3.58462E-05	0.000250361	0.000120152
C	0.006693979	0.001102042	0.001672295	0.00014929	-	0.000208348	9.7468E-06	0.00157661	0.001039233	0.0008552	0.002961479	4.75157E-05	0.001170338	0.002267528	0.000164631	0.007069579	0.003822788	0.000803665	0.002159793	0.006200432
Q	0.002608369	0.005788846	0.005363028	0.001249065	0.000174332	-	0.01150059	0.000742127	0.007809549	0.000194322	0.002903896	0.011584492	0.002190349	7.35599E-05	0.001363431	0.003107207	0.003611021	0.000283267	0.000476857	0.000665623
E	0.002792995	0.000750369	0.001713232	0.01251454	7.19257E-06	0.010142709	-	0.00096615	0.000687715	0.000118067	0.000347361	0.006472889	0.000227518	3.85926E-05	0.000915971	0.001553753	0.002021065	9.33656E-05	0.000222435	0.000775442
G	0.005556272	0.000804429	0.004546732	0.002016423	0.001170185	0.000658295	0.000971747	-	0.000505359	2.32186E-05	0.000220667	0.001062481	0.000182735	0.000183794	0.000430154	0.004417703	0.000434057	0.000321993	0.000101323	0.00024273
H	0.000965089	0.005002735	0.014261029	0.002212565	0.001316704	0.011825337	0.001180762	0.000862671	-	0.000290418	0.001826309	0.002497438	0.000579449	0.001399473	0.001111308	0.002513565	0.001953256	0.000716029	0.009833848	0.000376632
I	0.000402943	0.000261808	0.000605652	2.55118E-05	0.000659083	0.00017898	0.000123304	2.4109E-05	0.000176653	-	0.020665633	0.000569748	0.005596594	0.002282865	0.000170962	0.000162758	0.003455911	0.000133911	0.000430878	0.03370049
L	0.001063193	0.000622297	0.000216409	3.5979E-05	0.001221044	0.001430922	0.000194081	0.000122583	0.000594322	0.01105603	-	0.000492493	0.008266484	0.005319154	0.000543936	0.000462813	0.001012751	0.000743106	0.000555264	0.005388559
K	0.001442876	0.013041962	0.006784078	0.000675284	2.72697E-05	0.00794569	0.005034067	0.000821549	0.001131258	0.000424281	0.00068552	-	0.00085987	4.90701E-05	0.000852446	0.001900849	0.003800666	5.98508E-05	0.000244477	0.000586096
M	0.003022906	0.000998099	0.001173347	6.09705E-05	0.001837054	0.004109	0.000483956	0.000386458	0.000717877	0.011398882	0.031470872	0.002351803	-	0.003690518	0.000218066	0.000880905	0.006754321	0.000834902	0.000891886	0.006008741
F	0.000682287	0.000108693	0.000283134	4.15634E-05	0.002271961	8.8085E-05	5.23999E-05	0.000248113	0.001106719	0.002967948	0.012926117	8.56687E-05	0.002355727	-	0.000206305	0.000918631	0.000551618	0.002946753	0.014461049	0.00207183
P	0.003167097	0.000685558	0.000511672	0.000941373	0.000154956	0.001533703	0.001168304	0.000545494	0.000825572	0.000208797	0.001241713	0.001398043	0.000130759	0.000193802	-	0.003397416	0.001910485	0.000114088	0.000166058	0.000938316
S	0.012712973	0.001769183	0.012676934	0.002959927	0.00572379	0.003006578	0.00170471	0.004818995	0.00160622	0.000170986	0.000908809	0.002681609	0.000454369	0.000742306	0.002922422	-	0.021637588	0.000298453	0.000742235	0.000311302
T	0.005753834	0.001193653	0.006327398	0.001016319	0.002350544	0.00265357	0.001684019	0.000359588	0.000947921	0.002757265	0.001510317	0.00407198	0.002645814	0.000338515	0.001248061	0.016432626	-	0.000168887	0.000455557	0.006924712
W	0.000486008	0.001223794	0.000143507	7.13327E-05	0.001377519	0.00058027	0.000216864	0.0007436	0.000968675	0.000297828	0.003089235	0.000178752	0.000911691	0.005041017	0.000207762	0.000631842	0.000470795	-	0.005840482	0.000599729
Y	0.000588854	0.000648257	0.001935606	0.000322434	0.002395875	0.000632197	0.000334375	0.000151436	0.008609939	0.000620203	0.001493924	0.00047255	0.000630305	0.016010447	0.000195711	0.001016958	0.000821875	0.003779879	-	0.000788984
V	0.006852074	0.000352304	0.000264674	9.06086E-05	0.00402753	0.000516723	0.000682566	0.000212427	0.00019309	0.028404089	0.0084892	0.000663351	0.002486507	0.001343144	0.000647545	0.000249751	0.007315269	0.000227274	0.00046199	-

From\To	A	R	N	D	C	Q	E	G	H	I
A	-	0.001293534	0.001062918	0.002248338	0.001057597	0.001779796	0.00490127	0.006290392	0.000412993	0.000499744
R	0.002548351	-	0.001324553	0.00044816	0.000543906	0.005946053	0.001359843	0.002595977	0.002784721	0.000483315
N	0.002355584	0.001490001	-	0.016518547	0.000273148	0.003023741	0.002932984	0.00499761	0.005155072	0.001432623
D	0.003414296	0.000345454	0.011319116	-	3.11962E-05	0.001208178	0.019118189	0.003843288	0.001212677	0.000101939
C	0.004745098	0.001238703	0.000552999	9.21696E-05	-	0.000193568	6.61161E-05	0.001361666	0.000324412	0.000439775
Q	0.004197876	0.007118796	0.003218141	0.00187651	0.000101758	-	0.016936127	0.001465467	0.005595254	0.00029446
E	0.007313034	0.001029902	0.001974694	0.018784355	2.19873E-05	0.010713806	-	0.002520726	0.000742746	0.000329298
G	0.006545486	0.001371145	0.002346539	0.002633465	0.000315798	0.000646518	0.001757927	-	0.000324983	7.87093E-05
H	0.001464381	0.005012003	0.008247972	0.002831502	0.00025638	0.008411466	0.001765073	0.001107408	-	0.000357202
I	0.00089324	0.0004385	0.001155457	0.000119984	0.000175197	0.000223145	0.000394477	0.000135202	0.000180062	-
L	0.001838435	0.001167128	0.000274206	0.000258011	0.000395721	0.001703188	0.000477673	0.000272195	0.000650802	0.008196518
K	0.004187097	0.012550046	0.006279361	0.001459918	7.62366E-05	0.007629478	0.008002647	0.001658638	0.001160239	0.000837061
M	0.004128102	0.001602138	0.000413246	0.000315663	0.0004021	0.003026914	0.000975776	0.000773023	0.000526598	0.011004944
F	0.000972518	0.000240876	0.000200476	0.000142173	0.000409862	0.000195729	0.00025123	0.000221699	0.000885225	0.002738583
P	0.006646344	0.001593524	0.0004067	0.001289935	0.000112659	0.001828325	0.002112901	0.001081478	0.000907151	0.000258302
S	0.015573619	0.002870947	0.008285373	0.003260738	0.001449541	0.00201539	0.002182832	0.005957828	0.000964445	0.000825708
T	0.009799886	0.001300199	0.004232217	0.001140498	0.000528247	0.001680542	0.002547679	0.001002723	0.000616722	0.003769141
W	0.000522694	0.002729603	0.00014993	0.000394805	0.000738405	0.000422594	0.000484776	0.001496241	0.000342129	0.000549239
Y	0.001112236	0.000894764	0.002264065	0.000990948	0.000560013	0.000446047	0.000607849	0.000460013	0.005047118	0.001086081
V	0.009268105	0.000590631	0.000409129	0.000463466	0.001031956	0.000590161	0.001822996	0.000831397	0.000154221	0.020216976

From\To	L	K	M	F	P	S	T	W	Y	V
A	0.001829545	0.002998108	0.000929367	0.000431451	0.003511078	0.012497651	0.006902136	8.68013E-05	0.000452894	0.007584945
R	0.002288205	0.017703579	0.000710589	0.000210528	0.001658433	0.004538847	0.001804071	0.000893018	0.000717777	0.000952269
N	0.000604743	0.009964338	0.000206179	0.000197104	0.000476135	0.014734985	0.006605857	5.51781E-05	0.002043088	0.000742028
D	0.000389917	0.001587457	0.000107919	9.57837E-05	0.00103482	0.003973692	0.001219822	9.95638E-05	0.000612759	0.000575995
C	0.001766885	0.000244919	0.000406159	0.000815825	0.000267023	0.005219086	0.001669261	0.000550172	0.001023111	0.003789202
Q	0.00399776	0.012885117	0.001607298	0.000204808	0.002278087	0.003814672	0.002791715	0.000165524	0.00042839	0.001139177
E	0.000709275	0.008549817	0.000327775	0.000166301	0.001665428	0.002613655	0.002677294	0.000120118	0.000369304	0.002226057
G	0.000281864	0.001235805	0.00018109	0.000102344	0.000594483	0.004974976	0.000734865	0.00025855	0.00019491	0.000708002
H	0.00229644	0.002945729	0.000420366	0.001392512	0.001699215	0.002744275	0.001540151	0.000201456	0.007287089	0.000447525
I	0.014579573	0.001071302	0.004428385	0.002171604	0.000243897	0.001184366	0.004744883	0.000163028	0.000790464	0.029573198
L	-	0.000852044	0.005048895	0.00433548	0.001014953	0.001278166	0.001062835	0.000510459	0.000749919	0.006807284
K	0.001184193	-	0.000971785	0.000182088	0.00135922	0.003585763	0.004513262	0.000105501	0.000250709	0.00115488
M	0.022317947	0.003090774	-	0.002440443	0.000418164	0.001831218	0.004933486	0.000395676	0.000806017	0.007783227
F	0.009725187	0.000293887	0.00123843	-	0.000394037	0.002024112	0.000559376	0.001173617	0.012142414	0.002457313
P	0.001911979	0.001842325	0.000178207	0.000330913	-	0.00598145	0.002588196	0.000106959	0.000406447	0.001190622
S	0.00158505	0.003199462	0.000513734	0.001118999	0.003937542	-	0.014246168	0.000401841	0.001480567	0.000880012
T	0.001501752	0.00458841	0.001576988	0.000352351	0.001941299	0.01623209	-	8.50604E-05	0.000547736	0.005249051
W	0.003058976	0.000454894	0.00053641	0.003135315	0.000340247	0.001941843	0.000360754	-	0.004675755	0.0013815
Y	0.001832777	0.000440864	0.000445638	0.013229387	0.000527305	0.002917881	0.000947402	0.001906916	-	0.001190029
V	0.008277653	0.001010437	0.002141091	0.001332089	0.000768547	0.000862911	0.004517329	0.000280329	0.0005921	-

From\To	A	R	N	D	C	Q	E	G	H	I	L	K	M	F	P	S	T	W	Y	V
A	-	0.000817463	0.001017768	0.000223977	0.002656196	0.001029232	0.002609543	0.004578523	0.000434685	3.6038E-05	0.00243137	0.001514657	0.001870897	1.52326E-05	0.003505981	0.011914296	0.008517278	6.66092E-05	0.000928283	0.006472136
R	0.001165741	-	0.000698468	0.000671931	0.000556652	0.007108135	0.000322166	0.001390514	0.001304055	0.000864912	0.000972548	0.02363662	0.000453792	0.000106628	0.000867459	0.002641866	0.002112813	0.000173184	0.00079326	0.000952751
N	0.001748612	0.000841506	-	0.008600712	0.000774472	0.007590587	0.00254511	0.003188008	0.00463664	0.00126133	0.000810457	0.004902705	7.96126E-06	0.000746395	0.001192757	0.007614789	0.004885881	0.00021315	0.000472581	0.000689923
D	0.000342865	0.00072129	0.007663194	-	6.05056E-06	0.002508753	0.017461387	0.002068814	0.001318544	3.6038E-05	0.000270152	0.000797188	7.96126E-06	1.52326E-05	0.001987928	0.004221805	0.001815699	1.33218E-05	1.68779E-05	0.000197121
C	0.015051777	0.002211957	0.002554398	2.23977E-05	-	0.002251445	3.22166E-05	0.001627919	0.001796698	0.003747951	0.005943349	0.00063775	0.001241957	0.001066279	3.61441E-05	0.001942548	0.003862487	0.000732702	0.002211002	0.009691777
Q	0.001097168	0.005313507	0.004709671	0.00174702	0.000423539	-	0.011984568	0.00061047	0.005607436	0.001189254	0.002917644	0.012316553	0.00125788	1.52326E-05	0.002457801	0.005827645	0.004819855	0.000133218	0.000759505	0.001182725
E	0.002777207	0.00024043	0.001576543	0.012139547	6.05056E-06	0.011964824	-	0.002374049	0.000492643	3.6038E-05	0.001134639	0.005620174	7.96126E-06	1.52326E-05	0.001879495	0.002460561	0.002707042	0.000226471	1.68779E-05	0.001149872
G	0.004628679	0.000985764	0.001875886	0.001366259	0.000290427	0.000578943	0.002255161	-	0.000985286	3.6038E-05	0.000162091	0.001195782	0.000294567	0.000106628	0.00061445	0.003936898	0.000231089	0.000306403	0.000354435	9.85604E-05
H	0.001028595	0.002163871	0.006385995	0.00203819	0.000750269	0.012447277	0.001095364	0.002306219	-	0.001225292	0.002755553	0.003029314	0.000923507	0.00214779	0.001590342	0.004739818	0.001617623	0.000639449	0.005181509	3.28535E-05
I	3.42865E-05	0.000577032	0.000698468	2.23977E-05	0.000629258	0.001061396	3.22166E-05	3.3915E-05	0.000492643	-	0.02080172	0.001355219	0.002985474	0.000974883	0.000361441	0.000103603	0.002376915	0.000519552	0.000438825	0.034430449
L	0.001542893	0.000432774	0.000299344	0.000111988	0.000665562	0.001736829	0.000676548	0.000101745	0.000738964	0.013874625	-	0.000916766	0.004625494	0.002726627	0.000795171	0.000621615	0.000825318	0.000626127	0.001080184	0.00367959
K	0.001302887	0.014257509	0.002454617	0.000447954	9.6809E-05	0.009938523	0.004542538	0.001017449	0.001101202	0.001225292	0.0012427	-	0.001066809	0.000213256	0.001554198	0.00199435	0.003631398	7.99311E-05	1.68779E-05	0.000624216
M	0.00805733	0.001370452	1.99562E-05	2.23977E-05	0.000943887	0.005081834	3.22166E-05	0.001254854	0.001680782	0.013514245	0.031391687	0.005341159	-	0.00376244	3.61441E-05	2.59006E-05	0.004324665	0.001478725	0.001248963	0.007753422
F	3.42865E-05	0.000168301	0.000977855	2.23977E-05	0.000423539	3.21635E-05	3.22166E-05	0.000237405	0.002043019	0.002306431	0.009671449	0.000558032	0.001966432	-	0.000397586	0.000518013	0.002277877	0.002424576	0.017164803	0.00302252
P	0.003325791	0.000577032	0.000658556	0.001231873	6.05056E-06	0.002187118	0.001675262	0.000576555	0.000637538	0.00036038	0.00118867	0.001713954	7.96126E-06	0.000167558	-	0.003470686	0.002046788	0.000119897	0.00023629	0.000821337
S	0.015771794	0.002452388	0.005867133	0.003650823	0.000453792	0.007236789	0.003060575	0.005155077	0.002651578	0.000144152	0.001296731	0.003069173	7.96126E-06	0.000304651	0.004843314	-	0.022151526	0.000186506	0.000523214	0.001281286
T	0.008845919	0.001538753	0.002953523	0.001231873	0.000707916	0.004695872	0.00264176	0.000237405	0.000709985	0.002594735	0.001350761	0.004384533	0.001042926	0.001051046	0.002240936	0.017379332	-	1.33218E-05	0.000573848	0.006439282
W	0.000171433	0.000312559	0.0003193	2.23977E-05	0.000332781	0.000321635	0.000547682	0.000780045	0.000695496	0.001405481	0.002539431	0.000239156	0.0008837	0.002772324	0.000325297	0.000362609	3.30127E-05	-	0.002970507	0.000854191
Y	0.001885758	0.001130022	0.000558775	2.23977E-05	0.000792623	0.001447358	3.22166E-05	0.000712215	0.004448276	0.000936988	0.003457948	3.98594E-05	0.000589134	0.015491505	0.000506018	0.00080292	0.001122432	0.002344645	-	0.001938355
V	0.006754442	0.000697247	0.000419081	0.000134386	0.001784915	0.001157886	0.00112758	0.000101745	1.44895E-05	0.03776781	0.00605141	0.000757328	0.001878858	0.001401395	0.000903603	0.001010125	0.006470491	0.000346368	0.000995795	-

From\To	A	R	N	D	C	Q	E	G	H	I	L	K	M	F	P	S	T	W	Y	V
A	-	0.001293534	0.001062918	0.002248338	0.001057597	0.001779796	0.00490127	0.006290392	0.000412993	0.000499744	0.001829545	0.002998108	0.000929367	0.000431451	0.003511078	0.012497651	0.006902136	8.68013E-05	0.000452894	0.007584945
R	0.002548351	-	0.001324553	0.00044816	0.000543906	0.005946053	0.001359843	0.002595977	0.002784721	0.000483315	0.002288205	0.017703579	0.000710589	0.000210528	0.001658433	0.004538847	0.001804071	0.000893018	0.000717777	0.000952269
N	0.002355584	0.001490001	-	0.016518547	0.000273148	0.003023741	0.002932984	0.00499761	0.005155072	0.001432623	0.000604743	0.009964338	0.000206179	0.000197104	0.000476135	0.014734985	0.006605857	5.51781E-05	0.002043088	0.000742028
D	0.003414296	0.000345454	0.011319116	-	3.11962E-05	0.001208178	0.019118189	0.003843288	0.001212677	0.000101939	0.000389917	0.001587457	0.000107919	9.57837E-05	0.00103482	0.003973692	0.001219822	9.95638E-05	0.000612759	0.000575995
C	0.004745098	0.001238703	0.000552999	9.21696E-05	-	0.000193568	6.61161E-05	0.001361666	0.000324412	0.000439775	0.001766885	0.000244919	0.000406159	0.000815825	0.000267023	0.005219086	0.001669261	0.000550172	0.001023111	0.003789202
Q	0.004197876	0.007118796	0.003218141	0.00187651	0.000101758	-	0.016936127	0.001465467	0.005595254	0.00029446	0.00399776	0.012885117	0.001607298	0.000204808	0.002278087	0.003814672	0.002791715	0.000165524	0.00042839	0.001139177
E	0.007313034	0.001029902	0.001974694	0.018784355	2.19873E-05	0.010713806	-	0.002520726	0.000742746	0.000329298	0.000709275	0.008549817	0.000327775	0.000166301	0.001665428	0.002613655	0.002677294	0.000120118	0.000369304	0.002226057
G	0.006545486	0.001371145	0.002346539	0.002633465	0.000315798	0.000646518	0.001757927	-	0.000324983	7.87093E-05	0.000281864	0.001235805	0.00018109	0.000102344	0.000594483	0.004974976	0.000734865	0.00025855	0.00019491	0.000708002
H	0.001464381	0.005012003	0.008247972	0.002831502	0.00025638	0.008411466	0.001765073	0.001107408	-	0.000357202	0.00229644	0.002945729	0.000420366	0.001392512	0.001699215	0.002744275	0.001540151	0.000201456	0.007287089	0.000447525
I	0.00089324	0.0004385	0.001155457	0.000119984	0.000175197	0.000223145	0.000394477	0.000135202	0.000180062	-	0.014579573	0.001071302	0.004428385	0.002171604	0.000243897	0.001184366	0.004744883	0.000163028	0.000790464	0.029573198
L	0.001838435	0.001167128	0.000274206	0.000258011	0.000395721	0.001703188	0.000477673	0.000272195	0.000650802	0.008196518	-	0.000852044	0.005048895	0.00433548	0.001014953	0.001278166	0.001062835	0.000510459	0.000749919	0.006807284
K	0.004187097	0.012550046	0.006279361	0.001459918	7.62366E-05	0.007629478	0.008002647	0.001658638	0.001160239	0.000837061	0.001184193	-	0.000971785	0.000182088	0.00135922	0.003585763	0.004513262	0.000105501	0.000250709	0.00115488
M	0.004128102	0.001602138	0.000413246	0.000315663	0.0004021	0.003026914	0.000975776	0.000773023	0.000526598	0.011004944	0.022317947	0.003090774	-	0.002440443	0.000418164	0.001831218	0.004933486	0.000395676	0.000806017	0.007783227
F	0.000972518	0.000240876	0.000200476	0.000142173	0.000409862	0.000195729	0.00025123	0.000221699	0.000885225	0.002738583	0.009725187	0.000293887	0.00123843	-	0.000394037	0.002024112	0.000559376	0.001173617	0.012142414	0.002457313
P	0.006646344	0.001593524	0.0004067	0.001289935	0.000112659	0.001828325	0.002112901	0.001081478	0.000907151	0.000258302	0.001911979	0.001842325	0.000178207	0.000330913	-	0.00598145	0.002588196	0.000106959	0.000406447	0.001190622
S	0.015573619	0.002870947	0.008285373	0.003260738	0.001449541	0.00201539	0.002182832	0.005957828	0.000964445	0.000825708	0.00158505	0.003199462	0.000513734	0.001118999	0.003937542	-	0.014246168	0.000401841	0.001480567	0.000880012
T	0.009799886	0.001300199	0.004232217	0.001140498	0.000528247	0.001680542	0.002547679	0.001002723	0.000616722	0.003769141	0.001501752	0.00458841	0.001576988	0.000352351	0.001941299	0.01623209	-	8.50604E-05	0.000547736	0.005249051
W	0.000522694	0.002729603	0.00014993	0.000394805	0.000738405	0.000422594	0.000484776	0.001496241	0.000342129	0.000549239	0.003058976	0.000454894	0.00053641	0.003135315	0.000340247	0.001941843	0.000360754	-	0.004675755	0.0013815
Y	0.001112236	0.000894764	0.002264065	0.000990948	0.000560013	0.000446047	0.000607849	0.000460013	0.005047118	0.001086081	0.001832777	0.000440864	0.000445638	0.013229387	0.000527305	0.002917881	0.000947402	0.001906916	-	0.001190029
V	0.009268105	0.000590631	0.000409129	0.000463466	0.001031956	0.000590161	0.001822996	0.000831397	0.000154221	0.020216976	0.008277653	0.001010437	0.002141091	0.001332089	0.000768547	0.000862911	0.004517329	0.000280329	0.0005921	-

From\To	A	R	N	D	C	Q	E	G	H	I	L	K	M	F	P	S	T	W	Y	V
A	-	0.001180879	0.001674492	0.001831475	0.002192411	0.002318028	0.004578811	0.004074633	0.000534018	0.000535517	0.002060166	0.003370912	0.001215113	0.000448462	0.003262598	0.008887429	0.007363928	0.000140897	0.000463257	0.006592511
R	0.001540426	-	0.002086665	0.000365067	0.001127524	0.007744209	0.001270378	0.001681557	0.003600761	0.000517911	0.002576643	0.019904957	0.000929068	0.000218828	0.001541065	0.003227701	0.001924774	0.001449556	0.000734201	0.000827671
N	0.001423903	0.001360236	-	0.013455851	0.000566239	0.003938156	0.00274002	0.003237226	0.006665725	0.001535172	0.000680973	0.011203368	0.000269571	0.000204876	0.000442439	0.01047846	0.007047827	8.95657E-05	0.002089838	0.000644939
D	0.002063872	0.000315369	0.017831829	-	6.46702E-05	0.001573545	0.017860388	0.002489509	0.001568042	0.000109236	0.000439068	0.001784852	0.000141101	9.95602E-05	0.000961586	0.002825804	0.001301435	0.000161613	0.00062678	0.000500631
C	0.002868315	0.001130824	0.00087118	7.50805E-05	-	0.000252106	6.17663E-05	0.000882026	0.000419479	0.000471255	0.001989608	0.000275374	0.000531037	0.000847991	0.000248126	0.003711438	0.001780944	0.000893045	0.001046522	0.003293413
Q	0.002537531	0.006498814	0.005069773	0.001528587	0.000210945	-	0.015821886	0.000949264	0.007234898	0.000315537	0.004501694	0.014487336	0.002101481	0.000212884	0.002116866	0.00271272	0.002978497	0.000268681	0.000438192	0.000990124
E	0.00442058	0.000940207	0.00311088	0.015301556	4.55799E-05	0.013953787	-	0.001632813	0.000960402	0.00035287	0.000798682	0.009612957	0.000428554	0.000172858	0.001547565	0.001858643	0.002856421	0.000194977	0.000377755	0.001934794
G	0.003956613	0.001251731	0.003696674	0.002145196	0.000654654	0.000842033	0.001642271	-	0.000420217	8.43434E-05	0.000317394	0.001389473	0.000236768	0.000106379	0.000552411	0.003537844	0.000784032	0.000419682	0.00019937	0.000615365
H	0.000885189	0.004575503	0.012993632	0.002306515	0.000531478	0.010955192	0.001648948	0.000717329	-	0.000382771	0.002585916	0.00331202	0.000549613	0.001447416	0.001578961	0.001951531	0.001643196	0.000327006	0.007453831	0.000388969
I	0.000539946	0.00040031	0.001820276	9.77376E-05	0.000363185	0.000290627	0.000368524	8.75776E-05	0.000232828	-	0.016417386	0.001204514	0.005789946	0.002257226	0.000226636	0.000842236	0.005062343	0.000264628	0.000808552	0.025703762
L	0.001111296	0.001065482	0.000431977	0.000210173	0.000820333	0.002218252	0.000446246	0.000176316	0.000841515	0.008783236	-	0.000957993	0.00660124	0.00450642	0.000943125	0.000908939	0.001133944	0.000828582	0.000767078	0.005916601
K	0.002531015	0.011457053	0.009892336	0.001189235	0.000158039	0.009936722	0.007476146	0.001074391	0.001500238	0.000896979	0.001333466	-	0.001270572	0.000189267	0.001263027	0.002549936	0.004815225	0.00017125	0.000256446	0.001003772
M	0.002495354	0.001462607	0.000651017	0.000257136	0.000833558	0.003942289	0.000911579	0.00050073	0.000680914	0.011792692	0.025131212	0.003475101	-	0.002536666	0.00038857	0.001302231	0.005263564	0.000642265	0.00082446	0.006764849
F	0.000587867	0.000219898	0.000315825	0.000115813	0.000849649	0.000254919	0.000234701	0.000143607	0.001144633	0.002934615	0.010951085	0.000330431	0.001619201	-	0.000366151	0.001439402	0.000596801	0.001905027	0.012420255	0.002135792
P	0.00401758	0.001454743	0.000640704	0.001050769	0.000233544	0.002381231	0.001973892	0.000700532	0.001172984	0.000276792	0.002152992	0.002071411	0.000232999	0.00034396	-	0.004253576	0.002761361	0.000173616	0.000415747	0.001034838
S	0.009413936	0.002620914	0.013052552	0.002656166	0.003004916	0.002624867	0.002039222	0.003859213	0.001247068	0.000884813	0.001784852	0.003597303	0.000671688	0.001163119	0.003658882	-	0.015199318	0.000652273	0.001514445	0.000764869
T	0.005923832	0.001186963	0.00666732	0.000929039	0.001095061	0.002188758	0.002380065	0.000649519	0.000797448	0.004038942	0.001691053	0.005158963	0.002061852	0.000366243	0.001803913	0.011543093	-	0.000138071	0.000560269	0.004562251
W	0.000315958	0.002491879	0.000236196	0.000321604	0.001530721	0.000550391	0.000452882	0.000969198	0.000442387	0.000588554	0.003444572	0.000511459	0.000701336	0.003258934	0.000316167	0.001380899	0.00038489	-	0.004782746	0.001200741
Y	0.000672324	0.000816838	0.003566747	0.000807217	0.001160914	0.000580937	0.000567858	0.000297976	0.006526136	0.001163824	0.002063806	0.000495684	0.000582654	0.013750997	0.000489987	0.002074987	0.001010788	0.003095327	-	0.001034322
V	0.005602381	0.000539193	0.00064453	0.000377535	0.002139257	0.000768632	0.00170306	0.000538542	0.000199415	0.021664134	0.009321084	0.001136082	0.002799395	0.00138461	0.000714156	0.00061364	0.004819564	0.000455034	0.000605649	-

From\To	A	R	N	D	C	Q	E	G	H	I	L	K	M	F	P	S	T	W	Y	V
A	-	0.001180879	0.001674492	0.001831475	0.002192411	0.002318028	0.004578811	0.004074633	0.000534018	0.000535517	0.002060166	0.003370912	0.001215113	0.000448462	0.003262598	0.008887429	0.007363928	0.000140897	0.000463257	0.006592511
R	0.001540426	-	0.002086665	0.000365067	0.001127524	0.007744209	0.001270378	0.001681557	0.003600761	0.000517911	0.002576643	0.019904957	0.000929068	0.000218828	0.001541065	0.003227701	0.001924774	0.001449556	0.000734201	0.000827671
N	0.001423903	0.001360236	-	0.013455851	0.000566239	0.003938156	0.00274002	0.003237226	0.006665725	0.001535172	0.000680973	0.011203368	0.000269571	0.000204876	0.000442439	0.01047846	0.007047827	8.95657E-05	0.002089838	0.000644939
D	0.002063872	0.000315369	0.017831829	-	6.46702E-05	0.001573545	0.017860388	0.002489509	0.001568042	0.000109236	0.000439068	0.001784852	0.000141101	9.95602E-05	0.000961586	0.002825804	0.001301435	0.000161613	0.00062678	0.000500631
C	0.002868315	0.001130824	0.00087118	7.50805E-05	-	0.000252106	6.17663E-05	0.000882026	0.000419479	0.000471255	0.001989608	0.000275374	0.000531037	0.000847991	0.000248126	0.003711438	0.001780944	0.000893045	0.001046522	0.003293413
Q	0.002537531	0.006498814	0.005069773	0.001528587	0.000210945	-	0.015821886	0.000949264	0.007234898	0.000315537	0.004501694	0.014487336	0.002101481	0.000212884	0.002116866	0.00271272	0.002978497	0.000268681	0.000438192	0.000990124
E	0.00442058	0.000940207	0.00311088	0.015301556	4.55799E-05	0.013953787	-	0.001632813	0.000960402	0.00035287	0.000798682	0.009612957	0.000428554	0.000172858	0.001547565	0.001858643	0.002856421	0.000194977	0.000377755	0.001934794
G	0.003956613	0.001251731	0.003696674	0.002145196	0.000654654	0.000842033	0.001642271	-	0.000420217	8.43434E-05	0.000317394	0.001389473	0.000236768	0.000106379	0.000552411	0.003537844	0.000784032	0.000419682	0.00019937	0.000615365
H	0.000885189	0.004575503	0.012993632	0.002306515	0.000531478	0.010955192	0.001648948	0.000717329	-	0.000382771	0.002585916	0.00331202	0.000549613	0.001447416	0.001578961	0.001951531	0.001643196	0.000327006	0.007453831	0.000388969
I	0.000539946	0.00040031	0.001820276	9.77376E-05	0.000363185	0.000290627	0.000368524	8.75776E-05	0.000232828	-	0.016417386	0.001204514	0.005789946	0.002257226	0.000226636	0.000842236	0.005062343	0.000264628	0.000808552	0.025703762
L	0.001111296	0.001065482	0.000431977	0.000210173	0.000820333	0.002218252	0.000446246	0.000176316	0.000841515	0.008783236	-	0.000957993	0.00660124	0.00450642	0.000943125	0.000908939	0.001133944	0.000828582	0.000767078	0.005916601
K	0.002531015	0.011457053	0.009892336	0.001189235	0.000158039	0.009936722	0.007476146	0.001074391	0.001500238	0.000896979	0.001333466	-	0.001270572	0.000189267	0.001263027	0.002549936	0.004815225	0.00017125	0.000256446	0.001003772
M	0.002495354	0.001462607	0.000651017	0.000257136	0.000833558	0.003942289	0.000911579	0.00050073	0.000680914	0.011792692	0.025131212	0.003475101	-	0.002536666	0.00038857	0.001302231	0.005263564	0.000642265	0.00082446	0.006764849
F	0.000587867	0.000219898	0.000315825	0.000115813	0.000849649	0.000254919	0.000234701	0.000143607	0.001144633	0.002934615	0.010951085	0.000330431	0.001619201	-	0.000366151	0.001439402	0.000596801	0.001905027	0.012420255	0.002135792
P	0.00401758	0.001454743	0.000640704	0.001050769	0.000233544	0.002381231	0.001973892	0.000700532	0.001172984	0.000276792	0.002152992	0.002071411	0.000232999	0.00034396	-	0.004253576	0.002761361	0.000173616	0.000415747	0.001034838
S	0.009413936	0.002620914	0.013052552	0.002656166	0.003004916	0.002624867	0.002039222	0.003859213	0.001247068	0.000884813	0.001784852	0.003597303	0.000671688	0.001163119	0.003658882	-	0.015199318	0.000652273	0.001514445	0.000764869
T	0.005923832	0.001186963	0.00666732	0.000929039	0.001095061	0.002188758	0.002380065	0.000649519	0.000797448	0.004038942	0.001691053	0.005158963	0.002061852	0.000366243	0.001803913	0.011543093	-	0.000138071	0.000560269	0.004562251
W	0.000315958	0.002491879	0.000236196	0.000321604	0.001530721	0.000550391	0.000452882	0.000969198	0.000442387	0.000588554	0.003444572	0.000511459	0.000701336	0.003258934	0.000316167	0.001380899	0.00038489	-	0.004782746	0.001200741
Y	0.000672324	0.000816838	0.003566747	0.000807217	0.001160914	0.000580937	0.000567858	0.000297976	0.006526136	0.001163824	0.002063806	0.000495684	0.000582654	0.013750997	0.000489987	0.002074987	0.001010788	0.003095327	-	0.001034322
V	0.005602381	0.000539193	0.00064453	0.000377535	0.002139257	0.000768632	0.00170306	0.000538542	0.000199415	0.021664134	0.009321084	0.001136082	0.002799395	0.00138461	0.000714156	0.00061364	0.004819564	0.000455034	0.000605649	-

From\To	A	R	N	D	C	Q	E	G	H	I	L	K	M	F	P	S	T	W	Y	V
A	-	0.001406695	0.001230147	0.002198318	0.000603683	0.001184586	0.003417074	0.006737331	0.000261976	0.000985256	0.001463647	0.001139025	0.000569512	0.000290451	0.005131306	0.013742333	0.013896101	6.26464E-05	0.0002335	0.010057588
R	0.002116925	-	0.000994184	0.000411386	0.001071318	0.006427911	0.001019895	0.005262316	0.003822464	0.000651362	0.001756962	0.020123646	0.000522803	0.000137129	0.001859809	0.003539636	0.001971226	0.00093419	0.000394245	0.000591368
N	0.002225842	0.00119536	-	0.014766812	0.000329754	0.001638467	0.001854868	0.002998704	0.004802048	0.001339627	0.000649204	0.007811056	0.000401888	0.000154572	0.00031945	0.017909783	0.007141243	2.06096E-05	0.00117475	0.000566765
D	0.003295334	0.000409782	0.012233714	-	0.000110983	0.001109827	0.024877209	0.004925927	0.001229347	0.000315874	0.000290263	0.000870788	0.000230503	6.82971E-05	0.000332948	0.002083061	0.001289107	4.26857E-05	0.000759805	0.001084216
C	0.002286859	0.002696767	0.000690372	0.000280464	-	0.000194167	0.000172593	0.002114265	0.000862966	0.000409909	0.000776669	0.000151019	0.000496205	0.001423893	0.000323612	0.007615671	0.001423893	0.000819817	0.003538159	0.00213584
Q	0.002216415	0.007991879	0.001694278	0.001385259	9.59026E-05	-	0.010943547	0.00089509	0.006766458	0.000213117	0.003345934	0.00914271	0.000554104	9.59026E-05	0.004209056	0.001939363	0.00158772	0.00012787	0.000426234	0.000618039
E	0.00425199	0.000843311	0.001275597	0.020650499	5.66932E-05	0.00727799	-	0.004322857	0.000290553	0.000304726	0.000460632	0.005343334	0.0002126	9.21265E-05	0.000503152	0.001105517	0.001006304	8.50398E-05	0.0001063	0.001601583
G	0.006935753	0.003599791	0.00170609	0.003382865	0.000574559	0.00049248	0.003576339	-	0.000240377	0.000146571	0.00032832	0.000832525	0.000158297	0.000105531	0.000545245	0.006630885	0.000961508	0.000404537	8.79428E-05	0.001618147
H	0.00087591	0.008492519	0.008873349	0.002741979	0.000761661	0.012091366	0.000780702	0.000780702	-	0.00049508	0.002551564	0.001618529	0.000399872	0.000952076	0.002989519	0.00262773	0.001447156	9.52076E-05	0.009787342	0.000418913
I	0.001440422	0.000632787	0.001082398	0.000308067	0.000158197	0.000166523	0.000358024	0.000208154	0.00021648	-	0.01102381	0.000624461	0.005861603	0.001631924	0.00025811	0.001432096	0.007743311	9.99137E-05	0.000507895	0.032788341
L	0.001235504	0.000985519	0.000302867	0.000163452	0.000173067	0.001509527	0.000312482	0.000269215	0.000644193	0.00636501	-	0.000451896	0.004682417	0.005254498	0.002778683	0.00209603	0.000826874	0.000394208	0.000403822	0.006062143
K	0.001472118	0.017282661	0.005579326	0.00075078	5.15241E-05	0.006315385	0.005549883	0.001045204	0.00062565	0.000552044	0.000691895	-	0.000758141	5.15241E-05	0.000566765	0.001678214	0.002929514	6.62453E-05	0.000147212	0.000426914
M	0.001871603	0.001141678	0.000729925	0.000505333	0.000430469	0.000973233	0.000561481	0.000505333	0.000393037	0.013176082	0.018229409	0.001927751	-	0.000917085	0.000430469	0.001010665	0.006419597	0.000149728	0.000318172	0.010462258
F	0.000551499	0.000173019	0.000162206	8.65096E-05	0.000713704	9.73233E-05	0.000140578	0.000194647	0.000540685	0.002119486	0.011819378	7.56959E-05	0.000529871	-	0.000389293	0.003341434	0.000421734	0.000400107	0.009191648	0.00204379
P	0.007813811	0.001881906	0.000268844	0.000338223	0.000130086	0.003425589	0.000615739	0.000806531	0.001361563	0.000268844	0.005012633	0.000667773	0.000199465	0.000312206	-	0.009869164	0.003573019	5.20343E-05	0.000190792	0.00072848
S	0.015495387	0.002652132	0.011160789	0.001566877	0.002266834	0.001168736	0.001001774	0.007262861	0.000886185	0.00110452	0.00279983	0.001464131	0.000346768	0.001984283	0.007307812	-	0.014500035	0.000231179	0.001053147	0.001406336
T	0.018266841	0.001721874	0.005188082	0.001130448	0.000494103	0.001115475	0.00106307	0.001227771	0.000568967	0.006962361	0.001287663	0.002979591	0.002567839	0.00029197	0.003084401	0.016904314	-	5.98913E-05	0.000336888	0.003937852
W	0.000336888	0.003338258	6.12524E-05	0.000153131	0.001163796	0.000367515	0.000367515	0.002113209	0.000153131	0.000367515	0.00251135	0.000275636	0.00024501	0.00113317	0.000183757	0.001102544	0.00024501	-	0.001255675	0.000826908
Y	0.000555918	0.000623713	0.001545724	0.001206749	0.002223672	0.000542359	0.000203385	0.000203385	0.006969315	0.000827098	0.001138954	0.00027118	0.000230503	0.011525131	0.000298298	0.002223672	0.000610154	0.000555918	-	0.000569477
V	0.01164802	0.000455104	0.000362764	0.000837655	0.000652975	0.000382551	0.00149063	0.001820415	0.000145106	0.025973897	0.008317187	0.000382551	0.003687001	0.001246589	0.000554039	0.00144446	0.003469342	0.000178084	0.00027702	-

From\To	A	R	N	D	C	Q	E
A	-	0.001406695	0.001230147	0.002198318	0.000603683	0.001184586	0.003417074
R	0.002116925	-	0.000994184	0.000411386	0.001071318	0.006427911	0.001019895
N	0.002225842	0.00119536	-	0.014766812	0.000329754	0.001638467	0.001854868
D	0.003295334	0.000409782	0.012233714	-	0.000110983	0.001109827	0.024877209
C	0.002286859	0.002696767	0.000690372	0.000280464	-	0.000194167	0.000172593
Q	0.002216415	0.007991879	0.001694278	0.001385259	9.59026E-05	-	0.010943547
E	0.00425199	0.000843311	0.001275597	0.020650499	5.66932E-05	0.00727799	-
G	0.006935753	0.003599791	0.00170609	0.003382865	0.000574559	0.00049248	0.003576339
H	0.00087591	0.008492519	0.008873349	0.002741979	0.000761661	0.012091366	0.000780702
I	0.001440422	0.000632787	0.001082398	0.000308067	0.000158197	0.000166523	0.000358024
L	0.001235504	0.000985519	0.000302867	0.000163452	0.000173067	0.001509527	0.000312482
K	0.001472118	0.017282661	0.005579326	0.00075078	5.15241E-05	0.006315385	0.005549883
M	0.001871603	0.001141678	0.000729925	0.000505333	0.000430469	0.000973233	0.000561481
F	0.000551499	0.000173019	0.000162206	8.65096E-05	0.000713704	9.73233E-05	0.000140578
P	0.007813811	0.001881906	0.000268844	0.000338223	0.000130086	0.003425589	0.000615739
S	0.015495387	0.002652132	0.011160789	0.001566877	0.002266834	0.001168736	0.001001774
T	0.018266841	0.001721874	0.005188082	0.001130448	0.000494103	0.001115475	0.00106307
W	0.000336888	0.003338258	6.12524E-05	0.000153131	0.001163796	0.000367515	0.000367515
Y	0.000555918	0.000623713	0.001545724	0.001206749	0.002223672	0.000542359	0.000203385
V	0.01164802	0.000455104	0.000362764	0.000837655	0.000652975	0.000382551	0.00149063

From\To	G	H	I	L	K	M	F
A	0.006737331	0.000261976	0.000985256	0.001463647	0.001139025	0.000569512	0.000290451
R	0.005262316	0.003822464	0.000651362	0.001756962	0.020123646	0.000522803	0.000137129
N	0.002998704	0.004802048	0.001339627	0.000649204	0.007811056	0.000401888	0.000154572
D	0.004925927	0.001229347	0.000315874	0.000290263	0.000870788	0.000230503	6.82971E-05
C	0.002114265	0.000862966	0.000409909	0.000776669	0.000151019	0.000496205	0.001423893
Q	0.00089509	0.006766458	0.000213117	0.003345934	0.00914271	0.000554104	9.59026E-05
E	0.004322857	0.000290553	0.000304726	0.000460632	0.005343334	0.0002126	9.21265E-05
G	-	0.000240377	0.000146571	0.00032832	0.000832525	0.000158297	0.000105531
H	0.000780702	-	0.00049508	0.002551564	0.001618529	0.000399872	0.000952076
I	0.000208154	0.00021648	-	0.01102381	0.000624461	0.005861603	0.001631924
L	0.000269215	0.000644193	0.00636501	-	0.000451896	0.004682417	0.005254498
K	0.001045204	0.00062565	0.000552044	0.000691895	-	0.000758141	5.15241E-05
M	0.000505333	0.000393037	0.013176082	0.018229409	0.001927751	-	0.000917085
F	0.000194647	0.000540685	0.002119486	0.011819378	7.56959E-05	0.000529871	-
P	0.000806531	0.001361563	0.000268844	0.005012633	0.000667773	0.000199465	0.000312206
S	0.007262861	0.000886185	0.00110452	0.00279983	0.001464131	0.000346768	0.001984283
T	0.001227771	0.000568967	0.006962361	0.001287663	0.002979591	0.002567839	0.00029197
W	0.002113209	0.000153131	0.000367515	0.00251135	0.000275636	0.00024501	0.00113317
Y	0.000203385	0.006969315	0.000827098	0.001138954	0.00027118	0.000230503	0.011525131
V	0.001820415	0.000145106	0.025973897	0.008317187	0.000382551	0.003687001	0.001246589

From\To	P	S	T	W	Y	V
A	0.005131306	0.013742333	0.013896101	6.26464E-05	0.0002335	0.010057588
R	0.001859809	0.003539636	0.001971226	0.00093419	0.000394245	0.000591368
N	0.00031945	0.017909783	0.007141243	2.06096E-05	0.00117475	0.000566765
D	0.000332948	0.002083061	0.001289107	4.26857E-05	0.000759805	0.001084216
C	0.000323612	0.007615671	0.001423893	0.000819817	0.003538159	0.00213584
Q	0.004209056	0.001939363	0.00158772	0.00012787	0.000426234	0.000618039
E	0.000503152	0.001105517	0.001006304	8.50398E-05	0.0001063	0.001601583
G	0.000545245	0.006630885	0.000961508	0.000404537	8.79428E-05	0.001618147
H	0.002989519	0.00262773	0.001447156	9.52076E-05	0.009787342	0.000418913
I	0.00025811	0.001432096	0.007743311	9.99137E-05	0.000507895	0.032788341
L	0.002778683	0.00209603	0.000826874	0.000394208	0.000403822	0.006062143
K	0.000566765	0.001678214	0.002929514	6.62453E-05	0.000147212	0.000426914
M	0.000430469	0.001010665	0.006419597	0.000149728	0.000318172	0.010462258
F	0.000389293	0.003341434	0.000421734	0.000400107	0.009191648	0.00204379
P	-	0.009869164	0.003573019	5.20343E-05	0.000190792	0.00072848
S	0.007307812	-	0.014500035	0.000231179	0.001053147	0.001406336
T	0.003084401	0.016904314	-	5.98913E-05	0.000336888	0.003937852
W	0.000183757	0.001102544	0.00024501	-	0.001255675	0.000826908
Y	0.000298298	0.002223672	0.000610154	0.000555918	-	0.000569477
V	0.000554039	0.00144446	0.003469342	0.000178084	0.00027702	-

From\To	A	R	N	D	C	Q	E	G	H	I
A	-	0.001122774	0.001811006	0.002023206	0.001209364	0.001400815	0.003047111	0.004941754	0.000365596	0.000988406
R	0.001464631	-	0.001463624	0.000378617	0.002146182	0.007601237	0.000909472	0.003859848	0.005334384	0.000653444
N	0.001539986	0.000954093	-	0.013590533	0.0006606	0.001937546	0.001654044	0.002199514	0.006701427	0.001343909
D	0.002279933	0.000327074	0.018010311	-	0.000222333	0.001312411	0.022183781	0.00361311	0.001715598	0.000316884
C	0.001582202	0.002152464	0.001016357	0.000258123	-	0.00022961	0.000153907	0.001550789	0.001204299	0.000411219
Q	0.001533464	0.006378834	0.002494294	0.001274914	0.000192122	-	0.009758701	0.000656538	0.009442831	0.000213798
E	0.002941811	0.000673101	0.001877917	0.019005543	0.000113574	0.008606486	-	0.003170765	0.000405477	0.0003057
G	0.004798618	0.002873225	0.002511683	0.003113396	0.00115102	0.000582375	0.003189133	-	0.000335454	0.00014704
H	0.000606013	0.006778426	0.013063227	0.002523561	0.001525842	0.014298477	0.000696177	0.000572636	-	0.000496662
I	0.000996581	0.000505068	0.001593492	0.000283528	0.000316917	0.000196919	0.000319261	0.000152678	0.000302105	-
L	0.000854805	0.000786606	0.000445876	0.000150432	0.000346706	0.00178507	0.00027865	0.000197466	0.000898994	0.006385356
K	0.00101851	0.013794405	0.008213809	0.000690975	0.000103219	0.00746817	0.004949004	0.000766645	0.000873117	0.000553809
M	0.0012949	0.000911246	0.001074586	0.000465079	0.000862362	0.001150884	0.00050069	0.000370656	0.000548496	0.0132182
F	0.000381564	0.000138098	0.000238797	7.96185E-05	0.001429771	0.000115088	0.000125358	0.000142771	0.000754545	0.002126261
P	0.005406117	0.00150207	0.000395788	0.000311281	0.000260602	0.004050882	0.000549073	0.000591581	0.001900109	0.000269703
S	0.010720746	0.002116837	0.016430765	0.001442064	0.00454117	0.001382073	0.000893313	0.005327224	0.001236702	0.001108051
T	0.012638223	0.001374339	0.007637826	0.0010404	0.000989841	0.00131909	0.000947973	0.000900556	0.000794014	0.006984617
W	0.000233082	0.002664479	9.0175E-05	0.000140933	0.002331444	0.000434599	0.000327724	0.001550014	0.0002137	0.000368689
Y	0.000384621	0.000497826	0.002275594	0.001110623	0.004454703	0.000641359	0.000181364	0.00014918	0.009725925	0.000829742
V	0.00805888	0.000363248	0.000534056	0.00077093	0.001308111	0.00045238	0.001329241	0.001335253	0.0002025	0.026056924

From\To	L	K	M	F	P	S	T	W	Y	V
A	0.001584671	0.001356494	0.000630552	0.000291081	0.004390203	0.010121152	0.015710603	0.00010394	0.000265019	0.009483156
R	0.00190224	0.023965765	0.000578836	0.000137426	0.001591201	0.002606922	0.002228621	0.00154996	0.000447462	0.000557592
N	0.000702885	0.009302387	0.000444962	0.000154908	0.000273312	0.013190456	0.00807372	3.41945E-05	0.001333322	0.000534395
D	0.000314263	0.001037043	0.000255207	6.84452E-05	0.000284861	0.001534163	0.001457434	7.08219E-05	0.000862366	0.001022292
C	0.000840889	0.000179852	0.000549387	0.001426981	0.000276873	0.005608899	0.00160982	0.001360199	0.004015752	0.002013853
Q	0.003622598	0.010888287	0.000613491	9.61105E-05	0.003601152	0.00142833	0.001795039	0.000212155	0.000483768	0.00058274
E	0.000498721	0.006363514	0.000235386	9.23262E-05	0.000430483	0.000814207	0.001137704	0.000141094	0.000120648	0.00151011
G	0.000355467	0.000991475	0.000175263	0.00010576	0.000466497	0.00488361	0.001087058	0.000671187	9.98136E-05	0.001525728
H	0.002762545	0.001927548	0.000442729	0.000954141	0.002557749	0.001935309	0.00163612	0.000157964	0.011108473	0.000394988
I	0.011935335	0.000743686	0.006489838	0.001635462	0.000220832	0.001054731	0.008754404	0.000165772	0.000576452	0.030915659
L	-	0.000538175	0.005184269	0.005265893	0.002377363	0.001543715	0.000934845	0.000654049	0.000458332	0.005715908
K	0.000749106	-	0.000839397	5.16358E-05	0.000484909	0.001235995	0.003312039	0.000109911	0.000167083	0.000402531
M	0.019736742	0.002295808	-	0.000919074	0.000368297	0.000744349	0.007257844	0.000248421	0.00036112	0.009864714
F	0.012796686	9.01482E-05	0.000586662	-	0.000333069	0.002460948	0.000476803	0.000663837	0.01043237	0.00192706
P	0.005427113	0.000795268	0.000220843	0.000312883	-	0.007268584	0.004039571	8.63326E-05	0.000216546	0.000686873
S	0.003031339	0.001743671	0.000383934	0.001988586	0.006252361	-	0.016393396	0.00038356	0.001195304	0.001326015
T	0.001394135	0.003548471	0.002843055	0.000292603	0.002638928	0.012449933	-	9.93685E-05	0.000382363	0.003712944
W	0.002719006	0.000328262	0.000271269	0.001135628	0.000157218	0.000812018	0.000277002	-	0.001425171	0.00077968
Y	0.001233131	0.000322955	0.000255207	0.011550124	0.000255215	0.001637722	0.000689826	0.000922351	-	0.000536952
V	0.00900491	0.00045559	0.004082166	0.001249292	0.000474021	0.001063837	0.003922356	0.000295468	0.000314413	-

From\To	A	R	N	D	C	Q	E
A	-	0.001122774	0.001811006	0.002023206	0.001209364	0.001400815	0.003047111
R	0.001464631	-	0.001463624	0.000378617	0.002146182	0.007601237	0.000909472
N	0.001539986	0.000954093	-	0.013590533	0.0006606	0.001937546	0.001654044
D	0.002279933	0.000327074	0.018010311	-	0.000222333	0.001312411	0.022183781
C	0.001582202	0.002152464	0.001016357	0.000258123	-	0.00022961	0.000153907
Q	0.001533464	0.006378834	0.002494294	0.001274914	0.000192122	-	0.009758701
E	0.002941811	0.000673101	0.001877917	0.019005543	0.000113574	0.008606486	-
G	0.004798618	0.002873225	0.002511683	0.003113396	0.00115102	0.000582375	0.003189133
H	0.000606013	0.006778426	0.013063227	0.002523561	0.001525842	0.014298477	0.000696177
I	0.000996581	0.000505068	0.001593492	0.000283528	0.000316917	0.000196919	0.000319261
L	0.000854805	0.000786606	0.000445876	0.000150432	0.000346706	0.00178507	0.00027865
K	0.00101851	0.013794405	0.008213809	0.000690975	0.000103219	0.00746817	0.004949004
M	0.0012949	0.000911246	0.001074586	0.000465079	0.000862362	0.001150884	0.00050069
F	0.000381564	0.000138098	0.000238797	7.96185E-05	0.001429771	0.000115088	0.000125358
P	0.005406117	0.00150207	0.000395788	0.000311281	0.000260602	0.004050882	0.000549073
S	0.010720746	0.002116837	0.016430765	0.001442064	0.00454117	0.001382073	0.000893313
T	0.012638223	0.001374339	0.007637826	0.0010404	0.000989841	0.00131909	0.000947973
W	0.000233082	0.002664479	9.0175E-05	0.000140933	0.002331444	0.000434599	0.000327724
Y	0.000384621	0.000497826	0.002275594	0.001110623	0.004454703	0.000641359	0.000181364
V	0.00805888	0.000363248	0.000534056	0.00077093	0.001308111	0.00045238	0.001329241

From\To	G	H	I	L	K	M	F
A	0.004941754	0.000365596	0.000988406	0.001584671	0.001356494	0.000630552	0.000291081
R	0.003859848	0.005334384	0.000653444	0.00190224	0.023965765	0.000578836	0.000137426
N	0.002199514	0.006701427	0.001343909	0.000702885	0.009302387	0.000444962	0.000154908
D	0.00361311	0.001715598	0.000316884	0.000314263	0.001037043	0.000255207	6.84452E-05
C	0.001550789	0.001204299	0.000411219	0.000840889	0.000179852	0.000549387	0.001426981
Q	0.000656538	0.009442831	0.000213798	0.003622598	0.010888287	0.000613491	9.61105E-05
E	0.003170765	0.000405477	0.0003057	0.000498721	0.006363514	0.000235386	9.23262E-05
G	-	0.000335454	0.00014704	0.000355467	0.000991475	0.000175263	0.00010576
H	0.000572636	-	0.000496662	0.002762545	0.001927548	0.000442729	0.000954141
I	0.000152678	0.000302105	-	0.011935335	0.000743686	0.006489838	0.001635462
L	0.000197466	0.000898994	0.006385356	-	0.000538175	0.005184269	0.005265893
K	0.000766645	0.000873117	0.000553809	0.000749106	-	0.000839397	5.16358E-05
M	0.000370656	0.000548496	0.0132182	0.019736742	0.002295808	-	0.000919074
F	0.000142771	0.000754545	0.002126261	0.012796686	9.01482E-05	0.000586662	-
P	0.000591581	0.001900109	0.000269703	0.005427113	0.000795268	0.000220843	0.000312883
S	0.005327224	0.001236702	0.001108051	0.003031339	0.001743671	0.000383934	0.001988586
T	0.000900556	0.000794014	0.006984617	0.001394135	0.003548471	0.002843055	0.000292603
W	0.001550014	0.0002137	0.000368689	0.002719006	0.000328262	0.000271269	0.001135628
Y	0.00014918	0.009725925	0.000829742	0.001233131	0.000322955	0.000255207	0.011550124
V	0.001335253	0.0002025	0.026056924	0.00900491	0.00045559	0.004082166	0.001249292

From\To	P	S	T	W	Y	V
A	0.004390203	0.010121152	0.015710603	0.00010394	0.000265019	0.009483156
R	0.001591201	0.002606922	0.002228621	0.00154996	0.000447462	0.000557592
N	0.000273312	0.013190456	0.00807372	3.41945E-05	0.001333322	0.000534395
D	0.000284861	0.001534163	0.001457434	7.08219E-05	0.000862366	0.001022292
C	0.000276873	0.005608899	0.00160982	0.001360199	0.004015752	0.002013853
Q	0.003601152	0.00142833	0.001795039	0.000212155	0.000483768	0.00058274
E	0.000430483	0.000814207	0.001137704	0.000141094	0.000120648	0.00151011
G	0.000466497	0.00488361	0.001087058	0.000671187	9.98136E-05	0.001525728
H	0.002557749	0.001935309	0.00163612	0.000157964	0.011108473	0.000394988
I	0.000220832	0.001054731	0.008754404	0.000165772	0.000576452	0.030915659
L	0.002377363	0.001543715	0.000934845	0.000654049	0.000458332	0.005715908
K	0.000484909	0.001235995	0.003312039	0.000109911	0.000167083	0.000402531
M	0.000368297	0.000744349	0.007257844	0.000248421	0.00036112	0.009864714
F	0.000333069	0.002460948	0.000476803	0.000663837	0.01043237	0.00192706
P	-	0.007268584	0.004039571	8.63326E-05	0.000216546	0.000686873
S	0.006252361	-	0.016393396	0.00038356	0.001195304	0.001326015
T	0.002638928	0.012449933	-	9.93685E-05	0.000382363	0.003712944
W	0.000157218	0.000812018	0.000277002	-	0.001425171	0.00077968
Y	0.000255215	0.001637722	0.000689826	0.000922351	-	0.000536952
V	0.000474021	0.001063837	0.003922356	0.000295468	0.000314413	-

From\To	A	R	N	D	C	Q	E	G	H	I	L	K	M	F	P	S	T	W	Y	V
A	-	0.000551931	0.003114373	0.002860149	0.000739072	0.002177279	0.005463519	0.006605904	0.000370103	0.001741257	0.002024234	0.000925666	0.000936576	0.000376284	0.005424164	0.010278637	0.012306244	0	0.000452402	0.006516124
R	0.00071998	-	0.001034398	0	0.000476944	0.005982837	0	0.000242968	0.003865762	0.001685525	0.000771402	0.016496529	0.001169201	0.000280465	0.002243279	0.003884487	0.000888379	0.002396979	0.000144514	0.000760364
N	0.002648303	0.000674294	-	0.0213018	0	0.002523701	0.004162512	0.003837206	0.008587134	0.002047662	0.001699713	0.011273844	0	0.000283936	0.000915196	0.012400488	0.00759971	0.000269627	0.001755633	0.000500353
D	0.003223075	0	0.028229361	-	0	0.00330438	0.031698386	0.003432528	0.001407398	0.000636953	0	0.002563561	0	0	0.000291984	0.002423202	0.002207973	0	0	0.000563626
C	0.000966923	0.00047834	0	0	-	0	0	0.000296638	0.000458222	0.001166115	0	0	0	0	0.000408778	0.004050209	0.000542309	0	0.001764368	0.001531735
Q	0.002383454	0.005020699	0.003248879	0.003209969	0	-	0.019711635	0.000778385	0.009739321	0.000479986	0.003634274	0.005428953	0.001468908	0	0.003325187	0.0014231	0.001755071	0	0	0.001096174
E	0.005274715	0	0.004725904	0.027157005	0	0.017384271	-	0.002248458	0.000713253	0.0016248	0.000562395	0.002971841	0.000397792	0	0.00110659	0.002014789	0.001137753	0	0.000398022	0.001162281
G	0.006414568	0.000180862	0.004381806	0.00295779	0.00022017	0.000690457	0.002261483	-	0.000173256	0	0.000356099	0.000957889	0.000222243	0.000314427	0.000757346	0.005889998	0.001025246	0	0	0.001702365
H	0.000613483	0.004912241	0.016739073	0.002070214	0.000580566	0.014747426	0.001224609	0.000295755	-	0.000205173	0.002209408	0.000968247	0	0.000975428	0.002037805	0.000897368	0.000756973	0.000324195	0.002345489	0.001388343
I	0.001755659	0.001302797	0.002427942	0.000569906	0.000898698	0.000442092	0.001696878	0	0.0001248	-	0.012724752	0.001648313	0.004345221	0.003996876	0.000259779	0.00062855	0.006351188	0	0.000694112	0.027854139
L	0.001091914	0.000318987	0.001078217	0	0	0.001790823	0.000314226	0.000197817	0.000718991	0.006807691	-	0.000645958	0.006818296	0.003204521	0.000689513	0.000434539	0.001106209	0.000552725	0.000530619	0.005516954
K	0.000695027	0.009495202	0.009954564	0.001708083	0	0.003723666	0.002311247	0.000740674	0.000438584	0.001227467	0.000899133	-	0.003144923	0	0.000731483	0.002420192	0.004513628	0	0.000244747	0.000328373
M	0.001923352	0.00184064	0	0	0	0.002755609	0.000846146	0.000470011	0	0.008850144	0.025957556	0.008601581	-	0.001882312	0.000370109	0.001567129	0.003437067	0	0	0.008089909
F	0.000493253	0.000281836	0.000437701	0	0	0	0	0.000424461	0.000771379	0.005196329	0.00778733	0	0.001201515	-	0.00024085	0.001165503	0.000456466	0.000912306	0.012870724	0.000390689
P	0.006679342	0.002117623	0.001325312	0.000319064	0.000384754	0.003740453	0.001411435	0.000960417	0.001513851	0.000317269	0.00157404	0.001199659	0.00022193	0.000226253	-	0.006152912	0.002615812	0	0	0.001533475
S	0.010887561	0.003154228	0.015446737	0.002277733	0.003279198	0.001377012	0.002210539	0.00642503	0.000573436	0.000660325	0.000853289	0.003414267	0.000808322	0.000941793	0.005292671	-	0.018167353	0.000886877	0.000622768	0.000960669
T	0.009899624	0.000547843	0.007189407	0.001576177	0.000333453	0.001289719	0.000948014	0.000849349	0.000367361	0.005067235	0.001649691	0.004835837	0.001346373	0.000280123	0.00170883	0.013797162	-	0	0.000774613	0.004943923
W	0	0.00412056	0.00071104	0	0	0	0	0	0.000438584	0	0.002297784	0	0	0.001560685	0	0.001877569	0	-	0.001125835	0
Y	0.00065657	0.00016078	0.002996357	0	0.001957226	0	0.000598326	0	0.002053572	0.000999101	0.001427617	0.000473071	0	0.01424973	0	0.000853273	0.001397489	0.000728625	-	0.000884082
V	0.005537467	0.000495345	0.000500036	0.000425041	0.000994949	0.000850959	0.001023073	0.001489839	0.000711767	0.023476555	0.008691476	0.000371657	0.003347725	0.00025328	0.001058273	0.000770727	0.005222763	0	0.000517675	-

From\To	A	R	N	D	C	Q	E	G	H	I	L	K	M	F	P	S	T	W	Y	V
A	-	0.000551931	0.003114373	0.002860149	0.000739072	0.002177279	0.005463519	0.006605904	0.000370103	0.001741257	0.002024234	0.000925666	0.000936576	0.000376284	0.005424164	0.010278637	0.012306244	0	0.000452402	0.006516124
R	0.00071998	-	0.001034398	0	0.000476944	0.005982837	0	0.000242968	0.003865762	0.001685525	0.000771402	0.016496529	0.001169201	0.000280465	0.002243279	0.003884487	0.000888379	0.002396979	0.000144514	0.000760364
N	0.002648303	0.000674294	-	0.0213018	0	0.002523701	0.004162512	0.003837206	0.008587134	0.002047662	0.001699713	0.011273844	0	0.000283936	0.000915196	0.012400488	0.00759971	0.000269627	0.001755633	0.000500353
D	0.003223075	0	0.028229361	-	0	0.00330438	0.031698386	0.003432528	0.001407398	0.000636953	0	0.002563561	0	0	0.000291984	0.002423202	0.002207973	0	0	0.000563626
C	0.000966923	0.00047834	0	0	-	0	0	0.000296638	0.000458222	0.001166115	0	0	0	0	0.000408778	0.004050209	0.000542309	0	0.001764368	0.001531735
Q	0.002383454	0.005020699	0.003248879	0.003209969	0	-	0.019711635	0.000778385	0.009739321	0.000479986	0.003634274	0.005428953	0.001468908	0	0.003325187	0.0014231	0.001755071	0	0	0.001096174
E	0.005274715	0	0.004725904	0.027157005	0	0.017384271	-	0.002248458	0.000713253	0.0016248	0.000562395	0.002971841	0.000397792	0	0.00110659	0.002014789	0.001137753	0	0.000398022	0.001162281
G	0.006414568	0.000180862	0.004381806	0.00295779	0.00022017	0.000690457	0.002261483	-	0.000173256	0	0.000356099	0.000957889	0.000222243	0.000314427	0.000757346	0.005889998	0.001025246	0	0	0.001702365
H	0.000613483	0.004912241	0.016739073	0.002070214	0.000580566	0.014747426	0.001224609	0.000295755	-	0.000205173	0.002209408	0.000968247	0	0.000975428	0.002037805	0.000897368	0.000756973	0.000324195	0.002345489	0.001388343
I	0.001755659	0.001302797	0.002427942	0.000569906	0.000898698	0.000442092	0.001696878	0	0.0001248	-	0.012724752	0.001648313	0.004345221	0.003996876	0.000259779	0.00062855	0.006351188	0	0.000694112	0.027854139
L	0.001091914	0.000318987	0.001078217	0	0	0.001790823	0.000314226	0.000197817	0.000718991	0.006807691	-	0.000645958	0.006818296	0.003204521	0.000689513	0.000434539	0.001106209	0.000552725	0.000530619	0.005516954
K	0.000695027	0.009495202	0.009954564	0.001708083	0	0.003723666	0.002311247	0.000740674	0.000438584	0.001227467	0.000899133	-	0.003144923	0	0.000731483	0.002420192	0.004513628	0	0.000244747	0.000328373
M	0.001923352	0.00184064	0	0	0	0.002755609	0.000846146	0.000470011	0	0.008850144	0.025957556	0.008601581	-	0.001882312	0.000370109	0.001567129	0.003437067	0	0	0.008089909
F	0.000493253	0.000281836	0.000437701	0	0	0	0	0.000424461	0.000771379	0.005196329	0.00778733	0	0.001201515	-	0.00024085	0.001165503	0.000456466	0.000912306	0.012870724	0.000390689
P	0.006679342	0.002117623	0.001325312	0.000319064	0.000384754	0.003740453	0.001411435	0.000960417	0.001513851	0.000317269	0.00157404	0.001199659	0.00022193	0.000226253	-	0.006152912	0.002615812	0	0	0.001533475
S	0.010887561	0.003154228	0.015446737	0.002277733	0.003279198	0.001377012	0.002210539	0.00642503	0.000573436	0.000660325	0.000853289	0.003414267	0.000808322	0.000941793	0.005292671	-	0.018167353	0.000886877	0.000622768	0.000960669
T	0.009899624	0.000547843	0.007189407	0.001576177	0.000333453	0.001289719	0.000948014	0.000849349	0.000367361	0.005067235	0.001649691	0.004835837	0.001346373	0.000280123	0.00170883	0.013797162	-	0	0.000774613	0.004943923
W	0	0.00412056	0.00071104	0	0	0	0	0	0.000438584	0	0.002297784	0	0	0.001560685	0	0.001877569	0	-	0.001125835	0
Y	0.00065657	0.00016078	0.002996357	0	0.001957226	0	0.000598326	0	0.002053572	0.000999101	0.001427617	0.000473071	0	0.01424973	0	0.000853273	0.001397489	0.000728625	-	0.000884082
V	0.005537467	0.000495345	0.000500036	0.000425041	0.000994949	0.000850959	0.001023073	0.001489839	0.000711767	0.023476555	0.008691476	0.000371657	0.003347725	0.00025328	0.001058273	0.000770727	0.005222763	0	0.000517675	-

From\To	A	R	N	D	C	Q	E	G	H	I	L	K	M	F	P	S	T	W	Y	V
A	-	0.000561098	0.002038655	0.002880302	0.000617207	0.001739403	0.004975066	0.010829186	0.000392768	0.001234415	0.001776809	0.001066086	0.000542394	0.000374065	0.006452624	0.014438914	0.011034921	0	0.000374065	0.006826689
R	0.001194905	-	0.000677113	0	0.000398302	0.00477962	0	0.000398302	0.004102507	0.001194905	0.000677113	0.018998988	0.000677113	0.000278811	0.002668621	0.005456732	0.000796603	0.001075414	0.00011949	0.000796603
N	0.004395219	0.000685493	-	0.021451895	0	0.002016156	0.003790372	0.006290405	0.009113023	0.001451632	0.001491955	0.012984041	0	0.000282262	0.001088724	0.017419584	0.006814606	0.000120969	0.001451632	0.0005242
D	0.005349131	0	0.018478818	-	0	0.002639831	0.028864469	0.005627008	0.001493589	0.00045155	0	0.002952443	0	0	0.000347346	0.003403993	0.001979873	0	0	0.000590489
C	0.001604739	0.000486285	0	0	-	0	0	0.000486285	0.000486285	0.000826684	0	0	0	0	0.000486285	0.005689531	0.000486285	0	0.001458854	0.001604739
Q	0.003955666	0.005104085	0.002126702	0.003232587	0	-	0.017949364	0.001276021	0.010335771	0.000340272	0.003190053	0.006252504	0.000850681	0	0.003955666	0.0019991	0.001573759	0	0	0.001148419
E	0.008754106	0	0.003093556	0.027348355	0	0.013888094	-	0.00368594	0.000756934	0.001151856	0.000493653	0.003422658	0.000230371	0	0.001316407	0.002830275	0.001020215	0	0.000329102	0.001217676
G	0.010645847	0.000183866	0.002868311	0.002978631	0.000183866	0.000551598	0.0020593	-	0.000183866	0	0.000312572	0.001103197	0.000128706	0.000312572	0.000900944	0.008273975	0.000919331	0	0	0.001783501
H	0.001018159	0.004993825	0.010957325	0.002084801	0.000484837	0.011781549	0.001115126	0.000484837	-	0.000145451	0.00193935	0.001115126	0	0.000969675	0.002424187	0.001260577	0.000678772	0.000145451	0.00193935	0.001454512
I	0.002913755	0.001324434	0.001589321	0.000573921	0.000750513	0.000353182	0.001545173	0	0.000132443	-	0.011169392	0.001898355	0.002516424	0.003973302	0.000309035	0.000882956	0.005695066	0	0.000573921	0.029181693
L	0.001812179	0.000324285	0.000705796	0	0	0.001430668	0.000286134	0.000324285	0.000763023	0.004826119	-	0.000743947	0.003948643	0.00318562	0.000820249	0.000610418	0.00099193	0.000247982	0.000438738	0.005779898
K	0.001153491	0.009652902	0.006516215	0.001720119	0	0.002974794	0.002104616	0.001214201	0.000465444	0.000870178	0.000789231	-	0.001821302	0	0.000870178	0.003399764	0.004047338	0	0.000202367	0.000344024
M	0.003192065	0.00187121	0	0	0	0.002201424	0.000770498	0.000770498	0	0.006274058	0.022784737	0.009906407	-	0.00187121	0.000440285	0.002201424	0.003081993	0	0	0.008475482
F	0.00081862	0.000286517	0.000286517	0	0	0	0	0.000695827	0.00081862	0.00368379	0.006835476	0	0.000695827	-	0.000286517	0.00163724	0.00040931	0.00040931	0.010642059	0.00040931
P	0.011085276	0.002152793	0.000867543	0.000321312	0.000321312	0.002988205	0.001285249	0.001574431	0.001606562	0.000224919	0.001381643	0.001381643	0.000128525	0.000224919	-	0.008643302	0.00234558	0	0	0.001606562
S	0.018069388	0.003206614	0.010111367	0.002293782	0.002738495	0.001100079	0.002012911	0.010532674	0.000608555	0.000468119	0.00074899	0.003932198	0.000468119	0.000936238	0.006296199	-	0.016290537	0.000397901	0.000514931	0.001006456
T	0.016429772	0.000556941	0.004706155	0.001587283	0.000278471	0.001030342	0.000863259	0.001392354	0.000389859	0.003592272	0.001448048	0.005569414	0.000779718	0.000278471	0.002032836	0.019381561	-	0	0.000640483	0.005179555
W	0	0.004188995	0.000465444	0	0	0	0	0	0.000465444	0	0.002016924	0	0	0.00155148	0	0.002637515	0	-	0.000930888	0
Y	0.001089668	0.00016345	0.001961402	0	0.001634502	0	0.000544834	0	0.002179336	0.000708284	0.001253118	0.000544834	0	0.014165684	0	0.001198635	0.001253118	0.0003269	-	0.000926218
V	0.009190179	0.000503571	0.000327321	0.000428036	0.000830893	0.000679821	0.000931607	0.002442322	0.000755357	0.016643037	0.007629108	0.000428036	0.00193875	0.000251786	0.001258929	0.001082679	0.004683215	0	0.000428036	-

From\To	A	R	N	D	C	Q	E	G	H	I	L
A	-	0.000561098	0.002038655	0.002880302	0.000617207	0.001739403	0.004975066	0.010829186	0.000392768	0.001234415	0.001776809
R	0.001194905	-	0.000677113	0	0.000398302	0.00477962	0	0.000398302	0.004102507	0.001194905	0.000677113
N	0.004395219	0.000685493	-	0.021451895	0	0.002016156	0.003790372	0.006290405	0.009113023	0.001451632	0.001491955
D	0.005349131	0	0.018478818	-	0	0.002639831	0.028864469	0.005627008	0.001493589	0.00045155	0
C	0.001604739	0.000486285	0	0	-	0	0	0.000486285	0.000486285	0.000826684	0
Q	0.003955666	0.005104085	0.002126702	0.003232587	0	-	0.017949364	0.001276021	0.010335771	0.000340272	0.003190053
E	0.008754106	0	0.003093556	0.027348355	0	0.013888094	-	0.00368594	0.000756934	0.001151856	0.000493653
G	0.010645847	0.000183866	0.002868311	0.002978631	0.000183866	0.000551598	0.0020593	-	0.000183866	0	0.000312572
H	0.001018159	0.004993825	0.010957325	0.002084801	0.000484837	0.011781549	0.001115126	0.000484837	-	0.000145451	0.00193935
I	0.002913755	0.001324434	0.001589321	0.000573921	0.000750513	0.000353182	0.001545173	0	0.000132443	-	0.011169392
L	0.001812179	0.000324285	0.000705796	0	0	0.001430668	0.000286134	0.000324285	0.000763023	0.004826119	-
K	0.001153491	0.009652902	0.006516215	0.001720119	0	0.002974794	0.002104616	0.001214201	0.000465444	0.000870178	0.000789231
M	0.003192065	0.00187121	0	0	0	0.002201424	0.000770498	0.000770498	0	0.006274058	0.022784737
F	0.00081862	0.000286517	0.000286517	0	0	0	0	0.000695827	0.00081862	0.00368379	0.006835476
P	0.011085276	0.002152793	0.000867543	0.000321312	0.000321312	0.002988205	0.001285249	0.001574431	0.001606562	0.000224919	0.001381643
S	0.018069388	0.003206614	0.010111367	0.002293782	0.002738495	0.001100079	0.002012911	0.010532674	0.000608555	0.000468119	0.00074899
T	0.016429772	0.000556941	0.004706155	0.001587283	0.000278471	0.001030342	0.000863259	0.001392354	0.000389859	0.003592272	0.001448048
W	0	0.004188995	0.000465444	0	0	0	0	0	0.000465444	0	0.002016924
Y	0.001089668	0.00016345	0.001961402	0	0.001634502	0	0.000544834	0	0.002179336	0.000708284	0.001253118
V	0.009190179	0.000503571	0.000327321	0.000428036	0.000830893	0.000679821	0.000931607	0.002442322	0.000755357	0.016643037	0.007629108

From\To	K	M	F	P	S	T	W	Y	V
A	0.001066086	0.000542394	0.000374065	0.006452624	0.014438914	0.011034921	0	0.000374065	0.006826689
R	0.018998988	0.000677113	0.000278811	0.002668621	0.005456732	0.000796603	0.001075414	0.00011949	0.000796603
N	0.012984041	0	0.000282262	0.001088724	0.017419584	0.006814606	0.000120969	0.001451632	0.0005242
D	0.002952443	0	0	0.000347346	0.003403993	0.001979873	0	0	0.000590489
C	0	0	0	0.000486285	0.005689531	0.000486285	0	0.001458854	0.001604739
Q	0.006252504	0.000850681	0	0.003955666	0.0019991	0.001573759	0	0	0.001148419
E	0.003422658	0.000230371	0	0.001316407	0.002830275	0.001020215	0	0.000329102	0.001217676
G	0.001103197	0.000128706	0.000312572	0.000900944	0.008273975	0.000919331	0	0	0.001783501
H	0.001115126	0	0.000969675	0.002424187	0.001260577	0.000678772	0.000145451	0.00193935	0.001454512
I	0.001898355	0.002516424	0.003973302	0.000309035	0.000882956	0.005695066	0	0.000573921	0.029181693
L	0.000743947	0.003948643	0.00318562	0.000820249	0.000610418	0.00099193	0.000247982	0.000438738	0.005779898
K	-	0.001821302	0	0.000870178	0.003399764	0.004047338	0	0.000202367	0.000344024
M	0.009906407	-	0.00187121	0.000440285	0.002201424	0.003081993	0	0	0.008475482
F	0	0.000695827	-	0.000286517	0.00163724	0.00040931	0.00040931	0.010642059	0.00040931
P	0.001381643	0.000128525	0.000224919	-	0.008643302	0.00234558	0	0	0.001606562
S	0.003932198	0.000468119	0.000936238	0.006296199	-	0.016290537	0.000397901	0.000514931	0.001006456
T	0.005569414	0.000779718	0.000278471	0.002032836	0.019381561	-	0	0.000640483	0.005179555
W	0	0	0.00155148	0	0.002637515	0	-	0.000930888	0
Y	0.000544834	0	0.014165684	0	0.001198635	0.001253118	0.0003269	-	0.000926218
V	0.000428036	0.00193875	0.000251786	0.001258929	0.001082679	0.004683215	0	0.000428036	-

From\To	A	R	N	D	C	Q	E	G	H	I	L	K	M	F	P	S	T	W	Y	V
A	-	0.00064869	0.000934114	0.000643501	0.000596795	0.000596795	0.002433885	0.005506082	0.000160875	0.001183211	0.001972018	0.001178021	0.000404783	0.000342508	0.002086188	0.014899116	0.007166729	2.59476E-05	0.000171254	0.00628451
R	0.000788807	-	0.001458255	0.000166065	0.000731723	0.006564744	0.000778428	0.002018724	0.00173849	0.00108461	0.002029103	0.022133308	0.000269855	0.000264666	0.000373646	0.002371611	0.001691784	0.000409972	0.00097563	0.000607174
N	0.001722921	0.002210736	-	0.016129032	0.000482625	0.002895753	0.005163574	0.005397102	0.003404326	0.001338897	0.001136505	0.011987794	0.000134928	0.000487815	0.000742102	0.012714327	0.007436584	6.74638E-05	0.0022782	0.0005449
D	0.001312949	0.000275045	0.017826006	-	1.0379E-05	0.001525719	0.017950554	0.00357558	0.000809565	8.82219E-05	0.00010379	0.002075809	9.86009E-05	0.000114169	0.000726533	0.003627475	0.001437497	3.11371E-05	0.000851081	0.000487815
C	0.005007888	0.005023457	0.002174409	4.15162E-05	-	4.15162E-05	5.70847E-05	0.002511728	0.001074231	0.002241873	0.003954415	0.000259476	0.000347698	0.003627475	0.001219538	0.014229667	0.003098144	0.000778428	0.004431851	0.003845435
Q	0.001183211	0.010685224	0.003113713	0.001484203	1.0379E-05	-	0.015184539	0.001110558	0.003077386	0.000736912	0.002854237	0.01637294	0.00043592	5.18952E-05	0.001375223	0.002433885	0.00130257	9.34114E-05	0.001183211	0.000358077
E	0.003720887	0.00097563	0.004276166	0.01344086	1.0379E-05	0.011702371	-	0.00313966	0.000399593	0.001183211	0.000825134	0.012989372	0.000243908	0.000721343	0.000793997	0.003492548	0.001987587	0.00010898	0.00043073	0.00130257
G	0.004971561	0.001494582	0.002636277	0.001582804	0.000269855	0.000503384	0.001852659	-	4.67057E-05	0.00032175	0.000202391	0.001312949	4.15162E-05	0.000124549	0.000124549	0.004234649	0.000498194	0.000145307	3.11371E-05	0.000596795
H	0.000498194	0.004385146	0.005661768	0.001219538	0.000394404	0.00476917	0.000799186	0.000160875	-	0.000228339	0.000664259	0.001536098	2.07581E-05	0.000638311	0.00064869	0.001868228	0.000181633	0.000119359	0.00596795	0.000160875
I	0.001110558	0.000835513	0.000679827	3.63266E-05	0.000249097	0.000347698	0.000726533	0.000332129	6.74638E-05	-	0.017364138	0.001712542	0.003840246	0.00227301	0.000498194	0.001333707	0.005557977	7.26533E-05	0.000269855	0.031038527
L	0.001473824	0.001245485	0.000461867	3.63266E-05	0.000352887	0.001074231	0.000404783	0.000166065	0.000160875	0.013840453	-	0.00108461	0.002937269	0.006326026	0.000934114	0.003160418	0.000845892	0.000285424	0.000576037	0.005651389
K	0.001774816	0.027270935	0.009745921	0.001525719	4.67057E-05	0.012397766	0.012761033	0.002184788	0.000747291	0.002740067	0.002179599	-	0.000420351	0.000363266	0.001287001	0.00531407	0.004904098	1.55686E-05	0.000752481	0.001619131
M	0.001385602	0.000768049	0.000249097	0.000171254	0.000140117	0.00075767	0.000555279	0.000171254	2.07581E-05	0.014048034	0.013477187	0.000960061	-	0.001639889	0.000425541	0.000581226	0.003451032	0.000150496	0.00065388	0.003134471
F	0.000513763	0.00032694	0.000394404	8.30323E-05	0.00064869	4.15162E-05	0.000705775	0.000207581	0.000311371	0.003622286	0.012641674	0.000358077	0.000710964	-	0.000186823	0.002983975	0.000799186	0.000835513	0.007166729	0.002075809
P	0.003663802	0.00053971	0.000705775	0.000622743	0.000254287	0.001219538	0.000908166	0.000238718	0.000368456	0.000934114	0.002195168	0.001499772	0.00021796	0.00021796	-	0.007358741	0.001401171	8.82219E-05	0.000295803	0.000799186
S	0.018116619	0.002366422	0.008375887	0.002158841	0.002075809	0.001489393	0.002776394	0.005708473	0.000736912	0.001722921	0.005132437	0.004302113	0.000202391	0.002423506	0.005101299	-	0.011463653	0.000129738	0.001577614	0.001105368
T	0.009984639	0.001930502	0.005609873	0.00098082	0.000513763	0.000913356	0.001811143	0.000773239	7.78428E-05	0.008246149	0.001577614	0.004546021	0.001401171	0.000742102	0.001110558	0.013134678	-	5.18952E-05	0.00021796	0.004955993
W	0.00010898	0.00140636	0.000160875	6.22743E-05	0.000389214	0.000197202	0.000306182	0.000674638	0.000166065	0.000337319	0.001587994	5.18952E-05	0.000186823	0.002340474	0.000207581	0.000446299	0.000155686	-	0.001048283	6.74638E-05
Y	0.000425541	0.001977208	0.003041059	0.001032715	0.001307759	0.001473824	0.000700585	8.82219E-05	0.004784739	0.000716154	0.001904554	0.001235106	0.000467057	0.01182173	0.000415162	0.003201935	0.000389214	0.000617553	-	0.000773239
V	0.007197866	0.000570847	0.000337319	0.000269855	0.000524142	0.000207581	0.00097563	0.000752481	5.70847E-05	0.037878316	0.008656122	0.001235106	0.001043094	0.001593183	0.000524142	0.001043094	0.004073774	1.55686E-05	0.000358077	-

From\To	A	R	N	D	C	Q	E	G	H	I	L	K	M	F	P	S	T	W	Y	V
A	-	0.00064869	0.000934114	0.000643501	0.000596795	0.000596795	0.002433885	0.005506082	0.000160875	0.001183211	0.001972018	0.001178021	0.000404783	0.000342508	0.002086188	0.014899116	0.007166729	2.59476E-05	0.000171254	0.00628451
R	0.000788807	-	0.001458255	0.000166065	0.000731723	0.006564744	0.000778428	0.002018724	0.00173849	0.00108461	0.002029103	0.022133308	0.000269855	0.000264666	0.000373646	0.002371611	0.001691784	0.000409972	0.00097563	0.000607174
N	0.001722921	0.002210736	-	0.016129032	0.000482625	0.002895753	0.005163574	0.005397102	0.003404326	0.001338897	0.001136505	0.011987794	0.000134928	0.000487815	0.000742102	0.012714327	0.007436584	6.74638E-05	0.0022782	0.0005449
D	0.001312949	0.000275045	0.017826006	-	1.0379E-05	0.001525719	0.017950554	0.00357558	0.000809565	8.82219E-05	0.00010379	0.002075809	9.86009E-05	0.000114169	0.000726533	0.003627475	0.001437497	3.11371E-05	0.000851081	0.000487815
C	0.005007888	0.005023457	0.002174409	4.15162E-05	-	4.15162E-05	5.70847E-05	0.002511728	0.001074231	0.002241873	0.003954415	0.000259476	0.000347698	0.003627475	0.001219538	0.014229667	0.003098144	0.000778428	0.004431851	0.003845435
Q	0.001183211	0.010685224	0.003113713	0.001484203	1.0379E-05	-	0.015184539	0.001110558	0.003077386	0.000736912	0.002854237	0.01637294	0.00043592	5.18952E-05	0.001375223	0.002433885	0.00130257	9.34114E-05	0.001183211	0.000358077
E	0.003720887	0.00097563	0.004276166	0.01344086	1.0379E-05	0.011702371	-	0.00313966	0.000399593	0.001183211	0.000825134	0.012989372	0.000243908	0.000721343	0.000793997	0.003492548	0.001987587	0.00010898	0.00043073	0.00130257
G	0.004971561	0.001494582	0.002636277	0.001582804	0.000269855	0.000503384	0.001852659	-	4.67057E-05	0.00032175	0.000202391	0.001312949	4.15162E-05	0.000124549	0.000124549	0.004234649	0.000498194	0.000145307	3.11371E-05	0.000596795
H	0.000498194	0.004385146	0.005661768	0.001219538	0.000394404	0.00476917	0.000799186	0.000160875	-	0.000228339	0.000664259	0.001536098	2.07581E-05	0.000638311	0.00064869	0.001868228	0.000181633	0.000119359	0.00596795	0.000160875
I	0.001110558	0.000835513	0.000679827	3.63266E-05	0.000249097	0.000347698	0.000726533	0.000332129	6.74638E-05	-	0.017364138	0.001712542	0.003840246	0.00227301	0.000498194	0.001333707	0.005557977	7.26533E-05	0.000269855	0.031038527
L	0.001473824	0.001245485	0.000461867	3.63266E-05	0.000352887	0.001074231	0.000404783	0.000166065	0.000160875	0.013840453	-	0.00108461	0.002937269	0.006326026	0.000934114	0.003160418	0.000845892	0.000285424	0.000576037	0.005651389
K	0.001774816	0.027270935	0.009745921	0.001525719	4.67057E-05	0.012397766	0.012761033	0.002184788	0.000747291	0.002740067	0.002179599	-	0.000420351	0.000363266	0.001287001	0.00531407	0.004904098	1.55686E-05	0.000752481	0.001619131
M	0.001385602	0.000768049	0.000249097	0.000171254	0.000140117	0.00075767	0.000555279	0.000171254	2.07581E-05	0.014048034	0.013477187	0.000960061	-	0.001639889	0.000425541	0.000581226	0.003451032	0.000150496	0.00065388	0.003134471
F	0.000513763	0.00032694	0.000394404	8.30323E-05	0.00064869	4.15162E-05	0.000705775	0.000207581	0.000311371	0.003622286	0.012641674	0.000358077	0.000710964	-	0.000186823	0.002983975	0.000799186	0.000835513	0.007166729	0.002075809
P	0.003663802	0.00053971	0.000705775	0.000622743	0.000254287	0.001219538	0.000908166	0.000238718	0.000368456	0.000934114	0.002195168	0.001499772	0.00021796	0.00021796	-	0.007358741	0.001401171	8.82219E-05	0.000295803	0.000799186
S	0.018116619	0.002366422	0.008375887	0.002158841	0.002075809	0.001489393	0.002776394	0.005708473	0.000736912	0.001722921	0.005132437	0.004302113	0.000202391	0.002423506	0.005101299	-	0.011463653	0.000129738	0.001577614	0.001105368
T	0.009984639	0.001930502	0.005609873	0.00098082	0.000513763	0.000913356	0.001811143	0.000773239	7.78428E-05	0.008246149	0.001577614	0.004546021	0.001401171	0.000742102	0.001110558	0.013134678	-	5.18952E-05	0.00021796	0.004955993
W	0.00010898	0.00140636	0.000160875	6.22743E-05	0.000389214	0.000197202	0.000306182	0.000674638	0.000166065	0.000337319	0.001587994	5.18952E-05	0.000186823	0.002340474	0.000207581	0.000446299	0.000155686	-	0.001048283	6.74638E-05
Y	0.000425541	0.001977208	0.003041059	0.001032715	0.001307759	0.001473824	0.000700585	8.82219E-05	0.004784739	0.000716154	0.001904554	0.001235106	0.000467057	0.01182173	0.000415162	0.003201935	0.000389214	0.000617553	-	0.000773239
V	0.007197866	0.000570847	0.000337319	0.000269855	0.000524142	0.000207581	0.00097563	0.000752481	5.70847E-05	0.037878316	0.008656122	0.001235106	0.001043094	0.001593183	0.000524142	0.001043094	0.004073774	1.55686E-05	0.000358077	-

From\To	A	R	N	D	C	Q	E	G	H	I	L	K	M	F	P	S	T	W	Y	V
A	-	0.000407695	0.001364095	0.000783644	0.00255214	0.000726599	0.00259295	0.003445	0.000200863	0.000741269	0.001841017	0.001584897	0.000456152	0.000262902	0.002001248	0.011513324	0.008353215	3.25474E-05	0.000195691	0.005704682
R	0.00053122	-	0.002129503	0.000202231	0.003129146	0.007992588	0.000829302	0.001263058	0.002170613	0.000679496	0.001894309	0.029777906	0.000304101	0.000203151	0.000358432	0.001832668	0.001971867	0.00051425	0.001114848	0.000551154
N	0.001160297	0.001389426	-	0.019641663	0.002063905	0.003525584	0.005501034	0.003376814	0.004250514	0.000838804	0.001061007	0.016128245	0.000152051	0.000374436	0.000711887	0.009825024	0.008667746	8.46233E-05	0.002603289	0.000494626
D	0.000884202	0.000172863	0.026031475	-	4.4385E-05	0.001857566	0.019123695	0.00223714	0.001010793	5.527E-05	9.68956E-05	0.00279277	0.000111114	8.76339E-05	0.000696952	0.002803139	0.001675482	3.90569E-05	0.000972527	0.000442808
C	0.003372551	0.003157193	0.003175309	5.05577E-05	-	5.0546E-05	6.08155E-05	0.001571518	0.001341245	0.001404509	0.003691723	0.000349096	0.000391823	0.002784367	0.001169884	0.010996006	0.003611057	0.000976423	0.005064256	0.003490643
Q	0.000796831	0.006715557	0.004546983	0.001807437	4.4385E-05	-	0.01617691	0.000694845	0.003842309	0.000461667	0.00266463	0.02202797	0.00049124	3.98336E-05	0.001319231	0.00188079	0.001518216	0.000117171	0.00135205	0.00032504
E	0.002505823	0.000613174	0.006244523	0.016368053	4.4385E-05	0.014247658	-	0.001964397	0.000498917	0.000741269	0.00077032	0.017475756	0.000274861	0.000553687	0.000761669	0.002698874	0.002316641	0.000136699	0.000492194	0.001182391
G	0.003348087	0.00093933	0.003849779	0.001927512	0.001154011	0.00061287	0.001973738	-	5.8315E-05	0.000201573	0.000188946	0.001766427	4.67848E-05	9.56006E-05	0.000119477	0.003272334	0.000580672	0.000182266	3.55803E-05	0.000541733
H	0.000335508	0.00275602	0.00826793	0.001485132	0.001686632	0.005806473	0.000851416	0.000100655	-	0.000143052	0.000620132	0.00206665	2.33924E-05	0.000489953	0.000622279	0.001443677	0.000211703	0.000149718	0.006819549	0.000146032
I	0.000747902	0.000525112	0.000992758	4.4238E-05	0.001065241	0.000423323	0.000774015	0.000207804	8.42327E-05	-	0.016210638	0.002304035	0.004327593	0.001744711	0.00047791	0.001030625	0.006478127	9.11328E-05	0.000308362	0.028174815
L	0.000992543	0.000782775	0.000674469	4.4238E-05	0.001509092	0.001307878	0.000431237	0.000103902	0.000200863	0.008670893	-	0.001459222	0.003310024	0.004855713	0.000896081	0.00244222	0.000985933	0.000358022	0.000658235	0.005129974
K	0.001195246	0.017139511	0.014232055	0.001857995	0.000199733	0.015094303	0.013595018	0.00136696	0.00093304	0.001716622	0.002034808	-	0.000473696	0.000278835	0.001234601	0.004106459	0.005715994	1.95285E-05	0.000859856	0.001469745
M	0.000933131	0.000482711	0.000363759	0.00020855	0.000599198	0.000922465	0.000591568	0.000107149	2.59178E-05	0.00880094	0.012581897	0.001291656	-	0.001258741	0.000408215	0.000449144	0.004022366	0.000188775	0.000747185	0.002845275
F	0.000345992	0.000205478	0.000575951	0.000101115	0.002774065	5.0546E-05	0.0007519	0.000129877	0.000388767	0.002269323	0.011801887	0.000481753	0.00080119	-	0.000179216	0.002305873	0.000931495	0.001048028	0.008189389	0.001884288
P	0.002467379	0.000339203	0.001030649	0.000758365	0.001087434	0.001484789	0.000967519	0.000149359	0.00046004	0.000585212	0.002049342	0.002017776	0.00024562	0.000167301	-	0.005686483	0.001633141	0.000110661	0.000338012	0.000725451
S	0.012200596	0.001487273	0.012231383	0.002629	0.008877009	0.001813338	0.002957842	0.003571631	0.000920081	0.001079391	0.004791489	0.005788015	0.000228076	0.001860228	0.004893598	-	0.013361515	0.000162737	0.001802733	0.001003383
T	0.006724132	0.001213301	0.008192147	0.001194425	0.00219706	0.001112012	0.001929508	0.000483794	9.71916E-05	0.005166122	0.001472813	0.006116165	0.001578987	0.00056962	0.001065341	0.010149851	-	6.50949E-05	0.000249062	0.004498737
W	7.33923E-05	0.000883883	0.000234927	7.58365E-05	0.001664439	0.000240094	0.000326192	0.000422102	0.000207342	0.000211327	0.001482503	6.98192E-05	0.000210532	0.001796494	0.000199129	0.000344878	0.00018146	-	0.001197869	6.12394E-05
Y	0.000286579	0.001242655	0.004440886	0.001257623	0.005592516	0.001794384	0.000746371	5.51979E-05	0.005974046	0.000448663	0.001778035	0.001661698	0.000526329	0.009074089	0.000398258	0.002474302	0.00045365	0.000774629	-	0.000701897
V	0.004847386	0.000358772	0.00049259	0.000328625	0.002241445	0.00025273	0.001039391	0.000470806	7.12739E-05	0.023730351	0.008081095	0.001661698	0.001175468	0.001222891	0.000502801	0.000806053	0.004748207	1.95285E-05	0.000409173	-

From\To	A	R	N	D	C	Q	E	G	H	I
A	-	0.000407695	0.001364095	0.000783644	0.00255214	0.000726599	0.00259295	0.003445	0.000200863	0.000741269
R	0.00053122	-	0.002129503	0.000202231	0.003129146	0.007992588	0.000829302	0.001263058	0.002170613	0.000679496
N	0.001160297	0.001389426	-	0.019641663	0.002063905	0.003525584	0.005501034	0.003376814	0.004250514	0.000838804
D	0.000884202	0.000172863	0.026031475	-	4.4385E-05	0.001857566	0.019123695	0.00223714	0.001010793	5.527E-05
C	0.003372551	0.003157193	0.003175309	5.05577E-05	-	5.0546E-05	6.08155E-05	0.001571518	0.001341245	0.001404509
Q	0.000796831	0.006715557	0.004546983	0.001807437	4.4385E-05	-	0.01617691	0.000694845	0.003842309	0.000461667
E	0.002505823	0.000613174	0.006244523	0.016368053	4.4385E-05	0.014247658	-	0.001964397	0.000498917	0.000741269
G	0.003348087	0.00093933	0.003849779	0.001927512	0.001154011	0.00061287	0.001973738	-	5.8315E-05	0.000201573
H	0.000335508	0.00275602	0.00826793	0.001485132	0.001686632	0.005806473	0.000851416	0.000100655	-	0.000143052
I	0.000747902	0.000525112	0.000992758	4.4238E-05	0.001065241	0.000423323	0.000774015	0.000207804	8.42327E-05	-
L	0.000992543	0.000782775	0.000674469	4.4238E-05	0.001509092	0.001307878	0.000431237	0.000103902	0.000200863	0.008670893
K	0.001195246	0.017139511	0.014232055	0.001857995	0.000199733	0.015094303	0.013595018	0.00136696	0.00093304	0.001716622
M	0.000933131	0.000482711	0.000363759	0.00020855	0.000599198	0.000922465	0.000591568	0.000107149	2.59178E-05	0.00880094
F	0.000345992	0.000205478	0.000575951	0.000101115	0.002774065	5.0546E-05	0.0007519	0.000129877	0.000388767	0.002269323
P	0.002467379	0.000339203	0.001030649	0.000758365	0.001087434	0.001484789	0.000967519	0.000149359	0.00046004	0.000585212
S	0.012200596	0.001487273	0.012231383	0.002629	0.008877009	0.001813338	0.002957842	0.003571631	0.000920081	0.001079391
T	0.006724132	0.001213301	0.008192147	0.001194425	0.00219706	0.001112012	0.001929508	0.000483794	9.71916E-05	0.005166122
W	7.33923E-05	0.000883883	0.000234927	7.58365E-05	0.001664439	0.000240094	0.000326192	0.000422102	0.000207342	0.000211327
Y	0.000286579	0.001242655	0.004440886	0.001257623	0.005592516	0.001794384	0.000746371	5.51979E-05	0.005974046	0.000448663
V	0.004847386	0.000358772	0.00049259	0.000328625	0.002241445	0.00025273	0.001039391	0.000470806	7.12739E-05	0.023730351

From\To	L	K	M	F	P	S	T	W	Y	V
A	0.001841017	0.001584897	0.000456152	0.000262902	0.002001248	0.011513324	0.008353215	3.25474E-05	0.000195691	0.005704682
R	0.001894309	0.029777906	0.000304101	0.000203151	0.000358432	0.001832668	0.001971867	0.00051425	0.001114848	0.000551154
N	0.001061007	0.016128245	0.000152051	0.000374436	0.000711887	0.009825024	0.008667746	8.46233E-05	0.002603289	0.000494626
D	9.68956E-05	0.00279277	0.000111114	8.76339E-05	0.000696952	0.002803139	0.001675482	3.90569E-05	0.000972527	0.000442808
C	0.003691723	0.000349096	0.000391823	0.002784367	0.001169884	0.010996006	0.003611057	0.000976423	0.005064256	0.003490643
Q	0.00266463	0.02202797	0.00049124	3.98336E-05	0.001319231	0.00188079	0.001518216	0.000117171	0.00135205	0.00032504
E	0.00077032	0.017475756	0.000274861	0.000553687	0.000761669	0.002698874	0.002316641	0.000136699	0.000492194	0.001182391
G	0.000188946	0.001766427	4.67848E-05	9.56006E-05	0.000119477	0.003272334	0.000580672	0.000182266	3.55803E-05	0.000541733
H	0.000620132	0.00206665	2.33924E-05	0.000489953	0.000622279	0.001443677	0.000211703	0.000149718	0.006819549	0.000146032
I	0.016210638	0.002304035	0.004327593	0.001744711	0.00047791	0.001030625	0.006478127	9.11328E-05	0.000308362	0.028174815
L	-	0.001459222	0.003310024	0.004855713	0.000896081	0.00244222	0.000985933	0.000358022	0.000658235	0.005129974
K	0.002034808	-	0.000473696	0.000278835	0.001234601	0.004106459	0.005715994	1.95285E-05	0.000859856	0.001469745
M	0.012581897	0.001291656	-	0.001258741	0.000408215	0.000449144	0.004022366	0.000188775	0.000747185	0.002845275
F	0.011801887	0.000481753	0.00080119	-	0.000179216	0.002305873	0.000931495	0.001048028	0.008189389	0.001884288
P	0.002049342	0.002017776	0.00024562	0.000167301	-	0.005686483	0.001633141	0.000110661	0.000338012	0.000725451
S	0.004791489	0.005788015	0.000228076	0.001860228	0.004893598	-	0.013361515	0.000162737	0.001802733	0.001003383
T	0.001472813	0.006116165	0.001578987	0.00056962	0.001065341	0.010149851	-	6.50949E-05	0.000249062	0.004498737
W	0.001482503	6.98192E-05	0.000210532	0.001796494	0.000199129	0.000344878	0.00018146	-	0.001197869	6.12394E-05
Y	0.001778035	0.001661698	0.000526329	0.009074089	0.000398258	0.002474302	0.00045365	0.000774629	-	0.000701897
V	0.008081095	0.001661698	0.001175468	0.001222891	0.000502801	0.000806053	0.004748207	1.95285E-05	0.000409173	-

From\To	A	R	N	D	C	Q	E	G	H	I	L	K	M	F	P	S	T	W	Y	V
A	-	0.001207701	0.000590135	0.001064639	0.001635379	0.002008068	0.003775711	0.006016155	0.00040742	0.000472971	0.001989313	0.001760201	0.001310167	0.00054504	0.00263396	0.014691894	0.005790007	0.000110741	0.000379807	0.008947381
R	0.001706943	-	0.001602891	0.00033397	0.000351211	0.00581349	0.001323241	0.00113621	0.00275498	0.000400874	0.001518882	0.02075447	0.000564302	0.000113266	0.000743751	0.002667099	0.001566879	0.000363754	0.000545428	0.000600106
N	0.001111551	0.002136107	-	0.013676704	0.000347411	0.003510884	0.001969436	0.004186316	0.005119448	0.00060452	0.000344321	0.007037541	0.00043244	0.000192332	0.000361856	0.012457818	0.005414321	2.78058E-05	0.001061619	0.000293888
D	0.001586684	0.000352157	0.01082159	-	4.11006E-05	0.001083618	0.019064497	0.002460362	0.001052575	3.37453E-05	7.58616E-05	0.000928328	2.97786E-05	3.74158E-05	0.000882249	0.003854726	0.00115248	1.83162E-05	0.000234357	0.000133329
C	0.009994813	0.001518675	0.001127254	0.000168545	-	0.000175586	1.27209E-05	0.001657657	0.000727224	0.001012128	0.002989009	4.35229E-05	0.001041667	0.002374473	0.000168601	0.008654047	0.003094533	0.000410645	0.002021733	0.006880462
Q	0.003894569	0.007977346	0.00361509	0.001410163	5.57206E-05	-	0.015009813	0.000780277	0.00546489	0.000229979	0.00293089	0.010611025	0.001949535	7.70293E-05	0.00139631	0.003803609	0.002923109	0.000144739	0.000446375	0.000738625
E	0.004170234	0.001034049	0.001154849	0.014128596	2.29891E-06	0.008547831	-	0.001015817	0.000481242	0.000139732	0.000350591	0.00592896	0.000202504	4.04127E-05	0.00093806	0.001901988	0.001636045	4.77066E-05	0.000208216	0.000860488
G	0.008296097	0.001108547	0.003064844	0.00227649	0.000374019	0.000554782	0.00126826	-	0.000353635	2.74792E-05	0.000222719	0.000973199	0.000162645	0.000192463	0.000440527	0.00540782	0.000351368	0.000164527	9.48463E-05	0.000269352
H	0.001440979	0.006894042	0.009613021	0.00249793	0.00042085	0.009965877	0.001541052	0.000907018	-	0.000343709	0.001843286	0.002287574	0.000515742	0.001465477	0.001138107	0.003076918	0.001581154	0.000365866	0.00920524	0.000417939
I	0.000601636	0.000360785	0.000408256	2.88021E-05	0.000210659	0.000150837	0.000160929	2.53483E-05	0.000123616	-	0.020857741	0.000521871	0.004981287	0.002390534	0.000175085	0.000199236	0.002797548	6.84237E-05	0.000403335	0.037396578
L	0.001587459	0.000857559	0.000145876	4.06194E-05	0.000390275	0.001205919	0.000253302	0.000128885	0.000415889	0.013084795	-	0.000451108	0.007357641	0.005570025	0.000557053	0.000566541	0.000819818	0.000379702	0.00051977	0.005979547
K	0.002154366	0.017972535	0.004572985	0.000762378	8.71603E-06	0.00669628	0.006570132	0.000863782	0.000791621	0.000502136	0.000691892	-	0.000765333	5.13844E-05	0.000873003	0.002326877	0.003076626	3.05817E-05	0.000228849	0.000650376
M	0.004513515	0.001375436	0.000790925	6.88342E-05	0.000587166	0.003462886	0.000631627	0.000406324	0.000502349	0.01349056	0.031763426	0.002154177	-	0.003864577	0.000223324	0.001078338	0.005467599	0.000426606	0.000834874	0.006667748
F	0.001018726	0.000149785	0.000190854	4.69241E-05	0.000726173	7.42342E-05	6.83889E-05	0.000260868	0.000774449	0.003512562	0.013046278	7.84698E-05	0.002096731	-	0.00021128	0.001124519	0.000446533	0.001505689	0.013536657	0.002299057
P	0.004728808	0.000944736	0.000344906	0.001062786	4.95275E-05	0.001292538	0.001524794	0.000573536	0.000577711	0.000247111	0.001253256	0.001280563	0.000116383	0.000202942	-	0.004158861	0.001546531	5.82949E-05	0.000155443	0.001041226
S	0.018981801	0.002438031	0.00854522	0.003341682	0.001829459	0.002533812	0.002224875	0.005066722	0.001123985	0.000202361	0.000917258	0.002456268	0.000404414	0.000777316	0.002992896	-	0.017515552	0.000152499	0.000694789	0.000345443
T	0.008591077	0.001644918	0.004265148	0.001147398	0.00075129	0.002236313	0.00219787	0.000378073	0.000663327	0.003263219	0.001524357	0.003729804	0.002354925	0.000354481	0.001278158	0.020115586	-	8.62956E-05	0.000426436	0.007684177
W	0.000725662	0.001686454	9.67348E-05	8.05328E-05	0.000440288	0.000489026	0.000283037	0.000781826	0.00067785	0.000352479	0.003117953	0.000163731	0.000811457	0.00527877	0.000212772	0.000773453	0.000381107	-	0.005467142	0.000665504
Y	0.000879221	0.000893333	0.001304746	0.00036402	0.000765778	0.000532788	0.000436404	0.000159221	0.006024979	0.000734009	0.001507812	0.000432841	0.000561007	0.016765559	0.00020043	0.001244884	0.000665305	0.001931387	-	0.000875515
V	0.010230865	0.000485495	0.00017841	0.000102295	0.001287294	0.000435471	0.00089084	0.000223348	0.000135118	0.033616196	0.008568116	0.000607608	0.002213133	0.001406492	0.000663161	0.000305727	0.005921685	0.000116129	0.000432459	-

From\To	A	R	N	D	C	Q	E	G	H	I	L	K	M	F	P	S	T	W	Y	V
A	-	0.000476734	0.000851223	0.000420681	0.001227319	4.6565E-05	0.000269275	0.003333424	0.000224489	0.002568257	0.001266278	0.000298715	0.001854202	0.000132214	0.001183906	0.009821999	0.01603169	2.27313E-05	0.000119419	0.006160282
R	0.000621887	-	0.000419617	4.52345E-05	0.002119561	0.005415505	5.2799E-05	0.000635188	0.002675414	5.05562E-05	0.000774887	0.005051002	2.4822E-05	9.72162E-05	0.000513302	0.000153995	6.9703E-05	0.000263683	3.51233E-05	0.000239933
N	0.000723836	0.000273536	-	0.01891256	0.001207838	0.004251381	0.001752926	0.001475106	0.008029316	0.000722954	0.000755987	0.021751897	0.000853876	0.000311092	0.001597859	0.012521725	0.007952479	0.000127295	0.003536915	5.99833E-05
D	0.000474062	3.90766E-05	0.025063115	-	3.89625E-05	0.00135504	0.016214563	0.001569597	0.001845113	0.000116279	9.44984E-05	8.14678E-05	2.4822E-05	0.000101105	0.000293907	0.001746882	0.000931486	0.000238679	0.000393381	5.99833E-05
C	0.001605693	0.002125765	0.001858303	4.52345E-05	-	0.001843973	5.2799E-05	0.000850417	0.002291015	0.001668356	0.001275728	6.78898E-05	8.19125E-05	0.001450466	0.000683022	0.007016401	0.006000795	0.000402344	0.004710033	5.99833E-05
Q	5.09744E-05	0.004544604	0.005473003	0.001316325	0.001542916	-	0.00871183	0.000188982	0.009425452	0.000222447	0.001975016	0.016633005	0.000618067	0.000392754	0.00299288	0.001371519	0.003168318	2.27313E-05	0.000716515	0.000599833
E	0.000259969	3.90766E-05	0.001990183	0.013891526	3.89625E-05	0.007683219	-	0.000782174	0.000796474	8.59456E-05	9.44984E-05	0.011215398	2.4822E-05	5.44411E-05	0.000281488	0.001385956	0.000494258	2.27313E-05	0.000242351	0.000665814
G	0.003236873	0.000472826	0.001684462	0.001352513	0.000631193	0.000167634	0.000786705	-	3.07519E-05	0.000156724	0.000122848	0.000814678	2.4822E-05	3.88865E-05	4.13953E-05	0.003190586	0.000373862	0.000129568	5.97096E-05	7.79783E-05
H	0.000372113	0.00339966	0.015651708	0.002714072	0.002902708	0.014272161	0.001367493	5.24949E-05	-	0.000328616	0.00056699	0.004562196	0.000156378	0.000983828	0.001328789	0.001963437	0.001495446	8.41058E-05	0.012387983	5.99833E-05
I	0.002589499	3.90766E-05	0.000857217	0.000104039	0.001285763	0.000204886	8.97582E-05	0.000162734	0.000199887	-	0.016367117	0.000699265	0.006766471	0.001734338	0.000451209	0.001207899	0.012286738	2.27313E-05	0.000463627	0.038611235
L	0.000683057	0.000320428	0.000479562	4.52345E-05	0.000525994	0.000973208	5.2799E-05	6.82433E-05	0.000184511	0.008756342	-	0.000529541	0.007022137	0.004421395	0.000873441	0.001862378	0.004213863	0.000388705	0.00081486	0.002891194
K	0.000224287	0.002907296	0.019206462	5.42814E-05	3.89625E-05	0.011408416	0.008722389	0.000629938	0.002066526	0.000520729	0.000737087	-	0.001193937	0.000132214	0.001092836	0.002680477	0.004549705	0.000286414	0.000944816	5.99833E-05
M	0.003807786	3.90766E-05	0.002062117	4.52345E-05	0.000128576	0.001159468	5.2799E-05	5.24949E-05	0.000193737	0.013781633	0.026733588	0.0032655	-	0.001858775	0.000409813	0.002815223	0.017615849	0.000259137	0.000737589	0.012236588
F	0.000173313	9.76914E-05	0.000479562	0.00011761	0.001453302	0.000470306	7.39186E-05	5.24949E-05	0.000778023	0.002254809	0.010744464	0.000230825	0.00118649	-	0.000376697	0.001626573	0.001127921	9.31983E-05	0.008605205	0.000197945
P	0.001457867	0.000484549	0.002313887	0.000321165	0.000642882	0.003366647	0.000359033	5.24949E-05	0.000987135	0.000551063	0.001993916	0.001792291	0.000245738	0.000353867	-	0.004302238	0.004277229	5.00089E-05	0.000298548	0.000257928
S	0.010403871	0.000125045	0.015597758	0.001642014	0.005680736	0.001327101	0.00152061	0.00348041	0.001254677	0.001268962	0.003657087	0.003781463	0.001452086	0.001314364	0.00370074	-	0.019916048	0.000461445	0.001201216	5.99833E-05
T	0.012896518	4.29842E-05	0.007523131	0.000664948	0.003689751	0.002328248	0.000411832	0.00030972	0.000725744	0.009802856	0.006284141	0.004874489	0.006900509	0.00069218	0.002794183	0.015125205	-	0.000120476	0.000716515	0.006460199
W	5.09744E-05	0.000453288	0.000335693	0.000474963	0.000689637	4.6565E-05	5.2799E-05	0.000299221	0.000113782	5.05562E-05	0.001615922	0.000855412	0.000282971	0.000159435	9.10697E-05	0.000976906	0.000335842	-	0.000484701	0.000167953
Y	0.000173313	3.90766E-05	0.006036488	0.000506627	0.005224874	0.000949925	0.000364313	8.92413E-05	0.010846189	0.000667342	0.002192362	0.001826236	0.000521262	0.009527192	0.00035186	0.001645822	0.001292674	0.000313692	-	5.99833E-05
V	0.005235069	0.000156306	5.99453E-05	4.52345E-05	3.89625E-05	0.00046565	0.000586069	6.82433E-05	3.07519E-05	0.032543056	0.004554821	6.78898E-05	0.005063683	0.000128325	0.000178	4.81235E-05	0.006824557	6.36477E-05	3.51233E-05	-

From\To	A	R	N	D	C	Q	E	G	H	I	L	K	M	F	P	S	T	W	Y	V
A	-	0.000476734	0.000851223	0.000420681	0.001227319	4.6565E-05	0.000269275	0.003333424	0.000224489	0.002568257	0.001266278	0.000298715	0.001854202	0.000132214	0.001183906	0.009821999	0.01603169	2.27313E-05	0.000119419	0.006160282
R	0.000621887	-	0.000419617	4.52345E-05	0.002119561	0.005415505	5.2799E-05	0.000635188	0.002675414	5.05562E-05	0.000774887	0.005051002	2.4822E-05	9.72162E-05	0.000513302	0.000153995	6.9703E-05	0.000263683	3.51233E-05	0.000239933
N	0.000723836	0.000273536	-	0.01891256	0.001207838	0.004251381	0.001752926	0.001475106	0.008029316	0.000722954	0.000755987	0.021751897	0.000853876	0.000311092	0.001597859	0.012521725	0.007952479	0.000127295	0.003536915	5.99833E-05
D	0.000474062	3.90766E-05	0.025063115	-	3.89625E-05	0.00135504	0.016214563	0.001569597	0.001845113	0.000116279	9.44984E-05	8.14678E-05	2.4822E-05	0.000101105	0.000293907	0.001746882	0.000931486	0.000238679	0.000393381	5.99833E-05
C	0.001605693	0.002125765	0.001858303	4.52345E-05	-	0.001843973	5.2799E-05	0.000850417	0.002291015	0.001668356	0.001275728	6.78898E-05	8.19125E-05	0.001450466	0.000683022	0.007016401	0.006000795	0.000402344	0.004710033	5.99833E-05
Q	5.09744E-05	0.004544604	0.005473003	0.001316325	0.001542916	-	0.00871183	0.000188982	0.009425452	0.000222447	0.001975016	0.016633005	0.000618067	0.000392754	0.00299288	0.001371519	0.003168318	2.27313E-05	0.000716515	0.000599833
E	0.000259969	3.90766E-05	0.001990183	0.013891526	3.89625E-05	0.007683219	-	0.000782174	0.000796474	8.59456E-05	9.44984E-05	0.011215398	2.4822E-05	5.44411E-05	0.000281488	0.001385956	0.000494258	2.27313E-05	0.000242351	0.000665814
G	0.003236873	0.000472826	0.001684462	0.001352513	0.000631193	0.000167634	0.000786705	-	3.07519E-05	0.000156724	0.000122848	0.000814678	2.4822E-05	3.88865E-05	4.13953E-05	0.003190586	0.000373862	0.000129568	5.97096E-05	7.79783E-05
H	0.000372113	0.00339966	0.015651708	0.002714072	0.002902708	0.014272161	0.001367493	5.24949E-05	-	0.000328616	0.00056699	0.004562196	0.000156378	0.000983828	0.001328789	0.001963437	0.001495446	8.41058E-05	0.012387983	5.99833E-05
I	0.002589499	3.90766E-05	0.000857217	0.000104039	0.001285763	0.000204886	8.97582E-05	0.000162734	0.000199887	-	0.016367117	0.000699265	0.006766471	0.001734338	0.000451209	0.001207899	0.012286738	2.27313E-05	0.000463627	0.038611235
L	0.000683057	0.000320428	0.000479562	4.52345E-05	0.000525994	0.000973208	5.2799E-05	6.82433E-05	0.000184511	0.008756342	-	0.000529541	0.007022137	0.004421395	0.000873441	0.001862378	0.004213863	0.000388705	0.00081486	0.002891194
K	0.000224287	0.002907296	0.019206462	5.42814E-05	3.89625E-05	0.011408416	0.008722389	0.000629938	0.002066526	0.000520729	0.000737087	-	0.001193937	0.000132214	0.001092836	0.002680477	0.004549705	0.000286414	0.000944816	5.99833E-05
M	0.003807786	3.90766E-05	0.002062117	4.52345E-05	0.000128576	0.001159468	5.2799E-05	5.24949E-05	0.000193737	0.013781633	0.026733588	0.0032655	-	0.001858775	0.000409813	0.002815223	0.017615849	0.000259137	0.000737589	0.012236588
F	0.000173313	9.76914E-05	0.000479562	0.00011761	0.001453302	0.000470306	7.39186E-05	5.24949E-05	0.000778023	0.002254809	0.010744464	0.000230825	0.00118649	-	0.000376697	0.001626573	0.001127921	9.31983E-05	0.008605205	0.000197945
P	0.001457867	0.000484549	0.002313887	0.000321165	0.000642882	0.003366647	0.000359033	5.24949E-05	0.000987135	0.000551063	0.001993916	0.001792291	0.000245738	0.000353867	-	0.004302238	0.004277229	5.00089E-05	0.000298548	0.000257928
S	0.010403871	0.000125045	0.015597758	0.001642014	0.005680736	0.001327101	0.00152061	0.00348041	0.001254677	0.001268962	0.003657087	0.003781463	0.001452086	0.001314364	0.00370074	-	0.019916048	0.000461445	0.001201216	5.99833E-05
T	0.012896518	4.29842E-05	0.007523131	0.000664948	0.003689751	0.002328248	0.000411832	0.00030972	0.000725744	0.009802856	0.006284141	0.004874489	0.006900509	0.00069218	0.002794183	0.015125205	-	0.000120476	0.000716515	0.006460199
W	5.09744E-05	0.000453288	0.000335693	0.000474963	0.000689637	4.6565E-05	5.2799E-05	0.000299221	0.000113782	5.05562E-05	0.001615922	0.000855412	0.000282971	0.000159435	9.10697E-05	0.000976906	0.000335842	-	0.000484701	0.000167953
Y	0.000173313	3.90766E-05	0.006036488	0.000506627	0.005224874	0.000949925	0.000364313	8.92413E-05	0.010846189	0.000667342	0.002192362	0.001826236	0.000521262	0.009527192	0.00035186	0.001645822	0.001292674	0.000313692	-	5.99833E-05
V	0.005235069	0.000156306	5.99453E-05	4.52345E-05	3.89625E-05	0.00046565	0.000586069	6.82433E-05	3.07519E-05	0.032543056	0.004554821	6.78898E-05	0.005063683	0.000128325	0.000178	4.81235E-05	0.006824557	6.36477E-05	3.51233E-05	-

From\To	A	R	N	D	C	Q	E	G	H	I	L	K	M	F	P	S	T	W	Y	V
A	-	0.000817463	0.001017768	0.000223977	0.002656196	0.001029232	0.002609543	0.004578523	0.000434685	3.6038E-05	0.00243137	0.001514657	0.001870897	1.52326E-05	0.003505981	0.011914296	0.008517278	6.66092E-05	0.000928283	0.006472136
R	0.001165741	-	0.000698468	0.000671931	0.000556652	0.007108135	0.000322166	0.001390514	0.001304055	0.000864912	0.000972548	0.02363662	0.000453792	0.000106628	0.000867459	0.002641866	0.002112813	0.000173184	0.00079326	0.000952751
N	0.001748612	0.000841506	-	0.008600712	0.000774472	0.007590587	0.00254511	0.003188008	0.00463664	0.00126133	0.000810457	0.004902705	7.96126E-06	0.000746395	0.001192757	0.007614789	0.004885881	0.00021315	0.000472581	0.000689923
D	0.000342865	0.00072129	0.007663194	-	6.05056E-06	0.002508753	0.017461387	0.002068814	0.001318544	3.6038E-05	0.000270152	0.000797188	7.96126E-06	1.52326E-05	0.001987928	0.004221805	0.001815699	1.33218E-05	1.68779E-05	0.000197121
C	0.015051777	0.002211957	0.002554398	2.23977E-05	-	0.002251445	3.22166E-05	0.001627919	0.001796698	0.003747951	0.005943349	0.00063775	0.001241957	0.001066279	3.61441E-05	0.001942548	0.003862487	0.000732702	0.002211002	0.009691777
Q	0.001097168	0.005313507	0.004709671	0.00174702	0.000423539	-	0.011984568	0.00061047	0.005607436	0.001189254	0.002917644	0.012316553	0.00125788	1.52326E-05	0.002457801	0.005827645	0.004819855	0.000133218	0.000759505	0.001182725
E	0.002777207	0.00024043	0.001576543	0.012139547	6.05056E-06	0.011964824	-	0.002374049	0.000492643	3.6038E-05	0.001134639	0.005620174	7.96126E-06	1.52326E-05	0.001879495	0.002460561	0.002707042	0.000226471	1.68779E-05	0.001149872
G	0.004628679	0.000985764	0.001875886	0.001366259	0.000290427	0.000578943	0.002255161	-	0.000985286	3.6038E-05	0.000162091	0.001195782	0.000294567	0.000106628	0.00061445	0.003936898	0.000231089	0.000306403	0.000354435	9.85604E-05
H	0.001028595	0.002163871	0.006385995	0.00203819	0.000750269	0.012447277	0.001095364	0.002306219	-	0.001225292	0.002755553	0.003029314	0.000923507	0.00214779	0.001590342	0.004739818	0.001617623	0.000639449	0.005181509	3.28535E-05
I	3.42865E-05	0.000577032	0.000698468	2.23977E-05	0.000629258	0.001061396	3.22166E-05	3.3915E-05	0.000492643	-	0.02080172	0.001355219	0.002985474	0.000974883	0.000361441	0.000103603	0.002376915	0.000519552	0.000438825	0.034430449
L	0.001542893	0.000432774	0.000299344	0.000111988	0.000665562	0.001736829	0.000676548	0.000101745	0.000738964	0.013874625	-	0.000916766	0.004625494	0.002726627	0.000795171	0.000621615	0.000825318	0.000626127	0.001080184	0.00367959
K	0.001302887	0.014257509	0.002454617	0.000447954	9.6809E-05	0.009938523	0.004542538	0.001017449	0.001101202	0.001225292	0.0012427	-	0.001066809	0.000213256	0.001554198	0.00199435	0.003631398	7.99311E-05	1.68779E-05	0.000624216
M	0.00805733	0.001370452	1.99562E-05	2.23977E-05	0.000943887	0.005081834	3.22166E-05	0.001254854	0.001680782	0.013514245	0.031391687	0.005341159	-	0.00376244	3.61441E-05	2.59006E-05	0.004324665	0.001478725	0.001248963	0.007753422
F	3.42865E-05	0.000168301	0.000977855	2.23977E-05	0.000423539	3.21635E-05	3.22166E-05	0.000237405	0.002043019	0.002306431	0.009671449	0.000558032	0.001966432	-	0.000397586	0.000518013	0.002277877	0.002424576	0.017164803	0.00302252
P	0.003325791	0.000577032	0.000658556	0.001231873	6.05056E-06	0.002187118	0.001675262	0.000576555	0.000637538	0.00036038	0.00118867	0.001713954	7.96126E-06	0.000167558	-	0.003470686	0.002046788	0.000119897	0.00023629	0.00821337
S	0.015771794	0.002452388	0.005867133	0.003650823	0.000453792	0.007236789	0.003060575	0.005155077	0.002651578	0.000144152	0.001296731	0.003069173	7.96126E-06	0.000304651	0.004843314	-	0.022151526	0.000186506	0.000523214	0.001281286
T	0.008845919	0.001538753	0.002953523	0.001231873	0.000707916	0.004695872	0.00264176	0.000237405	0.000709985	0.002594735	0.001350761	0.004384533	0.001042926	0.001051046	0.002240936	0.017379332	-	1.33218E-05	0.000573848	0.006439282
W	0.000171433	0.000312559	0.0003193	2.23977E-05	0.000332781	0.000321635	0.000547682	0.000780045	0.000695496	0.001405481	0.002539431	0.000239156	0.0008837	0.002772324	0.000325297	0.000362609	3.30127E-05	-	0.002970507	0.000854191
Y	0.001885758	0.001130022	0.000558775	2.23977E-05	0.000792623	0.001447358	3.22166E-05	0.000712215	0.004448276	0.000936988	0.003457948	3.98594E-05	0.000589134	0.015491505	0.000506018	0.00080292	0.001122432	0.002344645	-	0.001938355
V	0.006754442	0.000697247	0.000419081	0.000134386	0.001784915	0.001157886	0.00112758	0.000101745	1.44895E-05	0.03776781	0.00605141	0.000757328	0.001878858	0.001401395	0.000903603	0.001010125	0.006470491	0.000346368	0.00995795	-

From\To	A	R	N	D	C	Q	E	G	H	I	L	K	M	F	P	S	T	W	Y	V
A	-	0.000876381	0.000875472	0.000943015	0.005116576	0.002382739	0.002892968	0.005722008	0.00058222	0.000399638	0.001970991	0.001921683	0.001472004	0.000520492	0.002571938	0.012001957	0.007152603	0.000216729	0.000405743	0.008063067
R	0.001143217	-	0.002377908	0.000295817	0.001098826	0.006898187	0.001013874	0.001080657	0.003936977	0.00033872	0.001504892	0.022658506	0.000634007	0.000108164	0.000726237	0.00217878	0.001935622	0.000711895	0.000582674	0.000540794
N	0.000744456	0.001550089	-	0.012114273	0.001086939	0.004165954	0.001508991	0.003981634	0.007315899	0.000510791	0.000341149	0.007683172	0.000485856	0.000183669	0.000353336	0.010176917	0.006688505	5.44181E-05	0.001134115	0.000264842
D	0.001062674	0.000255546	0.016053956	-	0.00012859	0.001285802	0.014607308	0.002340067	0.001504173	2.85132E-05	7.51629E-05	0.001013494	3.34569E-05	3.57306E-05	0.000861475	0.003148964	0.0014237	3.58462E-05	0.000250361	0.000120152
C	0.006693979	0.001102042	0.001672295	0.00014929	-	0.000208348	9.7468E-06	0.00157661	0.001039233	0.0008552	0.002961479	4.75157E-05	0.001170338	0.002267528	0.000164631	0.007069579	0.003822788	0.000803665	0.002159793	0.006200432
Q	0.002608369	0.005788846	0.005363028	0.001249065	0.000174332	-	0.01150059	0.000742127	0.007809549	0.000194322	0.002903896	0.011584492	0.002190349	7.35599E-05	0.001363431	0.003107207	0.003611021	0.000283267	0.000476857	0.000665623
E	0.002792995	0.000750369	0.001713232	0.01251454	7.19257E-06	0.010142709	-	0.00096615	0.000687715	0.000118067	0.000347361	0.006472889	0.000227518	3.85926E-05	0.000915971	0.001553753	0.002021065	9.33656E-05	0.000222435	0.000775442
G	0.005556272	0.000804429	0.004546732	0.002016423	0.001170185	0.000658295	0.000971747	-	0.000505359	2.32186E-05	0.000220667	0.001062481	0.000182735	0.000183794	0.000430154	0.004417703	0.000434057	0.000321993	0.000101323	0.00024273
H	0.000965089	0.005002735	0.014261029	0.002212565	0.001316704	0.011825337	0.001180762	0.000862671	-	0.000290418	0.001826309	0.002497438	0.000579449	0.001399473	0.001111308	0.002513565	0.001953256	0.000716029	0.009833848	0.000376632
I	0.000402943	0.000261808	0.000605652	2.55118E-05	0.000659083	0.00017898	0.000123304	2.4109E-05	0.000176653	-	0.020665633	0.000569748	0.005596594	0.002282865	0.000170962	0.000162758	0.003455911	0.000133911	0.000430878	0.03370049
L	0.001063193	0.000622297	0.000216409	3.5979E-05	0.001221044	0.001430922	0.000194081	0.000122583	0.000594322	0.01105603	-	0.000492493	0.008266484	0.005319154	0.000543936	0.000462813	0.001012751	0.000743106	0.000555264	0.005388559
K	0.001442876	0.013041962	0.006784078	0.000675284	2.72697E-05	0.00794569	0.005034067	0.000821549	0.001131258	0.000424281	0.00068552	-	0.00085987	4.90701E-05	0.000852446	0.001900849	0.003800666	5.98508E-05	0.000244477	0.000586096
M	0.003022906	0.000998099	0.001173347	6.09705E-05	0.001837054	0.004109	0.000483956	0.000386458	0.000717877	0.011398882	0.031470872	0.002351803	-	0.003690518	0.000218066	0.000880905	0.006754321	0.000834902	0.000891886	0.006008741
F	0.000682287	0.000108693	0.000283134	4.15634E-05	0.002271961	8.8085E-05	5.23999E-05	0.000248113	0.001106719	0.002967948	0.012926117	8.56687E-05	0.002355727	-	0.000206305	0.000918631	0.000551618	0.002946753	0.014461049	0.00207183
P	0.003167097	0.000685558	0.000511672	0.000941373	0.000154956	0.001533703	0.001168304	0.000545494	0.000825572	0.000208797	0.001241713	0.001398043	0.000130759	0.000193802	-	0.003397416	0.001910485	0.000114088	0.000166058	0.000938316
S	0.012712973	0.001769183	0.012676934	0.002959927	0.00572379	0.003006578	0.00170471	0.004818995	0.00160622	0.000170986	0.000908809	0.002681609	0.000454369	0.000742306	0.002922422	-	0.021637588	0.000298453	0.000742235	0.000311302
T	0.005753834	0.001193653	0.006327398	0.001016319	0.002350544	0.00265357	0.001684019	0.000359588	0.000947921	0.002757265	0.001510317	0.00407198	0.002645814	0.000338515	0.001248061	0.016432626	-	0.000168887	0.000455557	0.006924712
W	0.000486008	0.001223794	0.000143507	7.13327E-05	0.001377519	0.00058027	0.000216864	0.0007436	0.000968675	0.000297828	0.003089235	0.000178752	0.000911691	0.005041017	0.000207762	0.000631842	0.000470795	-	0.005840482	0.000599729
Y	0.000588854	0.000648257	0.001935606	0.000322434	0.002395875	0.000632197	0.000334375	0.000151436	0.008609939	0.000620203	0.001493924	0.00047255	0.000630305	0.016010447	0.000195711	0.001016958	0.000821875	0.003779879	-	0.000788984
V	0.006852074	0.000352304	0.000264674	9.06086E-05	0.00402753	0.000516723	0.000682566	0.000212427	0.00019309	0.028404089	0.0084892	0.000663351	0.002486507	0.001343144	0.000647545	0.000249751	0.007315269	0.000227274	0.00046199	-

From\To	A	R	N	D	C	Q	E	G	H	I	L	K	M	F	P	S	T	W	Y	V
A	-	0.001293534	0.001062918	0.002248338	0.001057597	0.001779796	0.00490127	0.006290392	0.000412993	0.000499744	0.001829545	0.002998108	0.000929367	0.000431451	0.003511078	0.012497651	0.006902136	8.68013E-05	0.000452894	0.007584945
R	0.002548351	-	0.001324553	0.00044816	0.000543906	0.005946053	0.001359843	0.002595977	0.002784721	0.000483315	0.002288205	0.017703579	0.000710589	0.000210528	0.001658433	0.004538847	0.001804071	0.000893018	0.000717777	0.000952269
N	0.002355584	0.001490001	-	0.016518547	0.000273148	0.003023741	0.002932984	0.00499761	0.005155072	0.001432623	0.000604743	0.009964338	0.000206179	0.000197104	0.000476135	0.014734985	0.006605857	5.51781E-05	0.002043088	0.000742028
D	0.003414296	0.000345454	0.011319116	-	3.11962E-05	0.001208178	0.019118189	0.003843288	0.001212677	0.000101939	0.000389917	0.001587457	0.000107919	9.57837E-05	0.00103482	0.003973692	0.001219822	9.95638E-05	0.000612759	0.000575995
C	0.004745098	0.001238703	0.000552999	9.21696E-05	-	0.000193568	6.61161E-05	0.001361666	0.000324412	0.000439775	0.001766885	0.000244919	0.000406159	0.000815825	0.000267023	0.005219086	0.001669261	0.000550172	0.001023111	0.003789202
Q	0.004197876	0.007118796	0.003218141	0.00187651	0.000101758	-	0.016936127	0.001465467	0.005595254	0.00029446	0.00399776	0.012885117	0.001607298	0.000204808	0.002278087	0.003814672	0.002791715	0.000165524	0.00042839	0.001139177
E	0.007313034	0.001029902	0.001974694	0.018784355	2.19873E-05	0.010713806	-	0.002520726	0.000742746	0.000329298	0.000709275	0.008549817	0.000327775	0.000166301	0.001665428	0.002613655	0.002677294	0.000120118	0.000369304	0.002226057
G	0.006545486	0.001371145	0.002346539	0.002633465	0.000315798	0.000646518	0.001757927	-	0.000324983	7.87093E-05	0.000281864	0.001235805	0.00018109	0.000102344	0.000594483	0.004974976	0.000734865	0.00025855	0.00019491	0.000708002
H	0.001464381	0.005012003	0.008247972	0.002831502	0.00025638	0.008411466	0.001765073	0.001107408	-	0.000357202	0.00229644	0.002945729	0.000420366	0.001392512	0.001699215	0.002744275	0.001540151	0.000201456	0.007287089	0.000447525
I	0.00089324	0.0004385	0.001155457	0.000119984	0.000175197	0.000223145	0.000394477	0.000135202	0.000180062	-	0.014579573	0.001071302	0.004428385	0.002171604	0.000243897	0.001184366	0.004744883	0.000163028	0.000790464	0.029573198
L	0.001838435	0.001167128	0.000274206	0.000258011	0.000395721	0.001703188	0.000477673	0.000272195	0.000650802	0.008196518	-	0.000852044	0.005048895	0.00433548	0.001014953	0.001278166	0.001062835	0.000510459	0.000749919	0.006807284
K	0.004187097	0.012550046	0.006279361	0.001459918	7.62366E-05	0.007629478	0.008002647	0.001658638	0.001160239	0.000837061	0.001184193	-	0.000971785	0.000182088	0.00135922	0.003585763	0.004513262	0.000105501	0.000250709	0.00115488
M	0.004128102	0.001602138	0.000413246	0.000315663	0.0004021	0.003026914	0.000975776	0.000773023	0.000526598	0.011004944	0.022317947	0.003090774	-	0.002440443	0.000418164	0.001831218	0.004933486	0.000395676	0.000806017	0.007783227
F	0.000972518	0.000240876	0.000200476	0.000142173	0.000409862	0.000195729	0.00025123	0.000221699	0.000885225	0.002738583	0.009725187	0.000293887	0.00123843	-	0.000394037	0.002024112	0.000559376	0.001173617	0.012142414	0.002457313
P	0.006646344	0.001593524	0.0004067	0.001289935	0.000112659	0.001828325	0.002112901	0.001081478	0.000907151	0.000258302	0.001911979	0.001842325	0.000178207	0.000330913	-	0.00598145	0.002588196	0.000106959	0.000406447	0.001190622
S	0.015573619	0.002870947	0.008285373	0.003260738	0.001449541	0.00201539	0.002182832	0.005957828	0.000964445	0.000825708	0.00158505	0.003199462	0.000513734	0.001118999	0.003937542	-	0.014246168	0.000401841	0.001480567	0.000880012
T	0.009799886	0.001300199	0.004232217	0.001140498	0.000528247	0.001680542	0.002547679	0.001002723	0.000616722	0.003769141	0.001501752	0.00458841	0.001576988	0.000352351	0.001941299	0.01623209	-	8.50604E-05	0.000547736	0.005249051
W	0.000522694	0.002729603	0.00014993	0.000394805	0.000738405	0.000422594	0.000484776	0.001496241	0.000342129	0.000549239	0.003058976	0.000454894	0.00053641	0.003135315	0.000340247	0.001941843	0.000360754	-	0.004675755	0.0013815
Y	0.001112236	0.000894764	0.002264065	0.000990948	0.000560013	0.000446047	0.000607849	0.000460013	0.005047118	0.001086081	0.001832777	0.000440864	0.000445638	0.013229387	0.000527305	0.002917881	0.000947402	0.001906916	-	0.001190029
V	0.009268105	0.000590631	0.000409129	0.000463466	0.001031956	0.000590161	0.001822996	0.000831397	0.000154221	0.020216976	0.008277653	0.001010437	0.002141091	0.001332089	0.000768547	0.000862911	0.004517329	0.000280329	0.0005921	-

From\To	A	R	N	D	C	Q	E	G	H	I	L	K	M	F	P	S	T	W	Y	V
A	-	0.000711895	0.001638121	0.000242377	0.009164999	0.000798418	0.00229156	0.00379727	0.000494326	2.70892E-05	0.002278545	0.001382322	0.00312554	2.08363E-05	0.002151511	0.01186139	0.008759904	6.08997E-05	0.001035091	0.006331655
R	0.000928649	-	0.001124201	0.00072713	0.001920683	0.005514072	0.000282909	0.001153245	0.001482979	0.00065014	0.000911418	0.021571492	0.00075811	0.000145854	0.000532333	0.002630134	0.002172999	0.000158339	0.000884533	0.000932071
N	0.001392974	0.000732833	-	0.009307268	0.002672255	0.00588833	0.002234979	0.002644025	0.005272815	0.000948121	0.000759515	0.004474357	1.33002E-05	0.001020977	0.000731957	0.007580975	0.005025061	0.000194879	0.000526956	0.000674948
D	0.000273132	0.000628143	0.012334086	-	2.0877E-05	0.001946143	0.015333651	0.001715804	0.001499457	2.70892E-05	0.000253172	0.000727538	1.33002E-05	2.08363E-05	0.001219929	0.004203058	0.001867421	1.21799E-05	1.88198E-05	0.000192842
C	0.011990501	0.001926305	0.004111362	2.42377E-05	-	0.001746539	2.82909E-05	0.00135014	0.002043216	0.002817274	0.005569778	0.00058203	0.002074827	0.001458538	2.21805E-05	0.001933922	0.003972515	0.000669897	0.0024654	0.009481413
Q	0.000874023	0.004627319	0.007580324	0.001890539	0.001461389	-	0.010524203	0.000506303	0.006376811	0.000893943	0.002734254	0.011240457	0.002101427	2.08363E-05	0.001508276	0.005801767	0.004957155	0.000121799	0.000846893	0.001157054
E	0.00221237	0.000209381	0.002537481	0.013136821	2.0877E-05	0.009281605	-	0.001968955	0.000560237	2.70892E-05	0.001063321	0.005129141	1.33002E-05	2.08363E-05	0.001153388	0.002449635	0.002784156	0.000207059	1.88198E-05	0.001124913
G	0.003687284	0.000858462	0.003019281	0.001478498	0.001002096	0.00044911	0.001980361	-	0.001120473	2.70892E-05	0.000151903	0.001091307	0.000492106	0.000145854	0.000377069	0.003919416	0.000237672	0.000280139	0.000395217	9.64211E-05
H	0.000819396	0.001884429	0.010278405	0.002205629	0.002588747	0.009655863	0.00096189	0.001912699	-	0.000921032	0.002582351	0.002764643	0.00154282	0.002937912	0.000975943	0.00471877	0.001663703	0.000584637	0.005777692	3.21404E-05
I	2.73132E-05	0.000502514	0.001124201	2.42377E-05	0.002171207	0.000823368	2.82909E-05	2.81279E-05	0.000560237	-	0.019494222	0.001236814	0.004987564	0.00133352	0.000221805	0.000103143	0.002444624	0.000475018	0.000489316	0.033683121
L	0.001229095	0.000376886	0.0004818	0.000121188	0.002296469	0.00134733	0.000594108	8.43838E-05	0.000840355	0.01042933	-	0.000836668	0.007727399	0.00372969	0.000487972	0.000618855	0.000848828	0.000572457	0.00120447	0.003599723
K	0.001037902	0.01241629	0.003950762	0.000484754	0.000334032	0.00770972	0.003989013	0.000843838	0.001252294	0.000921032	0.00116459	-	0.001782223	0.000291708	0.000953763	0.001985494	0.003734843	7.30797E-05	1.88198E-05	0.000610667
M	0.006418606	0.001193471	3.212E-05	2.42377E-05	0.003256811	0.003942187	2.82909E-05	0.001040733	0.001911395	0.010158438	0.029418553	0.004874502	-	0.005146555	2.21805E-05	2.57856E-05	0.004447858	0.001351974	0.001392668	0.00758513
F	2.73132E-05	0.000146567	0.001573881	2.42377E-05	0.001461389	2.49506E-05	2.82909E-05	0.000196895	0.002323334	0.001733707	0.009063547	0.000509276	0.003285142	-	0.000243986	0.000515713	0.002342765	0.002216749	0.019139781	0.002956915
P	0.002649382	0.000502514	0.00105996	0.001333072	2.0877E-05	0.001696637	0.001471125	0.000478175	0.000725012	0.000270892	0.001113956	0.001564206	1.33002E-05	0.000229199	-	0.003455274	0.002105093	0.000109619	0.000263478	0.00080351
S	0.012564079	0.002135686	0.009443284	0.003950741	0.001565774	0.005613874	0.002687633	0.004275445	0.003015391	0.000108357	0.001215224	0.00280102	1.33002E-05	0.000416725	0.002972191	-	0.022782541	0.000170519	0.000583415	0.001253475
T	0.007046809	0.001340038	0.004753762	0.001333072	0.002442608	0.00364278	0.002319851	0.000196895	0.0008074	0.00195042	0.001265859	0.004001457	0.001742322	0.001437702	0.001375193	0.017302158	-	1.21799E-05	0.000639875	0.006299515
W	0.000136566	0.000272195	0.00051392	2.42377E-05	0.001148235	0.000249506	0.000480945	0.000646942	0.000790922	0.001056478	0.002379814	0.000218261	0.001476319	0.003792199	0.000199625	0.000360999	3.39531E-05	-	0.003312293	0.00083565
Y	0.001502227	0.00098409	0.00089936	2.42377E-05	0.002734886	0.001122775	2.82909E-05	0.000590686	0.005058607	0.000704318	0.003240598	3.63769E-05	0.000984213	0.021190472	0.000310527	0.000799355	0.001154406	0.00214367	-	0.001896283
V	0.005380703	0.000607205	0.00067452	0.000145426	0.006158712	0.00089822	0.00099018	8.43838E-05	1.64775E-05	0.028389449	0.005671046	0.000691161	0.00313884	0.001916936	0.000554513	0.00100564	0.006654811	0.000316678	0.001110371	-

From\To	A	R	N	D	C	Q	E	G	H	I	L	K	M	F	P	S	T	W	Y	V
A	-	0.001180879	0.001674492	0.001831475	0.002192411	0.002318028	0.004578811	0.004074633	0.000534018	0.000535517	0.002060166	0.003370912	0.001215113	0.000448462	0.003262598	0.008887429	0.007363928	0.000140897	0.000463257	0.006592511
R	0.001540426	-	0.002086665	0.000365067	0.001127524	0.007744209	0.001270378	0.001681557	0.003600761	0.000517911	0.002576643	0.019904957	0.000929068	0.000218828	0.001541065	0.003227701	0.001924774	0.001449556	0.000734201	0.000827671
N	0.001423903	0.001360236	-	0.013455851	0.000566239	0.003938156	0.00274002	0.003237226	0.006665725	0.001535172	0.000680973	0.011203368	0.000269571	0.000204876	0.000442439	0.01047846	0.007047827	8.95657E-05	0.002089838	0.000644939
D	0.002063872	0.000315369	0.017831829	-	6.46702E-05	0.001573545	0.017860388	0.002489509	0.001568042	0.000109236	0.000439068	0.001784852	0.000141101	9.95602E-05	0.000961586	0.002825804	0.001301435	0.000161613	0.00062678	0.000500631
C	0.002868315	0.001130824	0.00087118	7.50805E-05	-	0.000252106	6.17663E-05	0.000882026	0.000419479	0.000471255	0.001989608	0.000275374	0.000531037	0.000847991	0.000248126	0.003711438	0.001780944	0.000893045	0.001046522	0.003293413
Q	0.002537531	0.006498814	0.005069773	0.001528587	0.000210945	-	0.015821886	0.000949264	0.007234898	0.000315537	0.004501694	0.014487336	0.002101481	0.000212884	0.002116866	0.00271272	0.002978497	0.000268681	0.000438192	0.000990124
E	0.00442058	0.000940207	0.00311088	0.015301556	4.55799E-05	0.013953787	-	0.001632813	0.000960402	0.00035287	0.000798682	0.009612957	0.000428554	0.000172858	0.001547565	0.001858643	0.002856421	0.000194977	0.000377755	0.001934794
G	0.003956613	0.001251731	0.003696674	0.002145196	0.000654654	0.000842033	0.001642271	-	0.000420217	8.43434E-05	0.000317394	0.001389473	0.000236768	0.000106379	0.000552411	0.003537844	0.000784032	0.000419682	0.00019937	0.000615365
H	0.000885189	0.004575503	0.012993632	0.002306515	0.000531478	0.010955192	0.001648948	0.000717329	-	0.000382771	0.002585916	0.00331202	0.000549613	0.001447416	0.001578961	0.001951531	0.001643196	0.000327006	0.007453831	0.000388969
I	0.000539946	0.00040031	0.001820276	9.77376E-05	0.000363185	0.000290627	0.000368524	8.75776E-05	0.000232828	-	0.016417386	0.001204514	0.005789946	0.002257226	0.000226636	0.000842236	0.005062343	0.000264628	0.000808552	0.025703762
L	0.001111296	0.001065482	0.000431977	0.000210173	0.000820333	0.002218252	0.000446246	0.000176316	0.000841515	0.008783236	-	0.000957993	0.00660124	0.00450642	0.000943125	0.000908939	0.001133944	0.000828582	0.000767078	0.005916601
K	0.002531015	0.011457053	0.009892336	0.001189235	0.000158039	0.009936722	0.007476146	0.001074391	0.001500238	0.000896979	0.001333466	-	0.001270572	0.000189267	0.001263027	0.002549936	0.004815225	0.00017125	0.000256446	0.001003772
M	0.002495354	0.001462607	0.000651017	0.000257136	0.000833558	0.003942289	0.000911579	0.00050073	0.000680914	0.011792692	0.025131212	0.003475101	-	0.002536666	0.00038857	0.001302231	0.005263564	0.000642265	0.00082446	0.006764849
F	0.000587867	0.000219898	0.000315825	0.000115813	0.000849649	0.000254919	0.000234701	0.000143607	0.001144633	0.002934615	0.010951085	0.000330431	0.001619201	-	0.000366151	0.001439402	0.000596801	0.001905027	0.012420255	0.002135792
P	0.00401758	0.001454743	0.000640704	0.001050769	0.000233544	0.002381231	0.001973892	0.000700532	0.001172984	0.000276792	0.002152992	0.002071411	0.000232999	0.00034396	-	0.004253576	0.002761361	0.000173616	0.000415747	0.001034838
S	0.009413936	0.002620914	0.013052552	0.002656166	0.003004916	0.002624867	0.002039222	0.003859213	0.001247068	0.000884813	0.001784852	0.003597303	0.000671688	0.001163119	0.003658882	-	0.015199318	0.000652273	0.001514445	0.000764869
T	0.005923832	0.001186963	0.00666732	0.000929039	0.001095061	0.002188758	0.002380065	0.000649519	0.000797448	0.004038942	0.001691053	0.005158963	0.002061852	0.000366243	0.001803913	0.011543093	-	0.000138071	0.000560269	0.004562251
W	0.000315958	0.002491879	0.000236196	0.000321604	0.001530721	0.000550391	0.000452882	0.000969198	0.000442387	0.000588554	0.003444572	0.000511459	0.000701336	0.003258934	0.000316167	0.001380899	0.00038489	-	0.004782746	0.001200741
Y	0.000672324	0.000816838	0.003566747	0.000807217	0.001160914	0.000580937	0.000567858	0.000297976	0.006526136	0.001163824	0.002063806	0.000495684	0.000582654	0.013750997	0.000489987	0.002074987	0.001010788	0.003095327	-	0.001034322
V	0.005602381	0.000539193	0.00064453	0.000377535	0.002139257	0.000768632	0.00170306	0.000538542	0.000199415	0.021664134	0.009321084	0.001136082	0.002799395	0.00138461	0.000714156	0.00061364	0.004819564	0.000455034	0.000605649	-

From\To	A	R	N	D	C	Q	E	G	H	I
A	-	0.001406695	0.001230147	0.002198318	0.000603683	0.001184586	0.003417074	0.006737331	0.000261976	0.000985256
R	0.002116925	-	0.000994184	0.000411386	0.001071318	0.006427911	0.001019895	0.005262316	0.003822464	0.000651362
N	0.002225842	0.00119536	-	0.014766812	0.000329754	0.001638467	0.001854868	0.002998704	0.004802048	0.001339627
D	0.003295334	0.000409782	0.012233714	-	0.000110983	0.001109827	0.024877209	0.004925927	0.001229347	0.000315874
C	0.002286859	0.002696767	0.000690372	0.000280464	-	0.000194167	0.000172593	0.002114265	0.000862966	0.000409909
Q	0.002216415	0.007991879	0.001694278	0.001385259	9.59026E-05	-	0.010943547	0.00089509	0.006766458	0.000213117
E	0.00425199	0.000843311	0.001275597	0.020650499	5.66932E-05	0.00727799	-	0.004322857	0.000290553	0.000304726
G	0.006935753	0.003599791	0.00170609	0.003382865	0.000574559	0.00049248	0.003576339	-	0.000240377	0.000146571
H	0.00087591	0.008492519	0.008873349	0.002741979	0.000761661	0.012091366	0.000780702	0.000780702	-	0.00049508
I	0.001440422	0.000632787	0.001082398	0.000308067	0.000158197	0.000166523	0.000358024	0.000208154	0.00021648	-
L	0.001235504	0.000985519	0.000302867	0.000163452	0.000173067	0.001509527	0.000312482	0.000269215	0.000644193	0.00636501
K	0.001472118	0.017282661	0.005579326	0.00075078	5.15241E-05	0.006315385	0.005549883	0.001045204	0.00062565	0.000552044
M	0.001871603	0.001141678	0.000729925	0.000505333	0.000430469	0.000973233	0.000561481	0.000505333	0.000393037	0.013176082
F	0.000551499	0.000173019	0.000162206	8.65096E-05	0.000713704	9.73233E-05	0.000140578	0.000194647	0.000540685	0.002119486
P	0.007813811	0.001881906	0.000268844	0.000338223	0.000130086	0.003425589	0.000615739	0.000806531	0.001361563	0.000268844
S	0.015495387	0.002652132	0.011160789	0.001566877	0.002266834	0.001168736	0.001001774	0.007262861	0.000886185	0.00110452
T	0.018266841	0.001721874	0.005188082	0.001130448	0.000494103	0.001115475	0.00106307	0.001227771	0.000568967	0.006962361
W	0.000336888	0.003338258	6.12524E-05	0.000153131	0.001163796	0.000367515	0.000367515	0.002113209	0.000153131	0.000367515
Y	0.000555918	0.000623713	0.001545724	0.001206749	0.002223672	0.000542359	0.000203385	0.000203385	0.006969315	0.000827098
V	0.01164802	0.000455104	0.000362764	0.000837655	0.000652975	0.000382551	0.00149063	0.001820415	0.000145106	0.025973897

From\To	L	K	M	F	P	S	T	W	Y	V
A	0.001463647	0.001139025	0.000569512	0.000290451	0.005131306	0.013742333	0.013896101	6.26464E-05	0.0002335	0.010057588
R	0.001756962	0.020123646	0.000522803	0.000137129	0.001859809	0.003539636	0.001971226	0.00093419	0.000394245	0.000591368
N	0.000649204	0.007811056	0.000401888	0.000154572	0.00031945	0.017909783	0.007141243	2.06096E-05	0.00117475	0.000566765
D	0.000290263	0.000870788	0.000230503	6.82971E-05	0.000332948	0.002083061	0.001289107	4.26857E-05	0.000759805	0.001084216
C	0.000776669	0.000151019	0.000496205	0.001423893	0.000323612	0.007615671	0.001423893	0.000819817	0.003538159	0.00213584
Q	0.003345934	0.00914271	0.000554104	9.59026E-05	0.004209056	0.001939363	0.00158772	0.00012787	0.000426234	0.000618039
E	0.000460632	0.005343334	0.0002126	9.21265E-05	0.000503152	0.001105517	0.001006304	8.50398E-05	0.0001063	0.001601583
G	0.00032832	0.000832525	0.000158297	0.000105531	0.000545245	0.006630885	0.000961508	0.000404537	8.79428E-05	0.001618147
H	0.002551564	0.001618529	0.000399872	0.000952076	0.002989519	0.00262773	0.001447156	9.52076E-05	0.009787342	0.000418913
I	0.01102381	0.000624461	0.005861603	0.001631924	0.00025811	0.001432096	0.007743311	9.99137E-05	0.000507895	0.032788341
L	-	0.000451896	0.004682417	0.005254498	0.002778683	0.00209603	0.000826874	0.000394208	0.000403822	0.006062143
K	0.000691895	-	0.000758141	5.15241E-05	0.000566765	0.001678214	0.002929514	6.62453E-05	0.000147212	0.000426914
M	0.018229409	0.001927751	-	0.000917085	0.000430469	0.001010665	0.006419597	0.000149728	0.000318172	0.010462258
F	0.011819378	7.56959E-05	0.000529871	-	0.000389293	0.003341434	0.000421734	0.000400107	0.009191648	0.00204379
P	0.005012633	0.000667773	0.000199465	0.000312206	-	0.009869164	0.003573019	5.20343E-05	0.000190792	0.00072848
S	0.00279983	0.001464131	0.000346768	0.001984283	0.007307812	-	0.014500035	0.000231179	0.001053147	0.001406336
T	0.001287663	0.002979591	0.002567839	0.00029197	0.003084401	0.016904314	-	5.98913E-05	0.000336888	0.003937852
W	0.00251135	0.000275636	0.00024501	0.00113317	0.000183757	0.001102544	0.00024501	-	0.001255675	0.000826908
Y	0.001138954	0.00027118	0.000230503	0.011525131	0.000298298	0.002223672	0.000610154	0.000555918	-	0.000569477
V	0.008317187	0.000382551	0.003687001	0.001246589	0.000554039	0.00144446	0.003469342	0.000178084	0.00027702	-

From\To	A	R	N	D	C	Q	E	G	H	I
A	-	0.001122774	0.001811006	0.002023206	0.001209364	0.001400815	0.003047111	0.004941754	0.000365596	0.000988406
R	0.001464631	-	0.001463624	0.000378617	0.002146182	0.007601237	0.000909472	0.003859848	0.005334384	0.000653444
N	0.001539986	0.000954093	-	0.013590533	0.0006606	0.001937546	0.001654044	0.002199514	0.006701427	0.001343909
D	0.002279933	0.000327074	0.018010311	-	0.000222333	0.001312411	0.022183781	0.00361311	0.001715598	0.000316884
C	0.001582202	0.002152464	0.001016357	0.000258123	-	0.00022961	0.000153907	0.001550789	0.001204299	0.000411219
Q	0.001533464	0.006378834	0.002494294	0.001274914	0.000192122	-	0.009758701	0.000656538	0.009442831	0.000213798
E	0.002941811	0.000673101	0.001877917	0.019005543	0.000113574	0.008606486	-	0.003170765	0.000405477	0.0003057
G	0.004798618	0.002873225	0.002511683	0.003113396	0.00115102	0.000582375	0.003189133	-	0.000335454	0.00014704
H	0.000606013	0.006778426	0.013063227	0.002523561	0.001525842	0.014298477	0.000696177	0.000572636	-	0.000496662
I	0.000996581	0.000505068	0.001593492	0.000283528	0.000316917	0.000196919	0.000319261	0.000152678	0.000302105	-
L	0.000854805	0.000786606	0.000445876	0.000150432	0.000346706	0.00178507	0.00027865	0.000197466	0.000898994	0.006385356
K	0.00101851	0.013794405	0.008213809	0.000690975	0.000103219	0.00746817	0.004949004	0.000766645	0.000873117	0.000553809
M	0.0012949	0.000911246	0.001074586	0.000465079	0.000862362	0.001150884	0.00050069	0.000370656	0.000548496	0.0132182
F	0.000381564	0.000138098	0.000238797	7.96185E-05	0.001429771	0.000115088	0.000125358	0.000142771	0.000754545	0.002126261
P	0.005406117	0.00150207	0.000395788	0.000311281	0.000260602	0.004050882	0.000549073	0.000591581	0.001900109	0.000269703
S	0.010720746	0.002116837	0.016430765	0.001442064	0.00454117	0.001382073	0.000893313	0.005327224	0.001236702	0.001108051
T	0.012638223	0.001374339	0.007637826	0.0010404	0.000989841	0.00131909	0.000947973	0.000900556	0.000794014	0.006984617
W	0.000233082	0.002664479	9.0175E-05	0.000140933	0.002331444	0.000434599	0.000327724	0.001550014	0.0002137	0.000368689
Y	0.000384621	0.000497826	0.002275594	0.001110623	0.004454703	0.000641359	0.000181364	0.00014918	0.009725925	0.000829742
V	0.00805888	0.000363248	0.000534056	0.00077093	0.001308111	0.00045238	0.001329241	0.001335253	0.0002025	0.026056924

From\To	L	K	M	F	P	S	T	W	Y	V
A	0.001584671	0.001356494	0.000630552	0.000291081	0.004390203	0.010121152	0.015710603	0.00010394	0.000265019	0.009483156
R	0.00190224	0.023965765	0.000578836	0.000137426	0.001591201	0.002606922	0.002228621	0.00154996	0.000447462	0.000557592
N	0.000702885	0.009302387	0.000444962	0.000154908	0.000273312	0.013190456	0.00807372	3.41945E-05	0.001333322	0.000534395
D	0.000314263	0.001037043	0.000255207	6.84452E-05	0.000284861	0.001534163	0.001457434	7.08219E-05	0.000862366	0.001022292
C	0.000840889	0.000179852	0.000549387	0.001426981	0.000276873	0.005608899	0.00160982	0.001360199	0.004015752	0.002013853
Q	0.003622598	0.010888287	0.000613491	9.61105E-05	0.003601152	0.00142833	0.001795039	0.000212155	0.000483768	0.00058274
E	0.000498721	0.006363514	0.000235386	9.23262E-05	0.000430483	0.000814207	0.001137704	0.000141094	0.000120648	0.00151011
G	0.000355467	0.000991475	0.000175263	0.00010576	0.000466497	0.00488361	0.001087058	0.000671187	9.98136E-05	0.001525728
H	0.002762545	0.001927548	0.000442729	0.000954141	0.002557749	0.001935309	0.00163612	0.000157964	0.011108473	0.000394988
I	0.011935335	0.000743686	0.006489838	0.001635462	0.000220832	0.001054731	0.008754404	0.000165772	0.000576452	0.030915659
L	-	0.000538175	0.005184269	0.005265893	0.002377363	0.001543715	0.000934845	0.000654049	0.000458332	0.005715908
K	0.000749106	-	0.000839397	5.16358E-05	0.000484909	0.001235995	0.003312039	0.000109911	0.000167083	0.000402531
M	0.019736742	0.002295808	-	0.000919074	0.000368297	0.000744349	0.007257844	0.000248421	0.00036112	0.009864714
F	0.012796686	9.01482E-05	0.000586662	-	0.000333069	0.002460948	0.000476803	0.000663837	0.01043237	0.00192706
P	0.005427113	0.000795268	0.000220843	0.000312883	-	0.007268584	0.004039571	8.63326E-05	0.000216546	0.000686873
S	0.003031339	0.001743671	0.000383934	0.001988586	0.006252361	-	0.016393396	0.00038356	0.001195304	0.001326015
T	0.001394135	0.003548471	0.002843055	0.000292603	0.002638928	0.012449933	-	9.93685E-05	0.000382363	0.003712944
W	0.002719006	0.000328262	0.000271269	0.001135628	0.000157218	0.000812018	0.000277002	-	0.001425171	0.00077968
Y	0.001233131	0.000322955	0.000255207	0.011550124	0.000255215	0.001637722	0.000689826	0.000922351	-	0.000536952
V	0.00900491	0.00045559	0.004082166	0.001249292	0.000474021	0.001063837	0.003922356	0.000295468	0.000314413	-

From\To	A	R	N	D	C	Q	E	G	H	I	L	K	M	F	P	S	T	W	Y	V
A	-	0.000551931	0.003114373	0.002860149	0.000739072	0.002177279	0.005463519	0.006605904	0.000370103	0.001741257	0.002024234	0.000925666	0.000936576	0.000376284	0.005424164	0.010278637	0.012306244	0	0.000452402	0.006516124
R	0.00071998	-	0.001034398	0	0.000476944	0.005982837	0	0.000242968	0.003865762	0.001685525	0.000771402	0.016496529	0.001169201	0.000280465	0.002243279	0.003884487	0.000888379	0.002396979	0.000144514	0.000760364
N	0.002648303	0.000674294	-	0.0213018	0	0.002523701	0.004162512	0.003837206	0.008587134	0.002047662	0.001699713	0.011273844	0	0.000283936	0.000915196	0.012400488	0.00759971	0.000269627	0.001755633	0.000500353
D	0.003223075	0	0.028229361	-	0	0.00330438	0.031698386	0.003432528	0.001407398	0.000636953	0	0.002563561	0	0	0.000291984	0.002423202	0.002207973	0	0	0.000563626
C	0.000966923	0.00047834	0	0	-	0	0	0.000296638	0.000458222	0.001166115	0	0	0	0	0.000408778	0.004050209	0.000542309	0	0.001764368	0.001531735
Q	0.002383454	0.005020699	0.003248879	0.003209969	0	-	0.019711635	0.000778385	0.009739321	0.000479986	0.003634274	0.005428953	0.001468908	0	0.003325187	0.0014231	0.001755071	0	0	0.001096174
E	0.005274715	0	0.004725904	0.027157005	0	0.017384271	-	0.002248458	0.000713253	0.0016248	0.000562395	0.002971841	0.000397792	0	0.00110659	0.002014789	0.001137753	0	0.000398022	0.001162281
G	0.006414568	0.000180862	0.004381806	0.00295779	0.00022017	0.000690457	0.002261483	-	0.000173256	0	0.000356099	0.000957889	0.000222243	0.000314427	0.000757346	0.005889998	0.001025246	0	0	0.001702365
H	0.000613483	0.004912241	0.016739073	0.002070214	0.000580566	0.014747426	0.001224609	0.000295755	-	0.000205173	0.002209408	0.000968247	0	0.000975428	0.002037805	0.000897368	0.000756973	0.000324195	0.002345489	0.001388343
I	0.001755659	0.001302797	0.002427942	0.000569906	0.000898698	0.000442092	0.001696878	0	0.0001248	-	0.012724752	0.001648313	0.004345221	0.003996876	0.000259779	0.00062855	0.006351188	0	0.000694112	0.027854139
L	0.001091914	0.000318987	0.001078217	0	0	0.001790823	0.000314226	0.000197817	0.000718991	0.006807691	-	0.000645958	0.006818296	0.003204521	0.000689513	0.000434539	0.001106209	0.000552725	0.000530619	0.005516954
K	0.000695027	0.009495202	0.009954564	0.001708083	0	0.003723666	0.002311247	0.000740674	0.000438584	0.001227467	0.000899133	-	0.003144923	0	0.000731483	0.002420192	0.004513628	0	0.000244747	0.000328373
M	0.001923352	0.00184064	0	0	0	0.002755609	0.000846146	0.000470011	0	0.008850144	0.025957556	0.008601581	-	0.001882312	0.000370109	0.001567129	0.003437067	0	0	0.008089909
F	0.000493253	0.000281836	0.000437701	0	0	0	0	0.000424461	0.000771379	0.005196329	0.00778733	0	0.001201515	-	0.00024085	0.001165503	0.000456466	0.000912306	0.012870724	0.000390689
P	0.006679342	0.002117623	0.001325312	0.000319064	0.000384754	0.003740453	0.001411435	0.000960417	0.001513851	0.000317269	0.00157404	0.001199659	0.00022193	0.000226253	-	0.006152912	0.002615812	0	0	0.001533475
S	0.010887561	0.003154228	0.015446737	0.002277733	0.003279198	0.001377012	0.002210539	0.00642503	0.000573436	0.000660325	0.000853289	0.003414267	0.000808322	0.000941793	0.005292671	-	0.018167353	0.000886877	0.000622768	0.000960669
T	0.009899624	0.000547843	0.007189407	0.001576177	0.000333453	0.001289719	0.000948014	0.000849349	0.000367361	0.005067235	0.001649691	0.004835837	0.001346373	0.000280123	0.00170883	0.013797162	-	0	0.000774613	0.004943923
W	0	0.00412056	0.00071104	0	0	0	0	0	0.000438584	0	0.002297784	0	0	0.001560685	0	0.001877569	0	-	0.001125835	0
Y	0.00065657	0.00016078	0.002996357	0	0.001957226	0	0.000598326	0	0.002053572	0.000999101	0.001427617	0.000473071	0	0.01424973	0	0.000853273	0.001397489	0.000728625	-	0.000884082
V	0.005537467	0.000495345	0.000500036	0.000425041	0.000994949	0.000850959	0.001023073	0.001489839	0.000711767	0.023476555	0.008691476	0.000371657	0.003347725	0.00025328	0.001058273	0.000770727	0.005222763	0	0.000517675	-

From\To	A	R	N	D	C	Q	E	G	H	I	L	K	M	F	P	S	T	W	Y	V
A	-	0.000219925	0.000525429	0.000167648	0.000179318	2.37193E-05	0.000116129	0.003373827	0.000193929	0.004193185	0.002135871	9.60155E-05	0.003756276	0.000193549	0.001492416	0.013942368	0.02112342	2.75143E-05	0.000106452	0.004287303
R	0.0008334	-	0.000259014	1.80266E-05	0.000309679	0.002758549	2.27705E-05	0.000642887	0.002311204	8.2543E-05	0.001307026	0.001623536	5.02848E-05	0.000142316	0.000647061	0.000218597	9.1841E-05	0.000319166	3.13094E-05	0.000166984
N	0.000970023	0.000126186	-	0.007536936	0.000176471	0.002165568	0.00075598	0.001492985	0.006936269	0.001180365	0.001275147	0.006991677	0.001729798	0.00045541	0.002014239	0.017774641	0.010478218	0.00015408	0.003152858	4.17459E-05
D	0.000635297	1.80266E-05	0.015470552	-	5.69262E-06	0.00069023	0.006992816	0.001588621	0.001593934	0.000189849	0.000159393	2.61861E-05	5.02848E-05	0.000148008	0.000370495	0.002479706	0.001227329	0.000288901	0.000350665	4.17459E-05
C	0.002151811	0.000980649	0.001147063	1.80266E-05	-	0.000939283	2.27705E-05	0.000860724	0.001979135	0.002723919	0.002151811	2.18217E-05	0.00016594	0.002123348	0.000861009	0.00995981	0.007906671	0.000487004	0.004198593	4.17459E-05
Q	6.83115E-05	0.002096498	0.003378286	0.000524575	0.000225428	-	0.00375713	0.000191272	0.008142346	0.000363189	0.003331322	0.00534632	0.001252092	0.000574955	0.003772785	0.001946876	0.004174589	2.75143E-05	0.000638712	0.000417459
E	0.000348388	1.80266E-05	0.001228468	0.005535979	5.69262E-06	0.003913677	-	0.000791654	0.000688048	0.000140323	0.000159393	0.003604947	5.02848E-05	7.96967E-05	0.00035484	0.00196737	0.000651236	2.75143E-05	0.000216035	0.000463379
G	0.004337777	0.000218122	0.001039757	0.000538996	9.22205E-05	8.53893E-05	0.00033928	-	2.65656E-05	0.000255883	0.000207211	0.000261861	5.02848E-05	5.69262E-05	5.21824E-05	0.004529049	0.000492601	0.000156832	5.3226E-05	5.42697E-05
H	0.000498674	0.001568317	0.009661232	0.001081598	0.0004241	0.007269952	0.000589756	5.31311E-05	-	0.00053653	0.00095636	0.001466419	0.000316794	0.001440233	0.001675054	0.002787107	0.001970406	0.000101803	0.011042831	4.17459E-05
I	0.003470222	1.80266E-05	0.000529129	4.14613E-05	0.000187857	0.000104365	3.87098E-05	0.000164707	0.000172676	-	0.027606936	0.000224764	0.013707642	0.002538909	0.000568788	0.001714618	0.016189056	2.75143E-05	0.000413284	0.026871829
L	0.000915373	0.000147818	0.000296016	1.80266E-05	7.68504E-05	0.000495732	2.27705E-05	6.90705E-05	0.000159393	0.014296449	-	0.000170209	0.014225576	0.00647251	0.001101048	0.002643653	0.005552203	0.000470495	0.000726378	0.002012152
K	0.00030057	0.001341182	0.011855453	2.1632E-05	5.69262E-06	0.005811217	0.003761684	0.000637574	0.001785206	0.000850193	0.001243268	-	0.0024187	0.000193549	0.001377614	0.003804948	0.00599471	0.000346681	0.000842223	4.17459E-05
M	0.005102866	1.80266E-05	0.00127287	1.80266E-05	1.87857E-05	0.000590609	2.27705E-05	5.31311E-05	0.000167363	0.022501224	0.045092391	0.001049624	-	0.002721073	0.000516605	0.00399622	0.023210714	0.000313663	0.000657498	0.008516161
F	0.000232259	4.50666E-05	0.000296016	4.68692E-05	0.000212335	0.000239564	3.18787E-05	5.31311E-05	0.000672109	0.003681418	0.018123029	7.41938E-05	0.002403614	-	0.000474859	0.002308927	0.001486154	0.000112809	0.007670807	0.000137761
P	0.001953708	0.00022353	0.001428279	0.000127989	9.39283E-05	0.001714902	0.000154839	5.31311E-05	0.000852755	0.000899719	0.003363201	0.000576093	0.00049782	0.000518029	-	0.006107044	0.005635695	6.05315E-05	0.00026613	0.000179507
S	0.013942368	5.76852E-05	0.00962793	0.000654367	0.000829984	0.000675999	0.00065579	0.003522594	0.001083875	0.002071829	0.006168524	0.00121547	0.002941662	0.001924106	0.004665103	-	0.026241466	0.000558541	0.001070782	4.17459E-05
T	0.017282798	1.98293E-05	0.004643756	0.000264992	0.000539091	0.001185963	0.00017761	0.000313474	0.000626947	0.016005089	0.010599661	0.001566799	0.01397918	0.001013287	0.003522309	0.02147029	-	0.000145826	0.000638712	0.004496032
W	6.83115E-05	0.000209109	0.000207211	0.00018928	0.000100759	2.37193E-05	2.27705E-05	0.000302847	9.82926E-05	8.2543E-05	0.002725627	0.000274954	0.000573247	0.000233397	0.000114801	0.001386723	0.000442506	-	0.00043207	0.000116888
Y	0.000232259	1.80266E-05	0.003726105	0.000201898	0.000763381	0.000483873	0.000157116	9.03229E-05	0.009369675	0.001089568	0.003697927	0.000587004	0.001055981	0.013946922	0.00044355	0.002336252	0.001703232	0.000379698	-	4.17459E-05
V	0.007015586	7.21065E-05	3.7002E-05	1.80266E-05	5.69262E-06	0.000237193	0.000252752	6.90705E-05	2.65656E-05	0.053132934	0.007682762	2.18217E-05	0.010258103	0.000187857	0.000224384	6.83115E-05	0.008992064	7.70401E-05	3.13094E-05	-

From\To	A	R	N	D	C	Q	E	G	H	I	L	K	M	F	P	S	T	W	Y	V
A	-	0.000219925	0.000525429	0.000167648	0.000179318	2.37193E-05	0.000116129	0.003373827	0.000193929	0.004193185	0.002135871	9.60155E-05	0.003756276	0.000193549	0.001492416	0.013942368	0.02112342	2.75143E-05	0.000106452	0.004287303
R	0.0008334	-	0.000259014	1.80266E-05	0.000309679	0.002758549	2.27705E-05	0.000642887	0.002311204	8.2543E-05	0.001307026	0.001623536	5.02848E-05	0.000142316	0.000647061	0.000218597	9.1841E-05	0.000319166	3.13094E-05	0.000166984
N	0.000970023	0.000126186	-	0.007536936	0.000176471	0.002165568	0.00075598	0.001492985	0.006936269	0.001180365	0.001275147	0.006991677	0.001729798	0.00045541	0.002014239	0.017774641	0.010478218	0.00015408	0.003152858	4.17459E-05
D	0.000635297	1.80266E-05	0.015470552	-	5.69262E-06	0.00069023	0.006992816	0.001588621	0.001593934	0.000189849	0.000159393	2.61861E-05	5.02848E-05	0.000148008	0.000370495	0.002479706	0.001227329	0.000288901	0.000350665	4.17459E-05
C	0.002151811	0.000980649	0.001147063	1.80266E-05	-	0.000939283	2.27705E-05	0.000860724	0.001979135	0.002723919	0.002151811	2.18217E-05	0.00016594	0.002123348	0.000861009	0.00995981	0.007906671	0.000487004	0.004198593	4.17459E-05
Q	6.83115E-05	0.002096498	0.003378286	0.000524575	0.000225428	-	0.00375713	0.000191272	0.008142346	0.000363189	0.003331322	0.00534632	0.001252092	0.000574955	0.003772785	0.001946876	0.004174589	2.75143E-05	0.000638712	0.000417459
E	0.000348388	1.80266E-05	0.001228468	0.005535979	5.69262E-06	0.003913677	-	0.000791654	0.000688048	0.000140323	0.000159393	0.003604947	5.02848E-05	7.96967E-05	0.00035484	0.00196737	0.000651236	2.75143E-05	0.000216035	0.000463379
G	0.004337777	0.000218122	0.001039757	0.000538996	9.22205E-05	8.53893E-05	0.00033928	-	2.65656E-05	0.000255883	0.000207211	0.000261861	5.02848E-05	5.69262E-05	5.21824E-05	0.004529049	0.000492601	0.000156832	5.3226E-05	5.42697E-05
H	0.000498674	0.001568317	0.009661232	0.001081598	0.0004241	0.007269952	0.000589756	5.31311E-05	-	0.00053653	0.00095636	0.001466419	0.000316794	0.001440233	0.001675054	0.002787107	0.001970406	0.000101803	0.011042831	4.17459E-05
I	0.003470222	1.80266E-05	0.000529129	4.14613E-05	0.000187857	0.000104365	3.87098E-05	0.000164707	0.000172676	-	0.027606936	0.000224764	0.013707642	0.002538909	0.000568788	0.001714618	0.016189056	2.75143E-05	0.000413284	0.026871829
L	0.000915373	0.000147818	0.000296016	1.80266E-05	7.68504E-05	0.000495732	2.27705E-05	6.90705E-05	0.000159393	0.014296449	-	0.000170209	0.014225576	0.00647251	0.001101048	0.002643653	0.005552203	0.000470495	0.000726378	0.002012152
K	0.00030057	0.001341182	0.011855453	2.1632E-05	5.69262E-06	0.005811217	0.003761684	0.000637574	0.001785206	0.000850193	0.001243268	-	0.0024187	0.000193549	0.001377614	0.003804948	0.00599471	0.000346681	0.000842223	4.17459E-05
M	0.005102866	1.80266E-05	0.00127287	1.80266E-05	1.87857E-05	0.000590609	2.27705E-05	5.31311E-05	0.000167363	0.022501224	0.045092391	0.001049624	-	0.002721073	0.000516605	0.00399622	0.023210714	0.000313663	0.000657498	0.008516161
F	0.000232259	4.50666E-05	0.000296016	4.68692E-05	0.000212335	0.000239564	3.18787E-05	5.31311E-05	0.000672109	0.003681418	0.018123029	7.41938E-05	0.002403614	-	0.000474859	0.002308927	0.001486154	0.000112809	0.007670807	0.000137761
P	0.001953708	0.00022353	0.001428279	0.000127989	9.39283E-05	0.001714902	0.000154839	5.31311E-05	0.000852755	0.000899719	0.003363201	0.000576093	0.00049782	0.000518029	-	0.006107044	0.005635695	6.05315E-05	0.00026613	0.000179507
S	0.013942368	5.76852E-05	0.00962793	0.000654367	0.000829984	0.000675999	0.00065579	0.003522594	0.001083875	0.002071829	0.006168524	0.00121547	0.002941662	0.001924106	0.004665103	-	0.026241466	0.000558541	0.001070782	4.17459E-05
T	0.017282798	1.98293E-05	0.004643756	0.000264992	0.000539091	0.001185963	0.00017761	0.000313474	0.000626947	0.016005089	0.010599661	0.001566799	0.01397918	0.001013287	0.003522309	0.02147029	-	0.000145826	0.000638712	0.004496032
W	6.83115E-05	0.000209109	0.000207211	0.00018928	0.000100759	2.37193E-05	2.27705E-05	0.000302847	9.82926E-05	8.2543E-05	0.002725627	0.000274954	0.000573247	0.000233397	0.000114801	0.001386723	0.000442506	-	0.00043207	0.000116888
Y	0.000232259	1.80266E-05	0.003726105	0.000201898	0.000763381	0.000483873	0.000157116	9.03229E-05	0.009369675	0.001089568	0.003697927	0.000587004	0.001055981	0.013946922	0.00044355	0.002336252	0.001703232	0.000379698	-	4.17459E-05
V	0.007015586	7.21065E-05	3.7002E-05	1.80266E-05	5.69262E-06	0.000237193	0.000252752	6.90705E-05	2.65656E-05	0.053132934	0.007682762	2.18217E-05	0.010258103	0.000187857	0.000224384	6.83115E-05	0.008992064	7.70401E-05	3.13094E-05	-

From\To	A	R	N	D	C	Q	E	G	H	I	L	K	M	F	P	S	T	W	Y	V
A	-	0.000561098	0.002038655	0.002880302	0.000617207	0.001739403	0.004975066	0.010829186	0.000392768	0.001234415	0.001776809	0.001066086	0.000542394	0.000374065	0.006452624	0.014438914	0.011034921	0	0.000374065	0.006826689
R	0.001194905	-	0.000677113	0	0.000398302	0.00477962	0	0.000398302	0.004102507	0.001194905	0.000677113	0.018998988	0.000677113	0.000278811	0.002668621	0.005456732	0.000796603	0.001075414	0.00011949	0.000796603
N	0.004395219	0.000685493	-	0.021451895	0	0.002016156	0.003790372	0.006290405	0.009113023	0.001451632	0.001491955	0.012984041	0	0.000282262	0.001088724	0.017419584	0.006814606	0.000120969	0.001451632	0.0005242
D	0.005349131	0	0.018478818	-	0	0.002639831	0.028864469	0.005627008	0.001493589	0.00045155	0	0.002952443	0	0	0.000347346	0.003403993	0.001979873	0	0	0.000590489
C	0.001604739	0.000486285	0	0	-	0	0	0.000486285	0.000486285	0.000826684	0	0	0	0	0.000486285	0.005689531	0.000486285	0	0.001458854	0.001604739
Q	0.003955666	0.005104085	0.002126702	0.003232587	0	-	0.017949364	0.001276021	0.010335771	0.000340272	0.003190053	0.006252504	0.000850681	0	0.003955666	0.0019991	0.001573759	0	0	0.001148419
E	0.008754106	0	0.003093556	0.027348355	0	0.013888094	-	0.00368594	0.000756934	0.001151856	0.000493653	0.003422658	0.000230371	0	0.001316407	0.002830275	0.001020215	0	0.000329102	0.001217676
G	0.010645847	0.000183866	0.002868311	0.002978631	0.000183866	0.000551598	0.0020593	-	0.000183866	0	0.000312572	0.001103197	0.000128706	0.000312572	0.000900944	0.008273975	0.000919331	0	0	0.001783501
H	0.001018159	0.004993825	0.010957325	0.002084801	0.000484837	0.011781549	0.001115126	0.000484837	-	0.000145451	0.00193935	0.001115126	0	0.000969675	0.002424187	0.001260577	0.000678772	0.000145451	0.00193935	0.001454512
I	0.002913755	0.001324434	0.001589321	0.000573921	0.000750513	0.000353182	0.001545173	0	0.000132443	-	0.011169392	0.001898355	0.002516424	0.003973302	0.000309035	0.000882956	0.005695066	0	0.000573921	0.029181693
L	0.001812179	0.000324285	0.000705796	0	0	0.001430668	0.000286134	0.000324285	0.000763023	0.004826119	-	0.000743947	0.003948643	0.00318562	0.000820249	0.000610418	0.00099193	0.000247982	0.000438738	0.005779898
K	0.001153491	0.009652902	0.006516215	0.001720119	0	0.002974794	0.002104616	0.001214201	0.000465444	0.000870178	0.000789231	-	0.001821302	0	0.000870178	0.003399764	0.004047338	0	0.000202367	0.000344024
M	0.003192065	0.00187121	0	0	0	0.002201424	0.000770498	0.000770498	0	0.006274058	0.022784737	0.009906407	-	0.00187121	0.000440285	0.002201424	0.003081993	0	0	0.008475482
F	0.00081862	0.000286517	0.000286517	0	0	0	0	0.000695827	0.00081862	0.00368379	0.006835476	0	0.000695827	-	0.000286517	0.00163724	0.00040931	0.00040931	0.010642059	0.00040931
P	0.011085276	0.002152793	0.000867543	0.000321312	0.000321312	0.002988205	0.001285249	0.001574431	0.001606562	0.000224919	0.001381643	0.001381643	0.000128525	0.000224919	-	0.008643302	0.00234558	0	0	0.001606562
S	0.018069388	0.003206614	0.010111367	0.002293782	0.002738495	0.001100079	0.002012911	0.010532674	0.000608555	0.000468119	0.00074899	0.003932198	0.000468119	0.000936238	0.006296199	-	0.016290537	0.000397901	0.000514931	0.001006456
T	0.016429772	0.000556941	0.004706155	0.001587283	0.000278471	0.001030342	0.000863259	0.001392354	0.000389859	0.003592272	0.001448048	0.005569414	0.000779718	0.000278471	0.002032836	0.019381561	-	0	0.000640483	0.005179555
W	0	0.004188995	0.000465444	0	0	0	0	0	0.000465444	0	0.002016924	0	0	0.00155148	0	0.002637515	0	-	0.000930888	0
Y	0.001089668	0.00016345	0.001961402	0	0.001634502	0	0.000544834	0	0.002179336	0.000708284	0.001253118	0.000544834	0	0.014165684	0	0.001198635	0.001253118	0.0003269	-	0.000926218
V	0.009190179	0.000503571	0.000327321	0.000428036	0.000830893	0.000679821	0.000931607	0.002442322	0.000755357	0.016643037	0.007629108	0.000428036	0.00193875	0.000251786	0.001258929	0.001082679	0.004683215	0	0.000428036	-

From\To	A	R	N	D	C	Q	E	G	H	I	L	K	M	F	P	S	T	W	Y	V
A	-	0.00064869	0.000934114	0.000643501	0.000596795	0.000596795	0.002433885	0.005506082	0.000160875	0.001183211	0.001972018	0.001178021	0.000404783	0.000342508	0.002086188	0.014899116	0.007166729	2.59476E-05	0.000171254	0.00628451
R	0.000788807	-	0.001458255	0.000166065	0.000731723	0.006564744	0.000778428	0.002018724	0.00173849	0.00108461	0.002029103	0.022133308	0.000269855	0.000264666	0.000373646	0.002371611	0.001691784	0.000409972	0.00097563	0.000607174
N	0.001722921	0.002210736	-	0.016129032	0.000482625	0.002895753	0.005163574	0.005397102	0.003404326	0.001338897	0.001136505	0.011987794	0.000134928	0.000487815	0.000742102	0.012714327	0.007436584	6.74638E-05	0.0022782	0.0005449
D	0.001312949	0.000275045	0.017826006	-	1.0379E-05	0.001525719	0.017950554	0.00357558	0.000809565	8.82219E-05	0.00010379	0.002075809	9.86009E-05	0.000114169	0.000726533	0.003627475	0.001437497	3.11371E-05	0.000851081	0.000487815
C	0.005007888	0.005023457	0.002174409	4.15162E-05	-	4.15162E-05	5.70847E-05	0.002511728	0.001074231	0.002241873	0.003954415	0.000259476	0.000347698	0.003627475	0.001219538	0.014229667	0.003098144	0.000778428	0.004431851	0.003845435
Q	0.001183211	0.010685224	0.003113713	0.001484203	1.0379E-05	-	0.015184539	0.001110558	0.003077386	0.000736912	0.002854237	0.01637294	0.00043592	5.18952E-05	0.001375223	0.002433885	0.00130257	9.34114E-05	0.001183211	0.000358077
E	0.003720887	0.00097563	0.004276166	0.01344086	1.0379E-05	0.011702371	-	0.00313966	0.000399593	0.001183211	0.000825134	0.012989372	0.000243908	0.000721343	0.000793997	0.003492548	0.001987587	0.00010898	0.00043073	0.00130257
G	0.004971561	0.001494582	0.002636277	0.001582804	0.000269855	0.000503384	0.001852659	-	4.67057E-05	0.00032175	0.000202391	0.001312949	4.15162E-05	0.000124549	0.000124549	0.004234649	0.000498194	0.000145307	3.11371E-05	0.000596795
H	0.000498194	0.004385146	0.005661768	0.001219538	0.000394404	0.00476917	0.000799186	0.000160875	-	0.000228339	0.000664259	0.001536098	2.07581E-05	0.000638311	0.00064869	0.001868228	0.000181633	0.000119359	0.00596795	0.000160875
I	0.001110558	0.000835513	0.000679827	3.63266E-05	0.000249097	0.000347698	0.000726533	0.000332129	6.74638E-05	-	0.017364138	0.001712542	0.003840246	0.00227301	0.000498194	0.001333707	0.005557977	7.26533E-05	0.000269855	0.031038527
L	0.001473824	0.001245485	0.000461867	3.63266E-05	0.000352887	0.001074231	0.000404783	0.000166065	0.000160875	0.013840453	-	0.00108461	0.002937269	0.006326026	0.000934114	0.003160418	0.000845892	0.000285424	0.000576037	0.005651389
K	0.001774816	0.027270935	0.009745921	0.001525719	4.67057E-05	0.012397766	0.012761033	0.002184788	0.000747291	0.002740067	0.002179599	-	0.000420351	0.000363266	0.001287001	0.00531407	0.004904098	1.55686E-05	0.000752481	0.001619131
M	0.001385602	0.000768049	0.000249097	0.000171254	0.000140117	0.00075767	0.000555279	0.000171254	2.07581E-05	0.014048034	0.013477187	0.000960061	-	0.001639889	0.000425541	0.000581226	0.003451032	0.000150496	0.00065388	0.003134471
F	0.000513763	0.00032694	0.000394404	8.30323E-05	0.00064869	4.15162E-05	0.000705775	0.000207581	0.000311371	0.003622286	0.012641674	0.000358077	0.000710964	-	0.000186823	0.002983975	0.000799186	0.000835513	0.007166729	0.002075809
P	0.003663802	0.00053971	0.000705775	0.000622743	0.000254287	0.001219538	0.000908166	0.000238718	0.000368456	0.000934114	0.002195168	0.001499772	0.00021796	0.00021796	-	0.007358741	0.001401171	8.82219E-05	0.000295803	0.000799186
S	0.018116619	0.002366422	0.008375887	0.002158841	0.002075809	0.001489393	0.002776394	0.005708473	0.000736912	0.001722921	0.005132437	0.004302113	0.000202391	0.002423506	0.005101299	-	0.011463653	0.000129738	0.001577614	0.001105368
T	0.009984639	0.001930502	0.005609873	0.00098082	0.000513763	0.000913356	0.001811143	0.000773239	7.78428E-05	0.008246149	0.001577614	0.004546021	0.001401171	0.000742102	0.001110558	0.013134678	-	5.18952E-05	0.00021796	0.004955993
W	0.00010898	0.00140636	0.000160875	6.22743E-05	0.000389214	0.000197202	0.000306182	0.000674638	0.000166065	0.000337319	0.001587994	5.18952E-05	0.000186823	0.002340474	0.000207581	0.000446299	0.000155686	-	0.001048283	6.74638E-05
Y	0.000425541	0.001977208	0.003041059	0.001032715	0.001307759	0.001473824	0.000700585	8.82219E-05	0.004784739	0.000716154	0.001904554	0.001235106	0.000467057	0.01182173	0.000415162	0.003201935	0.000389214	0.000617553	-	0.000773239
V	0.007197866	0.000570847	0.000337319	0.000269855	0.000524142	0.000207581	0.00097563	0.000752481	5.70847E-05	0.037878316	0.008656122	0.001235106	0.001043094	0.001593183	0.000524142	0.001043094	0.004073774	1.55686E-05	0.000358077	-

From\To	A	R	N	D	C	Q	E	G	H	I
A	-	0.000407695	0.001364095	0.000783644	0.00255214	0.000726599	0.00259295	0.003445	0.000200863	0.000741269
R	0.00053122	-	0.002129503	0.000202231	0.003129146	0.007992588	0.000829302	0.001263058	0.002170613	0.000679496
N	0.001160297	0.001389426	-	0.019641663	0.002063905	0.003525584	0.005501034	0.003376814	0.004250514	0.000838804
D	0.000884202	0.000172863	0.026031475	-	4.4385E-05	0.001857566	0.019123695	0.00223714	0.001010793	5.527E-05
C	0.003372551	0.003157193	0.003175309	5.05577E-05	-	5.0546E-05	6.08155E-05	0.001571518	0.001341245	0.001404509
Q	0.000796831	0.006715557	0.004546983	0.001807437	4.4385E-05	-	0.01617691	0.000694845	0.003842309	0.000461667
E	0.002505823	0.000613174	0.006244523	0.016368053	4.4385E-05	0.014247658	-	0.001964397	0.000498917	0.000741269
G	0.003348087	0.00093933	0.003849779	0.001927512	0.001154011	0.00061287	0.001973738	-	5.8315E-05	0.000201573
H	0.000335508	0.00275602	0.00826793	0.001485132	0.001686632	0.005806473	0.000851416	0.000100655	-	0.000143052
I	0.000747902	0.000525112	0.000992758	4.4238E-05	0.001065241	0.000423323	0.000774015	0.000207804	8.42327E-05	-
L	0.000992543	0.000782775	0.000674469	4.4238E-05	0.001509092	0.001307878	0.000431237	0.000103902	0.000200863	0.008670893
K	0.001195246	0.017139511	0.014232055	0.001857995	0.000199733	0.015094303	0.013595018	0.00136696	0.00093304	0.001716622
M	0.000933131	0.000482711	0.000363759	0.00020855	0.000599198	0.000922465	0.000591568	0.000107149	2.59178E-05	0.00880094
F	0.000345992	0.000205478	0.000575951	0.000101115	0.002774065	5.0546E-05	0.0007519	0.000129877	0.000388767	0.002269323
P	0.002467379	0.000339203	0.001030649	0.000758365	0.001087434	0.001484789	0.000967519	0.000149359	0.00046004	0.000585212
S	0.012200596	0.001487273	0.012231383	0.002629	0.008877009	0.001813338	0.002957842	0.003571631	0.000920081	0.001079391
T	0.006724132	0.001213301	0.008192147	0.001194425	0.00219706	0.001112012	0.001929508	0.000483794	9.71916E-05	0.005166122
W	7.33923E-05	0.000883883	0.000234927	7.58365E-05	0.001664439	0.000240094	0.000326192	0.000422102	0.000207342	0.000211327
Y	0.000286579	0.001242655	0.004440886	0.001257623	0.005592516	0.001794384	0.000746371	5.51979E-05	0.005974046	0.000448663
V	0.004847386	0.000358772	0.00049259	0.000328625	0.002241445	0.00025273	0.001039391	0.000470806	7.12739E-05	0.023730351

From\To	L	K	M	F	P	S	T	W	Y	V
A	0.001841017	0.001584897	0.000456152	0.000262902	0.002001248	0.011513324	0.008353215	3.25474E-05	0.000195691	0.005704682
R	0.001894309	0.029777906	0.000304101	0.000203151	0.000358432	0.001832668	0.001971867	0.00051425	0.001114848	0.000551154
N	0.001061007	0.016128245	0.000152051	0.000374436	0.000711887	0.009825024	0.008667746	8.46233E-05	0.002603289	0.000494626
D	9.68956E-05	0.00279277	0.000111114	8.76339E-05	0.000696952	0.002803139	0.001675482	3.90569E-05	0.000972527	0.000442808
C	0.003691723	0.000349096	0.000391823	0.002784367	0.001169884	0.010996006	0.003611057	0.000976423	0.005064256	0.003490643
Q	0.00266463	0.02202797	0.00049124	3.98336E-05	0.001319231	0.00188079	0.001518216	0.000117171	0.00135205	0.00032504
E	0.00077032	0.017475756	0.000274861	0.000553687	0.000761669	0.002698874	0.002316641	0.000136699	0.000492194	0.001182391
G	0.000188946	0.001766427	4.67848E-05	9.56006E-05	0.000119477	0.003272334	0.000580672	0.000182266	3.55803E-05	0.000541733
H	0.000620132	0.00206665	2.33924E-05	0.000489953	0.000622279	0.001443677	0.000211703	0.000149718	0.006819549	0.000146032
I	0.016210638	0.002304035	0.004327593	0.001744711	0.00047791	0.001030625	0.006478127	9.11328E-05	0.000308362	0.028174815
L	-	0.001459222	0.003310024	0.004855713	0.000896081	0.00244222	0.000985933	0.000358022	0.000658235	0.005129974
K	0.002034808	-	0.000473696	0.000278835	0.001234601	0.004106459	0.005715994	1.95285E-05	0.000859856	0.001469745
M	0.012581897	0.001291656	-	0.001258741	0.000408215	0.000449144	0.004022366	0.000188775	0.000747185	0.002845275
F	0.011801887	0.000481753	0.00080119	-	0.000179216	0.002305873	0.000931495	0.001048028	0.008189389	0.001884288
P	0.002049342	0.002017776	0.00024562	0.000167301	-	0.005686483	0.001633141	0.000110661	0.000338012	0.000725451
S	0.004791489	0.005788015	0.000228076	0.001860228	0.004893598	-	0.013361515	0.000162737	0.001802733	0.001003383
T	0.001472813	0.006116165	0.001578987	0.00056962	0.001065341	0.010149851	-	6.50949E-05	0.000249062	0.004498737
W	0.001482503	6.98192E-05	0.000210532	0.001796494	0.000199129	0.000344878	0.00018146	-	0.001197869	6.12394E-05
Y	0.001778035	0.001661698	0.000526329	0.009074089	0.000398258	0.002474302	0.00045365	0.000774629	-	0.000701897
V	0.008081095	0.001661698	0.001175468	0.001222891	0.000502801	0.000806053	0.004748207	1.95285E-05	0.000409173	-

From\To	A	R	N	D	C	Q	E	G	H	I	L	K	M	F	P	S	T	W	Y	V
A	-	0.001207701	0.000590135	0.001064639	0.001635379	0.002008068	0.003775711	0.006016155	0.00040742	0.000472971	0.001989313	0.001760201	0.001310167	0.00054504	0.00263396	0.014691894	0.005790007	0.000110741	0.000379807	0.008947381
R	0.001706943	-	0.001602891	0.00033397	0.000351211	0.00581349	0.001323241	0.00113621	0.00275498	0.000400874	0.001518882	0.02075447	0.000564302	0.000113266	0.000743751	0.002667099	0.001566879	0.000363754	0.000545428	0.000600106
N	0.001111551	0.002136107	-	0.013676704	0.000347411	0.003510884	0.001969436	0.004186316	0.005119448	0.00060452	0.000344321	0.007037541	0.00043244	0.000192332	0.000361856	0.012457818	0.005414321	2.78058E-05	0.001061619	0.000293888
D	0.001586684	0.000352157	0.01082159	-	4.11006E-05	0.001083618	0.019064497	0.002460362	0.001052575	3.37453E-05	7.58616E-05	0.000928328	2.97786E-05	3.74158E-05	0.000882249	0.003854726	0.00115248	1.83162E-05	0.000234357	0.000133329
C	0.009994813	0.001518675	0.001127254	0.000168545	-	0.000175586	1.27209E-05	0.001657657	0.000727224	0.001012128	0.002989009	4.35229E-05	0.001041667	0.002374473	0.000168601	0.008654047	0.003094533	0.000410645	0.002021733	0.006880462
Q	0.003894569	0.007977346	0.00361509	0.001410163	5.57206E-05	-	0.015009813	0.000780277	0.00546489	0.000229979	0.00293089	0.010611025	0.001949535	7.70293E-05	0.00139631	0.003803609	0.002923109	0.000144739	0.000446375	0.000738625
E	0.004170234	0.001034049	0.001154849	0.014128596	2.29891E-06	0.008547831	-	0.001015817	0.000481242	0.000139732	0.000350591	0.00592896	0.000202504	4.04127E-05	0.00093806	0.001901988	0.001636045	4.77066E-05	0.000208216	0.000860488
G	0.008296097	0.001108547	0.003064844	0.00227649	0.000374019	0.000554782	0.00126826	-	0.000353635	2.74792E-05	0.000222719	0.000973199	0.000162645	0.000192463	0.000440527	0.00540782	0.000351368	0.000164527	9.48463E-05	0.000269352
H	0.001440979	0.006894042	0.009613021	0.00249793	0.00042085	0.009965877	0.001541052	0.000907018	-	0.000343709	0.001843286	0.002287574	0.000515742	0.001465477	0.001138107	0.003076918	0.001581154	0.000365866	0.00920524	0.000417939
I	0.000601636	0.000360785	0.000408256	2.88021E-05	0.000210659	0.000150837	0.000160929	2.53483E-05	0.000123616	-	0.020857741	0.000521871	0.004981287	0.002390534	0.000175085	0.000199236	0.002797548	6.84237E-05	0.000403335	0.037396578
L	0.001587459	0.000857559	0.000145876	4.06194E-05	0.000390275	0.001205919	0.000253302	0.000128885	0.000415889	0.013084795	-	0.000451108	0.007357641	0.005570025	0.000557053	0.000566541	0.000819818	0.000379702	0.00051977	0.005979547
K	0.002154366	0.017972535	0.004572985	0.000762378	8.71603E-06	0.00669628	0.006570132	0.000863782	0.000791621	0.000502136	0.000691892	-	0.000765333	5.13844E-05	0.000873003	0.002326877	0.003076626	3.05817E-05	0.000228849	0.000650376
M	0.004513515	0.001375436	0.000790925	6.88342E-05	0.000587166	0.003462886	0.000631627	0.000406324	0.000502349	0.01349056	0.031763426	0.002154177	-	0.003864577	0.000223324	0.001078338	0.005467599	0.000426606	0.000834874	0.006667748
F	0.001018726	0.000149785	0.000190854	4.69241E-05	0.000726173	7.42342E-05	6.83889E-05	0.000260868	0.000774449	0.003512562	0.013046278	7.84698E-05	0.002096731	-	0.00021128	0.001124519	0.000446533	0.001505689	0.013536657	0.002299057
P	0.004728808	0.000944736	0.000344906	0.001062786	4.95275E-05	0.001292538	0.001524794	0.000573536	0.000577711	0.000247111	0.001253256	0.001280563	0.000116383	0.000202942	-	0.004158861	0.001546531	5.82949E-05	0.000155443	0.001041226
S	0.018981801	0.002438031	0.00854522	0.003341682	0.001829459	0.002533812	0.002224875	0.005066722	0.001123985	0.000202361	0.000917258	0.002456268	0.000404414	0.000777316	0.002992896	-	0.017515552	0.000152499	0.000694789	0.000345443
T	0.008591077	0.001644918	0.004265148	0.001147398	0.00075129	0.002236313	0.00219787	0.000378073	0.000663327	0.003263219	0.001524357	0.003729804	0.002354925	0.000354481	0.001278158	0.020115586	-	8.62956E-05	0.000426436	0.007684177
W	0.000725662	0.001686454	9.67348E-05	8.05328E-05	0.000440288	0.000489026	0.000283037	0.000781826	0.00067785	0.000352479	0.003117953	0.000163731	0.000811457	0.00527877	0.000212772	0.000773453	0.000381107	-	0.005467142	0.000665504
Y	0.000879221	0.000893333	0.001304746	0.00036402	0.000765778	0.000532788	0.000436404	0.000159221	0.006024979	0.000734009	0.001507812	0.000432841	0.000561007	0.016765559	0.00020043	0.001244884	0.000665305	0.001931387	-	0.000875515
V	0.010230865	0.000485495	0.00017841	0.000102295	0.001287294	0.000435471	0.00089084	0.000223348	0.000135118	0.033616196	0.008568116	0.000607608	0.002213133	0.001406492	0.000663161	0.000305727	0.005921685	0.000116129	0.000432459	-

From\To	A	R	N	D	C	Q	E	G	H	I	L	K	M	F	P	S	T	W	Y	V
A	-	0.000817463	0.001017768	0.000223977	0.002656196	0.001029232	0.002609543	0.004578523	0.000434685	3.6038E-05	0.00243137	0.001514657	0.001870897	1.52326E-05	0.003505981	0.011914296	0.008517278	6.66092E-05	0.000928283	0.006472136
R	0.001165741	-	0.000698468	0.000671931	0.000556652	0.007108135	0.000322166	0.001390514	0.001304055	0.000864912	0.000972548	0.02363662	0.000453792	0.000106628	0.000867459	0.002641866	0.002112813	0.000173184	0.00079326	0.000952751
N	0.001748612	0.000841506	-	0.008600712	0.000774472	0.007590587	0.00254511	0.003188008	0.00463664	0.00126133	0.000810457	0.004902705	7.96126E-06	0.000746395	0.001192757	0.007614789	0.004885881	0.00021315	0.000472581	0.000689923
D	0.000342865	0.00072129	0.007663194	-	6.05056E-06	0.002508753	0.017461387	0.002068814	0.001318544	3.6038E-05	0.000270152	0.000797188	7.96126E-06	1.52326E-05	0.001987928	0.004221805	0.001815699	1.33218E-05	1.68779E-05	0.000197121
C	0.015051777	0.002211957	0.002554398	2.23977E-05	-	0.002251445	3.22166E-05	0.001627919	0.001796698	0.003747951	0.005943349	0.00063775	0.001241957	0.001066279	3.61441E-05	0.001942548	0.003862487	0.000732702	0.002211002	0.009691777
Q	0.001097168	0.005313507	0.004709671	0.00174702	0.000423539	-	0.011984568	0.00061047	0.005607436	0.001189254	0.002917644	0.012316553	0.00125788	1.52326E-05	0.002457801	0.005827645	0.004819855	0.000133218	0.000759505	0.001182725
E	0.002777207	0.00024043	0.001576543	0.012139547	6.05056E-06	0.011964824	-	0.002374049	0.000492643	3.6038E-05	0.001134639	0.005620174	7.96126E-06	1.52326E-05	0.001879495	0.002460561	0.002707042	0.000226471	1.68779E-05	0.001149872
G	0.004628679	0.000985764	0.001875886	0.001366259	0.000290427	0.000578943	0.002255161	-	0.000985286	3.6038E-05	0.000162091	0.001195782	0.000294567	0.000106628	0.00061445	0.003936898	0.000231089	0.000306403	0.000354435	9.85604E-05
H	0.001028595	0.002163871	0.006385995	0.00203819	0.000750269	0.012447277	0.001095364	0.002306219	-	0.001225292	0.002755553	0.003029314	0.000923507	0.00214779	0.001590342	0.004739818	0.001617623	0.000639449	0.005181509	3.28535E-05
I	3.42865E-05	0.000577032	0.000698468	2.23977E-05	0.000629258	0.001061396	3.22166E-05	3.3915E-05	0.000492643	-	0.02080172	0.001355219	0.002985474	0.000974883	0.000361441	0.000103603	0.002376915	0.000519552	0.000438825	0.034430449
L	0.001542893	0.000432774	0.000299344	0.000111988	0.000665562	0.001736829	0.000676548	0.000101745	0.000738964	0.013874625	-	0.000916766	0.004625494	0.002726627	0.000795171	0.000621615	0.000825318	0.000626127	0.001080184	0.00367959
K	0.001302887	0.014257509	0.002454617	0.000447954	9.6809E-05	0.009938523	0.004542538	0.001017449	0.001101202	0.001225292	0.0012427	-	0.001066809	0.000213256	0.001554198	0.00199435	0.003631398	7.99311E-05	1.68779E-05	0.000624216
M	0.00805733	0.001370452	1.99562E-05	2.23977E-05	0.000943887	0.005081834	3.22166E-05	0.001254854	0.001680782	0.013514245	0.031391687	0.005341159	-	0.00376244	3.61441E-05	2.59006E-05	0.004324665	0.001478725	0.001248963	0.007753422
F	3.42865E-05	0.000168301	0.000977855	2.23977E-05	0.000423539	3.21635E-05	3.22166E-05	0.000237405	0.002043019	0.002306431	0.009671449	0.000558032	0.001966432	-	0.000397586	0.000518013	0.002277877	0.002424576	0.017164803	0.00302252
P	0.003325791	0.000577032	0.000658556	0.001231873	6.05056E-06	0.002187118	0.001675262	0.000576555	0.000637538	0.00036038	0.00118867	0.001713954	7.96126E-06	0.000167558	-	0.003470686	0.002046788	0.000119897	0.00023629	0.000821337
S	0.015771794	0.002452388	0.005867133	0.003650823	0.000453792	0.007236789	0.003060575	0.005155077	0.002651578	0.000144152	0.001296731	0.003069173	7.96126E-06	0.000304651	0.004843314	-	0.022151526	0.000186506	0.000523214	0.001281286
T	0.008845919	0.001538753	0.002953523	0.001231873	0.000707916	0.004695872	0.00264176	0.000237405	0.000709985	0.002594735	0.001350761	0.004384533	0.001042926	0.001051046	0.002240936	0.017379332	-	1.33218E-05	0.000573848	0.006439282
W	0.000171433	0.000312559	0.0003193	2.23977E-05	0.000332781	0.000321635	0.000547682	0.000780045	0.000695496	0.001405481	0.002539431	0.000239156	0.0008837	0.002772324	0.000325297	0.000362609	3.30127E-05	-	0.002970507	0.000854191
Y	0.001885758	0.001130022	0.000558775	2.23977E-05	0.000792623	0.001447358	3.22166E-05	0.000712215	0.004448276	0.000936988	0.003457948	3.98594E-05	0.000589134	0.015491505	0.000506018	0.00080292	0.001122432	0.002344645	-	0.001938355
V	0.006754442	0.000697247	0.000419081	0.000134386	0.001784915	0.001157886	0.00112758	0.000101745	1.44895E-05	0.03776781	0.00605141	0.000757328	0.001878858	0.001401395	0.000903603	0.001010125	0.006470491	0.000346368	0.000995795	-

From\To	A	R	N	D	C	Q	E	G	H	I	L	K	M	F	P	S	T	W	Y	V
A	-	0.000876381	0.000875472	0.000943015	0.005116576	0.002382739	0.002892968	0.005722008	0.00058222	0.000399638	0.001970991	0.001921683	0.001472004	0.000520492	0.002571938	0.012001957	0.007152603	0.000216729	0.000405743	0.008063067
R	0.001143217	-	0.002377908	0.000295817	0.001098826	0.006898187	0.001013874	0.001080657	0.003936977	0.00033872	0.001504892	0.022658506	0.000634007	0.000108164	0.000726237	0.00217878	0.001935622	0.000711895	0.000582674	0.000540794
N	0.000744456	0.001550089	-	0.012114273	0.001086939	0.004165954	0.001508991	0.003981634	0.007315899	0.000510791	0.000341149	0.007683172	0.000485856	0.000183669	0.000353336	0.010176917	0.006688505	5.44181E-05	0.001134115	0.000264842
D	0.001062674	0.000255546	0.016053956	-	0.00012859	0.001285802	0.014607308	0.002340067	0.001504173	2.85132E-05	7.51629E-05	0.001013494	3.34569E-05	3.57306E-05	0.000861475	0.003148964	0.0014237	3.58462E-05	0.000250361	0.000120152
C	0.006693979	0.001102042	0.001672295	0.00014929	-	0.000208348	9.7468E-06	0.00157661	0.001039233	0.0008552	0.002961479	4.75157E-05	0.001170338	0.002267528	0.000164631	0.007069579	0.003822788	0.000803665	0.002159793	0.006200432
Q	0.002608369	0.005788846	0.005363028	0.001249065	0.000174332	-	0.01150059	0.000742127	0.007809549	0.000194322	0.002903896	0.011584492	0.002190349	7.35599E-05	0.001363431	0.003107207	0.003611021	0.000283267	0.000476857	0.000665623
E	0.002792995	0.000750369	0.001713232	0.01251454	7.19257E-06	0.010142709	-	0.00096615	0.000687715	0.000118067	0.000347361	0.006472889	0.000227518	3.85926E-05	0.000915971	0.001553753	0.002021065	9.33656E-05	0.000222435	0.000775442
G	0.005556272	0.000804429	0.004546732	0.002016423	0.001170185	0.000658295	0.000971747	-	0.000505359	2.32186E-05	0.000220667	0.001062481	0.000182735	0.000183794	0.000430154	0.004417703	0.000434057	0.000321993	0.000101323	0.00024273
H	0.000965089	0.005002735	0.014261029	0.002212565	0.001316704	0.011825337	0.001180762	0.000862671	-	0.000290418	0.001826309	0.002497438	0.000579449	0.001399473	0.001111308	0.002513565	0.001953256	0.000716029	0.009833848	0.000376632
I	0.000402943	0.000261808	0.000605652	2.55118E-05	0.000659083	0.00017898	0.000123304	2.4109E-05	0.000176653	-	0.020665633	0.000569748	0.005596594	0.002282865	0.000170962	0.000162758	0.003455911	0.000133911	0.000430878	0.03370049
L	0.001063193	0.000622297	0.000216409	3.5979E-05	0.001221044	0.001430922	0.000194081	0.000122583	0.000594322	0.01105603	-	0.000492493	0.008266484	0.005319154	0.000543936	0.000462813	0.001012751	0.000743106	0.000555264	0.005388559
K	0.001442876	0.013041962	0.006784078	0.000675284	2.72697E-05	0.00794569	0.005034067	0.000821549	0.001131258	0.000424281	0.00068552	-	0.00085987	4.90701E-05	0.000852446	0.001900849	0.003800666	5.98508E-05	0.000244477	0.000586096
M	0.003022906	0.000998099	0.001173347	6.09705E-05	0.001837054	0.004109	0.000483956	0.000386458	0.000717877	0.011398882	0.031470872	0.002351803	-	0.003690518	0.000218066	0.000880905	0.006754321	0.000834902	0.000891886	0.006008741
F	0.000682287	0.000108693	0.000283134	4.15634E-05	0.002271961	8.8085E-05	5.23999E-05	0.000248113	0.001106719	0.002967948	0.012926117	8.56687E-05	0.002355727	-	0.000206305	0.000918631	0.000551618	0.002946753	0.014461049	0.00207183
P	0.003167097	0.000685558	0.000511672	0.000941373	0.000154956	0.001533703	0.001168304	0.000545494	0.000825572	0.000208797	0.001241713	0.001398043	0.000130759	0.000193802	-	0.003397416	0.001910485	0.000114088	0.000166058	0.000938316
S	0.012712973	0.001769183	0.012676934	0.002959927	0.00572379	0.003006578	0.00170471	0.004818995	0.00160622	0.000170986	0.000908809	0.002681609	0.000454369	0.000742306	0.002922422	-	0.021637588	0.000298453	0.000742235	0.000311302
T	0.005753834	0.001193653	0.006327398	0.001016319	0.002350544	0.00265357	0.001684019	0.000359588	0.000947921	0.002757265	0.001510317	0.00407198	0.002645814	0.000338515	0.001248061	0.016432626	-	0.000168887	0.000455557	0.006924712
W	0.000486008	0.001223794	0.000143507	7.13327E-05	0.001377519	0.00058027	0.000216864	0.0007436	0.000968675	0.000297828	0.003089235	0.000178752	0.000911691	0.005041017	0.000207762	0.000631842	0.000470795	-	0.005840482	0.000599729
Y	0.000588854	0.000648257	0.001935606	0.000322434	0.002395875	0.000632197	0.000334375	0.000151436	0.008609939	0.000620203	0.001493924	0.00047255	0.000630305	0.016010447	0.000195711	0.001016958	0.000821875	0.003779879	-	0.000788984
V	0.006852074	0.000352304	0.000264674	9.06086E-05	0.00402753	0.000516723	0.000682566	0.000212427	0.00019309	0.028404089	0.0084892	0.000663351	0.002486507	0.001343144	0.000647545	0.000249751	0.007315269	0.000227274	0.00046199	-

From\To	A	R	N	D	C	Q	E	G	H	I
A	-	0.001293534	0.001062918	0.002248338	0.001057597	0.001779796	0.00490127	0.006290392	0.000412993	0.000499744
R	0.002548351	-	0.001324553	0.00044816	0.000543906	0.005946053	0.001359843	0.002595977	0.002784721	0.000483315
N	0.002355584	0.001490001	-	0.016518547	0.000273148	0.003023741	0.002932984	0.00499761	0.005155072	0.001432623
D	0.003414296	0.000345454	0.011319116	-	3.11962E-05	0.001208178	0.019118189	0.003843288	0.001212677	0.000101939
C	0.004745098	0.001238703	0.000552999	9.21696E-05	-	0.000193568	6.61161E-05	0.001361666	0.000324412	0.000439775
Q	0.004197876	0.007118796	0.003218141	0.00187651	0.000101758	-	0.016936127	0.001465467	0.005595254	0.00029446
E	0.007313034	0.001029902	0.001974694	0.018784355	2.19873E-05	0.010713806	-	0.002520726	0.000742746	0.000329298
G	0.006545486	0.001371145	0.002346539	0.002633465	0.000315798	0.000646518	0.001757927	-	0.000324983	7.87093E-05
H	0.001464381	0.005012003	0.008247972	0.002831502	0.00025638	0.008411466	0.001765073	0.001107408	-	0.000357202
I	0.00089324	0.0004385	0.001155457	0.000119984	0.000175197	0.000223145	0.000394477	0.000135202	0.000180062	-
L	0.001838435	0.001167128	0.000274206	0.000258011	0.000395721	0.001703188	0.000477673	0.000272195	0.000650802	0.008196518
K	0.004187097	0.012550046	0.006279361	0.001459918	7.62366E-05	0.007629478	0.008002647	0.001658638	0.001160239	0.000837061
M	0.004128102	0.001602138	0.000413246	0.000315663	0.0004021	0.003026914	0.000975776	0.000773023	0.000526598	0.011004944
F	0.000972518	0.000240876	0.000200476	0.000142173	0.000409862	0.000195729	0.00025123	0.000221699	0.000885225	0.002738583
P	0.006646344	0.001593524	0.0004067	0.001289935	0.000112659	0.001828325	0.002112901	0.001081478	0.000907151	0.000258302
S	0.015573619	0.002870947	0.008285373	0.003260738	0.001449541	0.00201539	0.002182832	0.005957828	0.000964445	0.000825708
T	0.009799886	0.001300199	0.004232217	0.001140498	0.000528247	0.001680542	0.002547679	0.001002723	0.000616722	0.003769141
W	0.000522694	0.002729603	0.00014993	0.000394805	0.000738405	0.000422594	0.000484776	0.001496241	0.000342129	0.000549239
Y	0.001112236	0.000894764	0.002264065	0.000990948	0.000560013	0.000446047	0.000607849	0.000460013	0.005047118	0.001086081
V	0.009268105	0.000590631	0.000409129	0.000463466	0.001031956	0.000590161	0.001822996	0.000831397	0.000154221	0.020216976

From\To	L	K	M	F	P	S	T	W	Y	V
A	0.001829545	0.002998108	0.000929367	0.000431451	0.003511078	0.012497651	0.006902136	8.68013E-05	0.000452894	0.007584945
R	0.002288205	0.017703579	0.000710589	0.000210528	0.001658433	0.004538847	0.001804071	0.000893018	0.000717777	0.000952269
N	0.000604743	0.009964338	0.000206179	0.000197104	0.000476135	0.014734985	0.006605857	5.51781E-05	0.002043088	0.000742028
D	0.000389917	0.001587457	0.000107919	9.57837E-05	0.00103482	0.003973692	0.001219822	9.95638E-05	0.000612759	0.000575995
C	0.001766885	0.000244919	0.000406159	0.000815825	0.000267023	0.005219086	0.001669261	0.000550172	0.001023111	0.003789202
Q	0.00399776	0.012885117	0.001607298	0.000204808	0.002278087	0.003814672	0.002791715	0.000165524	0.00042839	0.001139177
E	0.000709275	0.008549817	0.000327775	0.000166301	0.001665428	0.002613655	0.002677294	0.000120118	0.000369304	0.002226057
G	0.000281864	0.001235805	0.00018109	0.000102344	0.000594483	0.004974976	0.000734865	0.00025855	0.00019491	0.000708002
H	0.00229644	0.002945729	0.000420366	0.001392512	0.001699215	0.002744275	0.001540151	0.000201456	0.007287089	0.000447525
I	0.014579573	0.001071302	0.004428385	0.002171604	0.000243897	0.001184366	0.004744883	0.000163028	0.000790464	0.029573198
L	-	0.000852044	0.005048895	0.00433548	0.001014953	0.001278166	0.001062835	0.000510459	0.000749919	0.006807284
K	0.001184193	-	0.000971785	0.000182088	0.00135922	0.003585763	0.004513262	0.000105501	0.000250709	0.00115488
M	0.022317947	0.003090774	-	0.002440443	0.000418164	0.001831218	0.004933486	0.000395676	0.000806017	0.007783227
F	0.009725187	0.000293887	0.00123843	-	0.000394037	0.002024112	0.000559376	0.001173617	0.012142414	0.002457313
P	0.001911979	0.001842325	0.000178207	0.000330913	-	0.00598145	0.002588196	0.000106959	0.000406447	0.001190622
S	0.00158505	0.003199462	0.000513734	0.001118999	0.003937542	-	0.014246168	0.000401841	0.001480567	0.000880012
T	0.001501752	0.00458841	0.001576988	0.000352351	0.001941299	0.01623209	-	8.50604E-05	0.000547736	0.005249051
W	0.003058976	0.000454894	0.00053641	0.003135315	0.000340247	0.001941843	0.000360754	-	0.004675755	0.0013815
Y	0.001832777	0.000440864	0.000445638	0.013229387	0.000527305	0.002917881	0.000947402	0.001906916	-	0.001190029
V	0.008277653	0.001010437	0.002141091	0.001332089	0.000768547	0.000862911	0.004517329	0.000280329	0.0005921	-

From\To	A	R	N	D	C	Q	E	G	H	I	L	K	M	F	P	S	T	W	Y	V
A	-	0.000711895	0.001638121	0.000242377	0.009164999	0.000798418	0.00229156	0.00379727	0.000494326	2.70892E-05	0.002278545	0.001382322	0.00312554	2.08363E-05	0.002151511	0.01186139	0.008759904	6.08997E-05	0.001035091	0.006331655
R	0.000928649	-	0.001124201	0.00072713	0.001920683	0.005514072	0.000282909	0.001153245	0.001482979	0.00065014	0.000911418	0.021571492	0.00075811	0.000145854	0.000532333	0.002630134	0.002172999	0.000158339	0.000884533	0.000932071
N	0.001392974	0.000732833	-	0.009307268	0.002672255	0.00588833	0.002234979	0.002644025	0.005272815	0.000948121	0.000759515	0.004474357	1.33002E-05	0.001020977	0.000731957	0.007580975	0.005025061	0.000194879	0.000526956	0.000674948
D	0.000273132	0.000628143	0.012334086	-	2.0877E-05	0.001946143	0.015333651	0.001715804	0.001499457	2.70892E-05	0.000253172	0.000727538	1.33002E-05	2.08363E-05	0.001219929	0.004203058	0.001867421	1.21799E-05	1.88198E-05	0.000192842
C	0.011990501	0.001926305	0.004111362	2.42377E-05	-	0.001746539	2.82909E-05	0.00135014	0.002043216	0.002817274	0.005569778	0.00058203	0.002074827	0.001458538	2.21805E-05	0.001933922	0.003972515	0.000669897	0.0024654	0.009481413
Q	0.000874023	0.004627319	0.007580324	0.001890539	0.001461389	-	0.010524203	0.000506303	0.006376811	0.000893943	0.002734254	0.011240457	0.002101427	2.08363E-05	0.001508276	0.005801767	0.004957155	0.000121799	0.000846893	0.001157054
E	0.00221237	0.000209381	0.002537481	0.013136821	2.0877E-05	0.009281605	-	0.001968955	0.000560237	2.70892E-05	0.001063321	0.005129141	1.33002E-05	2.08363E-05	0.001153388	0.002449635	0.002784156	0.000207059	1.88198E-05	0.001124913
G	0.003687284	0.000858462	0.003019281	0.001478498	0.001002096	0.00044911	0.001980361	-	0.001120473	2.70892E-05	0.000151903	0.001091307	0.000492106	0.000145854	0.000377069	0.003919416	0.000237672	0.000280139	0.000395217	9.64211E-05
H	0.000819396	0.001884429	0.010278405	0.002205629	0.002588747	0.009655863	0.00096189	0.001912699	-	0.000921032	0.002582351	0.002764643	0.00154282	0.002937912	0.000975943	0.00471877	0.001663703	0.000584637	0.005777692	3.21404E-05
I	2.73132E-05	0.000502514	0.001124201	2.42377E-05	0.002171207	0.000823368	2.82909E-05	2.81279E-05	0.000560237	-	0.019494222	0.001236814	0.004987564	0.00133352	0.000221805	0.000103143	0.002444624	0.000475018	0.000489316	0.033683121
L	0.001229095	0.000376886	0.0004818	0.000121188	0.002296469	0.00134733	0.000594108	8.43838E-05	0.000840355	0.01042933	-	0.000836668	0.007727399	0.00372969	0.000487972	0.000618855	0.000848828	0.000572457	0.00120447	0.003599723
K	0.001037902	0.01241629	0.003950762	0.000484754	0.000334032	0.00770972	0.003989013	0.000843838	0.001252294	0.000921032	0.00116459	-	0.001782223	0.000291708	0.000953763	0.001985494	0.003734843	7.30797E-05	1.88198E-05	0.000610667
M	0.006418606	0.001193471	3.212E-05	2.42377E-05	0.003256811	0.003942187	2.82909E-05	0.001040733	0.001911395	0.010158438	0.029418553	0.004874502	-	0.005146555	2.21805E-05	2.57856E-05	0.004447858	0.001351974	0.001392668	0.00758513
F	2.73132E-05	0.000146567	0.001573881	2.42377E-05	0.001461389	2.49506E-05	2.82909E-05	0.000196895	0.002323334	0.001733707	0.009063547	0.000509276	0.003285142	-	0.000243986	0.000515713	0.002342765	0.002216749	0.019139781	0.002956915
P	0.002649382	0.000502514	0.00105996	0.001333072	2.0877E-05	0.001696637	0.001471125	0.000478175	0.000725012	0.000270892	0.001113956	0.001564206	1.33002E-05	0.000229199	-	0.003455274	0.002105093	0.000109619	0.000263478	0.00080351
S	0.012564079	0.002135686	0.009443284	0.003950741	0.001565774	0.005613874	0.002687633	0.004275445	0.003015391	0.000108357	0.001215224	0.00280102	1.33002E-05	0.000416725	0.002972191	-	0.022782541	0.000170519	0.000583415	0.001253475
T	0.007046809	0.001340038	0.004753762	0.001333072	0.002442608	0.00364278	0.002319851	0.000196895	0.0008074	0.00195042	0.001265859	0.004001457	0.001742322	0.001437702	0.001375193	0.017302158	-	1.21799E-05	0.000639875	0.006299515
W	0.000136566	0.000272195	0.00051392	2.42377E-05	0.001148235	0.000249506	0.000480945	0.000646942	0.000790922	0.001056478	0.002379814	0.000218261	0.001476319	0.003792199	0.000199625	0.000360999	3.39531E-05	-	0.003312293	0.00083565
Y	0.001502227	0.00098409	0.00089936	2.42377E-05	0.002734886	0.001122775	2.82909E-05	0.000590686	0.005058607	0.000704318	0.003240598	3.63769E-05	0.000984213	0.021190472	0.000310527	0.000799355	0.001154406	0.00214367	-	0.001896283
V	0.005380703	0.000607205	0.00067452	0.000145426	0.006158712	0.00089822	0.00099018	8.43838E-05	1.64775E-05	0.028389449	0.005671046	0.000691161	0.00313884	0.001916936	0.000554513	0.00100564	0.006654811	0.000316678	0.001110371	-

From\To	A	R	N	D	C	Q	E	G	H	I	L	K	M	F	P	S	T	W	Y	V
A	-	0.001180879	0.001674492	0.001831475	0.002192411	0.002318028	0.004578811	0.004074633	0.000534018	0.000535517	0.002060166	0.003370912	0.001215113	0.000448462	0.003262598	0.008887429	0.007363928	0.000140897	0.000463257	0.006592511
R	0.001540426	-	0.002086665	0.000365067	0.001127524	0.007744209	0.001270378	0.001681557	0.003600761	0.000517911	0.002576643	0.019904957	0.000929068	0.000218828	0.001541065	0.003227701	0.001924774	0.001449556	0.000734201	0.000827671
N	0.001423903	0.001360236	-	0.013455851	0.000566239	0.003938156	0.00274002	0.003237226	0.006665725	0.001535172	0.000680973	0.011203368	0.000269571	0.000204876	0.000442439	0.01047846	0.007047827	8.95657E-05	0.002089838	0.000644939
D	0.002063872	0.000315369	0.017831829	-	6.46702E-05	0.001573545	0.017860388	0.002489509	0.001568042	0.000109236	0.000439068	0.001784852	0.000141101	9.95602E-05	0.000961586	0.002825804	0.001301435	0.000161613	0.00062678	0.000500631
C	0.002868315	0.001130824	0.00087118	7.50805E-05	-	0.000252106	6.17663E-05	0.000882026	0.000419479	0.000471255	0.001989608	0.000275374	0.000531037	0.000847991	0.000248126	0.003711438	0.001780944	0.000893045	0.001046522	0.003293413
Q	0.002537531	0.006498814	0.005069773	0.001528587	0.000210945	-	0.015821886	0.000949264	0.007234898	0.000315537	0.004501694	0.014487336	0.002101481	0.000212884	0.002116866	0.00271272	0.002978497	0.000268681	0.000438192	0.000990124
E	0.00442058	0.000940207	0.00311088	0.015301556	4.55799E-05	0.013953787	-	0.001632813	0.000960402	0.00035287	0.000798682	0.009612957	0.000428554	0.000172858	0.001547565	0.001858643	0.002856421	0.000194977	0.000377755	0.001934794
G	0.003956613	0.001251731	0.003696674	0.002145196	0.000654654	0.000842033	0.001642271	-	0.000420217	8.43434E-05	0.000317394	0.001389473	0.000236768	0.000106379	0.000552411	0.003537844	0.000784032	0.000419682	0.00019937	0.000615365
H	0.000885189	0.004575503	0.012993632	0.002306515	0.000531478	0.010955192	0.001648948	0.000717329	-	0.000382771	0.002585916	0.00331202	0.000549613	0.001447416	0.001578961	0.001951531	0.001643196	0.000327006	0.007453831	0.000388969
I	0.000539946	0.00040031	0.001820276	9.77376E-05	0.000363185	0.000290627	0.000368524	8.75776E-05	0.000232828	-	0.016417386	0.001204514	0.005789946	0.002257226	0.000226636	0.000842236	0.005062343	0.000264628	0.000808552	0.025703762
L	0.001111296	0.001065482	0.000431977	0.000210173	0.000820333	0.002218252	0.000446246	0.000176316	0.000841515	0.008783236	-	0.000957993	0.00660124	0.00450642	0.000943125	0.000908939	0.001133944	0.000828582	0.000767078	0.005916601
K	0.002531015	0.011457053	0.009892336	0.001189235	0.000158039	0.009936722	0.007476146	0.001074391	0.001500238	0.000896979	0.001333466	-	0.001270572	0.000189267	0.001263027	0.002549936	0.004815225	0.00017125	0.000256446	0.001003772
M	0.002495354	0.001462607	0.000651017	0.000257136	0.000833558	0.003942289	0.000911579	0.00050073	0.000680914	0.011792692	0.025131212	0.003475101	-	0.002536666	0.00038857	0.001302231	0.005263564	0.000642265	0.00082446	0.006764849
F	0.000587867	0.000219898	0.000315825	0.000115813	0.000849649	0.000254919	0.000234701	0.000143607	0.001144633	0.002934615	0.010951085	0.000330431	0.001619201	-	0.000366151	0.001439402	0.000596801	0.001905027	0.012420255	0.002135792
P	0.00401758	0.001454743	0.000640704	0.001050769	0.000233544	0.002381231	0.001973892	0.000700532	0.001172984	0.000276792	0.002152992	0.002071411	0.000232999	0.00034396	-	0.004253576	0.002761361	0.000173616	0.000415747	0.001034838
S	0.009413936	0.002620914	0.013052552	0.002656166	0.003004916	0.002624867	0.002039222	0.003859213	0.001247068	0.000884813	0.001784852	0.003597303	0.000671688	0.001163119	0.003658882	-	0.015199318	0.000652273	0.001514445	0.000764869
T	0.005923832	0.001186963	0.00666732	0.000929039	0.001095061	0.002188758	0.002380065	0.000649519	0.000797448	0.004038942	0.001691053	0.005158963	0.002061852	0.000366243	0.001803913	0.011543093	-	0.000138071	0.000560269	0.004562251
W	0.000315958	0.002491879	0.000236196	0.000321604	0.001530721	0.000550391	0.000452882	0.000969198	0.000442387	0.000588554	0.003444572	0.000511459	0.000701336	0.003258934	0.000316167	0.001380899	0.00038489	-	0.004782746	0.001200741
Y	0.000672324	0.000816838	0.003566747	0.000807217	0.001160914	0.000580937	0.000567858	0.000297976	0.006526136	0.001163824	0.002063806	0.000495684	0.000582654	0.013750997	0.000489987	0.002074987	0.001010788	0.003095327	-	0.001034322
V	0.005602381	0.000539193	0.00064453	0.000377535	0.002139257	0.000768632	0.00170306	0.000538542	0.000199415	0.021664134	0.009321084	0.001136082	0.002799395	0.00138461	0.000714156	0.00061364	0.004819564	0.000455034	0.000605649	-

From\To	A	R	N	D	C	Q	E	G	H	I
A	-	0.001406695	0.001230147	0.002198318	0.000603683	0.001184586	0.003417074	0.006737331	0.000261976	0.000985256
R	0.002116925	-	0.000994184	0.000411386	0.001071318	0.006427911	0.001019895	0.005262316	0.003822464	0.000651362
N	0.002225842	0.00119536	-	0.014766812	0.000329754	0.001638467	0.001854868	0.002998704	0.004802048	0.001339627
D	0.003295334	0.000409782	0.012233714	-	0.000110983	0.001109827	0.024877209	0.004925927	0.001229347	0.000315874
C	0.002286859	0.002696767	0.000690372	0.000280464	-	0.000194167	0.000172593	0.002114265	0.000862966	0.000409909
Q	0.002216415	0.007991879	0.001694278	0.001385259	9.59026E-05	-	0.010943547	0.00089509	0.006766458	0.000213117
E	0.00425199	0.000843311	0.001275597	0.020650499	5.66932E-05	0.00727799	-	0.004322857	0.000290553	0.000304726
G	0.006935753	0.003599791	0.00170609	0.003382865	0.000574559	0.00049248	0.003576339	-	0.000240377	0.000146571
H	0.00087591	0.008492519	0.008873349	0.002741979	0.000761661	0.012091366	0.000780702	0.000780702	-	0.00049508
I	0.001440422	0.000632787	0.001082398	0.000308067	0.000158197	0.000166523	0.000358024	0.000208154	0.00021648	-
L	0.001235504	0.000985519	0.000302867	0.000163452	0.000173067	0.001509527	0.000312482	0.000269215	0.000644193	0.00636501
K	0.001472118	0.017282661	0.005579326	0.00075078	5.15241E-05	0.006315385	0.005549883	0.001045204	0.00062565	0.000552044
M	0.001871603	0.001141678	0.000729925	0.000505333	0.000430469	0.000973233	0.000561481	0.000505333	0.000393037	0.013176082
F	0.000551499	0.000173019	0.000162206	8.65096E-05	0.000713704	9.73233E-05	0.000140578	0.000194647	0.000540685	0.002119486
P	0.007813811	0.001881906	0.000268844	0.000338223	0.000130086	0.003425589	0.000615739	0.000806531	0.001361563	0.000268844
S	0.015495387	0.002652132	0.011160789	0.001566877	0.002266834	0.001168736	0.001001774	0.007262861	0.000886185	0.00110452
T	0.018266841	0.001721874	0.005188082	0.001130448	0.000494103	0.001115475	0.00106307	0.001227771	0.000568967	0.006962361
W	0.000336888	0.003338258	6.12524E-05	0.000153131	0.001163796	0.000367515	0.000367515	0.002113209	0.000153131	0.000367515
Y	0.000555918	0.000623713	0.001545724	0.001206749	0.002223672	0.000542359	0.000203385	0.000203385	0.006969315	0.000827098
V	0.01164802	0.000455104	0.000362764	0.000837655	0.000652975	0.000382551	0.00149063	0.001820415	0.000145106	0.025973897

From\To	L	K	M	F	P	S	T	W	Y	V
A	0.001463647	0.001139025	0.000569512	0.000290451	0.005131306	0.013742333	0.013896101	6.26464E-05	0.0002335	0.010057588
R	0.001756962	0.020123646	0.000522803	0.000137129	0.001859809	0.003539636	0.001971226	0.00093419	0.000394245	0.000591368
N	0.000649204	0.007811056	0.000401888	0.000154572	0.00031945	0.017909783	0.007141243	2.06096E-05	0.00117475	0.000566765
D	0.000290263	0.000870788	0.000230503	6.82971E-05	0.000332948	0.002083061	0.001289107	4.26857E-05	0.000759805	0.001084216
C	0.000776669	0.000151019	0.000496205	0.001423893	0.000323612	0.007615671	0.001423893	0.000819817	0.003538159	0.00213584
Q	0.003345934	0.00914271	0.000554104	9.59026E-05	0.004209056	0.001939363	0.00158772	0.00012787	0.000426234	0.000618039
E	0.000460632	0.005343334	0.0002126	9.21265E-05	0.000503152	0.001105517	0.001006304	8.50398E-05	0.0001063	0.001601583
G	0.00032832	0.000832525	0.000158297	0.000105531	0.000545245	0.006630885	0.000961508	0.000404537	8.79428E-05	0.001618147
H	0.002551564	0.001618529	0.000399872	0.000952076	0.002989519	0.00262773	0.001447156	9.52076E-05	0.009787342	0.000418913
I	0.01102381	0.000624461	0.005861603	0.001631924	0.00025811	0.001432096	0.007743311	9.99137E-05	0.000507895	0.032788341
L	-	0.000451896	0.004682417	0.005254498	0.002778683	0.00209603	0.000826874	0.000394208	0.000403822	0.006062143
K	0.000691895	-	0.000758141	5.15241E-05	0.000566765	0.001678214	0.002929514	6.62453E-05	0.000147212	0.000426914
M	0.018229409	0.001927751	-	0.000917085	0.000430469	0.001010665	0.006419597	0.000149728	0.000318172	0.010462258
F	0.011819378	7.56959E-05	0.000529871	-	0.000389293	0.003341434	0.000421734	0.000400107	0.009191648	0.00204379
P	0.005012633	0.000667773	0.000199465	0.000312206	-	0.009869164	0.003573019	5.20343E-05	0.000190792	0.00072848
S	0.00279983	0.001464131	0.000346768	0.001984283	0.007307812	-	0.014500035	0.000231179	0.001053147	0.001406336
T	0.001287663	0.002979591	0.002567839	0.00029197	0.003084401	0.016904314	-	5.98913E-05	0.000336888	0.003937852
W	0.00251135	0.000275636	0.00024501	0.00113317	0.000183757	0.001102544	0.00024501	-	0.001255675	0.000826908
Y	0.001138954	0.00027118	0.000230503	0.011525131	0.000298298	0.002223672	0.000610154	0.000555918	-	0.000569477
V	0.008317187	0.000382551	0.003687001	0.001246589	0.000554039	0.00144446	0.003469342	0.000178084	0.00027702	-

From\To	A	R	N	D	C	Q	E	G	H	I	L	K	M	F	P	S	T	W	Y	V
A	-	0.000476734	0.000851223	0.000420681	0.001227319	4.6565E-05	0.000269275	0.003333424	0.000224489	0.002568257	0.001266278	0.000298715	0.001854202	0.000132214	0.001183906	0.009821999	0.01603169	2.27313E-05	0.000119419	0.006160282
R	0.000621887	-	0.000419617	4.52345E-05	0.002119561	0.005415505	5.2799E-05	0.000635188	0.002675414	5.05562E-05	0.000774887	0.005051002	2.4822E-05	9.72162E-05	0.000513302	0.000153995	6.9703E-05	0.000263683	3.51233E-05	0.000239933
N	0.000723836	0.000273536	-	0.01891256	0.001207838	0.004251381	0.001752926	0.001475106	0.008029316	0.000722954	0.000755987	0.021751897	0.000853876	0.000311092	0.001597859	0.012521725	0.007952479	0.000127295	0.003536915	5.99833E-05
D	0.000474062	3.90766E-05	0.025063115	-	3.89625E-05	0.00135504	0.016214563	0.001569597	0.001845113	0.000116279	9.44984E-05	8.14678E-05	2.4822E-05	0.000101105	0.000293907	0.001746882	0.000931486	0.000238679	0.000393381	5.99833E-05
C	0.001605693	0.002125765	0.001858303	4.52345E-05	-	0.001843973	5.2799E-05	0.000850417	0.002291015	0.001668356	0.001275728	6.78898E-05	8.19125E-05	0.001450466	0.000683022	0.007016401	0.006000795	0.000402344	0.004710033	5.99833E-05
Q	5.09744E-05	0.004544604	0.005473003	0.001316325	0.001542916	-	0.00871183	0.000188982	0.009425452	0.000222447	0.001975016	0.016633005	0.000618067	0.000392754	0.00299288	0.001371519	0.003168318	2.27313E-05	0.000716515	0.000599833
E	0.000259969	3.90766E-05	0.001990183	0.013891526	3.89625E-05	0.007683219	-	0.000782174	0.000796474	8.59456E-05	9.44984E-05	0.011215398	2.4822E-05	5.44411E-05	0.000281488	0.001385956	0.000494258	2.27313E-05	0.000242351	0.000665814
G	0.003236873	0.000472826	0.001684462	0.001352513	0.000631193	0.000167634	0.000786705	-	3.07519E-05	0.000156724	0.000122848	0.000814678	2.4822E-05	3.88865E-05	4.13953E-05	0.003190586	0.000373862	0.000129568	5.97096E-05	7.79783E-05
H	0.000372113	0.00339966	0.015651708	0.002714072	0.002902708	0.014272161	0.001367493	5.24949E-05	-	0.000328616	0.00056699	0.004562196	0.000156378	0.000983828	0.001328789	0.001963437	0.001495446	8.41058E-05	0.012387983	5.99833E-05
I	0.002589499	3.90766E-05	0.000857217	0.000104039	0.001285763	0.000204886	8.97582E-05	0.000162734	0.000199887	-	0.016367117	0.000699265	0.006766471	0.001734338	0.000451209	0.001207899	0.012286738	2.27313E-05	0.000463627	0.038611235
L	0.000683057	0.000320428	0.000479562	4.52345E-05	0.000525994	0.000973208	5.2799E-05	6.82433E-05	0.000184511	0.008756342	-	0.000529541	0.007022137	0.004421395	0.000873441	0.001862378	0.004213863	0.000388705	0.00081486	0.002891194
K	0.000224287	0.002907296	0.019206462	5.42814E-05	3.89625E-05	0.011408416	0.008722389	0.000629938	0.002066526	0.000520729	0.000737087	-	0.001193937	0.000132214	0.001092836	0.002680477	0.004549705	0.000286414	0.000944816	5.99833E-05
M	0.003807786	3.90766E-05	0.002062117	4.52345E-05	0.000128576	0.001159468	5.2799E-05	5.24949E-05	0.000193737	0.013781633	0.026733588	0.0032655	-	0.001858775	0.000409813	0.002815223	0.017615849	0.000259137	0.000737589	0.012236588
F	0.000173313	9.76914E-05	0.000479562	0.00011761	0.001453302	0.000470306	7.39186E-05	5.24949E-05	0.000778023	0.002254809	0.010744464	0.000230825	0.00118649	-	0.000376697	0.001626573	0.001127921	9.31983E-05	0.008605205	0.000197945
P	0.001457867	0.000484549	0.002313887	0.000321165	0.000642882	0.003366647	0.000359033	5.24949E-05	0.000987135	0.000551063	0.001993916	0.001792291	0.000245738	0.000353867	-	0.004302238	0.004277229	5.00089E-05	0.000298548	0.000257928
S	0.010403871	0.000125045	0.015597758	0.001642014	0.005680736	0.001327101	0.00152061	0.00348041	0.001254677	0.001268962	0.003657087	0.003781463	0.001452086	0.001314364	0.00370074	-	0.019916048	0.000461445	0.001201216	5.99833E-05
T	0.012896518	4.29842E-05	0.007523131	0.000664948	0.003689751	0.002328248	0.000411832	0.00030972	0.000725744	0.009802856	0.006284141	0.004874489	0.006900509	0.00069218	0.002794183	0.015125205	-	0.000120476	0.000716515	0.006460199
W	5.09744E-05	0.000453288	0.000335693	0.000474963	0.000689637	4.6565E-05	5.2799E-05	0.000299221	0.000113782	5.05562E-05	0.001615922	0.000855412	0.000282971	0.000159435	9.10697E-05	0.000976906	0.000335842	-	0.000484701	0.000167953
Y	0.000173313	3.90766E-05	0.006036488	0.000506627	0.005224874	0.000949925	0.000364313	8.92413E-05	0.010846189	0.000667342	0.002192362	0.001826236	0.000521262	0.009527192	0.00035186	0.001645822	0.001292674	0.000313692	-	5.99833E-05
V	0.005235069	0.000156306	5.99453E-05	4.52345E-05	3.89625E-05	0.00046565	0.000586069	6.82433E-05	3.07519E-05	0.032543056	0.004554821	6.78898E-05	0.005063683	0.000128325	0.000178	4.81235E-05	0.006824557	6.36477E-05	3.51233E-05	-

From\To	A	R	N	D	C	Q	E	G	H	I	L	K	M	F	P	S	T	W	Y	V
A	-	0.001122774	0.001811006	0.002023206	0.001209364	0.001400815	0.003047111	0.004941754	0.000365596	0.000988406	0.001584671	0.001356494	0.000630552	0.000291081	0.004390203	0.010121152	0.015710603	0.00010394	0.000265019	0.009483156
R	0.001464631	-	0.001463624	0.000378617	0.002146182	0.007601237	0.000909472	0.003859848	0.005334384	0.000653444	0.00190224	0.023965765	0.000578836	0.000137426	0.001591201	0.002606922	0.002228621	0.00154996	0.000447462	0.000557592
N	0.001539986	0.000954093	-	0.013590533	0.0006606	0.001937546	0.001654044	0.002199514	0.006701427	0.001343909	0.000702885	0.009302387	0.000444962	0.000154908	0.000273312	0.013190456	0.00807372	3.41945E-05	0.001333322	0.000534395
D	0.002279933	0.000327074	0.018010311	-	0.000222333	0.001312411	0.022183781	0.00361311	0.001715598	0.000316884	0.000314263	0.001037043	0.000255207	6.84452E-05	0.000284861	0.001534163	0.001457434	7.08219E-05	0.000862366	0.001022292
C	0.001582202	0.002152464	0.001016357	0.000258123	-	0.00022961	0.000153907	0.001550789	0.001204299	0.000411219	0.000840889	0.000179852	0.000549387	0.001426981	0.000276873	0.005608899	0.00160982	0.001360199	0.004015752	0.002013853
Q	0.001533464	0.006378834	0.002494294	0.001274914	0.000192122	-	0.009758701	0.000656538	0.009442831	0.000213798	0.003622598	0.010888287	0.000613491	9.61105E-05	0.003601152	0.00142833	0.001795039	0.000212155	0.000483768	0.00058274
E	0.002941811	0.000673101	0.001877917	0.019005543	0.000113574	0.008606486	-	0.003170765	0.000405477	0.0003057	0.000498721	0.006363514	0.000235386	9.23262E-05	0.000430483	0.000814207	0.001137704	0.000141094	0.000120648	0.00151011
G	0.004798618	0.002873225	0.002511683	0.003113396	0.00115102	0.000582375	0.003189133	-	0.000335454	0.00014704	0.000355467	0.000991475	0.000175263	0.00010576	0.000466497	0.00488361	0.001087058	0.000671187	9.98136E-05	0.001525728
H	0.000606013	0.006778426	0.013063227	0.002523561	0.001525842	0.014298477	0.000696177	0.000572636	-	0.000496662	0.002762545	0.001927548	0.000442729	0.000954141	0.002557749	0.001935309	0.00163612	0.000157964	0.011108473	0.000394988
I	0.000996581	0.000505068	0.001593492	0.000283528	0.000316917	0.000196919	0.000319261	0.000152678	0.000302105	-	0.011935335	0.000743686	0.006489838	0.001635462	0.000220832	0.001054731	0.008754404	0.000165772	0.000576452	0.030915659
L	0.000854805	0.000786606	0.000445876	0.000150432	0.000346706	0.00178507	0.00027865	0.000197466	0.000898994	0.006385356	-	0.000538175	0.005184269	0.005265893	0.002377363	0.001543715	0.000934845	0.000654049	0.000458332	0.005715908
K	0.00101851	0.013794405	0.008213809	0.000690975	0.000103219	0.00746817	0.004949004	0.000766645	0.000873117	0.000553809	0.000749106	-	0.000839397	5.16358E-05	0.000484909	0.001235995	0.003312039	0.000109911	0.000167083	0.000402531
M	0.0012949	0.000911246	0.001074586	0.000465079	0.000862362	0.001150884	0.00050069	0.000370656	0.000548496	0.0132182	0.019736742	0.002295808	-	0.000919074	0.000368297	0.000744349	0.007257844	0.000248421	0.00036112	0.009864714
F	0.000381564	0.000138098	0.000238797	7.96185E-05	0.001429771	0.000115088	0.000125358	0.000142771	0.000754545	0.002126261	0.012796686	9.01482E-05	0.000586662	-	0.000333069	0.002460948	0.000476803	0.000663837	0.01043237	0.00192706
P	0.005406117	0.00150207	0.000395788	0.000311281	0.000260602	0.004050882	0.000549073	0.000591581	0.001900109	0.000269703	0.005427113	0.000795268	0.000220843	0.000312883	-	0.007268584	0.004039571	8.63326E-05	0.000216546	0.000686873
S	0.010720746	0.002116837	0.016430765	0.001442064	0.00454117	0.001382073	0.000893313	0.005327224	0.001236702	0.001108051	0.003031339	0.001743671	0.000383934	0.001988586	0.006252361	-	0.016393396	0.00038356	0.001195304	0.001326015
T	0.012638223	0.001374339	0.007637826	0.0010404	0.000989841	0.00131909	0.000947973	0.000900556	0.000794014	0.006984617	0.001394135	0.003548471	0.002843055	0.000292603	0.002638928	0.012449933	-	9.93685E-05	0.000382363	0.003712944
W	0.000233082	0.002664479	9.0175E-05	0.000140933	0.002331444	0.000434599	0.000327724	0.001550014	0.0002137	0.000368689	0.002719006	0.000328262	0.000271269	0.001135628	0.000157218	0.000812018	0.000277002	-	0.001425171	0.00077968
Y	0.000384621	0.000497826	0.002275594	0.001110623	0.004454703	0.000641359	0.000181364	0.00014918	0.009725925	0.000829742	0.001233131	0.000322955	0.000255207	0.011550124	0.000255215	0.001637722	0.000689826	0.000922351	-	0.000536952
V	0.00805888	0.000363248	0.000534056	0.00077093	0.001308111	0.00045238	0.001329241	0.001335253	0.0002025	0.026056924	0.00900491	0.00045559	0.004082166	0.001249292	0.000474021	0.001063837	0.003922356	0.000295468	0.000314413	-

From\To	A	R	N	D	C	Q	E	G	H	I	L	K	M	F	P	S	T	W	Y	V
A	-	0.000551931	0.003114373	0.002860149	0.000739072	0.002177279	0.005463519	0.006605904	0.000370103	0.001741257	0.002024234	0.000925666	0.000936576	0.000376284	0.005424164	0.010278637	0.012306244	0	0.000452402	0.006516124
R	0.00071998	-	0.001034398	0	0.000476944	0.005982837	0	0.000242968	0.003865762	0.001685525	0.000771402	0.016496529	0.001169201	0.000280465	0.002243279	0.003884487	0.000888379	0.002396979	0.000144514	0.000760364
N	0.002648303	0.000674294	-	0.0213018	0	0.002523701	0.004162512	0.003837206	0.008587134	0.002047662	0.001699713	0.011273844	0	0.000283936	0.000915196	0.012400488	0.00759971	0.000269627	0.001755633	0.000500353
D	0.003223075	0	0.028229361	-	0	0.00330438	0.031698386	0.003432528	0.001407398	0.000636953	0	0.002563561	0	0	0.000291984	0.002423202	0.002207973	0	0	0.000563626
C	0.000966923	0.00047834	0	0	-	0	0	0.000296638	0.000458222	0.001166115	0	0	0	0	0.000408778	0.004050209	0.000542309	0	0.001764368	0.001531735
Q	0.002383454	0.005020699	0.003248879	0.003209969	0	-	0.019711635	0.000778385	0.009739321	0.000479986	0.003634274	0.005428953	0.001468908	0	0.003325187	0.0014231	0.001755071	0	0	0.001096174
E	0.005274715	0	0.004725904	0.027157005	0	0.017384271	-	0.002248458	0.000713253	0.0016248	0.000562395	0.002971841	0.000397792	0	0.00110659	0.002014789	0.001137753	0	0.000398022	0.001162281
G	0.006414568	0.000180862	0.004381806	0.00295779	0.00022017	0.000690457	0.002261483	-	0.000173256	0	0.000356099	0.000957889	0.000222243	0.000314427	0.000757346	0.005889998	0.001025246	0	0	0.001702365
H	0.000613483	0.004912241	0.016739073	0.002070214	0.000580566	0.014747426	0.001224609	0.000295755	-	0.000205173	0.002209408	0.000968247	0	0.000975428	0.002037805	0.000897368	0.000756973	0.000324195	0.002345489	0.001388343
I	0.001755659	0.001302797	0.002427942	0.000569906	0.000898698	0.000442092	0.001696878	0	0.0001248	-	0.012724752	0.001648313	0.004345221	0.003996876	0.000259779	0.00062855	0.006351188	0	0.000694112	0.027854139
L	0.001091914	0.000318987	0.001078217	0	0	0.001790823	0.000314226	0.000197817	0.000718991	0.006807691	-	0.000645958	0.006818296	0.003204521	0.000689513	0.000434539	0.001106209	0.000552725	0.000530619	0.005516954
K	0.000695027	0.009495202	0.009954564	0.001708083	0	0.003723666	0.002311247	0.000740674	0.000438584	0.001227467	0.000899133	-	0.003144923	0	0.000731483	0.002420192	0.004513628	0	0.000244747	0.000328373
M	0.001923352	0.00184064	0	0	0	0.002755609	0.000846146	0.000470011	0	0.008850144	0.025957556	0.008601581	-	0.001882312	0.000370109	0.001567129	0.003437067	0	0	0.008089909
F	0.000493253	0.000281836	0.000437701	0	0	0	0	0.000424461	0.000771379	0.005196329	0.00778733	0	0.001201515	-	0.00024085	0.001165503	0.000456466	0.000912306	0.012870724	0.000390689
P	0.006679342	0.002117623	0.001325312	0.000319064	0.000384754	0.003740453	0.001411435	0.000960417	0.001513851	0.000317269	0.00157404	0.001199659	0.00022193	0.000226253	-	0.006152912	0.002615812	0	0	0.001533475
S	0.010887561	0.003154228	0.015446737	0.002277733	0.003279198	0.001377012	0.002210539	0.00642503	0.000573436	0.000660325	0.000853289	0.003414267	0.000808322	0.000941793	0.005292671	-	0.018167353	0.000886877	0.000622768	0.000960669
T	0.009899624	0.000547843	0.007189407	0.001576177	0.000333453	0.001289719	0.000948014	0.000849349	0.000367361	0.005067235	0.001649691	0.004835837	0.001346373	0.000280123	0.00170883	0.013797162	-	0	0.000774613	0.004943923
W	0	0.00412056	0.00071104	0	0	0	0	0	0.000438584	0	0.002297784	0	0	0.001560685	0	0.001877569	0	-	0.001125835	0
Y	0.00065657	0.00016078	0.002996357	0	0.001957226	0	0.000598326	0	0.002053572	0.000999101	0.001427617	0.000473071	0	0.01424973	0	0.000853273	0.001397489	0.000728625	-	0.000884082
V	0.005537467	0.000495345	0.000500036	0.000425041	0.000994949	0.000850959	0.001023073	0.001489839	0.000711767	0.023476555	0.008691476	0.000371657	0.003347725	0.00025328	0.001058273	0.000770727	0.005222763	0	0.000517675	-

From\To	A	R	N	D	C	Q	E	G	H	I	L	K	M	F	P	S	T	W	Y	V
A	-	0.00064869	0.000934114	0.000643501	0.000596795	0.000596795	0.002433885	0.005506082	0.000160875	0.001183211	0.001972018	0.001178021	0.000404783	0.000342508	0.002086188	0.014899116	0.007166729	2.59476E-05	0.000171254	0.00628451
R	0.000788807	-	0.001458255	0.000166065	0.000731723	0.006564744	0.000778428	0.002018724	0.00173849	0.00108461	0.002029103	0.022133308	0.000269855	0.000264666	0.000373646	0.002371611	0.001691784	0.000409972	0.00097563	0.000607174
N	0.001722921	0.002210736	-	0.016129032	0.000482625	0.002895753	0.005163574	0.005397102	0.003404326	0.001338897	0.001136505	0.011987794	0.000134928	0.000487815	0.000742102	0.012714327	0.007436584	6.74638E-05	0.0022782	0.0005449
D	0.001312949	0.000275045	0.017826006	-	1.0379E-05	0.001525719	0.017950554	0.00357558	0.000809565	8.82219E-05	0.00010379	0.002075809	9.86009E-05	0.000114169	0.000726533	0.003627475	0.001437497	3.11371E-05	0.000851081	0.000487815
C	0.005007888	0.005023457	0.002174409	4.15162E-05	-	4.15162E-05	5.70847E-05	0.002511728	0.001074231	0.002241873	0.003954415	0.000259476	0.000347698	0.003627475	0.001219538	0.014229667	0.003098144	0.000778428	0.004431851	0.003845435
Q	0.001183211	0.010685224	0.003113713	0.001484203	1.0379E-05	-	0.015184539	0.001110558	0.003077386	0.000736912	0.002854237	0.01637294	0.00043592	5.18952E-05	0.001375223	0.002433885	0.00130257	9.34114E-05	0.001183211	0.000358077
E	0.003720887	0.00097563	0.004276166	0.01344086	1.0379E-05	0.011702371	-	0.00313966	0.000399593	0.001183211	0.000825134	0.012989372	0.000243908	0.000721343	0.000793997	0.003492548	0.001987587	0.00010898	0.00043073	0.00130257
G	0.004971561	0.001494582	0.002636277	0.001582804	0.000269855	0.000503384	0.001852659	-	4.67057E-05	0.00032175	0.000202391	0.001312949	4.15162E-05	0.000124549	0.000124549	0.004234649	0.000498194	0.000145307	3.11371E-05	0.000596795
H	0.000498194	0.004385146	0.005661768	0.001219538	0.000394404	0.00476917	0.000799186	0.000160875	-	0.000228339	0.000664259	0.001536098	2.07581E-05	0.000638311	0.00064869	0.001868228	0.000181633	0.000119359	0.00596795	0.000160875
I	0.001110558	0.000835513	0.000679827	3.63266E-05	0.000249097	0.000347698	0.000726533	0.000332129	6.74638E-05	-	0.017364138	0.001712542	0.003840246	0.00227301	0.000498194	0.001333707	0.005557977	7.26533E-05	0.000269855	0.031038527
L	0.001473824	0.001245485	0.000461867	3.63266E-05	0.000352887	0.001074231	0.000404783	0.000166065	0.000160875	0.013840453	-	0.00108461	0.002937269	0.006326026	0.000934114	0.003160418	0.000845892	0.000285424	0.000576037	0.005651389
K	0.001774816	0.027270935	0.009745921	0.001525719	4.67057E-05	0.012397766	0.012761033	0.002184788	0.000747291	0.002740067	0.002179599	-	0.000420351	0.000363266	0.001287001	0.00531407	0.004904098	1.55686E-05	0.000752481	0.001619131
M	0.001385602	0.000768049	0.000249097	0.000171254	0.000140117	0.00075767	0.000555279	0.000171254	2.07581E-05	0.014048034	0.013477187	0.000960061	-	0.001639889	0.000425541	0.000581226	0.003451032	0.000150496	0.00065388	0.003134471
F	0.000513763	0.00032694	0.000394404	8.30323E-05	0.00064869	4.15162E-05	0.000705775	0.000207581	0.000311371	0.003622286	0.012641674	0.000358077	0.000710964	-	0.000186823	0.002983975	0.000799186	0.000835513	0.007166729	0.002075809
P	0.003663802	0.00053971	0.000705775	0.000622743	0.000254287	0.001219538	0.000908166	0.000238718	0.000368456	0.000934114	0.002195168	0.001499772	0.00021796	0.00021796	-	0.007358741	0.001401171	8.82219E-05	0.000295803	0.000799186
S	0.018116619	0.002366422	0.008375887	0.002158841	0.002075809	0.001489393	0.002776394	0.005708473	0.000736912	0.001722921	0.005132437	0.004302113	0.000202391	0.002423506	0.005101299	-	0.011463653	0.000129738	0.001577614	0.001105368
T	0.009984639	0.001930502	0.005609873	0.00098082	0.000513763	0.000913356	0.001811143	0.000773239	7.78428E-05	0.008246149	0.001577614	0.004546021	0.001401171	0.000742102	0.001110558	0.013134678	-	5.18952E-05	0.00021796	0.004955993
W	0.00010898	0.00140636	0.000160875	6.22743E-05	0.000389214	0.000197202	0.000306182	0.000674638	0.000166065	0.000337319	0.001587994	5.18952E-05	0.000186823	0.002340474	0.000207581	0.000446299	0.000155686	-	0.001048283	6.74638E-05
Y	0.000425541	0.001977208	0.003041059	0.001032715	0.001307759	0.001473824	0.000700585	8.82219E-05	0.004784739	0.000716154	0.001904554	0.001235106	0.000467057	0.01182173	0.000415162	0.003201935	0.000389214	0.000617553	-	0.000773239
V	0.007197866	0.000570847	0.000337319	0.000269855	0.000524142	0.000207581	0.00097563	0.000752481	5.70847E-05	0.037878316	0.008656122	0.001235106	0.001043094	0.001593183	0.000524142	0.001043094	0.004073774	1.55686E-05	0.000358077	-

From\To	A	R	N	D	C	Q	E	G	H	I	L	K	M	F	P	S	T	W	Y	V
A	-	0.000561098	0.002038655	0.002880302	0.000617207	0.001739403	0.004975066	0.010829186	0.000392768	0.001234415	0.001776809	0.001066086	0.000542394	0.000374065	0.006452624	0.014438914	0.011034921	0	0.000374065	0.006826689
R	0.001194905	-	0.000677113	0	0.000398302	0.00477962	0	0.000398302	0.004102507	0.001194905	0.000677113	0.018998988	0.000677113	0.000278811	0.002668621	0.005456732	0.000796603	0.001075414	0.00011949	0.000796603
N	0.004395219	0.000685493	-	0.021451895	0	0.002016156	0.003790372	0.006290405	0.009113023	0.001451632	0.001491955	0.012984041	0	0.000282262	0.001088724	0.017419584	0.006814606	0.000120969	0.001451632	0.0005242
D	0.005349131	0	0.018478818	-	0	0.002639831	0.028864469	0.005627008	0.001493589	0.00045155	0	0.002952443	0	0	0.000347346	0.003403993	0.001979873	0	0	0.000590489
C	0.001604739	0.000486285	0	0	-	0	0	0.000486285	0.000486285	0.000826684	0	0	0	0	0.000486285	0.005689531	0.000486285	0	0.001458854	0.001604739
Q	0.003955666	0.005104085	0.002126702	0.003232587	0	-	0.017949364	0.001276021	0.010335771	0.000340272	0.003190053	0.006252504	0.000850681	0	0.003955666	0.0019991	0.001573759	0	0	0.001148419
E	0.008754106	0	0.003093556	0.027348355	0	0.013888094	-	0.00368594	0.000756934	0.001151856	0.000493653	0.003422658	0.000230371	0	0.001316407	0.002830275	0.001020215	0	0.000329102	0.001217676
G	0.010645847	0.000183866	0.002868311	0.002978631	0.000183866	0.000551598	0.0020593	-	0.000183866	0	0.000312572	0.001103197	0.000128706	0.000312572	0.000900944	0.008273975	0.000919331	0	0	0.001783501
H	0.001018159	0.004993825	0.010957325	0.002084801	0.000484837	0.011781549	0.001115126	0.000484837	-	0.000145451	0.00193935	0.001115126	0	0.000969675	0.002424187	0.001260577	0.000678772	0.000145451	0.00193935	0.001454512
I	0.002913755	0.001324434	0.001589321	0.000573921	0.000750513	0.000353182	0.001545173	0	0.000132443	-	0.011169392	0.001898355	0.002516424	0.003973302	0.000309035	0.000882956	0.005695066	0	0.000573921	0.029181693
L	0.001812179	0.000324285	0.000705796	0	0	0.001430668	0.000286134	0.000324285	0.000763023	0.004826119	-	0.000743947	0.003948643	0.00318562	0.000820249	0.000610418	0.00099193	0.000247982	0.000438738	0.005779898
K	0.001153491	0.009652902	0.006516215	0.001720119	0	0.002974794	0.002104616	0.001214201	0.000465444	0.000870178	0.000789231	-	0.001821302	0	0.000870178	0.003399764	0.004047338	0	0.000202367	0.000344024
M	0.003192065	0.00187121	0	0	0	0.002201424	0.000770498	0.000770498	0	0.006274058	0.022784737	0.009906407	-	0.00187121	0.000440285	0.002201424	0.003081993	0	0	0.008475482
F	0.00081862	0.000286517	0.000286517	0	0	0	0	0.000695827	0.00081862	0.00368379	0.006835476	0	0.000695827	-	0.000286517	0.00163724	0.00040931	0.00040931	0.010642059	0.00040931
P	0.011085276	0.002152793	0.000867543	0.000321312	0.000321312	0.002988205	0.001285249	0.001574431	0.001606562	0.000224919	0.001381643	0.001381643	0.000128525	0.000224919	-	0.008643302	0.00234558	0	0	0.001606562
S	0.018069388	0.003206614	0.010111367	0.002293782	0.002738495	0.001100079	0.002012911	0.010532674	0.000608555	0.000468119	0.00074899	0.003932198	0.000468119	0.000936238	0.006296199	-	0.016290537	0.000397901	0.000514931	0.001006456
T	0.016429772	0.000556941	0.004706155	0.001587283	0.000278471	0.001030342	0.000863259	0.001392354	0.000389859	0.003592272	0.001448048	0.005569414	0.000779718	0.000278471	0.002032836	0.019381561	-	0	0.000640483	0.005179555
W	0	0.004188995	0.000465444	0	0	0	0	0	0.000465444	0	0.002016924	0	0	0.00155148	0	0.002637515	0	-	0.000930888	0
Y	0.001089668	0.00016345	0.001961402	0	0.001634502	0	0.000544834	0	0.002179336	0.000708284	0.001253118	0.000544834	0	0.014165684	0	0.001198635	0.001253118	0.0003269	-	0.000926218
V	0.009190179	0.000503571	0.000327321	0.000428036	0.000830893	0.000679821	0.000931607	0.002442322	0.000755357	0.016643037	0.007629108	0.000428036	0.00193875	0.000251786	0.001258929	0.001082679	0.004683215	0	0.000428036	-

From\To	A	R	N	D	C	Q	E	G	H	I
A	-	0.000407695	0.001364095	0.000783644	0.00255214	0.000726599	0.00259295	0.003445	0.000200863	0.000741269
R	0.00053122	-	0.002129503	0.000202231	0.003129146	0.007992588	0.000829302	0.001263058	0.002170613	0.000679496
N	0.001160297	0.001389426	-	0.019641663	0.002063905	0.003525584	0.005501034	0.003376814	0.004250514	0.000838804
D	0.000884202	0.000172863	0.026031475	-	4.4385E-05	0.001857566	0.019123695	0.00223714	0.001010793	5.527E-05
C	0.003372551	0.003157193	0.003175309	5.05577E-05	-	5.0546E-05	6.08155E-05	0.001571518	0.001341245	0.001404509
Q	0.000796831	0.006715557	0.004546983	0.001807437	4.4385E-05	-	0.01617691	0.000694845	0.003842309	0.000461667
E	0.002505823	0.000613174	0.006244523	0.016368053	4.4385E-05	0.014247658	-	0.001964397	0.000498917	0.000741269
G	0.003348087	0.00093933	0.003849779	0.001927512	0.001154011	0.00061287	0.001973738	-	5.8315E-05	0.000201573
H	0.000335508	0.00275602	0.00826793	0.001485132	0.001686632	0.005806473	0.000851416	0.000100655	-	0.000143052
I	0.000747902	0.000525112	0.000992758	4.4238E-05	0.001065241	0.000423323	0.000774015	0.000207804	8.42327E-05	-
L	0.000992543	0.000782775	0.000674469	4.4238E-05	0.001509092	0.001307878	0.000431237	0.000103902	0.000200863	0.008670893
K	0.001195246	0.017139511	0.014232055	0.001857995	0.000199733	0.015094303	0.013595018	0.00136696	0.00093304	0.001716622
M	0.000933131	0.000482711	0.000363759	0.00020855	0.000599198	0.000922465	0.000591568	0.000107149	2.59178E-05	0.00880094
F	0.000345992	0.000205478	0.000575951	0.000101115	0.002774065	5.0546E-05	0.0007519	0.000129877	0.000388767	0.002269323
P	0.002467379	0.000339203	0.001030649	0.000758365	0.001087434	0.001484789	0.000967519	0.000149359	0.00046004	0.000585212
S	0.012200596	0.001487273	0.012231383	0.002629	0.008877009	0.001813338	0.002957842	0.003571631	0.000920081	0.001079391
T	0.006724132	0.001213301	0.008192147	0.001194425	0.00219706	0.001112012	0.001929508	0.000483794	9.71916E-05	0.005166122
W	7.33923E-05	0.000883883	0.000234927	7.58365E-05	0.001664439	0.000240094	0.000326192	0.000422102	0.000207342	0.000211327
Y	0.000286579	0.001242655	0.004440886	0.001257623	0.005592516	0.001794384	0.000746371	5.51979E-05	0.005974046	0.000448663
V	0.004847386	0.000358772	0.00049259	0.000328625	0.002241445	0.00025273	0.001039391	0.000470806	7.12739E-05	0.023730351

From\To	L	K	M	F	P	S	T	W	Y	V
A	0.001841017	0.001584897	0.000456152	0.000262902	0.002001248	0.011513324	0.008353215	3.25474E-05	0.000195691	0.005704682
R	0.001894309	0.029777906	0.000304101	0.000203151	0.000358432	0.001832668	0.001971867	0.00051425	0.001114848	0.000551154
N	0.001061007	0.016128245	0.000152051	0.000374436	0.000711887	0.009825024	0.008667746	8.46233E-05	0.002603289	0.000494626
D	9.68956E-05	0.00279277	0.000111114	8.76339E-05	0.000696952	0.002803139	0.001675482	3.90569E-05	0.000972527	0.000442808
C	0.003691723	0.000349096	0.000391823	0.002784367	0.001169884	0.010996006	0.003611057	0.000976423	0.005064256	0.003490643
Q	0.00266463	0.02202797	0.00049124	3.98336E-05	0.001319231	0.00188079	0.001518216	0.000117171	0.00135205	0.00032504
E	0.00077032	0.017475756	0.000274861	0.000553687	0.000761669	0.002698874	0.002316641	0.000136699	0.000492194	0.001182391
G	0.000188946	0.001766427	4.67848E-05	9.56006E-05	0.000119477	0.003272334	0.000580672	0.000182266	3.55803E-05	0.000541733
H	0.000620132	0.00206665	2.33924E-05	0.000489953	0.000622279	0.001443677	0.000211703	0.000149718	0.006819549	0.000146032
I	0.016210638	0.002304035	0.004327593	0.001744711	0.00047791	0.001030625	0.006478127	9.11328E-05	0.000308362	0.028174815
L	-	0.001459222	0.003310024	0.004855713	0.000896081	0.00244222	0.000985933	0.000358022	0.000658235	0.005129974
K	0.002034808	-	0.000473696	0.000278835	0.001234601	0.004106459	0.005715994	1.95285E-05	0.000859856	0.001469745
M	0.012581897	0.001291656	-	0.001258741	0.000408215	0.000449144	0.004022366	0.000188775	0.000747185	0.002845275
F	0.011801887	0.000481753	0.00080119	-	0.000179216	0.002305873	0.000931495	0.001048028	0.008189389	0.001884288
P	0.002049342	0.002017776	0.00024562	0.000167301	-	0.005686483	0.001633141	0.000110661	0.000338012	0.000725451
S	0.004791489	0.005788015	0.000228076	0.001860228	0.004893598	-	0.013361515	0.000162737	0.001802733	0.001003383
T	0.001472813	0.006116165	0.001578987	0.00056962	0.001065341	0.010149851	-	6.50949E-05	0.000249062	0.004498737
W	0.001482503	6.98192E-05	0.000210532	0.001796494	0.000199129	0.000344878	0.00018146	-	0.001197869	6.12394E-05
Y	0.001778035	0.001661698	0.000526329	0.009074089	0.000398258	0.002474302	0.00045365	0.000774629	-	0.000701897
V	0.008081095	0.001661698	0.001175468	0.001222891	0.000502801	0.000806053	0.004748207	1.95285E-05	0.000409173	-

From\To	A	R	N	D	C	Q	E	G	H	I	L	K	M	F	P	S	T	W	Y	V
A	-	0.000219925	0.000525429	0.000167648	0.000179318	2.37193E-05	0.000116129	0.003373827	0.000193929	0.004193185	0.002135871	9.60155E-05	0.003756276	0.000193549	0.001492416	0.013942368	0.02112342	2.75143E-05	0.000106452	0.004287303
R	0.0008334	-	0.000259014	1.80266E-05	0.000309679	0.002758549	2.27705E-05	0.000642887	0.002311204	8.2543E-05	0.001307026	0.001623536	5.02848E-05	0.000142316	0.000647061	0.000218597	9.1841E-05	0.000319166	3.13094E-05	0.000166984
N	0.000970023	0.000126186	-	0.007536936	0.000176471	0.002165568	0.00075598	0.001492985	0.006936269	0.001180365	0.001275147	0.006991677	0.001729798	0.00045541	0.002014239	0.017774641	0.010478218	0.00015408	0.003152858	4.17459E-05
D	0.000635297	1.80266E-05	0.015470552	-	5.69262E-06	0.00069023	0.006992816	0.001588621	0.001593934	0.000189849	0.000159393	2.61861E-05	5.02848E-05	0.000148008	0.000370495	0.002479706	0.001227329	0.000288901	0.000350665	4.17459E-05
C	0.002151811	0.000980649	0.001147063	1.80266E-05	-	0.000939283	2.27705E-05	0.000860724	0.001979135	0.002723919	0.002151811	2.18217E-05	0.00016594	0.002123348	0.000861009	0.00995981	0.007906671	0.000487004	0.004198593	4.17459E-05
Q	6.83115E-05	0.002096498	0.003378286	0.000524575	0.000225428	-	0.00375713	0.000191272	0.008142346	0.000363189	0.003331322	0.00534632	0.001252092	0.000574955	0.003772785	0.001946876	0.004174589	2.75143E-05	0.000638712	0.000417459
E	0.000348388	1.80266E-05	0.001228468	0.005535979	5.69262E-06	0.003913677	-	0.000791654	0.000688048	0.000140323	0.000159393	0.003604947	5.02848E-05	7.96967E-05	0.00035484	0.00196737	0.000651236	2.75143E-05	0.000216035	0.000463379
G	0.004337777	0.000218122	0.001039757	0.000538996	9.22205E-05	8.53893E-05	0.00033928	-	2.65656E-05	0.000255883	0.000207211	0.000261861	5.02848E-05	5.69262E-05	5.21824E-05	0.004529049	0.000492601	0.000156832	5.3226E-05	5.42697E-05
H	0.000498674	0.001568317	0.009661232	0.001081598	0.0004241	0.007269952	0.000589756	5.31311E-05	-	0.00053653	0.00095636	0.001466419	0.000316794	0.001440233	0.001675054	0.002787107	0.001970406	0.000101803	0.011042831	4.17459E-05
I	0.003470222	1.80266E-05	0.000529129	4.14613E-05	0.000187857	0.000104365	3.87098E-05	0.000164707	0.000172676	-	0.027606936	0.000224764	0.013707642	0.002538909	0.000568788	0.001714618	0.016189056	2.75143E-05	0.000413284	0.026871829
L	0.000915373	0.000147818	0.000296016	1.80266E-05	7.68504E-05	0.000495732	2.27705E-05	6.90705E-05	0.000159393	0.014296449	-	0.000170209	0.014225576	0.00647251	0.001101048	0.002643653	0.005552203	0.000470495	0.000726378	0.002012152
K	0.00030057	0.001341182	0.011855453	2.1632E-05	5.69262E-06	0.005811217	0.003761684	0.000637574	0.001785206	0.000850193	0.001243268	-	0.0024187	0.000193549	0.001377614	0.003804948	0.00599471	0.000346681	0.000842223	4.17459E-05
M	0.005102866	1.80266E-05	0.00127287	1.80266E-05	1.87857E-05	0.000590609	2.27705E-05	5.31311E-05	0.000167363	0.022501224	0.045092391	0.001049624	-	0.002721073	0.000516605	0.00399622	0.023210714	0.000313663	0.000657498	0.008516161
F	0.000232259	4.50666E-05	0.000296016	4.68692E-05	0.000212335	0.000239564	3.18787E-05	5.31311E-05	0.000672109	0.003681418	0.018123029	7.41938E-05	0.002403614	-	0.000474859	0.002308927	0.001486154	0.000112809	0.007670807	0.000137761
P	0.001953708	0.00022353	0.001428279	0.000127989	9.39283E-05	0.001714902	0.000154839	5.31311E-05	0.000852755	0.000899719	0.003363201	0.000576093	0.00049782	0.000518029	-	0.006107044	0.005635695	6.05315E-05	0.00026613	0.000179507
S	0.013942368	5.76852E-05	0.00962793	0.000654367	0.000829984	0.000675999	0.00065579	0.003522594	0.001083875	0.002071829	0.006168524	0.00121547	0.002941662	0.001924106	0.004665103	-	0.026241466	0.000558541	0.001070782	4.17459E-05
T	0.017282798	1.98293E-05	0.004643756	0.000264992	0.000539091	0.001185963	0.00017761	0.000313474	0.000626947	0.016005089	0.010599661	0.001566799	0.01397918	0.001013287	0.003522309	0.02147029	-	0.000145826	0.000638712	0.004496032
W	6.83115E-05	0.000209109	0.000207211	0.00018928	0.000100759	2.37193E-05	2.27705E-05	0.000302847	9.82926E-05	8.2543E-05	0.002725627	0.000274954	0.000573247	0.000233397	0.000114801	0.001386723	0.000442506	-	0.00043207	0.000116888
Y	0.000232259	1.80266E-05	0.003726105	0.000201898	0.000763381	0.000483873	0.000157116	9.03229E-05	0.009369675	0.001089568	0.003697927	0.000587004	0.001055981	0.013946922	0.00044355	0.002336252	0.001703232	0.000379698	-	4.17459E-05
V	0.007015586	7.21065E-05	3.7002E-05	1.80266E-05	5.69262E-06	0.000237193	0.000252752	6.90705E-05	2.65656E-05	0.053132934	0.007682762	2.18217E-05	0.010258103	0.000187857	0.000224384	6.83115E-05	0.008992064	7.70401E-05	3.13094E-05	-

From\To	A	R	N	D	C	Q	E	G	H	I	L	K	M	F	P	S	T	W	Y	V
A	-	0.000476734	0.000851223	0.000420681	0.001227319	4.6565E-05	0.000269275	0.003333424	0.000224489	0.002568257	0.001266278	0.000298715	0.001854202	0.000132214	0.001183906	0.009821999	0.01603169	2.27313E-05	0.000119419	0.006160282
R	0.000621887	-	0.000419617	4.52345E-05	0.002119561	0.005415505	5.2799E-05	0.000635188	0.002675414	5.05562E-05	0.000774887	0.005051002	2.4822E-05	9.72162E-05	0.000513302	0.000153995	6.9703E-05	0.000263683	3.51233E-05	0.000239933
N	0.000723836	0.000273536	-	0.01891256	0.001207838	0.004251381	0.001752926	0.001475106	0.008029316	0.000722954	0.000755987	0.021751897	0.000853876	0.000311092	0.001597859	0.012521725	0.007952479	0.000127295	0.003536915	5.99833E-05
D	0.000474062	3.90766E-05	0.025063115	-	3.89625E-05	0.00135504	0.016214563	0.001569597	0.001845113	0.000116279	9.44984E-05	8.14678E-05	2.4822E-05	0.000101105	0.000293907	0.001746882	0.000931486	0.000238679	0.000393381	5.99833E-05
C	0.001605693	0.002125765	0.001858303	4.52345E-05	-	0.001843973	5.2799E-05	0.000850417	0.002291015	0.001668356	0.001275728	6.78898E-05	8.19125E-05	0.001450466	0.000683022	0.007016401	0.006000795	0.000402344	0.004710033	5.99833E-05
Q	5.09744E-05	0.004544604	0.005473003	0.001316325	0.001542916	-	0.00871183	0.000188982	0.009425452	0.000222447	0.001975016	0.016633005	0.000618067	0.000392754	0.00299288	0.001371519	0.003168318	2.27313E-05	0.000716515	0.000599833
E	0.000259969	3.90766E-05	0.001990183	0.013891526	3.89625E-05	0.007683219	-	0.000782174	0.000796474	8.59456E-05	9.44984E-05	0.011215398	2.4822E-05	5.44411E-05	0.000281488	0.001385956	0.000494258	2.27313E-05	0.000242351	0.000665814
G	0.003236873	0.000472826	0.001684462	0.001352513	0.000631193	0.000167634	0.000786705	-	3.07519E-05	0.000156724	0.000122848	0.000814678	2.4822E-05	3.88865E-05	4.13953E-05	0.003190586	0.000373862	0.000129568	5.97096E-05	7.79783E-05
H	0.000372113	0.00339966	0.015651708	0.002714072	0.002902708	0.014272161	0.001367493	5.24949E-05	-	0.000328616	0.00056699	0.004562196	0.000156378	0.000983828	0.001328789	0.001963437	0.001495446	8.41058E-05	0.012387983	5.99833E-05
I	0.002589499	3.90766E-05	0.000857217	0.000104039	0.001285763	0.000204886	8.97582E-05	0.000162734	0.000199887	-	0.016367117	0.000699265	0.006766471	0.001734338	0.000451209	0.001207899	0.012286738	2.27313E-05	0.000463627	0.038611235
L	0.000683057	0.000320428	0.000479562	4.52345E-05	0.000525994	0.000973208	5.2799E-05	6.82433E-05	0.000184511	0.008756342	-	0.000529541	0.007022137	0.004421395	0.000873441	0.001862378	0.004213863	0.000388705	0.00081486	0.002891194
K	0.000224287	0.002907296	0.019206462	5.42814E-05	3.89625E-05	0.011408416	0.008722389	0.000629938	0.002066526	0.000520729	0.000737087	-	0.001193937	0.000132214	0.001092836	0.002680477	0.004549705	0.000286414	0.000944816	5.99833E-05
M	0.003807786	3.90766E-05	0.002062117	4.52345E-05	0.000128576	0.001159468	5.2799E-05	5.24949E-05	0.000193737	0.013781633	0.026733588	0.0032655	-	0.001858775	0.000409813	0.002815223	0.017615849	0.000259137	0.000737589	0.012236588
F	0.000173313	9.76914E-05	0.000479562	0.00011761	0.001453302	0.000470306	7.39186E-05	5.24949E-05	0.000778023	0.002254809	0.010744464	0.000230825	0.00118649	-	0.000376697	0.001626573	0.001127921	9.31983E-05	0.008605205	0.000197945
P	0.001457867	0.000484549	0.002313887	0.000321165	0.000642882	0.003366647	0.000359033	5.24949E-05	0.000987135	0.000551063	0.001993916	0.001792291	0.000245738	0.000353867	-	0.004302238	0.004277229	5.00089E-05	0.000298548	0.000257928
S	0.010403871	0.000125045	0.015597758	0.001642014	0.005680736	0.001327101	0.00152061	0.00348041	0.001254677	0.001268962	0.003657087	0.003781463	0.001452086	0.001314364	0.00370074	-	0.019916048	0.000461445	0.001201216	5.99833E-05
T	0.012896518	4.29842E-05	0.007523131	0.000664948	0.003689751	0.002328248	0.000411832	0.00030972	0.000725744	0.009802856	0.006284141	0.004874489	0.006900509	0.00069218	0.002794183	0.015125205	-	0.000120476	0.000716515	0.006460199
W	5.09744E-05	0.000453288	0.000335693	0.000474963	0.000689637	4.6565E-05	5.2799E-05	0.000299221	0.000113782	5.05562E-05	0.001615922	0.000855412	0.000282971	0.000159435	9.10697E-05	0.000976906	0.000335842	-	0.000484701	0.000167953
Y	0.000173313	3.90766E-05	0.006036488	0.000506627	0.005224874	0.000949925	0.000364313	8.92413E-05	0.010846189	0.000667342	0.002192362	0.001826236	0.000521262	0.009527192	0.00035186	0.001645822	0.001292674	0.000313692	-	5.99833E-05
V	0.005235069	0.000156306	5.99453E-05	4.52345E-05	3.89625E-05	0.00046565	0.000586069	6.82433E-05	3.07519E-05	0.032543056	0.004554821	6.78898E-05	0.005063683	0.000128325	0.000178	4.81235E-05	0.006824557	6.36477E-05	3.51233E-05	-

From\To	A	R	N	D	C	Q	E	G	H	I	L	K	M	F	P	S	T	W	Y	V
A	-	0.000219925	0.000525429	0.000167648	0.000179318	2.37193E-05	0.000116129	0.003373827	0.000193929	0.004193185	0.002135871	9.60155E-05	0.003756276	0.000193549	0.001492416	0.013942368	0.02112342	2.75143E-05	0.000106452	0.004287303
R	0.0008334	-	0.000259014	1.80266E-05	0.000309679	0.002758549	2.27705E-05	0.000642887	0.002311204	8.2543E-05	0.001307026	0.001623536	5.02848E-05	0.000142316	0.000647061	0.000218597	9.1841E-05	0.000319166	3.13094E-05	0.000166984
N	0.000970023	0.000126186	-	0.007536936	0.000176471	0.002165568	0.00075598	0.001492985	0.006936269	0.001180365	0.001275147	0.006991677	0.001729798	0.00045541	0.002014239	0.017774641	0.010478218	0.00015408	0.003152858	4.17459E-05
D	0.000635297	1.80266E-05	0.015470552	-	5.69262E-06	0.00069023	0.006992816	0.001588621	0.001593934	0.000189849	0.000159393	2.61861E-05	5.02848E-05	0.000148008	0.000370495	0.002479706	0.001227329	0.000288901	0.000350665	4.17459E-05
C	0.002151811	0.000980649	0.001147063	1.80266E-05	-	0.000939283	2.27705E-05	0.000860724	0.001979135	0.002723919	0.002151811	2.18217E-05	0.00016594	0.002123348	0.000861009	0.00995981	0.007906671	0.000487004	0.004198593	4.17459E-05
Q	6.83115E-05	0.002096498	0.003378286	0.000524575	0.000225428	-	0.00375713	0.000191272	0.008142346	0.000363189	0.003331322	0.00534632	0.001252092	0.000574955	0.003772785	0.001946876	0.004174589	2.75143E-05	0.000638712	0.000417459
E	0.000348388	1.80266E-05	0.001228468	0.005535979	5.69262E-06	0.003913677	-	0.000791654	0.000688048	0.000140323	0.000159393	0.003604947	5.02848E-05	7.96967E-05	0.00035484	0.00196737	0.000651236	2.75143E-05	0.000216035	0.000463379
G	0.004337777	0.000218122	0.001039757	0.000538996	9.22205E-05	8.53893E-05	0.00033928	-	2.65656E-05	0.000255883	0.000207211	0.000261861	5.02848E-05	5.69262E-05	5.21824E-05	0.004529049	0.000492601	0.000156832	5.3226E-05	5.42697E-05
H	0.000498674	0.001568317	0.009661232	0.001081598	0.0004241	0.007269952	0.000589756	5.31311E-05	-	0.00053653	0.00095636	0.001466419	0.000316794	0.001440233	0.001675054	0.002787107	0.001970406	0.000101803	0.011042831	4.17459E-05
I	0.003470222	1.80266E-05	0.000529129	4.14613E-05	0.000187857	0.000104365	3.87098E-05	0.000164707	0.000172676	-	0.027606936	0.000224764	0.013707642	0.002538909	0.000568788	0.001714618	0.016189056	2.75143E-05	0.000413284	0.026871829
L	0.000915373	0.000147818	0.000296016	1.80266E-05	7.68504E-05	0.000495732	2.27705E-05	6.90705E-05	0.000159393	0.014296449	-	0.000170209	0.014225576	0.00647251	0.001101048	0.002643653	0.005552203	0.000470495	0.000726378	0.002012152
K	0.00030057	0.001341182	0.011855453	2.1632E-05	5.69262E-06	0.005811217	0.003761684	0.000637574	0.001785206	0.000850193	0.001243268	-	0.0024187	0.000193549	0.001377614	0.003804948	0.00599471	0.000346681	0.000842223	4.17459E-05
M	0.005102866	1.80266E-05	0.00127287	1.80266E-05	1.87857E-05	0.000590609	2.27705E-05	5.31311E-05	0.000167363	0.022501224	0.045092391	0.001049624	-	0.002721073	0.000516605	0.00399622	0.023210714	0.000313663	0.000657498	0.008516161
F	0.000232259	4.50666E-05	0.000296016	4.68692E-05	0.000212335	0.000239564	3.18787E-05	5.31311E-05	0.000672109	0.003681418	0.018123029	7.41938E-05	0.002403614	-	0.000474859	0.002308927	0.001486154	0.000112809	0.007670807	0.000137761
P	0.001953708	0.00022353	0.001428279	0.000127989	9.39283E-05	0.001714902	0.000154839	5.31311E-05	0.000852755	0.000899719	0.003363201	0.000576093	0.00049782	0.000518029	-	0.006107044	0.005635695	6.05315E-05	0.00026613	0.000179507
S	0.013942368	5.76852E-05	0.00962793	0.000654367	0.000829984	0.000675999	0.00065579	0.003522594	0.001083875	0.002071829	0.006168524	0.00121547	0.002941662	0.001924106	0.004665103	-	0.026241466	0.000558541	0.001070782	4.17459E-05
T	0.017282798	1.98293E-05	0.004643756	0.000264992	0.000539091	0.001185963	0.00017761	0.000313474	0.000626947	0.016005089	0.010599661	0.001566799	0.01397918	0.001013287	0.003522309	0.02147029	-	0.000145826	0.000638712	0.004496032
W	6.83115E-05	0.000209109	0.000207211	0.00018928	0.000100759	2.37193E-05	2.27705E-05	0.000302847	9.82926E-05	8.2543E-05	0.002725627	0.000274954	0.000573247	0.000233397	0.000114801	0.001386723	0.000442506	-	0.00043207	0.000116888
Y	0.000232259	1.80266E-05	0.003726105	0.000201898	0.000763381	0.000483873	0.000157116	9.03229E-05	0.009369675	0.001089568	0.003697927	0.000587004	0.001055981	0.013946922	0.00044355	0.002336252	0.001703232	0.000379698	-	4.17459E-05
V	0.007015586	7.21065E-05	3.7002E-05	1.80266E-05	5.69262E-06	0.000237193	0.000252752	6.90705E-05	2.65656E-05	0.053132934	0.007682762	2.18217E-05	0.010258103	0.000187857	0.000224384	6.83115E-05	0.008992064	7.70401E-05	3.13094E-05	-
