# Supplementary material for: Identification and characterization of a novel rodent bocavirus from different rodent species in China
Source: Emerg Microbes Infect. 2018 Mar 29;7:48. doi: 10.1038/s41426-018-0052-y (PMC5874251; doi:10.1038/s41426-018-0052-y)
Supplement: Supplementary file 3 — Supplementary Table S3(PDF 141 kb) [file 41426_2018_52_MOESM3_ESM.pdf]

Supplementary Material

Identification and characterization of a novel rodent bocavirus from different rodent species in China

Chi Zhang[1], Fenglin Song[1], Leshan Xiu[1], Yang Liu, Jian Yang, Lisi Yao[2], Junping Peng[2]

[1]These authors contributed equally to this article.

[2]These authors are both corresponding authors.

Correspondence:

Junping Peng, pengjp@hotmail.com

Lisi Yao, yaolisi@hotmail.com

Supplementary information

Table S3. Maximum Likelihood fits of 24 different different nucleotide substitution models of phylogenetic analyses of partial VP1 nucleotide sequences (460-bp fragment)

Table S3. Maximum Likelihood fits of 24 different different nucleotide substitution models of phylogenetic analyses of partial VP1 nucleotide sequences (460-bp fragment)

Model	#Param	BIC	AICc	lnL	Invariant	Gamma	R	Freq A	Freq T	Freq C	Freq G	A=>T	A=>C	A=>G	T=>A	T=>C	T=>G	C=>A	C=>T	C=>G	G=>A	G=>T	G=>C
HKY+G	112	3380.159895	2470.138601	-1122.564615	n/a	0.242850069	3.131198762	0.330369194	0.185271933	0.183287019	0.301071854	0.02	0.02	0.22	0.04	0.13	0.04	0.04	0.14	0.04	0.24	0.02	0.02
K2+G	109	3382.313403	2496.641485	-1138.842672	n/a	0.247405023	3.092500569	0.25	0.25	0.25	0.25	0.03	0.03	0.19	0.03	0.19	0.03	0.03	0.19	0.03	0.19	0.03	0.03
TN93+G	113	3388.516863	2470.379432	-1121.675998	n/a	0.245832219	3.06928797	0.330369194	0.185271933	0.183287019	0.301071854	0.02	0.02	0.19	0.04	0.17	0.04	0.04	0.17	0.04	0.2	0.02	0.02
HKY+G+I	113	3390.306745	2472.169314	-1122.570939	0	0.242861931	3.131202963	0.330369194	0.185271933	0.183287019	0.301071854	0.02	0.02	0.22	0.04	0.13	0.04	0.04	0.14	0.04	0.24	0.02	0.02
HKY	111	3390.572162	2488.667166	-1132.83785	n/a	n/a	2.919825685	0.330369194	0.185271933	0.183287019	0.301071854	0.03	0.03	0.22	0.05	0.13	0.04	0.05	0.13	0.04	0.24	0.03	0.03
T92+G	110	3391.338263	2497.549726	-1138.288001	n/a	0.247743954	3.092452841	0.257820564	0.257820564	0.242179436	0.242179436	0.03	0.03	0.18	0.03	0.18	0.03	0.03	0.19	0.03	0.19	0.03	0.03
K2	108	3392.425256	2514.870119	-1148.9657	n/a	n/a	2.924095396	0.25	0.25	0.25	0.25	0.03	0.03	0.19	0.03	0.19	0.03	0.03	0.19	0.03	0.19	0.03	0.03
K2+G+I	110	3392.451512	2498.662974	-1138.844626	0	0.247408634	3.092501993	0.25	0.25	0.25	0.25	0.03	0.03	0.19	0.03	0.19	0.03	0.03	0.19	0.03	0.19	0.03	0.03
T92+I	110	3395.247276	2501.458738	-1140.242508	0.500749598	n/a	2.994387374	0.257820564	0.257820564	0.242179436	0.242179436	0.03	0.03	0.18	0.03	0.18	0.03	0.03	0.19	0.03	0.19	0.03	0.03
K2+I	109	3397.037112	2511.365194	-1146.204527	0.184046902	n/a	2.939170113	0.25	0.25	0.25	0.25	0.03	0.03	0.19	0.03	0.19	0.03	0.03	0.19	0.03	0.19	0.03	0.03
TN93+G+I	114	3398.651366	2472.397958	-1121.676148	0	0.245832369	3.069299743	0.330369194	0.185271933	0.183287019	0.301071854	0.02	0.02	0.19	0.04	0.17	0.04	0.04	0.17	0.04	0.2	0.02	0.02
TN93	112	3398.738088	2488.716794	-1131.853711	n/a	n/a	2.919920035	0.330369194	0.185271933	0.183287019	0.301071854	0.03	0.02	0.19	0.05	0.17	0.04	0.05	0.17	0.04	0.2	0.03	0.02
HKY+I	112	3400.706203	2490.684909	-1132.837769	0.00001	n/a	2.919826637	0.330369194	0.185271933	0.183287019	0.301071854	0.03	0.03	0.22	0.05	0.13	0.04	0.05	0.13	0.04	0.24	0.03	0.03
T92	109	3401.403991	2515.732073	-1148.387966	n/a	n/a	2.92392567	0.257820564	0.257820564	0.242179436	0.242179436	0.03	0.03	0.18	0.03	0.18	0.03	0.03	0.19	0.03	0.19	0.03	0.03
T92+G+I	111	3401.437457	2499.532461	-1138.270497	0.00001	0.247716876	3.092448746	0.257820564	0.257820564	0.242179436	0.242179436	0.03	0.03	0.18	0.03	0.18	0.03	0.03	0.19	0.03	0.19	0.03	0.03
TN93+I	113	3408.872008	2490.734577	-1131.85357	0.00001	n/a	2.919920588	0.330369194	0.185271933	0.183287019	0.301071854	0.03	0.02	0.19	0.05	0.17	0.04	0.05	0.17	0.04	0.2	0.03	0.02
GTR+I	116	3414.122737	2471.637861	-1119.277631	0.572701115	n/a	2.543863338	0.330369194	0.185271933	0.183287019	0.301071854	0.04	0.04	0.19	0.06	0.15	0.04	0.07	0.15	0.02	0.21	0.02	0.01
GTR+G	116	3415.059667	2472.574791	-1119.746096	n/a	0.256564423	2.446591392	0.330369194	0.185271933	0.183287019	0.301071854	0.03	0.04	0.19	0.06	0.14	0.04	0.07	0.14	0.03	0.21	0.02	0.02
GTR	115	3422.226792	2487.85757	-1128.39676	n/a	n/a	2.487294492	0.330369194	0.185271933	0.183287019	0.301071854	0.04	0.04	0.19	0.06	0.15	0.04	0.07	0.15	0.02	0.21	0.02	0.01
GTR+G+I	117	3425.185724	2474.585355	-1119.742023	0	0.256556557	2.446598257	0.330369194	0.185271933	0.183287019	0.301071854	0.03	0.04	0.19	0.06	0.14	0.04	0.07	0.14	0.03	0.21	0.02	0.02
JC+G	108	3432.476575	2554.921437	-1168.991359	n/a	0.26230396	0.5	0.25	0.25	0.25	0.25	0.08	0.08	0.08	0.08	0.08	0.08	0.08	0.08	0.08	0.08	0.08	0.08
JC+I	108	3439.953419	2562.398281	-1172.729781	0.379857988	n/a	0.5	0.25	0.25	0.25	0.25	0.08	0.08	0.08	0.08	0.08	0.08	0.08	0.08	0.08	0.08	0.08	0.08
JC	107	3441.686082	2572.247886	-1178.663214	n/a	n/a	0.5	0.25	0.25	0.25	0.25	0.08	0.08	0.08	0.08	0.08	0.08	0.08	0.08	0.08	0.08	0.08	0.08
JC+G+I	109	3442.616281	2556.944363	-1168.994111	0.00001	0.262314811	0.5	0.25	0.25	0.25	0.25	0.08	0.08	0.08	0.08	0.08	0.08	0.08	0.08	0.08	0.08	0.08	0.08

Table. Maximum Likelihood fits of 24 different nucleotide substitution models
NOTE.-- Models with the lowest BIC scores (Bayesian Information Criterion) are considered to describe the substitution pattern the best. For each model, AICc value (Akaike Information Criterion, corrected), Maximum Likelihood value (lnL), and the number of parameters (including branch lengths) are also presented [1]. Non-uniformity of evolutionary rates among sites may be modeled by using a discrete Gamma distribution (+G) with 5 rate categories and by assuming that a certain fraction of sites are evolutionarily invariable (+I). Whenever applicable, estimates of gamma shape parameter and/or the estimated fraction of invariant sites are shown. Assumed or estimated values of transition/transversion bias (R) are shown for each model, as well. They are followed by nucleotide frequencies (f) and rates of base substitutions (r) for each nucleotide pair. Relative values of instantaneous r should be considered when evaluating them. For simplicity, sum of r values is made equal to 1 for each model. For estimating ML values, a tree topology was automatically computed. The analysis involved 55 nucleotide sequences. Codon positions included were 1st+2nd+3rd+Noncoding. All positions containing gaps and missing data were eliminated. There were a total of 458 positions in the final dataset. Evolutionary analyses were conducted in MEGA6 [2].

Abbreviations: GTR: General Time Reversible; HKY: Hasegawa-Kishino-Yano; TN93: Tamura-Nei; T92: Tamura 3-parameter; K2: Kimura 2-parameter; JC: Jukes-Cantor.

From\To	A	T	C	G
A	-	0.024830035	0.024564018	0.220372909
T	0.044275885	-	0.134158982	0.040349473
C	0.044275885	0.135611862	-	0.040349473
G	0.241817424	0.024830035	0.024564018	-

From\To	A	T	C	G
A	-	0.030543673	0.030543673	0.188912654
T	0.030543673	-	0.188912654	0.030543673
C	0.030543673	0.188912654	-	0.030543673
G	0.188912654	0.030543673	0.030543673	-

From\To	A	T	C	G
A	-	0.024263331	0.024003385	0.1862483
T	0.043265361	-	0.172793501	0.039428562
C	0.043265361	0.174664776	-	0.039428562
G	0.204372145	0.024263331	0.024003385	-

From\To	A	T	C	G
A	-	0.024830011	0.024563994	0.220372988
T	0.044275841	-	0.13415903	0.040349433
C	0.044275841	0.135611911	-	0.040349433
G	0.241817511	0.024830011	0.024563994	-

From\To	A	T	C	G
A	-	0.026120695	0.025840851	0.216178195
T	0.046577336	-	0.131605317	0.042446829
C	0.046577336	0.133030543	-	0.042446829
G	0.237214522	0.026120695	0.025840851	-

From\To	A	T	C	G
A	-	0.031476217	0.029566658	0.183046119
T	0.031476217	-	0.183046119	0.029566658
C	0.031476217	0.194868129	-	0.029566658
G	0.194868129	0.031476217	0.029566658	-

From\To	A	T	C	G
A	-	0.031854475	0.031854475	0.186291049
T	0.031854475	-	0.186291049	0.031854475
C	0.031854475	0.186291049	-	0.031854475
G	0.186291049	0.031854475	0.031854475	-

From\To	A	T	C	G
A	-	0.030543663	0.030543663	0.188912675
T	0.030543663	-	0.188912675	0.030543663
C	0.030543663	0.188912675	-	0.030543663
G	0.188912675	0.030543663	0.030543663	-

From\To	A	T	C	G
A	-	0.032249173	0.030292722	0.181593992
T	0.032249173	-	0.181593992	0.030292722
C	0.032249173	0.193322217	-	0.030292722
G	0.193322217	0.032249173	0.030292722	-

From\To	A	T	C	G
A	-	0.031732572	0.031732572	0.186534856
T	0.031732572	-	0.186534856	0.031732572
C	0.031732572	0.186534856	-	0.031732572
G	0.186534856	0.031732572	0.031732572	-

From\To	A	T	C	G
A	-	0.024263266	0.024003321	0.186248622
T	0.043265245	-	0.172793513	0.039428457
C	0.043265245	0.174664788	-	0.039428457
G	0.204372499	0.024263266	0.024003321	-

From\To	A	T	C	G
A	-	0.02524762	0.024977129	0.186516319
T	0.045020505	-	0.167229893	0.041028059
C	0.045020505	0.169040917	-	0.041028059
G	0.204666246	0.02524762	0.024977129	-

From\To	A	T	C	G
A	-	0.026120689	0.025840845	0.216178215
T	0.046577325	-	0.131605329	0.04244682
C	0.046577325	0.133030555	-	0.04244682
G	0.237214544	0.026120689	0.025840845	-

From\To	A	T	C	G
A	-	0.032828414	0.030836822	0.180505792
T	0.032828414	-	0.180505792	0.030836822
C	0.032828414	0.192163735	-	0.030836822
G	0.192163735	0.032828414	0.030836822	-

From\To	A	T	C	G
A	-	0.031476249	0.029566688	0.18304606
T	0.031476249	-	0.18304606	0.029566688
C	0.031476249	0.194868066	-	0.029566688
G	0.194868066	0.031476249	0.029566688	-

From\To	A	T	C	G
A	-	0.025247616	0.024977126	0.186516319
T	0.045020498	-	0.167229913	0.041028053
C	0.045020498	0.169040937	-	0.041028053
G	0.204666246	0.025247616	0.024977126	-

From\To	A	T	C	G
A	-	0.035278766	0.038572669	0.190257432
T	0.062907626	-	0.14806293	0.038650591
C	0.069526045	0.149666384	-	0.021458202
G	0.208771407	0.023784587	0.01306336	-

From\To	A	T	C	G
A	-	0.034937575	0.038077074	0.192441961
T	0.062299229	-	0.140718746	0.03820404
C	0.06863275	0.142242666	-	0.02969182
G	0.211168513	0.023509791	0.018075835	-

From\To	A	T	C	G
A	-	0.035450082	0.039710626	0.188549709
T	0.06321311	-	0.14754705	0.039317939
C	0.071577178	0.149144917	-	0.021380545
G	0.206897505	0.024195256	0.013016083	-

From\To	A	T	C	G
A	-	0.034937683	0.038077053	0.192443498
T	0.062299421	-	0.140717203	0.038204142
C	0.068632713	0.142241106	-	0.029691494
G	0.211170199	0.023509854	0.018075637	-

From\To	A	T	C	G
A	-	0.083333333	0.083333333	0.083333333
T	0.083333333	-	0.083333333	0.083333333
C	0.083333333	0.083333333	-	0.083333333
G	0.083333333	0.083333333	0.083333333	-

From\To	A	T	C	G
A	-	0.083333333	0.083333333	0.083333333
T	0.083333333	-	0.083333333	0.083333333
C	0.083333333	0.083333333	-	0.083333333
G	0.083333333	0.083333333	0.083333333	-

From\To	A	T	C	G
A	-	0.083333333	0.083333333	0.083333333
T	0.083333333	-	0.083333333	0.083333333
C	0.083333333	0.083333333	-	0.083333333
G	0.083333333	0.083333333	0.083333333	-

From\To	A	T	C	G
A	-	0.083333333	0.083333333	0.083333333
T	0.083333333	-	0.083333333	0.083333333
C	0.083333333	0.083333333	-	0.083333333
G	0.083333333	0.083333333	0.083333333	-
