# Supplementary material for: Identification and characterization of a novel rodent bocavirus from different rodent species in China
Source: Emerg Microbes Infect. 2018 Mar 29;7:48. doi: 10.1038/s41426-018-0052-y (PMC5874251; doi:10.1038/s41426-018-0052-y)
Supplement: Supplementary file 4 — Supplementary Figure S1(PDF 109 kb) [file 41426_2018_52_MOESM4_ESM.pdf]

Supplementary Material

Identification and characterization of a novel rodent bocavirus from different rodent species in China

Chi Zhang[1], Fenglin Song[1], Leshan Xiu[1], Yang Liu, Jian Yang, Lisi Yao[2], Junping Peng[2]

[1]These authors contributed equally to this article.

[2]These authors are both corresponding authors.

Correspondence:

Junping Peng, pengjp@hotmail.com

Lisi Yao, yaolisi@hotmail.com

Supplementary information

Figure S1, Sequence alignments showing the short VP1u region of RoBoVs and a lack of phospholipase A_2 motif.

Figure S1

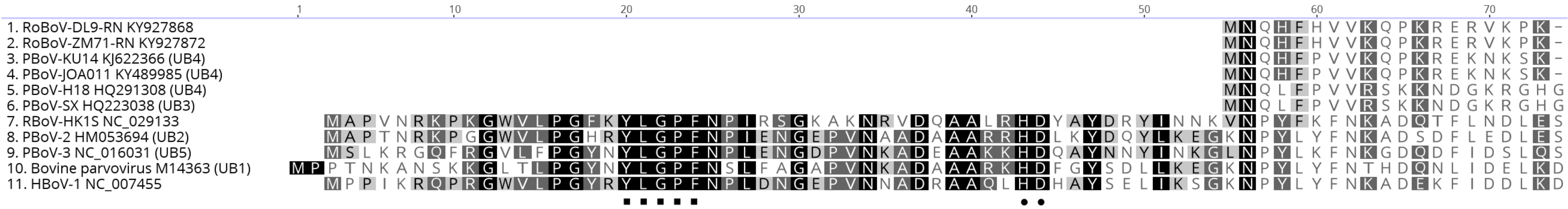
1. RoBoV-DL9-RN KY927868
2. RoBoV-ZM71-RN KY927872
3. PBoV-KU14 KJ622366 (UB4)
4. PBoV-JOA011 KY489985 (UB4)
5. PBoV-H18 HQ291308 (UB4)
6. PBoV-SX HQ223038 (UB3)
7. RBoV-HK1S NC_029133
8. PBoV-2 HM053694 (UB2)
9. PBoV-3 NC_016031 (UB5)
10. Bovine parvovirus M14363 (UB1)
11. HBoV-1 NC_007455

Figure legend

Figure S1. Amino acid sequences of VP1 protein were aligned to show the short VP1u region of RoBoVs and a lack of phospholipase A_2 (PLA_2) related motifs. The PLA_2 related Ca^{2+} binding loop (the YXGXG motif) was underlined by filled squares and residuals in the catalytic site (the HDXXY motif) were underlined by filled circles.
