# Supplementary material for: Identification and characterization of a novel rodent bocavirus from different rodent species in China
Source: Emerg Microbes Infect. 2018 Mar 29;7:48. doi: 10.1038/s41426-018-0052-y (PMC5874251; doi:10.1038/s41426-018-0052-y)
Supplement: Supplementary file 5 — Supplementary Figure S2(PDF 625 kb) [file 41426_2018_52_MOESM5_ESM.pdf]

Supplementary Material

Identification and characterization of a novel rodent bocavirus from different rodent species in China

Chi Zhang[1], Fenglin Song[1], Leshan Xiu[1], Yang Liu, Jian Yang, Lisi Yao[2], Junping Peng[2]

[1]These authors contributed equally to this article.

[2]These authors are both corresponding authors.

Correspondence:

Junping Peng, pengjp@hotmail.com

Lisi Yao, yaolisi@hotmail.com

Supplementary information

Figure S2 (A), Conserved RNA splicing sites of RoBoV and HBoV.

Figure S2 (B), Genomic organization of rodent bocavirus (RoBoV) with possible extended NS1 protein generated by RNA splicing.

Figure S2 (C), Actual reading frames of extended NS1 of RoBoV isolate DL9-RN.

Figure legend

Figure S2. (A) Conserved RNA splicing sites of RoBoV and HBoV. Sequences of RoBoV and HBoV (NC_007455) are aligned to show the conserved RNA splicing sites (sequences in box). The splice donor site (GT) and splice acceptor site (AG) are highlighted in red. Shaded areas on the sequences represent the predicted exons. The stop codons of protein NS1 are underlined. An online tool NetGene 2.4.2 was used to predict the splice sites (http://www.cbs.dtu.dk/services/NetGene2/). The score represented the confidence of prediction and “H” means it is a highly confident donor/acceptor site. (B) Genomic organization of rodent bocavirus (RoBoV) compared with that of GBoV, HBoV and PBoV. Different colored boxes represent four ORFs of bocaparvovirus. Orange boxes show the possible extended NS1 protein generated by RNA splicing. The illustration was constructed by geneious software (Biomatters Ltd, Auckland, New Zealand). (C) Predicted reading frames of extended NS1 of RoBoV isolate DL9-RN. Grey shaded areas represent the predicted introns. Yellow shaded areas represent the exons.

(A)

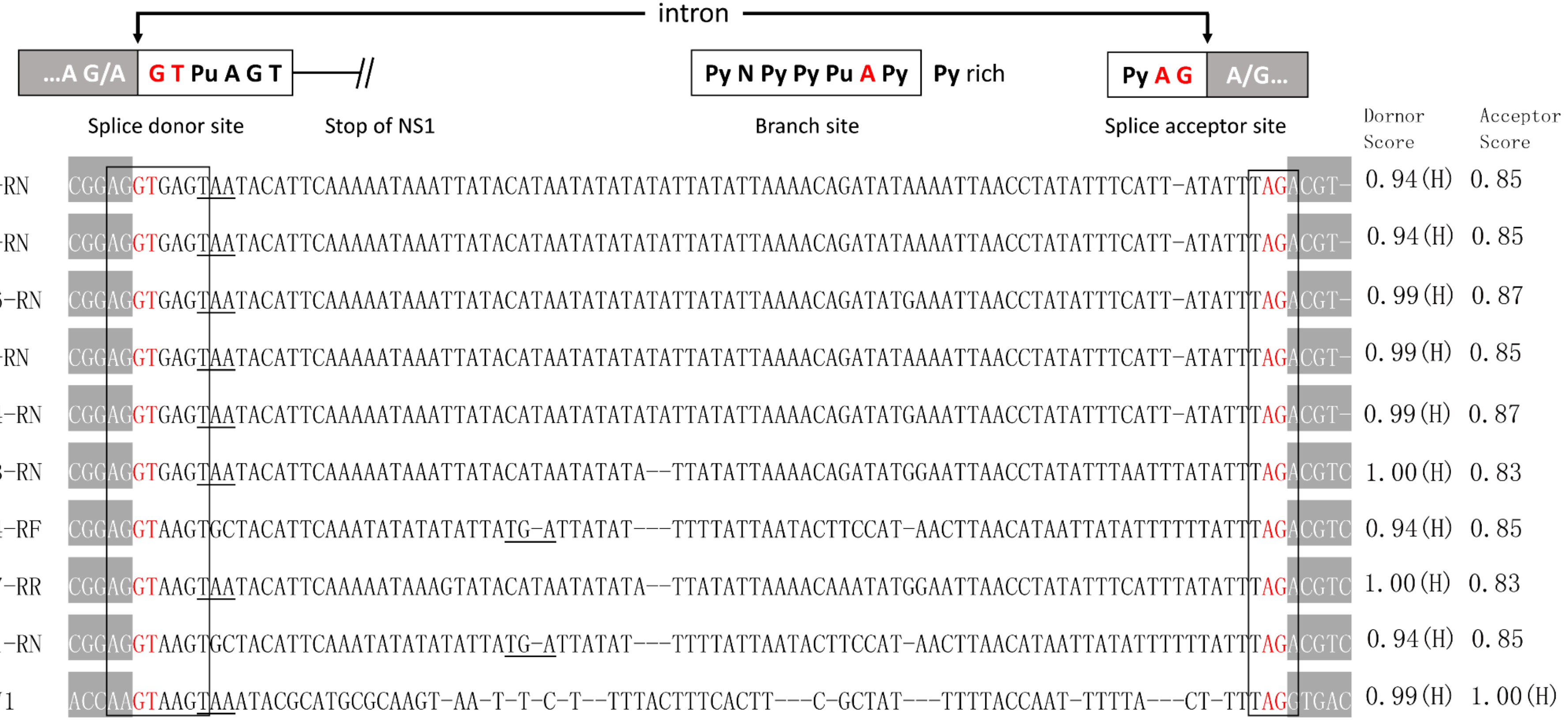

intron
...A G/A
G T Pu A G T
Py N Py Py Pu A Py
Py rich
Py A G
A/G...
Splice donor site
Stop of NS1
Branch site
Splice acceptor site
Dornor Score
Acceptor Score
DL8-RN CGGAGGTGAGTAATACATTCAAAAATAAATTATACATAATATATATATTATATTAAAACAGATATAAAATTAACCTATATTTCATT-ATATTTAGACGT- 0.94(H) 0.85
DL9-RN CGGAGGTGAGTAATACATTCAAAAATAAATTATACATAATATATATATTATATTAAAACAGATATAAAATTAACCTATATTTCATT-ATATTTAGACGT- 0.94(H) 0.85
DL16-RN CGGAGGTGAGTAATACATTCAAAAATAAATTATACATAATATATATATTATATTAAAACAGATATGAAATTAACCTATATTTCATT-ATATTTAGACGT- 0.99(H) 0.87
DL7-RN CGGAGGTGAGTAATACATTCAAAAATAAATTATACATAATATATATATTATATTAAAACAGATATAAAATTAACCTATATTTCATT-ATATTTAGACGT- 0.99(H) 0.85
DL14-RN CGGAGGTGAGTAATACATTCAAAAATAAATTATACATAATATATATATTATATTAAAACAGATATGAAATTAACCTATATTTCATT-ATATTTAGACGT- 0.99(H) 0.87
DL23-RN CGGAGGTGAGTAATACATTCAAAAATAAATTATACATAATATATA--TTATATTAAAACAGATATGGAATTAACCTATATTTAATTTATATTTAGACGTC 1.00(H) 0.83
ZM14-RF CGGAGGTAAGTGCTACATTCAAATATATATATTATG-ATTATAT---TTTTATTAATACTTCCAT-AACTTAACATAATTATATTTTTTATTTAGACGTC 0.94(H) 0.85
ZM37-RR CGGAGGTAAGTAATACATTCAAAAATAAAGTATACATAATATATA--TTATATTAAAACAAATATGGAATTAACCTATATTTCATTTATATTTAGACGTC 1.00(H) 0.83
ZM71-RN CGGAGGTAAGTGCTACATTCAAATATATATATTATG-ATTATAT---TTTTATTAATACTTCCAT-AACTTAACATAATTATATTTTTTATTTAGACGTC 0.94(H) 0.85
HBoV1 ACCAAGTAAGTAAATACGCATGCGCAAGT-AA-T-T-C-T--TTTACTTTCACTT---C-GCTAT---TTTTACCAAT-TTTTA---CT-TTTAGGTGAC 0.99(H) 1.00(H)

(B)

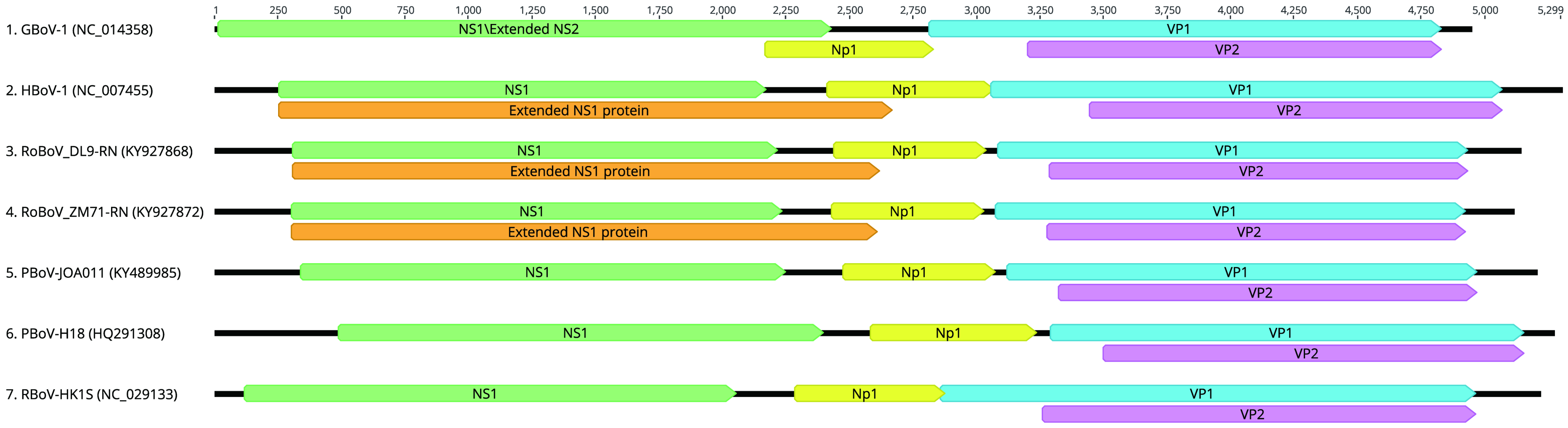

1 250 500 750 1,000 1,250 1,500 1,750 2,000 2,250 2,500 2,750 3,000 3,250 3,500 3,750 4,000 4,250 4,500 4,750 5,000 5,299
1. GBoV-1 (NC_014358)
NS1\Extended NS2
VP1
Np1
VP2
2. HBoV-1 (NC_007455)
NS1
Np1
VP1
Extended NS1 protein
VP2
3. RoBoV_DL9-RN (KY927868)
NS1
Np1
VP1
Extended NS1 protein
VP2
4. RoBoV_ZM71-RN (KY927872)
NS1
Np1
VP1
Extended NS1 protein
VP2
5. PBoV-JOA011 (KY489985)
NS1
Np1
VP1
VP2
6. PBoV-H18 (HQ291308)
NS1
Np1
VP1
VP2
7. RBoV-HK1S (NC_029133)
NS1
Np1
VP1
VP2

(C)

ATGCCTCTGATCAACTTTCAAGCAGCATTTGAGAGCTTCGGGGGAATTGCATATACCTATATTTTAAGATTTCCTAAATTTCCTACGGATGACTATCATAATATGCTACAGCAGTGCTTAGGAG
ATAGCGAAAACAGTTGCTTGCATGAATCGGAAGAATGTGGAAAACACTATCCAGAATTTCCAAATGAATGTTCCGATTTAGCCGATTATCAACGAAAAACATATGAACACAACATTTCAGTG
TTCGCAGAAACAGCTGTAAGAAAGGTGTTTGAAAATCAGCAAATGACAAGAACACCATCTTACACGAGCTTTGTTCAAGTTGAGCACGGACAAAATCTCCATGTTCACTTAGTGCTGTCCG
GAGACGGACTCACAAAATATACAGCGAAAAACTTTAGATCAAAACTAGCTGTATATTTTTATGCACAACTTGAACAAAAACAAAAAGAAGAGCTGCAAGAGCTGTATGGAACGCCGAGAT
GGGCACCATACACAAAACCAATTTCAGACGCGCTAACTAAATCTCAAGAAGGAAGCACAGACTTCTGCACTGTACTACAGTACAAAAGCAGAAATGGAGAAATGTACTCGTGCCGTGTGG
ATGCGCGAAGCTTCATTGCTAACTACATGCTGCCAAAGAACCTAGAAATCAACAACAAAATATGGTCAAATACTTACAAAGTTCCAGGGCCAATGGCAGACTCATTTATTTTAAATGGAAAA
ACATACACATACAGCAGCATCAACTCAATGCAAATCTTGCCGCACATTAGAAGAGATCTTAAGGAATGGCTGGAAGATTTGCATGGAATAAATGCTGACGAGCCGGTCTTTACTGGAGATCC
GCTTTCTGACCTACCAAAGGTAAGAAAAGCACAATGGGAAAAAACTGCGCAACCGGGAGGAAAAATGTCTAAACGAGAAGGCTTAGTATTAGATTGCATGAATAGAGCCATTGAATCTGA
CTGCTTAACATACGAACAATTAGTAGACAAACACCCGGAAATAATAATCATGATGGAAAGCCAAGCAGGTGGAAGCAAGCTGATAGAACAGACTTTGAACATGGTGCATATAAAACTAACA
CAAAAGTATACAGCCATGTCATATATGATGAAACGGTTTCCAACCTTTAACCTGCAATTAGACAATAAAGCAATTAGACTATTGAATTATCAAGGCTATAATTGCTGGCAAGTTGGACATTGGC
TGTGTACTGTGTTAGATAAGAAGAGCGGAAAGCAGAACACCATAAGTTTTTATGGTCCTGCCAGTACAGGAAAGACCAACCTGGCCAAAGCCATTGTAAATTGTGTAAATCTATTTGGCAAT
GTGAATCATTTAAACAAAAACTTTGTGTTTAACGATTGCTCGAACAAGCTGGTTGTATGGTGGGAAGAAGCACTAATGCACACCGACTGGGTAGAACCTGCTAAATGTGTTTTGGGTGGAA
CAACAGTAAGAATTGACAGAAAACACAAAGATTCGCAACTGCTGCCGCAGACTCCATGCATCATATCTACAAACAACAACATTTACGAGTGTGTAGGTGGAAACCACGTATCTCATGTGCAC
TGCAAGCCATTAAAGGACAGAGTGGTTCAGCTAAATTTTATGAAAAGCTTACCACAAAACTTCGGAGAAATACAAGAGCAAGAGATTGCAGATTGGTTGCTAACCTGCAAAGAGAAATAC
ACATGCACATTGGACGGATACTGCACTACATGGAAAACGAAGAACGTGACCAATGACATGCCGTTGGCTAAGTTATGCCCTTCTCATTCGCAGGACTTCACGCTCCATGAGAACGGAACGT
GTACCCGCTGCGGTGGTTATTTGCCTCTTGATACTGACCCTCAATTTGATCTCGATAGTGATTTTGGGGACGACGGAGGTGAGTAATACATTCAAAAATAAATTATACATAATATATATATTATAT
TAAAACAGATATAAAATTAACCTATATTTCATTATATTTAGACGTCCCTACTGCTGAAGTCATCGGAAACAATCTCGGAAGTCCTGAGCCTGGTATCACAGAACCAGACAGAAAAAAAATAAA
AAAACAGGTACAACAAAGGCTGGAATTTCTTGAAAAGTGGGCATCACAACCTCGAGACAGCTATGAGTGGGCAGAATACATACAGGAAAGAGACCAACTATTCAGAGAGGACAACTCGG
AGGAGTCCTTTGAAGGAGAGAAAGGAGAAACACCACGAGAAGGAACGGAGACGGAGCAGGAGCAGGAGCCCCTTAAGAAAGATTCAGAACAGAATGGAGGAATTGAAGAAACAGC
CTTTTAA
