# Supplementary material for: Identification and characterization of a novel rodent bocavirus from different rodent species in China
Source: Emerg Microbes Infect. 2018 Mar 29;7:48. doi: 10.1038/s41426-018-0052-y (PMC5874251; doi:10.1038/s41426-018-0052-y)

*Supplementary Material*

# Identification and characterization of a novel rodent bocavirus from different rodent species in China

Chi Zhang[1], Fenglin Song[1], Leshan Xiu[1], Yang Liu, Jian Yang, Lisi Yao[2], Junping Peng[2]

[1]These authors contributed equally to this article.

[2]These authors are both corresponding authors.

Correspondence:

Junping Peng, pengjp@hotmail.com

Lisi Yao, yaolisi@hotmail.com

Supplementary information

Figure S5, Alignments of nucleotide sequences of 460bp partial VP1 among different isolates of RoBoV.

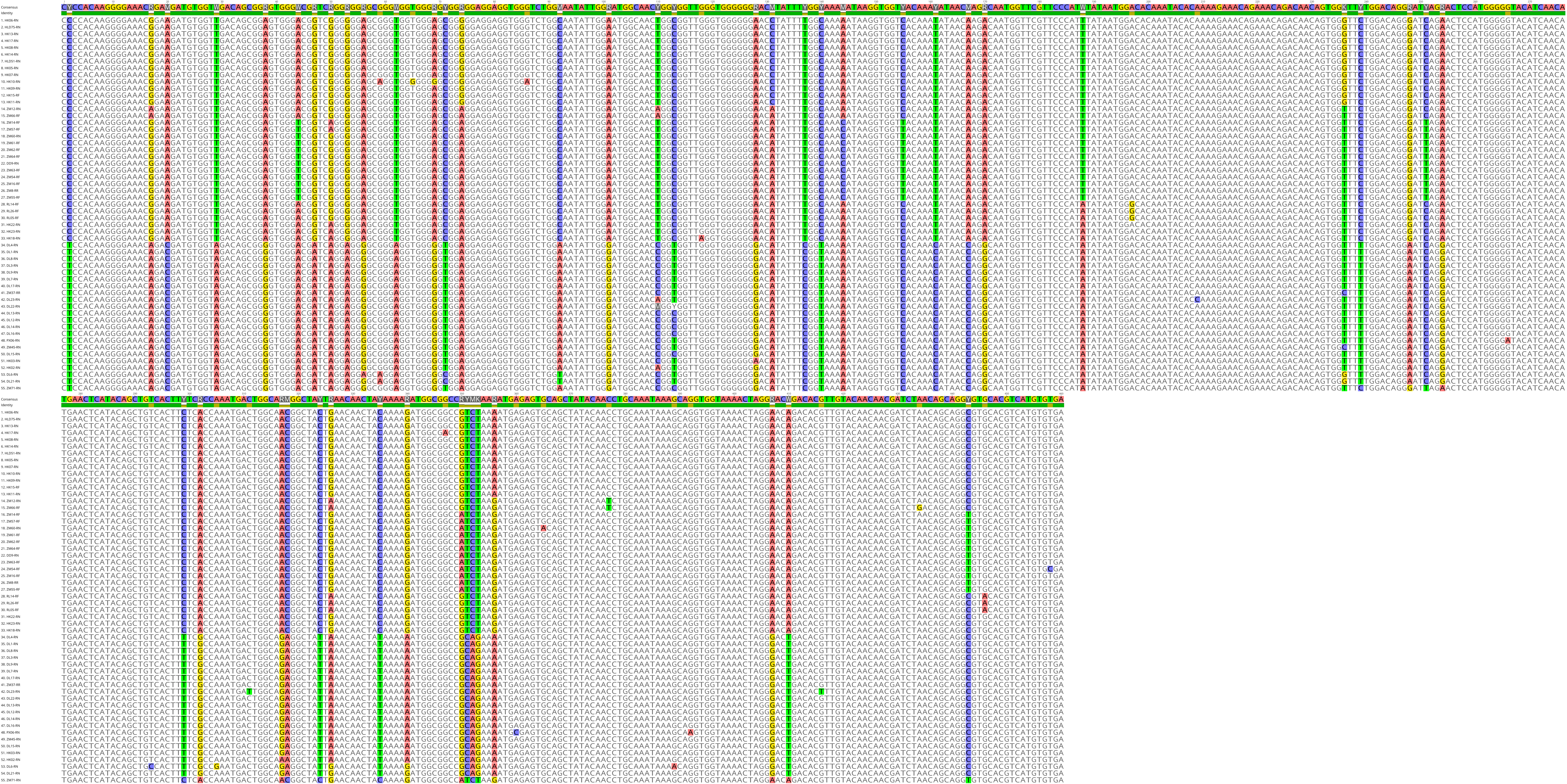

Supplement: Supplementary file 8 — Supplementary Figure S5(PDF 758 kb) [file 41426_2018_52_MOESM8_ESM.pdf]
